# Ugi bisamides based on pyrrolyl-β-chlorovinylaldehyde and their unusual transformations

**DOI:** 10.3762/bjoc.20.156

**Published:** 2024-07-26

**Authors:** Alexander V Tsygankov, Vladyslav O Vereshchak, Tetiana O Savluk, Serhiy M Desenko, Valeriia V Ananieva, Oleksandr V Buravov, Yana I Sakhno, Svitlana V Shishkina, Valentyn A Chebanov

**Affiliations:** 1 Institute of Functional Materials Chemistry, State Scientific Institution “Institute for Single Crystals” of National Academy of Sciences of Ukraine, Nauky Ave., 60, 61072, Kharkiv, Ukraine; 2 National Technical University “Kharkiv Polytechnic Institute”, Kyrpychova st., 2, Kharkiv, 61002, Ukraine; 3 Enamine Ltd., Winston Churchill Street 78, Kyiv 02094, Ukraine; 4 Faculty of Chemistry, V. N. Karazin Kharkiv National University, Svobody sq., 4, 61022, Kharkiv, Ukraine

**Keywords:** convertible isocyanides, multicomponent reaction, post-Ugi transformation, pyrrole derivative, Ugi reaction

## Abstract

By one-pot four- and three-component Ugi reactions involving convertible isocyanides and unexplored pyrrole-containing β-chlorovinylaldehyde, a small library of 20 bisamides with unusual behavior in post-Ugi transformations was prepared and characterized. Surprisingly, a well-documented approach to obtain peptide-containing carboxylic acids through acid hydrolysis of the convertible isocyanide moiety in the Ugi bisamides proceeded in an unexpected manner in our case, leading to the formation of derivatives of amides of heterylidenepyruvic acid. An optimized synthetic protocol for this transformation was elaborated and a plausible sequence involving the elimination of the 2-chloroacetamide moiety and the conversion of the β-chlorovinyl fragment into a vinyl one is provided.

## Introduction

One of the keys to the development of mankind is the constant search for new substances and advanced materials. Today, we have powerful tools at our disposal that allow us to create entire libraries of structurally complex organic compounds [1–6] to expand and systematically explore the chemical space within the concepts of molecular diversity chemistry (diversity oriented synthesis) [6] and biologically oriented synthesis [7].

**Scheme 1:**
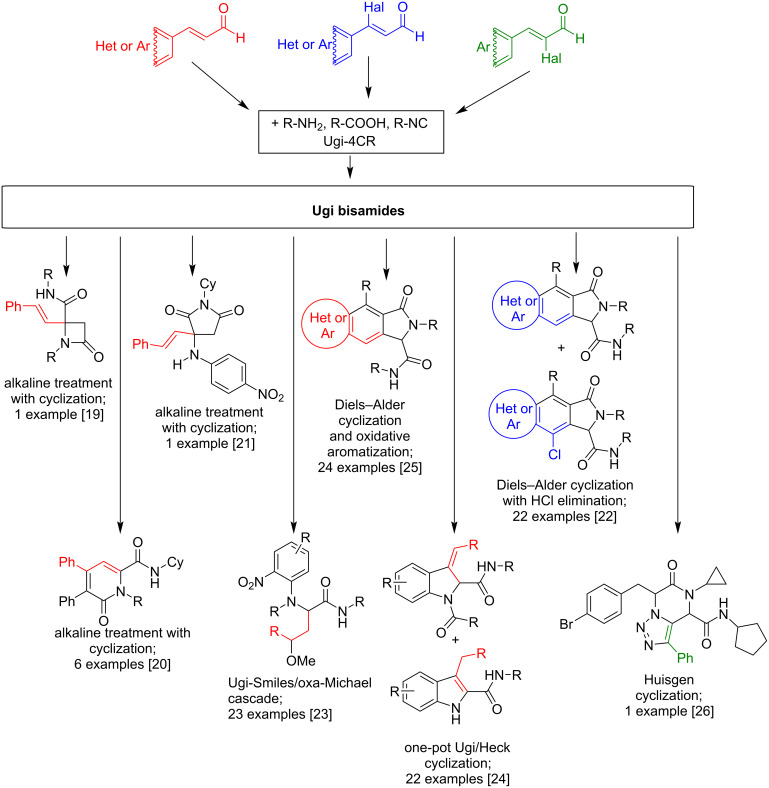
The use of α,β-unsaturated aldehydes in the Ugi reaction.

Among the multicomponent processes, the four-component Ugi reaction (Ugi-4CR) [8–13] is characterized by the greatest versatility, through the variability of the starting components leading to a variety of possible products [12–17]. The Ugi-4CR has been used for the synthesis of numerous natural substances, e.g., bicyclomycin, furanomycin, penicillin, etc. [15,18]. Further, the application of reagents with additional functional groups in the Ugi reaction makes it possible to further increase the complexity of the product structures, also due to possible post-transformation reactions. For example, if an unsaturated bond is present in the aldehyde component, after the formation of the expected Ugi bisamide products, subsequent post-transformations allow the synthesis of products of intramolecular cyclization [19–22] and/or products of a tandem combination of several reactions (Scheme 1) [22–26].

**Scheme 2:**
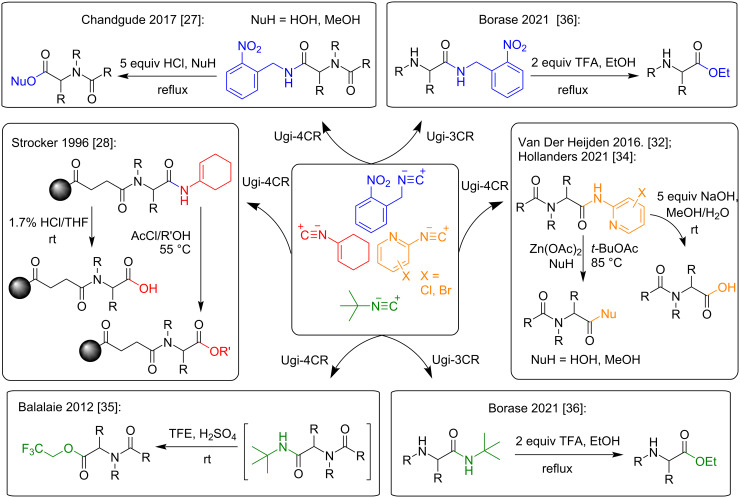
Comparison of isocyanide conversion conditions.

At the same time, the use of so-called convertible isocyanides [27–36] in Ugi-4CR makes it possible to obtain carboxylic acids or esters after hydrolysis of the secondary amide group in the Ugi products (Scheme 2) [27–29,31–32,34–36]. Ugi bisamides modified in this way may be subsequently used as acid components in tandem combinations of various multicomponent processes such as Ugi and Ugi, azido-Ugi and Ugi, Ugi and Passerini, Groebke–Blackburn–Bienaymé and Ugi, etc. [15,17,37–38].

The creation of hybrid molecules by using primary and post-modified Ugi products in combination with other isocyanide MCRs is effective and one promising direction to increase the diversity of new peptidomimetics is the use of, for example, α,β-unsaturated aldehydes including those containing a halogen atom in the β-position, in the Ugi-4CR reaction [1,39].

**Figure 1:**
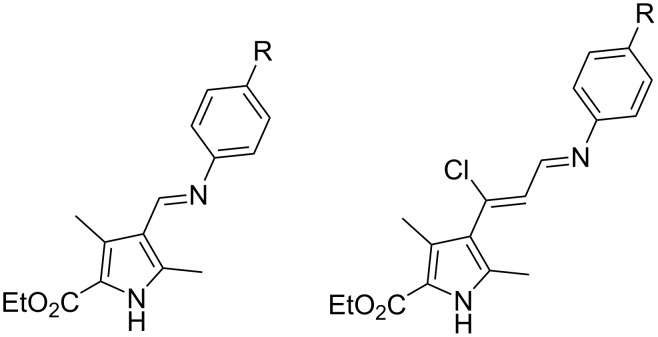
Azomethines based on ethyl 4-acetyl-3,5-dimethyl-1*H*-pyrrole-2-carboxylate and 4-[(*E*)-1-chloro-3-oxoprop-1-enyl]-3,5-dimethyl-1*H*-pyrrole-2-carboxylate.

As our previous studies have shown, azomethines based on aromatic amines and substituted pyrrolecarbaldehyde [40] or pyrrolyl-β-chlorovinylaldehyde [39], contain several frequently encountered motifs in drugs and drug candidates – a pyrrole heterocycle and an azomethine C=N fragment (Figure 1) – and exhibit some biological activity. Thus, Ugi bisamides based on the same aldehydes and amines may also demonstrate biological activity.

In view of these facts, we decided to develop a new approach for the synthesis of hybrid molecules containing substituted heterocyclic and peptidomimetic moieties. The first stage of this approach was the preparation of Ugi bisamides based on pyrrole-containing β-chlorovinylaldehyde and convertible isocyanides. The subsequent post-transformation of the products by acidic hydrolysis conditions should then lead to an acidic component. However, due to the cascade nature of the multicomponent processes and the presence of several alternative reaction centers in the structure of our substances, we sometimes encountered unexpected and intriguing results.

## Results and Discussion

### Synthesis of Ugi bisamides

#### Four-component and three-component Ugi reactions

The combination of pyrrole-containing α,β-unsaturated aldehyde **1**, which contains a chlorine atom in the β-position, with convertible isocyanides **4a**–**d**, *para*-substituted anilines **2a**–**e** and monochloroacetic acid (**3**) as the smallest building blocks in a four-component reaction leads to the formation of the Ugi bisamides **5**–**8** (Table 1). Their structures offer several possibilities for subsequent post-transformation reactions.

**Table 1:** Library of Ugi bisamides **5**–**8** containing a β-chlorovinyl fragment.

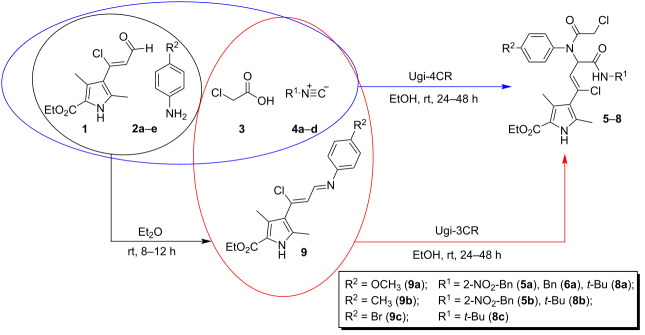				

Bisamide	R^1^	R^2^	Yield 4CR^a^	Yield 3CR^a^

**5a**	2-NO_2_-Bn	OMe	55	47
**5b**		Me	64	47
**5c**		Br	63	–
**5d**		CF_3_	54	–
**5e**		Cl	50	–
**6a**	Bn	OMe	54^b^	26^b^
**6b**		Me	69	–
**6c**		Br	72	–
**6d**		CF_3_	49^b^	–
**6e**		Cl	93	–
**7a**	Cy	OMe	59	–
**7b**		Me	87	–
**7c**		Br	64	–
**7d**		CF_3_	79	–
**7c**		Cl	72	–
**8a**	*t*-Bu	OMe	65	27
**8b**		Me	69	66
**8c**		Br	79	53
**8d**		CF_3_	64	–
**8e**		Cl	58	–

^a^Isolated, EtOH as a solvent; ^b^the first precipitated compound was amide **10b**.

The synthesis of the target Ugi bisamides **5**–**8** was carried out at room temperature in ethanol with stirring for 24–48 hours (depending on the type of starting material) with a yield of 54–93% (Table 1).

It is worth noting that the Ugi-4CR reaction also led to the formation of bisamides **5**–**8** when other solvents were used, e.g., methanol or acetonitrile. However, the yields of the targeted reaction products in methanol were generally lower than in ethanol, while the procedure in acetonitrile was not suitable for all reagents.

It is known that the Ugi-4CR proceeds through the formation of an azomethine (Schiff base) in the first stage [39–40]. Therefore, considering the results of our previous work [39] on the nature and properties of azomethines based on β-chlorovinylaldehyde **1** (Figure 1), we decided to study the possibility of using the three-component Ugi reaction (Ugi-3CR) with preliminary synthesis of azomethines **9a**–**c** (Table 1). In this case, the Ugi bisamides **5**, **6**, **8** were formed, however, it was found that the application of the Ugi-3CR approach had no significant effect on the yields of the target products. Considering the additional reaction step for the synthesis and purification of the starting azomethines **9**, we cannot propose the Ugi-3CR approach as more suitable compared to the Ugi-4CR approach.

**Figure 2:**
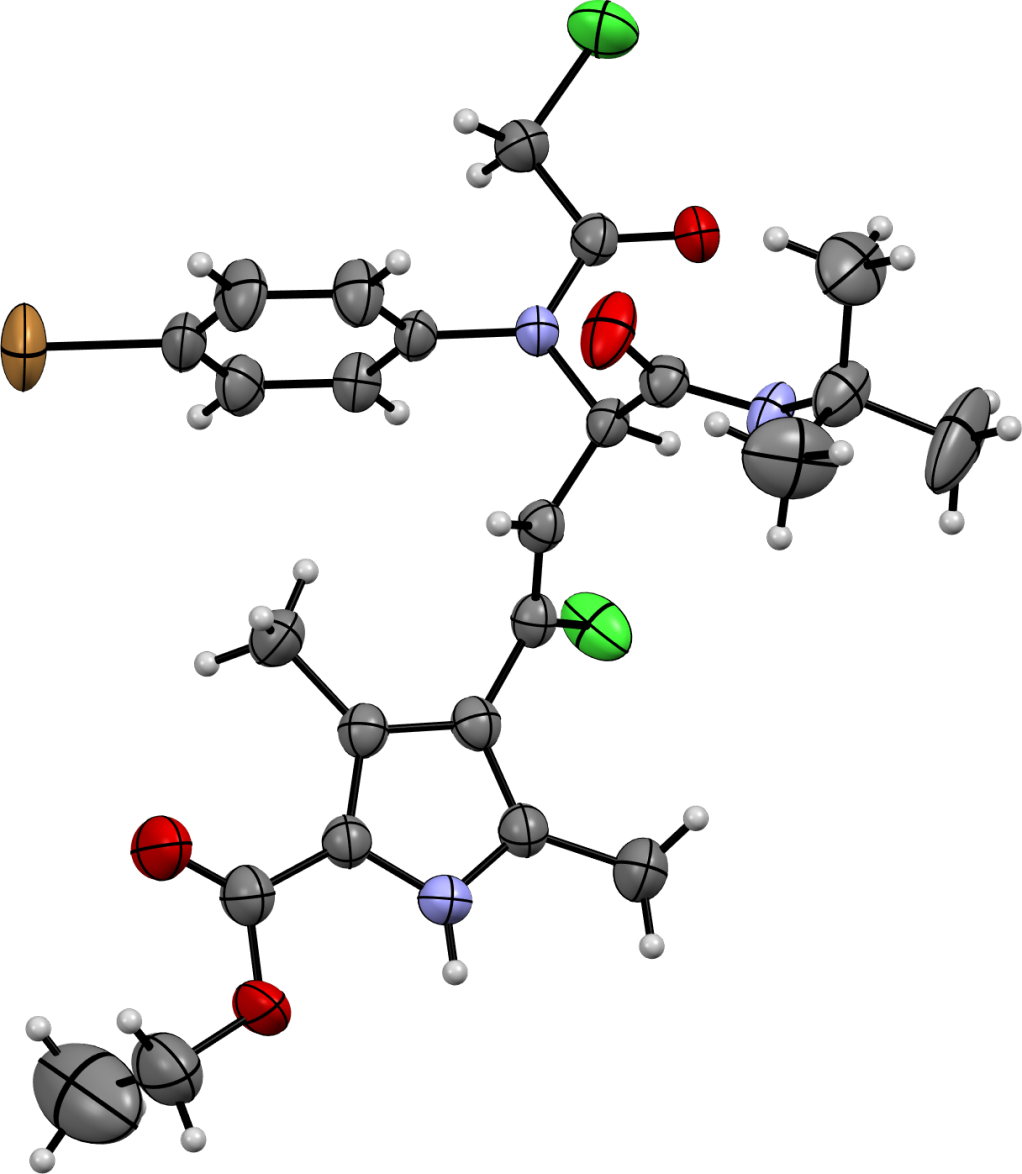
Molecular structure of ethyl (*Z*)-4-(3-(*N*-(4-bromophenyl)-2-chloroacetamido)-4-(*tert*-butylamino)-1-chloro-4-oxobut-1-en-1-yl)-3,5-dimethyl-1*H*-pyrrole-2-carboxylate (**8c**) according to the X-ray diffraction study. Non-hydrogen atoms are presented as thermal ellipsoids with 50% probability.

The structure of the Ugi bisamides **5**–**8** were proved by X-ray diffraction study on the example of substance **8c** (Figure 2), according to which the *Z*-configuration of the chlorovinyl fragment was detected.

### Post-Ugi transformations

**Scheme 3:**
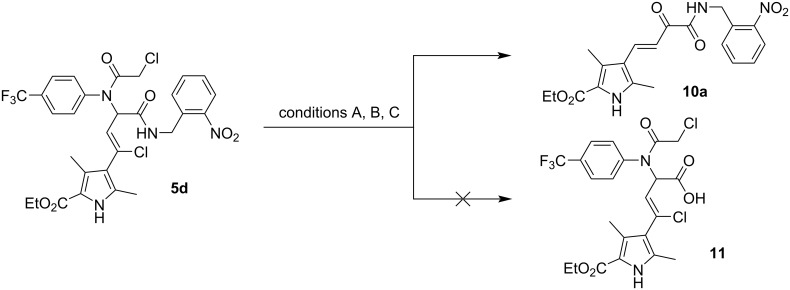
Hydrolysis of Ugi bisamide **5d** in the presence of HCl. Conditions: (A) 5 equiv HCl, MeOH, 80 °C, 3 h; (B) 5 equiv HCl, EtOH, MW 120 °C, 15 min; (C) 5 equiv HCl, MeCN, MW 100 °C, 20 min.

As previously mentioned [32], the introduction of the convertible 2‑bromo-6-isocyanopyridine into the Ugi bisamide structure allows the conversion of the newly formed amide into a carboxylic acid fragment after acid hydrolysis. Similar results were obtained by Dömling and co-workers [27], who used 2-nitrobenzyl isocyanide as a universal convertible isocyanide, and the amide group was also converted into a carboxylic acid under the conditions of acid hydrolysis (Scheme 2). Therefore, taking into account the experience of the authors [27,32], we tried to apply the described hydrolysis conditions (5 equiv HCl in MeOH) to our products (compounds **5**). As a model reaction, we heated a mixture of Ugi bisamide **5d** and an aqueous solution of HCl in methanol (glycerol bath, 80 °C) in a hermetically sealed vial with stirring for three hours (Scheme 3, conditions A).

However, the results of this attempted post-Ugi transformation were quite unexpected: Instead of acid **11**, we isolated the amide of the unsaturated derivative of pyruvic acid **10a** according to the ^1^H and ^13^C NMR spectra and mass spectrometry data. In order to drive the process towards the desired hydrolysis of the secondary amide group, we performed the post-Ugi transformation under MW activation in ethanol or acetonitrile (Scheme 3, conditions B or C). However, the application of MW irradiation did not change the course of the reaction, and as under conventional thermal heating, amide **10a** was isolated, albeit in a lower yield and accompanied with tar formation.

**Figure 3:**
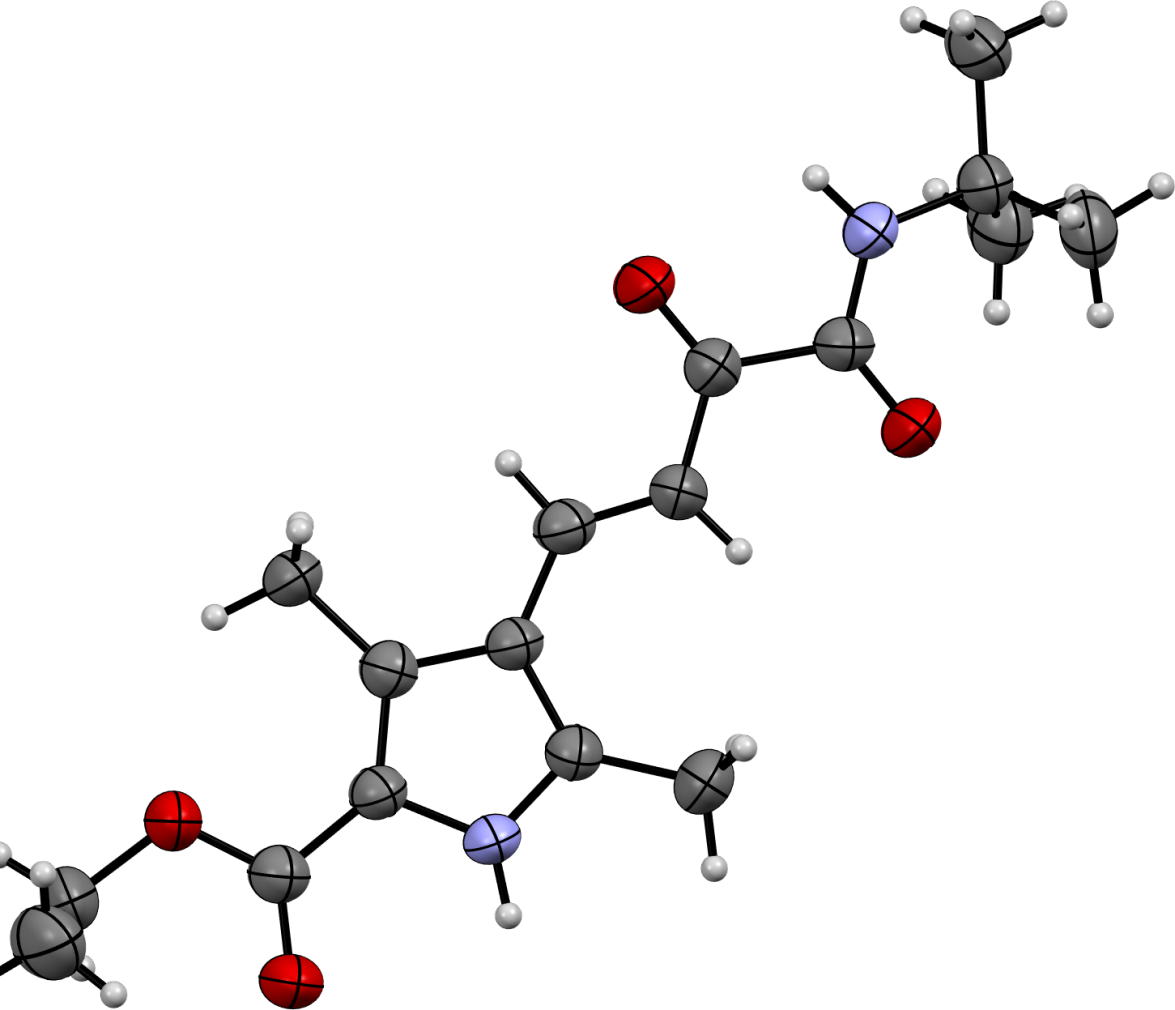
Molecular structure of ethyl (*E*)-4-(4-(*tert*-butylamino)-3,4-dioxobut-1-en-1-yl)-3,5-dimethyl-1*H*-pyrrole-2-carboxylate (**10d**) according to X-ray diffraction data. Non-hydrogen atoms are presented as thermal ellipsoids with 50% probability.

In addition to the ^1^H, ^13^C NMR spectra and mass spectrometry data, the structure of compound **10d** was established by X-ray diffraction analysis (Figure 3). It was also found that the substituents at the double bond are *trans* configured.

To find out the patterns of the new reaction, we applied different conditions to a wider range of starting bisamides **5**–**8** (Table 2). As in the model reaction (Scheme 3), the main products of the transformation were the corresponding amides **10a**–**d** (Table 2).

**Table 2:** Post-Ugi transformations of bisamides **5–8** under different conditions.

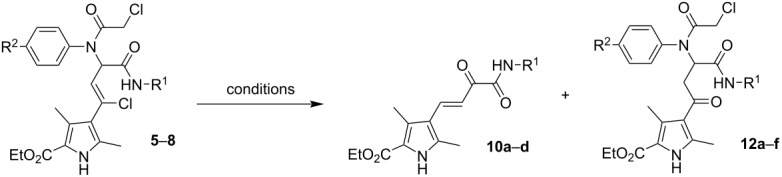									

Entry	Bisamide			Conditions				Yield^a^	
	R^1^	R^2^	No.	solvent	cat	temperature^b^, °C	time, h	amide **10**	ketobisamide **12**

1	2-NO_2_-Bn	Br	**5c**	MeOH	HCl 5 equiv^c^	70	3	**10a**, 13	–
2		CF_3_	**5d**	MeOH	HCl 5 equiv^c^	80	3	**10a**, 42	–
3		CF_3_	**5d**	EtOH	–	80	3.5	**10a**, 44	**12a**, 19
4		CF_3_	**5d**	EtOH	HCl 1 equiv^c^	80	3.5	**10a**, 47	**12a**, 20
5		CF_3_	**5d**	MeOH	HCl 5 equiv^c^	85, MW	0.5	**10a**, 40	–
6		CF_3_	**5d**	EtOH	HCl 5 equiv^c^	100, MW	0.5	**10a**, 39	–
7		CF_3_	**5d**	EtOH	HCl 5 equiv^c^	120, MW	0.25	**10a**, 35	–
8		CH_3_	**5b**	MeCN	HCl 5 equiv^c^	50	6	**10a**, 35	–
9		CF_3_	**5d**	MeCN	HCl 5 equiv^c^	100, MW	0.5	**10a**, 26	–
10	Bn	Br	**6c**	EtOH	–	80	3	**10b**,42	–
11		Me	**6b**	EtOH	–	80	3	**10b**, 35	–
12		Cl	**6e**	EtOH	HCl 1 equiv^c^	50	168	**10b**, 20	–
13		Cl	**6e**	EtOH	HCl 1 equiv^c^	80	3	**10b**, 40	–
14		Br	**6c**	EtOH	HCl 1 equiv^c^	80	2.5	**10b**, 25	–
15		OMe	**6a**	EtOH	HCl 1 equiv^c^	80	3	**10b**, 39	**12b**, traces
16		Me	**6b**	EtOH	HCl 1 equiv^c^	80	3	**10b**, 38	–
17		Br	**6c**	MeCN	HCl 1 equiv^c^	25	216	**10b**, 20	**12c**, 9
18	Cy	Me	**7b**	EtOH	–	25	850	**10c**, 18	**12d**, 71
19		Me	**7b**	EtOH	HCl 5 equiv^c^	80	3	**10c**, 34	–
20		Br	**7c**	EtOH	HCl 5 equiv^c^	80	4.5	**10c**, 40	–
21		OMe	**7a**	EtOH	HCl 5 equiv^c^	80	3	**10c**, 38	–
23	*t*-Bu	Br	**8c**	EtOH	–	25	840	**10d**, 6	**12e**, 6
24		Br	**8c**	EtOH	–	80	3	**10d**, 56	**12e**, 15
25		Br	**8c**	EtOH	HCl 1 equiv^c^	25	850	**10d**, 14	**12e**, 5
26		Br	**8c**	EtOH	DIPEA 2 equiv	80	3	–	–
27		Br	**8c**	EtOH	Et_3_N 2 equiv	80	3	–	–
28		Br	**8c**	EtOH	MCA 1 equiv	80	2	**10d**, 52	**12e**, 13
29		Br	**8c**	EtOH	HCl 0.5 equiv^c^	80	3	**10d**, 64	**12e**, 12
30		Br	**8c**	EtOH	HCl 1 equiv^c^	80	3	**10d**, 46	**12e**, 12
31		Br	**8c**	EtOH	HCl 2 equiv^c^	80	3	**10d**, 47	**12e**, 12
32		Br	**8c**	EtOH	HCl 5 equiv^c^	80	3	**10d**, 51	**12e**, 12
33		OMe	**8a**	EtOH	HCl 5 equiv^c^	80	3	**10d**, 62	**12f**, 21
34		Br	**8c**	MeCN	HCl 1 equiv^c^	25	72	**10d**, 34	**12e**, 46
35		Br	**8c**	MeCN	–	80	3	**10d**, 10	**12e**, 18

^a^Isolated yield; ^b^the temperature in bath; ^c^water 36% solution.

**Figure 4:**
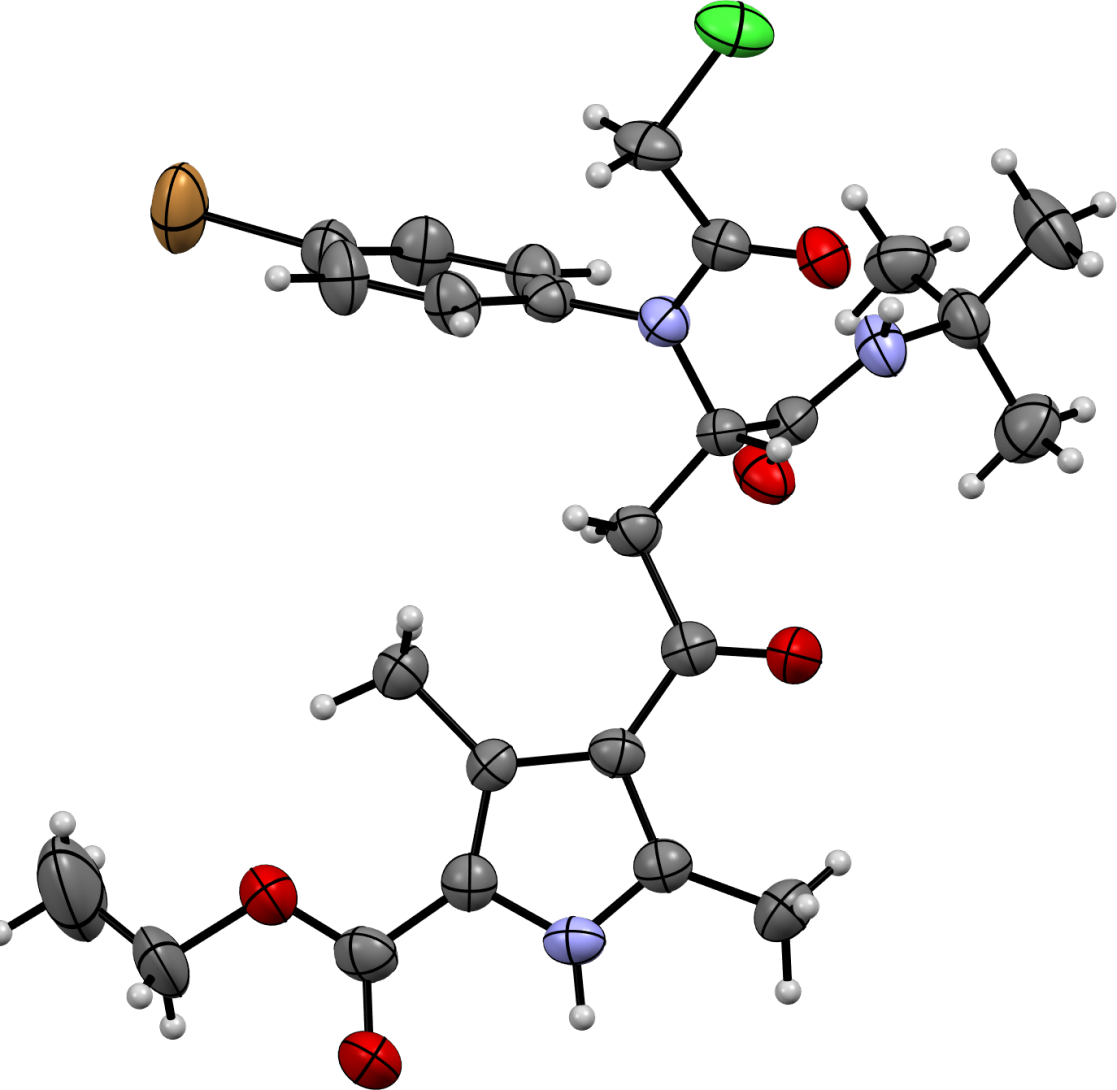
Molecular structure of ethyl 4-(3-(*N*-(4-bromophenyl)-2-chloroacetamido)-4-(*tert*-butylamino)-4-oxobutanoyl)-3,5-dimethyl-1*H*-pyrrole-2-carboxylate (**12e**) according to the X-ray diffraction data. Non-hydrogen atoms are presented as thermal ellipsoids with 50% probability.

In the case of bisamides **5d**, **6a**, **6c**, **7b**, **8a**, and **8c** (Table 2), additional transformation products were also isolated from the reaction mixture. According to ^1^H and ^13^C NMR, MS, and X-ray diffraction studies these were the corresponding ketobisamides **12**, which are products of a nucleophilic substitution of the chlorine atom in the chlorovinyl fragment to the hydroxy group, probably under the influence of water (Figure 4).

**Scheme 4:**
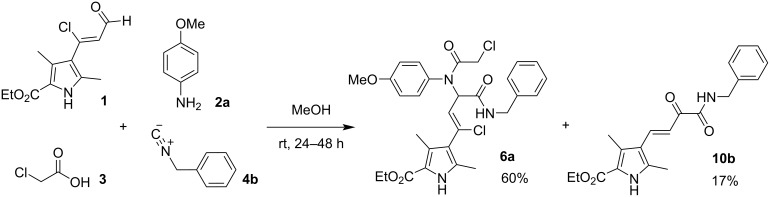
The Ugi-4CR with the participation of *p*-anisidine and benzyl isocyanide.

It is worth noting that the synthesis of Ugi bisamides **5**–**8** (Table 1) yielded compounds **10** and ketobisamide **12** in some cases. For example, in the Ugi reaction involving benzyl isocyanide (**4b**) and *p*-anisidine (**2a**), the product that precipitated first from the reaction mixture was 2-oxo-4-(1*H*-pyrrol-3-yl)but-3-enoic acid amide **10b** (Scheme 4; Table 1, footnote b). Pure Ugi bisamide **6a** could only be obtained when the reaction was carried out in MeCN or EtOH. The same was observed in the case of *p*-CF_3_-substituted aniline when synthesizing bisamide **6d**. The yield of the amide **10c** was about 8% and it was also the first to precipitate.

**Scheme 5:**
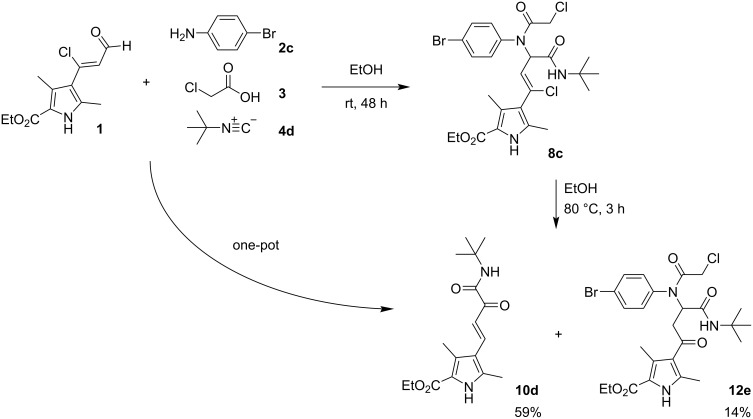
Successful attempt at tandem one-pot coupling of the Ugi-4CR reaction and post-transformation of the corresponding bisamide **8c**.

In addition, it is worth mentioning that the corresponding amides **10** were observed in ^1^H NMR spectra and LC–MS analysis in many mother liquors after filtration of the bisamides **6**–**8**. Moreover, traces of the ketobisamides **12** were also observed when the synthesis was carried out in 96% EtOH. The one-pot synthesis allows to combine two consecutive steps, the Ugi-4CR and the post-Ugi reaction, by keeping the mixture of starting materials **1**, **2c**, **3** and **4d** at 25 °C for two days and then heating the reaction mixture in a closed vessel at 80 °C for 3 hours (Scheme 5). This procedure led to the formation and isolation of the products **10d** and **12e** with yields which are similar to the tandem reaction.

Using the example of bisamide **8c**, it was found that changing the amount of HCl or replacing it with chloroacetic acid under otherwise identical conditions had no significant effect on the yields of products **10** and **12** (Table 2, entries 28–32). At the same time, decreasing the temperature of the post-Ugi transformation of bisamide **8c** in the presence of HCl to 25 °C significantly slowed down the reaction (Table 2, entry 25) and after 36 days a large amount of the starting bisamide remained unchanged in the reaction mixture, while the target amide **10d** and ketobisamide **12e** were observed in low yield. However, when the same reaction was carried out in MeCN, the bisamide **8c** completely disappeared within 3 days (Table 2, entry 34), and the amide **10d** and the ketobisamide **12e** were isolated from the reaction mixture in sufficiently higher yields than when the reaction was carried out in ethanol.

Moreover, the post-Ugi transformation of bisamides **6b**, **6c**, **7b**, and **8c** without addition of acid also led to the formation of amides **10b**–**d** as main products and the corresponding ketobisamides **12d**–**e** as minor products (Table 2, entries 10, 11, 18, 23, and 24). This can be explained by the formation of HCl in the reaction mixture due to the substitution of chlorine in the vinyl chloride moiety under the influence of water. The appearance of HCl in these cases was identified by the specific odor and detected by pH measurements. It is likely that this catalytic amount of HCl is enough for the conversion and formation of the amides **10**.

To confirm the influence of HCl and its necessity to initiate the formation of amide **10**, the post-Ugi transformation of bisamide **8c** was carried out in the presence of Et_3_N or DIPEA (Table 2, entries 26 and 27). The expected corresponding quaternary ammonium salts were isolated, and no trace of amide **10d** was observed.

**Scheme 6:**
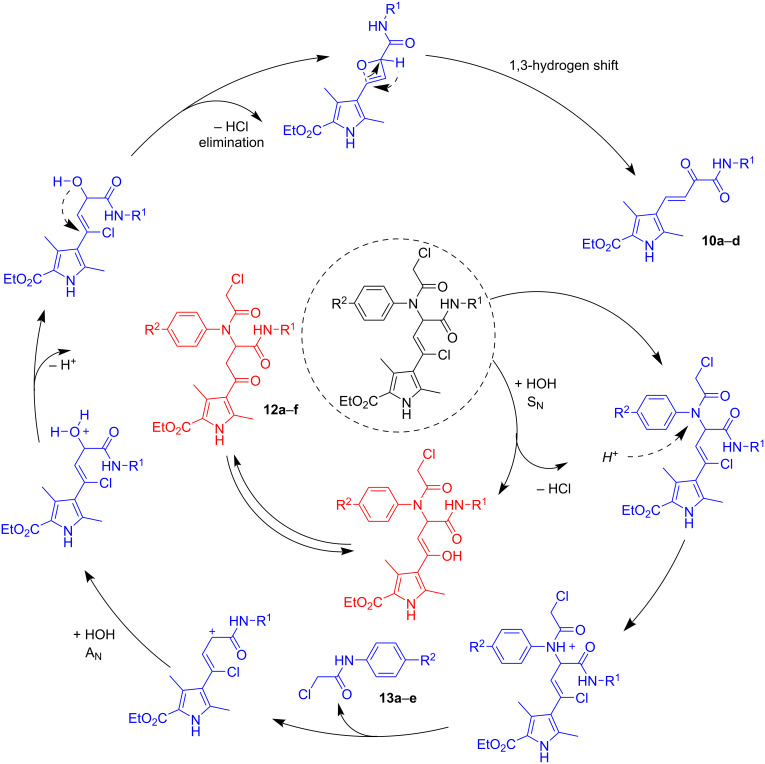
Plausible transformation sequence of the formation of amides **10** and ketobisamides **12**.

It should also be noted that in the ^1^H NMR spectra of the reaction mixtures and the various mother liquors, the signals of the 2-chloroacetamides **13a**–**d** (Scheme 6) [41] were clearly recognizable in many cases and were sometimes isolated individually. In addition, the hydrochlorides of the corresponding *para*-substituted anilines were identified. The amount of salt formed increased with increasing HCl excess, indicating that they were formed by the acidic hydrolysis of the corresponding 2-chloroacetamides **13a**–**e**. The amount of either 2-chloroacetamide or 2-chloroacetamide and ammonium salt together correlated well with the amount of the corresponding amide **10**.

The expected fact that the amides **10** could not be formed from the ketobisamides **12** (Table 3) was also confirmed; stirring the latter in MeCN or EtOH in the presence of HCl at room temperature or under heating did not lead to formation, not even to the appearance of traces of the amide **10d**.

**Table 3:** Hydrolysis of ketobisamide **12e**.

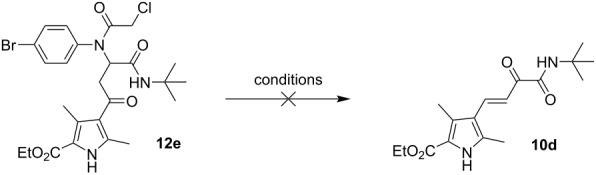						

Entry	Conditions				residue of ketobisamide, **12e**, %	amide **10d**

	solvent	cat	temperature in bath, °C	time, h		

1	MeCN	HCl 1 equiv	25	168	95	not observed
2	MeCN	HCl 1 equiv	80	3	68	not observed
3	EtOH	HCl 2 equiv	80	3	70	not observed

Based on the above facts, we have proposed a plausible transformation sequence for the formation of amides **10** and ketobisamides **12** (Scheme 6).

## Conclusion

Thus, in this work, the multicomponent reaction of pyrrole-containing β-chlorovinylaldehyde, *para*-substituted anilines, monochloroacetic acid, and different convertible isocyanides gives rise to products of the classic Ugi reaction, ethyl (*E*)-4-(4-(R^1^-amino)-1-chloro-3-(2-chloro-*N*-(4-(R^2^)phenyl)acetamido)-4-oxobut-1-en-1-yl)-3,5-dimethyl-1*H*-pyrrole-2-carboxylates, which, however, exhibit unusual behavior in *post*-Ugi transformations. The attempt to apply a well-documented approach for the subsequent synthesis of peptide-containing carboxylic acids by acid hydrolysis of the convertible isocyanide moiety in the Ugi bisamides proceeded in an unexpected manner: their treatment with acids led to elimination of the 2-chloroacetamide moiety and conversion of the β-chlorovinyl fragment into a vinyl fragment giving rise to ethyl (*E*)-4-(4-(R^1^-amino)-3,4-dioxobut-1-en-1-yl)-3,5-dimethyl-1*H*-pyrrole-2-carboxylates. Another direction of the post-transformation was the replacement of the chlorine atom in the β-chlorovinyl group of the Ugi bisamides with a hydroxy group and the formation of a different type of peptidomimetic, namely ethyl 4-(3-(*N*-(4-R^2^-phenyl)-2-chloroacetamido)-4-(R^1^-amino))-4-oxobutanoyl)-3,5-dimethyl-1*H*-pyrrole-2-carboxylates. It was also found that these two unusual products of acidic transformation were observed as byproducts of the Ugi reaction. Optimized synthetic protocols were developed for all reactions and a plausible sequence of the post-Ugi transformation was provided.

## Supporting Information

File 1 Experimental section, NMR and LC–MS spectra as well as X-ray data.

## Data Availability

The data that supports the findings of this study is available from the corresponding author upon reasonable request.
