# Supplementary material for: Ugi bisamides based on pyrrolyl-β-chlorovinylaldehyde and their unusual transformations
Source: Beilstein J Org Chem. 2024 Jul 26;20:1773–84. doi: 10.3762/bjoc.20.156 (PMC11285049; doi:10.3762/bjoc.20.156)
Supplement: File 1 — Experimental section, NMR and LC–MS spectra as well as X-ray data. [file Beilstein_J_Org_Chem-20-1773-s001.pdf]

BEILSTEIN JOURNAL OF ORGANIC CHEMISTRY

Supporting Information

for

Ugi bisamides based on pyrrolyl-β-chlorovinylaldehyde and their unusual transformations

Alexander V. Tsygankov, Vladyslav O. Vereshchak, Tetiana O. Savluk, Serhiy M. Desenko, Valeriia V. Ananieva, Oleksandr V. Buravov, Yana I. Sakhno, Svitlana V. Shishkina and Valentyn A. Chebanov

Beilstein J. Org. Chem. **2024,** *20,* 1773–1784. doi:10.3762/bjoc.20.156

Experimental section, NMR and LC–MS spectra as well as X-ray data

License and Terms: This is a supporting information file under the terms of the Creative Commons Attribution License (https://creativecommons.org/licenses/by/4.0). Please note that the reuse, redistribution and reproduction in particular requires that the author(s) and source are credited and that individual graphics may be subject to special legal provisions.
The license is subject to the *Beilstein Journal of Organic Chemistry* terms and conditions: (https://www.beilstein-journals.org/bjoc/terms)

Table of contents

General S2

Synthetic aspects and procedures S4

^{1}H NMR and ^{13}C NMR spectra of compounds **5–8**, **10a–d**, and **12a**,**c–f** S22

LC–MS spectra of compounds **5–8**, **10a–d**, and **12a–f** S79

X-ray diffraction study of compounds **8c**, **10d**, and **12e** S109

References S112

General

The starting ethyl (*E*)-4-(1-chloro-3-oxoprop-1-en-1-yl)-3,5-dimethyl-1*H*-pyrrole-2-carboxylate (**1**) and azomethines **9** derived from **1** were synthesized according to the known literature procedure [40]. *p*-Substituted anilines **2a**–**e**, 2-chloroacetic acid (**3**), 2-nitrobenzyl-, benzyl-, cyclohexyl-, and *tert*-butyl isocyanides **4a**–**d** are commercially available.

Microwave experiments were performed using a monomode microwave reactor Monowave 300 from Anton Paar (operating frequency 2.45 GHz). The experiments were carried out in sealed borosilicate vials. The reaction time was determined by the duration of irradiation of the reaction mixture at a given temperature and did not include the heating and cooling period.

The course of the reactions and the purity of the obtained compounds were controlled by TLC on ALUGRAM Xtra SIL G UV254 plates eluting with EtOAc, hexane, $CHCl_3$ and their mixtures, visualization under UV light and in iodine chamber.

Melting points of all synthesized compounds were determined with a Stuart SMP10 melting point electronic device and were uncorrected. The NMR spectra were recorded in DMSO-d_6 and $CDCl_3$ at 400 MHz (100 MHz for ^{13}C) with a Varian MR-400 spectrometer, in all cases δ-scale of the chemical shifts was used. Mass spectra (ESI) were recorded in both positive and negative ion detection modes on a Shimadzu LCMS-2020 spectrometer. Elemental analysis was realized on EA-3000 CHNS-O analyzer (Eurovector, Italy).

X-ray diffraction studies were performed on an automatic “Bruker APEX II” diffractometer (graphite monochromated MoKα radiation, CCD-detector, φ- and ω-scanning). The structures were solved by direct method using SHELXTL package [1]. Positions of the hydrogen atoms were located from electron density difference maps and refined by “riding” model with U_{iso} = nU_{eq} of the carrier atom (n = 1.5 for methyl and hydroxy groups and n = 1.2 for other hydrogen atoms). The crystallographic data and experimental parameters are listed in Table S1. Final atomic coordinates, geometrical parameters and crystallographic data have been deposited with the Cambridge Crystallographic Data Centre, 11 Union Road, Cambridge, CB2 1EZ, UK (fax: +44 1223 336033; e-mail: deposit@ccdc.cam.ac.uk). The deposition numbers are given in Table S1.

Table S1. The crystallographic data and experimental parameters for compounds **8c, 10d, 12e**.

Parameter	**8c**	**10d**	**12e**
Unit cell<br>a, Å	18.4279(16)	7.9849(9)	18.5878(16)
b, Å	10.4917(8)	10.3167(13)	22.0376(16)
c, Å	33.008(2)	11.5927(14)	14.5817(13)
α, deg	90.0	105.842(8)	90.0
β, deg	103.183(9)	102.053(8)	116.252(8)
γ, deg	90.0	99.619(9)	90.0
V, $Å^3$	6213.7(9)	872.59(19)	5357.0(8)
F(000)	2624	344	2352
Crystal system	Monoclinic	Triclinic	Monoclinic
Space group	*C*2/c	$P\bar{1}$	*C*2/c
Z	8	2	8
μ, mm^{-1}	1.533	0.087	1.673
D_{calc}, g/cm^3	1.354	1.219	1.411
$2\Theta_{max}$, grad	50.0	50.0	50.0
Measured reflections	42628	12277	36858
Independent reflections	5481	3073	4734
R_{int}	0.0435	0.0723	0.0783
Reflections with F>4σ(F)	3638	1750	2567
Parameters	361	214	322
R_1	0.0571	0.1042	0.0573
wR_2	0.1502	0.3417	0.1529
S	1.021	1.118	1.004
CCDC number	2350875	2350876	2350874

Synthetic aspects and procedures

Synthesis Ugi bisamides 5–8

Four-component procedure

(*E*)-4-(1-Chloro-3-oxoprop-1-en-1-yl)-3,5-dimethyl-1*H*-pyrrole-2-carboxylate (**1**, 1.6 mmol) and *p*-substituted aniline **2a–e** (1.6 mmol) were added to a 10 mL screw cap vial and dissolved in 2 mL of solvent (EtOH, MeOH or MeCN). Then, 2-chloroacetic acid (**3**) and isocyanide **4a–d** were added to the resulting solution. The mixture is diluted with 2 mL of the corresponding solvent, the vial was hermetically closed, and the mixture left to stir for 24–48 hours at a temperature of 25 °C. After that, the formed precipitate was collected by filtration and washed with cold solvent, then dried in a vacuum drying oven at a temperature of 30 °C. In some cases, the solid products were not pure or the products which did not precipitate out. In these cases, the solvents were evaporated under vacuum, the residue dissolved in DMF, and poured onto ice. The precipitate was filtered and washed with aqueous ethanol. The crude product was purified by column chromatography, eluent $CHCl_3$/EtOAc.

Three-component procedure

The corresponding azomethine **9a**–**c** (1.6 mmol) were added to a 10 mL screw cap vial and dissolved in 2 mL of EtOH. Then, 2-chloroacetic acid (**3**) and isocyanide (**4a**,**b**,**d**) were added to the reaction mixture. The mixture was diluted with 2 mL of EtOH, the vial was hermetically closed and the mixture left to stir for 24–48 hours at a temperature of 25 °C. After that, the formed precipitate was collected by filtration, washed with cold EtOH and dried in a vacuum drying oven at a temperature of 30 °C.

In these ways, a library of 20 bisamides was obtained:

Ethyl (*Z*)-4-(1-chloro-3-(2-chloro-*N*-(4-methoxyphenyl)acetamido)-4-((2-nitrobenzyl)amino)-4-oxobut-1-en-1-yl)-3,5-dimethyl-1*H*-pyrrole-2-carboxylate (5a). Yield 55%, dirty yellowish solid, mp = 177-178 °C (with decomp.).
^{1}H NMR spectrum (DMSO-d_6), δ, ppm (*J*, Hz): 1.25 (t, 3H, -CH$_2$<u>CH$_3$</u>, J^3 7.1), 1.96 (s, 3H, 5-CH$_3$), 2.05 (s, 3H, 3-CH$_3$), 3.76 (s, 3H, -OCH$_3$), 3.93 (d, 1H, CH$_2$Cl, J^2 13.7), 4.08 (d, 1H, CH$_2$Cl, J^2 13.7), 4.19 (qd, 2H, -<u>CH$_2$</u>CH$_3$, *J* 7.1), 4.64 m (2H, <u>CH$_2$</u>C$_6$H$_4$), 5.32 (d, =CH-, J^3 8.8), 5.75 (d, 1H, CH, *J* 8.8), 6.96-8.05 (m, 8H, Ar), 8.83 (br.s, 1H, <u>NH</u>C(O), J^3 5.6), 11.48 (s, 1H, <u>NHPyrrole</u>).

^{13}C NMR spectrum (DMSO-d_6), δ, ppm: 10.75, 11.11, 14.39, 39.13-40.13 (1C, CH_2Ph), 42.62, 55.36, 59.19, 60.35, 114.45, 116.72, 120.05, 123.66, 124.52, 125.65, 128.24, 129.22 (2C, Ar), 130.53 (2C, Ar), 131.23, 132.33, 132.41, 133.67, 133.95, 147.78, 159.24, 160.52, 166.01, 169.22.

Mass spectrum (ESI), m/z$^+$: 617 (68%), 618 (25%), 619 (53%), 620 (18%), 621 (12%) $[M+H]^+$, 634 (22%), 636 (16%), 639 (100%), 640 (36%), 641 (68%), 642 (24%), 643 (16%), 644 (18%) $[M+Na]^+$, 655 (26%), 656 (11%), 657 (21%) $[M+K]^+$, 680 (60%), 681 (23%), 682 (43%), 683 (16%) $[M+ACN+Na]^+$.

Anal. Calcd (%) for $C_{29}H_{30}Cl_2N_4O_7$: C, 56.41; H, 4.90; N, 9,07. Found: C, 56.35; H, 4.95; N, 9.12.

Ethyl (*Z*)-4-(1-chloro-3-(2-chloro-*N*-(*p*-tolyl)acetamido)-4-((2-nitrobenzyl)amino)-4-oxobut-1-en-1-yl)-3,5-dimethyl-1*H*-pyrrole-2-carboxylate (5b). Yield 64%, yellowish solid, mp = 179-180 °C (with decomp.).

^1H NMR spectrum (DMSO-d_6), δ, ppm (*J*, Hz): 1.25 (t, 3H, -$CH_2\underline{CH_3}$, J^3 7.1), 1.94 (s, 3H, 5-CH_3), 2.04 (s, 3H, 3-CH_3), 2.31 (s, 3H, $C_6H_4\underline{CH_3}$), 3.92 (d, 1H, CH_2Cl, J^2 13.7), 4.08 (d, 1H, CH_2Cl, J^2 13.7), 4.19 (q, 2H, -$\underline{CH_2}CH_3$, J^3 7.1), 4.59-4.70 (m, 2H, $\underline{CH_2}C_6H_4$), 5.33 (d, =CH-, J^3 8.7), 5.75 (d, 1H, CH, J^3 8.8), 7.22-8.05 (m, 8H, Ar), 8.82 (br.t, 1H, <u>NH</u>C(O)), 11.47 (s, 1H, <u>NHPyrrole</u>).

^{13}C NMR spectrum (DMSO-d_6), δ, ppm: 11.14, 11.51, 14.85 21.06, 39.34-40.61 (1C, $\underline{C}H_2Ph$), 42.99, 59.65, 60.82, 117.21, 120.53, 124.13, 124.97 (2C, Ar), 126.14, 128.71, 129.53, 129.75, 130.34 (2C, Ar), 132.80, 132.94, 134.12, 134.41, 136.66, 138.96, 148.29, 161.00, 166.27, 169.61.

Mass spectrum (ESI), m/z$^+$: 601 (100%), 603 (70%), 602 (31%), 604 (22%) $[M+H]^+$, 618 (35%), 620 (26%), 619 (15%) $[M+NH_4]^+$, 623 (17%), 625 (11%), 624 (5%) $[M+Na]^+$, 639 (9%), 641 (8%), 640 (3%), $[M+K]^+$; m/z$^-$: 599 (100%), 601 (71%), 600 (40%), 602 (23%) $[M-H]^-$, 637 (35%), 635 (31%), 639 (12%), 636 (10%) $[M+Cl]^-$.

Anal. Calcd (%) for $C_{29}H_{30}Cl_2N_4O_6$: C, 57.91; H, 5.03; N, 9.32. Found: C, 58.12; H, 5.09; N, 9.24.

Ethyl (*Z*)-4-(3-(*N*-(4-bromophenyl)-2-chloroacetamido)-1-chloro-4-((2-nitrobenzyl)amino)-4-oxobut-1-en-1-yl)-3,5-dimethyl-1*H*-pyrrole-2-carboxylate (5c). Yield 63%, yellowish solid, mp = 186 °C (with decomp.).

^{1}H NMR spectrum (DMSO-d_6), δ, ppm (*J*, Hz): 1.25 (t, 3H, -CH$_2$<u>CH$_3$</u>), 1.93 (s, 3H, 5-CH$_3$), 2.01 (s, 3H, 3-CH$_3$), 3.98 (d, CH$_2$Cl, J^2 14.3), 4.12-4.24 (m, 3H, CH$_2$Cl, -<u>CH$_2$</u>CH$_3$), 4.57-4.72 (m, 2H, <u>CH$_2$C$_6$H$_4$</u>), 5.34 (d, =CH-, J^3 8.6), 5.76 (d, 1H, CH, J^3 8.6), 7.47-8.06 (m, 8H, Ar), 8.95 (br.s 1H, <u>NH</u>C(O)), 11.51 (s, 1H, <u>NHPyrrole</u>).

^{13}C NMR spectrum (DMSO-d_6), δ, ppm: 10.65, 11.01, 14.38, 38.96-40.06 (1C, <u>C</u>H$_2$Ph) 42.69, 59.20, 60.27, 116.76, 119.89, 122.10, 123.33, 124.53 (2C, Ar), 125.61, 128.26, 129.24, 131.57, 132.35 (2C, Ar), 133.04, 133.68, 133.88, 138.06, 147.77, 158.54, 160.51, 165.58, 169.18.

Mass spectrum (ESI), m/z$^+$: 667 (100%), 669 (58%), 665 (49%), 668 (38%), 666 (17%), 670 (16%) $[M+H]^+$, 684 (90%), 682,1 (51%), 686 (49%), 685 (36%) $[M+NH_4]^+$, 689,0 (48%), 687,0 (39%), 691,0 (22%), 690,0 (13%) $[M+Na]^+$, 705 (52%), 703 (29%), 707 (28%), 706 (18%) $[M+K]^+$, 730 (65%), 728 (44%), 732 (30%), 731 (26%) $[M+ACN+Na]^+$; m/z$^-$: 665 (100%), 663 (50%), 667 (49%) $[M-H]^-$, 701 (30%), 703 (22%), 699 (19%) $[M+Cl]^-$.

Anal. Calcd (%) for $C_{28}H_{27}BrCl_2N_4O_6$: C, 50.47; H, 4.08; N, 8.41. Found: C, 50.30; H, 3.98; N, 8.45.

Ethyl (*Z*)-4-(1-chloro-3-(2-chloro-*N*-(4-(trifluoromethyl)phenyl)acetamido)-4-((2-nitrobenzyl)amino)-4-oxobut-1-en-1-yl)-3,5-dimethyl-1*H*-pyrrole-2-carboxylate (5d). Yield 54%, creamy solid, mp = 188-189 °C (with decomp.).

^{1}H NMR spectrum (DMSO-d_6), δ, ppm (*J*, Hz): 1.24 (t, 3H, -CH$_2$<u>CH$_3$</u>, J^3 7.1), 1.86 (s, 3H, 5-CH$_3$), 1.93 (s, 3H, 3-CH$_3$), 4.00 (d, 1H, CH$_2$Cl, J^2 14.3), 4.16-4.24 (m, 3H, CH$_2$Cl, -<u>CH$_2$</u>CH$_3$), 4.60-4.71 (qd, 2H, <u>CH$_2$</u>C$_6$H$_4$), 5.36 (d, =CH-, J^3 8.4), 5.81 (d, 1H, CH, J^3 8.4), 7.52-8.09 (m, 8H, Ar), 8.99 (br.t, 1H, <u>NH</u>C(O)), 11.49 (s, 1H, <u>NHPyrrole</u>).

^{13}C NMR spectrum (DMSO-d_6), δ, ppm: 10.99, 11.39, 14.83, 39.42-40.64 (1C, <u>C</u>H$_2$Ph), 43.17, 59.67, 60.85, 117.24, 120.34, 122.99-129.42 (1C, CF$_3$), 123.72, 125.01 (2C, Ar), 126.07, 127.03 (3C, Ar), 128.77, 129.78, 130.80, 132.76, 133.83, 134.16, 134.31, 142.98, 148.30, 160.97, 166.02, 169.68.

Mass spectrum (ESI), m/z$^+$: 657 (17%), 655 (12%), 658 (8%), 659 (5%), $[M+H]^+$; 677 (71%), 679 (58%), 678 (20%), 680 (15%) $[M+Na]^+$, 693 (100%), 695 (86%), 694 (31%), 696 (28%), 697 (18%), 698 (5%), 699 (2%) $[M+K]^+$, 718 (85%), 720 (68%), 719 (30%) $[M+CH_3CN+Na]^+$; m/z$^-$: 653 (100%), 655 (74%), 654 (20%), 656 (19%), 657 (11%), 658 (4%) $[M-H]^-$, 691 (100%), 689 (98%), 693 (36%) $[M+Cl]^-$.

Anal. Calcd (%) for $C_{29}H_{27}Cl_2F_3N_4O_6$: C, 53.14; H, 4.15; N, 8.55. Found: C, 52.81; H, 4.27; N, 8.39.

Ethyl (*Z*)-4-(1-chloro-3-(2-chloro-*N*-(4-chlorophenyl)acetamido)-4-((2-nitrobenzyl)amino)-4-oxobut-1-en-1-yl)-3,5-dimethyl-1*H*-pyrrole-2-carboxylate (5e). Yield 50%, creamy solid, mp = 205-207 °C (with decomp.).

^{1}H NMR spectrum (DMSO-d_6), δ, ppm (J, Hz): 1.25 (t, 3H, -CH_2<u>CH_3</u>, J^3 7.2), 1.94 (s, 3H, 5-CH_3), 2.02 (s, 3H, 3-CH_3), 3.98 (d, 1H, CH_2Cl, J^2 14.3), 4.13-4.23 (m, 3H, CH_2Cl, -<u>CH_2</u>CH_3), 4.65 (qd, 2H, <u>CH_2</u>C_6H_4, J^3 5.8), 5.34 (d, =CH-, J^3 8.5), 5.77 (d, 1H, CH, J^3 8.6), 7.42-7.78 (m, 8H, Ar), 8.95 (t, 1H, <u>NH</u>C(O), J^3 6.0), 11.51 (s, 1H, <u>NHPyrrole</u>).

^{13}C NMR spectrum (DMSO-d_6), δ, ppm: 10.66, 11.04, 14.39, 39.02-40.02 (1C, <u>C</u>H_2Ph), 42.65, 59.20, 60.36, 116.81, 119.94, 123.37, 124.51, 125.63, 128.26, 129.32, 129.40 (2C, Ar), 131.28 (2C, Ar), 132.34, 133.04, 133.53, 133.66, 133.87, 137.66, 147.83, 160.53, 165.66, 169.17.

Mass spectrum (ESI), m/z$^+$: 621 (9%), 623 (6%) $[M+H]^+$; 643 (14%), 645 (13%) $[M+Na]^+$, 659 (85%), 660 (21%) $[M+K]^+$, 661 (100%), 663 (41%), 662 (31%), 664 (11%) $[M+CH_3CN+H]^+$, 684 (13%), 686 (14%) $[M+CH_3CN+Na]^+$.

Anal. Calcd (%) for $C_{28}H_{27}Cl_3N_4O_6$: C, 54.08; H, 4.38; N, 9.01. Found: C, 54.22; H, 4.26; N, 8.90.

Ethyl (*Z*)-4-(4-(benzylamino)-1-chloro-3-(2-chloro-*N*-(4-methoxyphenyl)acetamido)-4-oxobut-1-en-1-yl)-3,5-dimethyl-1*H*-pyrrole-2-carboxylate (6a). Yield 54% (61% in MeCN), creamy solid, mp = 144-145 °C.

^{1}H NMR spectrum (DMSO-d_6), δ, ppm (J, Hz): 1.25 (t, 3H, -CH_2<u>CH_3</u>, J^3 7.0), 1.94 (s, 3H, 5-CH_3), 2.04 (s, 3H, 3-CH_3), 3.75 (s, 3H, -OCH_3), 3.92 (d, 1H, -CH_2Cl, J^2 13.7), 4.09 (d, 1H, -CH_2Cl, J^2 13.7), 4.19 (q, 2H, <u>-CH_2</u>CH_3, J^3 7.0), 4.29 (dd, 1H, <u>CH_2</u>C_6H_5, J^2 14.9, J^3 5.9), 4.41 (dd, 1H, <u>CH_2</u>C_6H_5, J^2 15.1, J^3 6.1), 5.26 (d, 1H, =CH-, J^3 8.7), 5.76 (d, 1H, CH, J^3 8.6), 6.93-7.41 (m, 4H, Ar), 7.23-7.33 (m, 5H, CH_2<u>C_6H_5</u>), 8.78 (br.s, 1H, <u>NH</u>C(O)), 11.49 (s, 1H, <u>NHPyrrole</u>).

^{13}C NMR spectrum (DMSO-d_6), δ, ppm: 10.75, 11.10, 14.38, 38.94, 39.11, 39.28, 39.45, 39.61, 39.71, 39.78, 39.87, 39.95, 42.36, 42.67, 55.33, 59.18, 60.11, 114.39 (2C, Ar), 116.71, 120.10, 123.98 (2C, Ar), 125.65, 126.70, 127.08 (2C, Ar), 128.16 (2C, Ar), 130.61, 131.21, 132.16, 132.29, 139.21, 159.20, 160.52, 165.84, 168.71.

Mass spectrum (ESI), m/z$^+$: 572 (100%), 574 (71%), 573 (36%), 575 (22%), 576 (14%) $[M+H]^+$, 594 (26%), 596 (19%), 595 (9%), 597 (6%) $[M+Na]^+$, 610 (13%), 612 (12%), 611 (5%), 613 (4%) $[M+K]^+$, 635 (16%), 637 (12%), 636 (5%), 638 (4%) $[M+CH_3CN+Na]^+$; m/z$^-$ 570 (100%),

572 (61%), 571 (41%), 573 (19%), 574 (11%), 575 (5%) [M-H]$^-$, 606 (77%), 607 (27%) [M+Cl]$^-$, 608 (85%), 610 (30%), 609 (29%), 611 (12%) [M+K-2H]$^-$.

Anal. Calcd (%) for $C_{29}H_{31}Cl_2N_3O_5$: C, 60.84; H, 5.46; N, 7.34. Found: C, 60.57; H, 5.56; N, 7.20.

Ethyl (*Z*)-4-(4-(benzylamino)-1-chloro-3-(2-chloro-*N*-(*p*-tolyl)acetamido)-4-oxobut-1-en-1-yl)-3,5-dimethyl-1*H*-pyrrole-2-carboxylate (6b). Yield 69%, white solid, mp = 173-175 °C.

^{1}H NMR spectrum (DMSO-d_6), δ, ppm (J, Hz): 1.25 (t, 3H, -CH$_2$<u>CH$_3$</u>, J^3 7.1), 1.93 (s, 3H, 5-CH$_3$), 2.02 (s, 3H, 3-CH$_3$), 2.30 (s, 3H, C$_6$H$_4$<u>CH$_3$</u>), 3.91 (d, 1H, -CH$_2$Cl, J^2 13.8), 4.09 (d, 1H, -CH$_2$Cl, J^2 13.8), 4.19 (q, 2H, <u>-CH$_2$</u>CH$_3$, J^3 7.1), 4.30 (dd, 1H, <u>CH$_2$</u>C$_6$H$_5$, J^2 15.1, J^3 5.9), 4.41 (dd, 1H, <u>CH$_2$</u>C$_6$H$_5$, J^2 15.2, J^3 6.1), 5.27 (d, 1H, =CH-, J^3 8.7), 5.76 (d, 1H, CH, J^3 8.6), 7.17-7.31 (m, 9H, Ar), 8.78 (br.s, 1H, <u>NH</u>C(O)), 11.48 (s, 1H, <u>NHPyrrole</u>).

^{13}C NMR spectrum (DMSO-d_6), δ, ppm: 10.69, 11.04, 14.38, 20.59, 38.98, 39.14, 39.31, 39.48, 39.56, 39.65, 39.81, 39.98, 42.38, 42.62, 59.17, 60.08, 116.71, 120.10, 123.96, 125.65, 126.70, 127.08 (2C, Ar), 128.16 (2C, Ar), 129.15 (2C, Ar), 129.81 (2C, Ar), 132.23, 136.15, 138.41, 139.23, 160.52, 165.60, 168.64.

Mass spectrum (ESI), m/z$^+$: 556 (100%), 558 (75%), 557 (33%), 559 (24%), 560 (15%) [M+H]$^+$, 573 (8%), 575 (6%), 574 (3%) [M+NH$_4$]$^+$, 594 (18%), 596 (15%), 595 (6%), 597 (5%) [M+K]$^+$; m/z$^-$ 554 (100%), 556 (60%), 555 (30%), 557 (210%), 558 (14%) [M-H]$^-$, 590 (21%), 591 (9%) [M+Cl]$^-$, 592 (24%), 593 (8%) [M+K-2H]$^-$.

Anal. Calcd (%) for $C_{29}H_{31}Cl_2N_3O_4$: C, 62.95; H, 5.62; N, 7.55. Found: C, 62.68; H, 5.42; N, 7.73.

Ethyl (*Z*)-4-(4-(benzylamino)-3-(*N*-(4-bromophenyl)-2-chloroacetamido)-1-chloro-4-oxobut-1-en-1-yl)-3,5-dimethyl-1*H*-pyrrole-2-carboxylate (6c). Yield 72%, white solid, mp = 193-195 °C.

^{1}H NMR spectrum (DMSO-d_6), δ, ppm (J, Hz): 1.25 (t, 3H, -CH$_2$<u>CH$_3$</u>, J^3 7.1), 1.92 (s, 3H, 5-CH$_3$), 2.00 (s, 3H, 3-CH$_3$), 3.96 (d, 1H, -CH$_2$Cl, J^2 14.0), 4.14 (d, 1H, -CH$_2$Cl, J^2 14.2), 4.19 (q, 2H, <u>-CH$_2$</u>CH$_3$, J^3 7.1), 4.29 (d, 1H, <u>CH$_2$</u>C$_6$H$_5$, J^3 14.3), 4.40 (d, 1H, <u>CH$_2$</u>C$_6$H$_5$, J^3 14.3), 5.29 (d, 1H, =CH-, J^3 8.4), 5.75 (d, 1H, CH, J^3 8.6), 7.29 (m, 5H, CH$_2$<u>C$_6$H$_5$</u>), 7.57 (m, 4H, C$_6$H$_4$), 8.77 (br.s, 1H, <u>NH</u>C(O)), 11.48 (s, 1H, <u>NHPyrrole</u>).

^{13}C NMR spectrum (DMSO-d_6), δ, ppm: 10.66, 11.01, 14.38, 38.97, 39.13, 39.30, 39.47, 39.63, 39.72, 39.80, 39.89, 39.97, 42.38, 42.73, 59.19, 60.09, 116.75, 119.94, 122.06, 123.61, 125.61,

126.73, 127.09 (2C, Ar), 128.18 (3C, Ar), 131.65 (2C, Ar), 132.31 (2C, Ar), 132.82, 138.07, 139.12, 160.51, 165.40, 168.65.

Mass spectrum (ESI), m/z$^+$: 622 (77%), 620 (45%), 624 (39%), 623 (25%), 621 (13%), 625 (11%), 626 (7%) $[M+H]^+$, 644 (54%), 642 (37%), 646 (28%), 645 (18%), 647 (14%), 643 (11%) $[M+Na]^+$, 667 (100%), 665 (61%), 669 (49%), 668 (33%), 666 (20%), 670 (17%) $[M+2Na-H]^+$; m/z$^-$ 620 (44%), 618 (26%), 622 (23%), 621 (15%), 623 (6%), 619 (5%) $[M-H]^-$, 656 (100%), 658 (71%) 654 (51%), 657 (34%), 660 (22%), 659 (21%), 655 (11%) $[M+K-2H]^-$.

Anal. Calcd (%) for $C_{29}H_{28}BrCl_2N_3O_4$: C, 54.13; H, 4.54; N, 6.76. Found: C, 53.90; H, 4.69; N, 6.54.

Ethyl (*Z*)-4-(4-(benzylamino)-1-chloro-3-(2-chloro-*N*-(4-(trifluoromethyl)phenyl)acetamido)-4-oxobut-1-en-1-yl)-3,5-dimethyl-1*H*-pyrrole-2-carboxylate (6d). Yield 49% (63% in MeCN), white solid, mp = 145-147 °C.

^{1}H NMR spectrum (DMSO-d_6), δ, ppm (*J*, Hz): 1.24 (t, 3H, -$CH_2\underline{CH_3}$, J^3 7.0), 1.84 (s, 3H, 5-CH_3), 1.92 (s, 3H, 3-CH_3), 3.99 (d, 1H, -CH_2Cl, J^2 14.7), 4.14-4.25 (m, 3H, -CH_2Cl, -$\underline{CH_2}CH_3$), 4.33 (d, 1H, $\underline{CH_2}C_6H_5$, J^3 14.6), 4.41 (d, 1H, $\underline{CH_2}C_6H_5$, J^3 14.8), 5.31 (d, 1H, =CH-, J^3 8.4), 5.80 (d, 1H, CH, J^3 8.2), 7.27-7.34 (m, 5H, $CH_2\underline{C_6H_5}$), 7.77-7.87 (m, 4H, C_6H_4), 8.92 (br.s, 1H, <u>NH</u>C(O)), 11.49 (s, 1H, <u>NH</u>Pyrrole).

^{13}C NMR spectrum (DMSO-d_6), δ, ppm: 10.52, 10.89, 14.34, 38.97, 39.14, 39.31, 39.47, 39.57, 39.64, 39.81, 39.97, 42.41, 42.76, 59.17, 60.20, 116.73, 119.88, 123.52, 125.57, 126.49 (3C, Ar, C-CF_3), 126.74 (2C, Ar), 127.09 (2C, Ar), 128.19 (2C, Ar), 130.38 (2C, Ar), 132.25, 133.14, 139.10, 142.54, 160.48, 165.35, 168.68.

Mass spectrum (ESI), m/z$^+$: 610 (100%), 612 (75%), 611 (28%), 613 (21%), 614 (15%) $[M+H]^+$, 627 (26%), 629 (20%), 628 (9%), 630 (5%) $[M+NH_4]^+$, 632 (11%), 634 (7%) $[M+Na]^+$, 648 (52%), 650 (43%), 649 (15%), 651 (14%), 652 (12%), 653 (5%) $[M+K]^+$; m/z$^-$ 608 (100%), 610 (67%), 609 (28%), 611 (23%), 612 (14%) $[M-H]^-$, 644 (53%), 645 (18%) $[M+Cl]^-$, 646 (48%), 648 (19%), 647 (18%), 649 (6%) $[M+K-2H]^-$.

Anal. Calcd (%) for $C_{29}H_{27}Cl_2F_3N_3O_4$: C, 57.06; H, 4.62; N, 6.88. Found: C, 57.31; H, 4.31; N, 6.77.

Ethyl (*Z*)-4-(4-(benzylamino)-1-chloro-3-(2-chloro-*N*-(4-chlorophenyl)acetamido)-4-oxobut-1-en-1-yl)-3,5-dimethyl-1*H*-pyrrole-2-carboxylate (6e). Yield 93%, white solid, mp = 193-195 °C.

^{1}H NMR spectrum (DMSO-d_6), δ, ppm (J, Hz): 1.25 (t, 3H, -CH$_2$<u>CH$_3$</u>, J^3 7.1), 1.92 (s, 3H, 5-CH$_3$), 2.00 (s, 3H, 3-CH$_3$), 3.96 (d, 1H, -CH$_2$Cl, J^2 13.9), 4.13 (s, 1H, -CH$_2$Cl, J^2 14.2), 4.18 (q, 2H, <u>-CH$_2$</u>CH$_3$, J^3 7.0), 4.30 (dd, 1H, <u>CH$_2$</u>C$_6$H$_5$), 4.41 (dd, 1H, <u>CH$_2$</u>C$_6$H$_5$), 5.28 (d, 1H, =CH-, J^3 8.6), 5.76 (d, 1H, CH, J^3 8.6), 7.22-7.57 (m, 9H, CH$_2$<u>C$_6$H$_5$</u>, Ar), 8.84 (br.s, 1H, <u>NH</u>C(O)), 11.48 (s, 1H, <u>NHPyrrole</u>).

^{13}C NMR spectrum (DMSO-d_6), δ, ppm: 10.67, 11.02, 14.38, 38.97, 39.14, 39.30, 39.39, 39.47, 39.56, 39.64, 39.73, 39.80, 39.89, 39.97, 40.06, 42.38, 42.74, 59.19, 60.12, 116.75, 119.93, 123.61, 125.60, 126.72, 127.09 (2C, Ar), 128.17 (2C, Ar), 129.34 (2C, Ar), 131.34 (2C, Ar), 132.31, 132.81, 133.45, 137.64, 139.13, 160.51, 165.45, 168.66.

Mass spectrum (ESI), m/z$^+$: 576 (94%), 578 (91%), 580 (32%), 577 (28%), 579 (26%) [M+H]$^+$, 600 (33%), 598 (32%), 602 (13%), 599 (11%), 601 (9%) [M+Na]$^+$, 623 (100%), 621 (97%), 625 (40%), 622 (35%), 624 (34%) [M+2Na-H]$^+$, 641 (27%), 639 (23%), 640 (10%), 642 (10%) [M+CH$_3$CN+Na]$^+$; m/z$^-$: 576 (45%), 574 (41%), 578 (16%), 577 (15%), 575 (14%) [M-H]$^-$, 612 (100%), 610 (73%), 614 (51%), 613 (34%), 611 (17%), 615 (14%) [M+Cl]$^-$.

Anal. Calcd (%) for $C_{28}H_{28}Cl_3N_3O_4$: C, 58.30; H, 4.89; N, 7.28. Found: C, 57.99; H, 5.08; N, 7.59.

Ethyl (*Z*)-4-(1-chloro-3-(2-chloro-*N*-(4-methoxyphenyl)acetamido)-4-(cyclohexylamino)-4-oxobut-1-en-1-yl)-3,5-dimethyl-1*H*-pyrrole-2-carboxylate (7a). Yield 59%, yellow solid, mp = 94-95 °C.

^{1}H NMR spectrum (DMSO-d_6), δ, ppm (J, Hz): 1.09-1.33 (m, 8H, C$_6$H$_{11}$-, -CH$_2$<u>CH$_3$</u>,), 1.51-1.80 (5H, C$_6$H$_{11}$-), 1.98 (s, 5-CH$_3$, 3H), 2.06 (s, 3-CH$_3$, 3H), 3.57 (br. s, 1H, C$_6$H$_{11}$-), 3.75 (s, 3H, -OCH$_3$), 3.89 (d, 1H, -CH$_2$Cl, J^2 13.7), 4.06 (d, 1H, -CH$_2$Cl, J^2 13.6), 4.19 (q, 2H, -<u>CH$_2$</u>CH$_3$), 5.18 (d, =CH-, J^3 8.5), 5.68 (d, 1H, CH, J^3 8.4), 6.95-7.42 (m, 4H, Ar), 7.98 (d, 1H, <u>NH</u>C(O)), 11.46 (s, 1H, <u>NHPyrrole</u>).

^{13}C NMR spectrum (DMSO-d_6), δ, ppm: 11.27, 11.62, 14.91, 25.14 (2C), 25.66, 32.71, 32.81, 43.16, 48.54, 55.83, 59.69, 60.33, 114.79 (2C), 117.23, 120.66, 124.83 (2C), 126.16, 131.21, 131.73, 132.48, 132.87, 159.68, 161.07, 166.17, 168.15.

Mass spectrum (ESI), m/z$^+$: 564 (100%), 566 (75%), 565 (32%), 567 (23%), 568 (14%) [M+H]$^+$, 586 (23%), 588 (21%), 587 (5%), 589 (5%) [M+Na]$^+$, 602 (28%), 604 (27%), 603 (32%), 605 (23%) [M+K]$^+$, 627 (62%), 629 (51%), 628 (18%) [M+ACN+Na]$^+$; m/z$^-$: 562 (38%), 564 (31%), 565 (8%), 563 (5%) [M-H]$^-$, 600 (100%), 598 (88%), 602 (28%), 601 (27%), 599 (15%) [M+Cl]$^-$
.

Anal. Calcd (%) for $C_{29}H_{35}Cl_2N_3O_5$: C, 59.58; H, 6.25; N, 7.44. Found: C, 59.89; H, 6.38; N, 7.28.

Ethyl (*Z*)-4-(1-chloro-3-(2-chloro-*N*-(*p*-tolyl)acetamido)-4-(cyclohexylamino)-4-oxobut-1-en-1-yl)-3,5-dimethyl-1*H*-pyrrole-2-carboxylate (7b). Yield 87%, yellow solid, mp = 99-100 °C.

^{1}H NMR spectrum (DMSO-*d*$_6$), δ, ppm (*J*, Hz): 1.17-1.32 (m, 8H, C_6H_{11}-, -$CH_2$$\underline{CH_3}$), 1.49-1.78 (m, 5H, C_6H_{11}-), 1.96 (s, 3H, 5-CH_3), 2.04 (s, 3H, 3-CH_3), 2.29 s (3H, CH_3-Ar), 3.58 (br. s, 1H, C_6H_{11}-), 3.88 (d, 1H, -CH_2Cl, J^2 13.8), 4,05 (d, 1H, -CH_2Cl, J^2 14.0), 4.18 (q, 2H, -$\underline{CH_2}$$CH_3$), 5.18 (d, =CH-, J^3 8.4), 5.68 (d, 1H, CH, J^3 8.5), 7.21-7.38 (m, 4H, Ar), 8.00 d (1H, $\underline{NH}$C(O)), 11.47 (s, 1H, $\underline{NHPyrrole}$).

^{13}C NMR spectrum (DMSO-*d*$_6$), δ, ppm: 11.19, 11.55, 14.90, 21.10, 25.11, 25.16, 25.66, 32.71, 32.81, 43.09, 48.55, 59.68, 60.30, 117.23, 120.66, 124.79, 126.17, 129.78 (2C, Ar), 130.21 (2C, Ar), 132.57, 132.88, 136.65, 138.85, 161.07, 165.95, 168.10.

Mass spectrum (ESI), m/z$^+$: 548 (100%), 550 (78%), 549 (32%), 551 (25%), 552 (15%), 553 (4%) $[M+H]^+$, 570 (25%), 572 (18%) $[M+Na]^+$, 586 (29%), 588 (22%), 587 (11%), 589 (10%) $[M+K]^+$, 611 (70%), 613 (55%) $[M+CH_3CN+Na]^+$; m/z$^-$: 546 (31%), 548 (27%), 547 (9%) $[M-H]^-$, 584 (100%), 582 (78%), 586 (30%), 585 (29%), 583 (15%) $[M+Cl]^-$.

Anal. Calcd (%) for $C_{28}H_{35}Cl_2N_3O_4$: C, 61.31; H, 6.43; N, 7.66. Found: C, 61.04; H, 6.62; N, 7.49.

Ethyl (*Z*)-4-(3-(*N*-(4-bromophenyl)-2-chloroacetamido)-1-chloro-4-(cyclohexylamino)-4-oxobut-1-en-1-yl)-3,5-dimethyl-1*H*-pyrrole-2-carboxylate (7c). Yield 64%, white solid, mp = 138-139 °C.

^{1}H NMR spectrum (DMSO-*d*$_6$), δ, ppm (*J*, Hz): 1.13-1.26 (m, 8H, C_6H_{11}-, -$CH_2$$\underline{CH_3}$), 1.54-1.74 (m, 5H, C_6H_{11}-), 1.94 (s, 3H, 5-CH_3), 2.02 (s, 3H, 3-CH_3), 3.57 (br. s, 1H, C_6H_{11}-), 3.94 (d, 1H, -CH_2Cl, J^2 13.9), 4.12-4.21 (m, 3H, -CH_2Cl, -$\underline{CH_2}$$CH_3$), 5.17 (d, =CH-, J^3 8.3), 5.66 (d, 1H, CH, J^3 8.4), 7.49-7.63 (m, 4H, Ar), 8.12 (d, 1H, $\underline{NH}$C(O)), 11.49 (s, 1H, $\underline{NHPyrrole}$).

^{13}C NMR spectrum (DMSO-*d*$_6$), δ, ppm: 11.17, 11.53, 14.92, 25.08, 25.14, 25.65, 32.72, 32.81, 43.24, 48.56, 59.72, 60.33, 117.26, 120.48, 122.50, 124.48, 126.13, 132.26 (2C, Ar), 132.72 (2C, Ar), 132.90, 133.13, 138.60, 161.06, 165.73, 168.11.

Mass spectrum (ESI), m/z$^+$: 614 (28%), 612 (17%), 616 (12%) $[M+H]^+$, 652 (27%), 650 (15%), 654 (13%) $[M+K]^+$, 677 (100,0%), 675 (63%), 679 (58%), 678 (35%), 676 (21%), 680 (17%) $[M+CH_3CN+Na]^+$.

Anal. Calcd (%) for $C_{27}H_{32}BrCl_2N_3O_4$: C, 52.87; H, 5.26; N, 6.85. Found: C, 52.80; H, 5.35; N, 6.77.

Ethyl (*Z*)-4-(1-chloro-3-(2-chloro-*N*-(4-(trifluoromethyl)phenyl)acetamido)-4-(cyclohexylamino)-4-oxobut-1-en-1-yl)-3,5-dimethyl-1*H*-pyrrole-2-carboxylate (7d). Yield 79%, dark yellow solid, mp = 113-115 °C.

^{1}H NMR spectrum (DMSO-*d*6), δ, ppm (*J*, Hz): 1.13-1.26 (m, 8H, C_6H_{11}-, -CH2<u>CH3</u>,), 1.54-1.76 (m, 5H, C_6H_{11}-), 1.87 (s, 3H, 5-CH_3), 1.94 (s, 3H, 3-CH_3), 3.58 (br. s, 1H, C_6H_{11}-), 3.96 (d, 1H, -CH_2Cl, J^2 14.0), 4.15-4.20 (m, 3H, -CH_2Cl, -<u>CH2</u>CH3), 5.20 (d, =CH-, J^3 8.2), 5.70 (d, 1H, CH, J^3 8.2), 7.76-7.85 (m, 4H, Ar), 8.17 (d, 1H, <u>NH</u>C(O)), 11.47 (s, 1H, <u>NHPyrrole</u>).

^{13}C NMR spectrum (DMSO-*d*6), δ, ppm: 11.04, 11.41, 14.89, 25.06, 25.12, 25.65, 32.72, 32.80, 43.28, 48.59, 59.70, 60.46, 117.23, 120.42, 123.06-129.33 (2C, C-CF_3), 124.38, 126.09, 126.88 (2C, Ar), 129.33, 130.96 (2C, Ar), 132.83, 133.42, 143.06, 161.02, 165.68, 168.13.

Mass spectrum (ESI), m/z$^+$: 602 (28%), 604 (20%), 603 (11%), 605 (8%) $[M+H]^+$, 640 (18%), 642 (14%), 641 (6%), 643 (3%), 644 (2%) $[M+K]^+$, 665 (100%), 667 (77%), 666 (40%), 668 (27%) $[M+CH_3CN+Na]^+$.

Anal. Calcd (%) for $C_{28}H_{32}Cl_2F_3N_3O_4$: C, 55.82; H, 5.35; N, 6.97. Found: C, 55.91; H, 5.43; N, 6.78.

Ethyl (*Z*)-4-(1-chloro-3-(2-chloro-*N*-(4-chlorophenyl)acetamido)-4-(cyclohexylamino)-4-oxobut-1-en-1-yl)-3,5-dimethyl-1*H*-pyrrole-2-carboxylate (7e). Yield 72%, yellow solid, mp = 103-105 °C.

^{1}H NMR spectrum (DMSO-*d*6), δ, ppm (*J*, Hz): 1.07-1.34 (m, 8H, C_6H_{11}-, -CH2<u>CH3</u>,), 1.50-1.78 (m, 5H, C_6H_{11}-), 1.96 (s, 3H, 5-CH_3), 2.03 (s, 3H, 3-CH_3), 3.58 (br. s, 1H, C_6H_{11}-), 3.93 (d, 1H, -CH_2Cl, J^2 14.0), 4.13 (d, 1H, -CH_2Cl, J^2 14.0), 4.19 (q, 2H, -<u>CH2</u>CH3, J^3 7.3), 5.19 (d, =CH-, J^3 8.3), 5.67 (d, 1H, CH, J^3 8.6), 7.45-7.61 (m, 4H, Ar), 8.09 (d, 1H, <u>NH</u>C(O)), 11.48 (s, 1H, <u>NHPyrrole</u>).

^{13}C NMR spectrum (DMSO-*d*6), δ, ppm: 10.63, 11.01, 14.38, 24.57 (2C, Cy), 25.13, 32.26 (2C, Cy), 39.02, 39.19, 39.35, 39.52, 39.69, 39.78, 39.85, 39.95, 40.02, 42.64, 48.06, 59.16, 59.87, 116.77, 120.00, 123.99, 125.59, 129.21 (2C, Ar), 131.41 (2C, Ar), 132.34, 132.56, 133.38, 137.66, 160.53, 165.27, 167.56.

Mass spectrum (ESI), m/z$^+$: 570 (100%), 568 (98%), 572 (36%), 569 (28%), 571 (26%), 573 (10%), 574 (5%) $[M+H]^+$; 633 (37%), 631 (34%), 635 (14%), 632 (10%), 634 (10%) $[M+CH_3CN+Na]^+$; m/z$^-$: 566 (26%), 568 (26%), 567 (13%), 570 (10%), 569 (9%) $[M-H]^-$, 602

(71%), 603 (26%) [M+Cl]⁻; 604 (100%), 606 (51%), 605 (34%), 607 (16%), 608 (12%) [M+K-2H]⁻.

Anal. Calcd (%) for $C_{27}H_{32}Cl_3N_3O_4$: C, 57.00; H, 5.67; N, 7.39. Found: C, 56.91; H, 5.82; N, 7.48.

Ethyl (*Z*)-4-(4-(*tert*-butylamino)-1-chloro-3-(2-chloro-*N*-(4-methoxyphenyl)acetamido)-4-oxobut-1-en-1-yl)-3,5-dimethyl-1*H*-pyrrole-2-carboxylate (8a). Yield 65%, white solid, mp = 124-125 °C.

^{1}H NMR spectrum (DMSO-d_6), δ, ppm (*J*, Hz): 1.25 (m, 12H, -CH_2<u>CH_3</u>, *t*-Bu), 1.99 (s, 3H, 5-CH_3), 2.06 (s, 3H, 3-CH_3), 3.75 (s, 3H, -OCH_3), 3.90 (d, 1H, CH_2Cl, J^2 13.8), 4.05 (d, 1H, CH_2Cl, J^2 13.8), 4.19 (q, 2H, -<u>CH_2</u>CH_3, J^3 7.2), 5.16 (d, =CH-, J^3 8.6), 5.64 (d, 1H, CH, J^3 8.6), 6.95-7.42 (m, 4H, C_6H_4), 7.62 (s, 1H, <u>NH</u>(CO)), 11.45 (s, 1H, <u>NHPyrrole</u>).

^{13}C NMR spectrum (DMSO-d_6), δ, ppm: 10.71, 11.06, 14.37, 28.42 (3C, *t*-Bu), 42.60, 50.49, 55.30, 59.16, 60.22, 114.26 (2C, Ar), 116.73, 120.18, 124.49 (2C, Ar), 125.60, 130.70, 131.30, 131.93, 132.29, 159.15, 160.54, 165.64, 168.12.

Mass spectrum (ESI): m/z⁺: 538 (100%), 540 (78%), 539 (32%), 541 (21%), 542 (12%) [M+H]⁺; 576 (6%) [M+K]⁺; 601 (39%), 603 (30%), 602 (8%), [M+CH_3CN+Na]⁺; m/z⁻: 536 (100%), 538 (73%), 537 (32%), 539 (20%), [M-H]⁻; 572 (100%), 574 (100%), 576 (32%), 573 (31%), 575 (30%), [M+Cl]⁻.

Anal. Calcd (%) for $C_{26}H_{33}Cl_2N_3O_5$: C, 58.00; H, 6.18; N, 7.80. Found: C, 57.74; H, 6.03; N, 7.67.

Ethyl (*Z*)-4-(4-(*tert*-butylamino)-1-chloro-3-(2-chloro-*N*-(*p*-tolyl)acetamido)-4-oxobut-1-en-1-yl)-3,5-dimethyl-1*H*-pyrrole-2-carboxylate (8b). Yield 69%, yellowish solid, mp = 112-113 °C.

^{1}H NMR spectrum (DMSO-d_6), δ, ppm (*J*, Hz): 1.25 (m, 12H, -CH_2<u>CH_3</u>, *t*-Bu), 1.95 (s, 3H, 5-CH_3), 2.04 (s, 3H, 3-CH_3), 2.30 (s, 3H, Ar<u>CH_3</u>), 3.89 (d, 1H, CH_2Cl, J^2 13.8), 4.05 (d, 1H, CH_2Cl, J^2 13.8), 4.18 (q, 2H, -<u>CH_2</u>CH_3, J^3 7.1), 5.13 (d, =CH-, J^3 8.5), 5.63 (d, 1H, CH, J^3 8.5), 7.21-7.38 (m, 4H, C_6H_4), 7.69 (s, 1H, <u>NH</u>(CO)), 11.48 (s, 1H, <u>NHPyrrole</u>).

^{13}C NMR spectrum (DMSO-d_6), δ, ppm: 11.18, 11.53, 14.91, 21.12, 28.96 (3C, *t*-Bu), 43.10, 51.03, 59.70, 60.68, 117.23, 120.68, 124.97, 126.12, 129.81 (2C, Ar), 130.19 (2C, Ar), 132.54, 132.83, 136.70, 138.81, 161.06, 165.92, 168.59.

Mass spectrum (ESI), m/z⁺: 522 (100%), 524 (79%), 523 (30%), 525 (21%), 526 (18%), 527 (3%) [M+H]⁺, 560 (30%), 562 (22%), 561 (8%), 563 (7%), 564 (5%) [M+K]⁺, 585 (80%), 587

(55%), 586 (24%), 588 (20%), 589 (11%), 590 (4%) [M+CH_3CN+Na]$^+$; m/z$^-$: 520 (100%), 522 (68%), 521 (30%), 523 (20%) [M-H]$^-$; 558 (92%), 556 (85%), 560 (32%), 557 (28%) [M+Cl]$^-$.
Anal. Calcd (%) for $C_{26}H_{33}Cl_2N_3O_4$: C, 59.77; H, 6.37; N, 8.04. Found: C, 59.44; H, 6.28; N, 7.89.

Ethyl (*Z*)-4-(3-(*N*-(4-bromophenyl)-2-chloroacetamido)-4-(*tert*-butylamino)-1-chloro-4-oxobut-1-en-1-yl)-3,5-dimethyl-1*H*-pyrrole-2-carboxylate (8c). Yield 79%, yellowish solid, mp = 122-123 °C.
^{1}H NMR spectrum (DMSO-d_6), δ, ppm (*J*, Hz): 1.25 (m, 12H, -CH_2<u>CH_3</u>, *t*-Bu), 1.94 (s, 3H, 5-CH_3), 2.02 (s, 3H, 3-CH_3), 3.95 (d, 1H, CH_2Cl, J^2 14.3), 4.13 (d, 2H, CH_2Cl), 4.17 (q, 2H, -<u>CH_2</u>CH_3, *J*^{3} 7.1), 5.13 (d, 1H, =CH-, *J*^{3} 8.3), 5.63 (d, 1H, CH, *J*^{3} 8.3), 7.49-7.64 (m, 4H, C_6H_4), 7.83 (s, 1H, <u>NH(CO)</u>), 11.50 (s, 1H, <u>NHPyrrole</u>).
^{13}C NMR spectrum (DMSO-d_6), δ, ppm: 11.10, 11.46, 14.87, 28.92 (3C, *t*-Bu), 43.21, 51.03, 59.68, 60.69, 117.25, 120.50, 122.43, 124.68, 126.04, 132.28 (2C, Ar), 132.66 (2C, Ar), 132.81, 133.04, 138.63, 161.01, 165.64, 168.58.
Mass spectrum (ESI), m/z$^+$: 588 (100%), 586 (67%), 590 (55%), 589 (30%), 587 (15%), 591 (14%), 592 (9%) [M+H]$^+$, 628 (13%), 624 (7%), 626 (5%) [M+K]$^+$, 651 (40%), 649 (24%), 653 (21%), 652 (14%), 650 (6%), 653 (6%) [M+ACN+Na]$^+$; m/z$^-$: 586 (16%), 584 (10%), 588 (9%), 587 (5%), 585 (4%) [M-H]$^-$, 622 (100%) 624 (65%), 620 (49%), 623 (37%), 625 (22%), 621 (21%) [M-Cl]$^-$.
Anal. Calcd (%) for $C_{25}H_{30}BrCl_2N_3O_4$: C, 51.12; H, 5.15; N, 7.15. Found: C, 50.73; H, 5.28; N, 7.01.

Ethyl (*Z*)-4-(4-(*tert*-butylamino)-1-chloro-3-(2-chloro-*N*-(4-(trifluoromethyl)phenyl)acetamido)-4-oxobut-1-en-1-yl)-3,5-dimethyl-1*H*-pyrrole-2-carboxylate (8d). Yield 64%, white solid, mp = 103-104 °C.
^{1}H NMR spectrum (DMSO-d_6), δ, ppm (*J*, Hz): 1.23 (t, 3H, -CH_2<u>CH_3</u>, *J*^{3} 7.1), 1.27 (s, 9H, *t*-Bu), 1.88 (s, 3H, 5-CH_3), 1.95 (s, 3H, 3-CH_3), 3.98 (d, CH_2Cl, *J*^{2} 14.2), 4.15-4.20 (m, 3H, CH_2Cl, -<u>CH_2</u>CH_3,), 5.16 (d, =CH-, *J*^{3} 8.2), 5.68 (d, 1H, CH, *J*^{3} 8.2), 7.79-7.84 (m, 4H, C_6H_4), 7.89 (s, 1H, <u>NH(CO)</u>), 11.47 (s, 1H, <u>NHPyrrole</u>).
^{13}C NMR spectrum (DMSO-d_6), δ, ppm: 10.98, 11.35, 14.84, 28.91 (3C, *t*-Bu), 43.25, 51.05, 59.65, 60.81, 117.22, 120.45, 121.68-127.09 (1C, <u>CF_3</u>-C^{Ar}) 124.61, 126.01, 126.82 (2C, Ar), 129.35-129.56 (1C, CF_3-<u>C</u>^{Ar}), 131.01 (2C, Ar), 132.74, 133.32, 143.10, 160.98, 165.58, 168.57.

Mass spectrum (ESI), m/z$^+$: 576 (18%), 578 (16%), 577 (3%) [M+H]$^+$; 614 (17%), 616 (12%), 615 (4%) [M+K]$^+$, 639 (100%), 641 (80%), 640 (32%), 642 (25%), 643 (17%), 644 (4%) [M+CH_3CN+Na]$^+$; m/z$^-$: 574 (71%), 576 (46%), 575 (12%), 577 (10%), 578 (9%), 579 (3%) [M-H]$^-$, 612 (100%), 610 (91%), 614 (38%), 611 (16%), 613 (26%), 615 (10%) [M+Cl]$^-$.
Anal. Calcd (%) for $C_{26}H_{30}Cl_2F_3N_3O_4$: C, 54.18; H, 5.25; N, 7.29. Found: C, 54.23; H, 5.06; N, 7.24.

Ethyl (*Z*)-4-(4-(*tert*-butylamino)-1-chloro-3-(2-chloro-*N*-(4-chlorophenyl)acetamido)-4-oxobut-1-en-1-yl)-3,5-dimethyl-1*H*-pyrrole-2-carboxylate (8e). Yield 58%, white solid, mp = 112-114 °C.
^{1}H NMR spectrum (DMSO-d_6), δ, ppm (*J*, Hz): 1.24 (t, 3H, -$CH_2\underline{CH_3}$, J^3 7.0), 1.27 (s, 9H, *t*-Bu), 1.96 (s, 3H, 5-CH_3), 2.03 (s, 3H, 3-CH_3), 3.95 (d, CH_2Cl, J^2 14.1), 4.12 (d, 1H, CH_2Cl, J^2 14.0), 4.19 (q, 2H, -$\underline{CH_2}CH_3$, J^3 7.0), 5.15 (d, =CH-, J^3 8.4), 5.64 (d, 1H, CH, J^3 8.3), 7.46-7.60 (m, 4H, C_6H_4), 7.78 (s, 1H, <u>NH</u>Bu-t), 11.47 (s, 1H, <u>NHPyrrole</u>).
^{13}C NMR spectrum (DMSO-d_6), δ, ppm: 10.61, 10.98, 14.36, 28.42 (3C, t-Bu), 39.17, 39.34, 39.50, 39.67, 39.77, 39.84, 40.00, 42.66, 50.54, 59.16, 60.26, 116.78, 120.03, 124.20, 125.54, 129.19 (2C, Ar), 131.46 (2C, Ar), 132.29, 132.52, 133.34, 137.72, 160.52, 165.22, 168.05.
Mass spectrum (ESI), m/z$^+$: 544 (100%), 542 (98%), 546 (37%), 545 (26%), 543 (25%), 547 (10%), 548 (4%) [M+H]$^+$, 607 (61%), 605 (59%), 609 (23%), 608 (19%), 606 (18%), 610 (6%) [M+CH_3CN+Na]$^+$; m/z$^-$: 540 (42%), 542 (40%), 541 (15%), 544 (13%), 543 (12%) [M-H]$^-$, 576 (75%), 577 (29%) [M+Cl]$^-$, 578 (100%), 580 (52%), 579 (34%), 581 (15%), 582 (12%) [M+K-2H]$^-$.
Anal. Calcd (%) for $C_{25}H_{30}Cl_3N_3O_4$: C, 55.31; H, 5.57; N, 7.74. Found: C, 55.07; H, 5.62; N, 7.65.

Post-Ugi transformations

Procedure A

Ethyl (*Z*)-4-(1-chloro-3-(2-chloro-*N*-(4-(trifluoromethyl)phenyl)acetamido)-4-((2-nitrobenzyl)amino)-4-oxobut-1-en-1-yl)-3,5-dimethyl-1*H*-pyrrole-2-carboxylate (**5d**, 0.3 mmol) and 3 mL of MeOH were added to a 10 mL screw cap vial. While stirring, 36% aqueous HCl solution (1.5 mmol) was added dropwise. The vial was hermetically closed and the mixture stirred for 3 hours immersed in a glycerol bath with a temperature of 80 °C. After that, the reaction mixture was cooled to 4 °C and left overnight. The formed precipitate was collected by filtration, washed with cold solvent, then dried in a vacuum drying oven at a temperature of 30 °C.

Procedure B

Ethyl (*Z*)-4-(1-chloro-3-(2-chloro-*N*-(4-(trifluoromethyl)phenyl)acetamido)-4-((2-nitrobenzyl)amino)-4-oxobut-1-en-1-yl)-3,5-dimethyl-1*H*-pyrrole-2-carboxylate (**5d**, 0.3 mmol) and 3 mL of EtOH were added to a 10 mL screw cap vial. While stirring, 36% aqueous HCl solution (1.5 mmol) was added dropwise. The vial was hermetically closed and the mixture stirred for 15 minutes under MW irradiation with a temperature of 120 °C. After that, the reaction mixture was cooled to 4 °C and left overnight. The formed precipitate was collected by filtration, washed with cold solvent, then dried in a vacuum drying oven at a temperature of 30 °C.

Procedure C

Ethyl (*Z*)-4-(1-chloro-3-(2-chloro-*N*-(4-(trifluoromethyl)phenyl)acetamido)-4-((2-nitrobenzyl)amino)-4-oxobut-1-en-1-yl)-3,5-dimethyl-1*H*-pyrrole-2-carboxylate (**5d**, 0.3 mmol) and 3 mL of MeCN were added to a 10 mL screw cap vial. While stirring, 36% aqueous HCl solution (1.5 mmol) was added dropwise. The vial was hermetically closed and the mixture stirred for 20 minutes under MW irradiation with a temperature of 100 °C. After that, the reaction mixture was cooled to 4 °C and left overnight. The formed precipitate was collected by filtration and washed with cold solvent, then dried in a vacuum drying oven at a temperature of 30 °C.

General procedure for acidic post-transformations of bisamides

The corresponding bisamide **5–8** (0.3 mmol) and 3 mL of EtOH or MeCN were added to a 10 mL screw cap vial (see Table 2). In some cases, while stirring 36% aqueous HCl solution

(0.15–1.5 mmol) or MCA (0.3 mmol) was added to the mixture (see Table 2). The vial was hermetically closed and the mixture stirred for 2.5–6 h immersed in a glycerol bath heated to 50–80 °C or for 72–850 h in a glycerol bath at 25 °C (see Table 2). After that, the reaction mixture was cooled to 4 °C and left overnight. The formed precipitate was collected by filtration and washed with cold solvent, then dried in a vacuum drying oven at a temperature of 30 °C. The first precipitate was purified by recrystallization from MeCN or MTBE. Additional products were isolated by evaporating the mother liquor under vacuum and separating the crude mixture using solvents as MTBE, EtOAc, DCM and MeCN and/or using silica gel column chromatography, eluent $CHCl_3$/EtOAc.

In this way, amides **10a**–**d** and ketobisamides **12a**–**e** were obtained:

Ethyl (*E*)-3,5-dimethyl-4-(4-((2-nitrobenzyl)amino)-3,4-dioxobut-1-en-1-yl)-1*H*-pyrrole-2-carboxylate (10a). Yield 42%, yellow solid, mp = 223 °C (with decomp.).
^{1}H NMR spectrum (DMSO-d_6), δ, ppm (*J*, Hz): 1.30 (t, 3H, -$CH_2\underline{CH_3}$, J^3 7.0), 2.39 (s, 3H, 5-CH_3), 2.42 (s, 3H, 3-CH_3), 4.25 (q, 2H, -$\underline{CH_2}CH_3$, J^3 7.3), 4.66 (d, 2H, -$\underline{CH_2}C_6H_4$), 7.12 (d, 1H, -CH=, J^3 16.1), 7.52-8.05 (m, 4H, -$CH_2\underline{C_6H_4}$), 7.83 (d, 1H, CH-, J^3 16.4) 9.24 (t, 1H, NH(CO)), 11.99 (s, 1H, NHPyrrole).
^{13}C NMR spectrum (DMSO-d_6), δ, ppm: 11.19, 12.38, 14.34, 40.37, 59.59, 114.23, 117.31, 118.57, 124.55, 128.11, 128.35, 129.30, 133.32, 133.80, 138.97, 140.24, 147.93, 160.56, 163.17, 185.11.
Mass spectrum (ESI), m/z$^+$: 400 (100%), 401(29%), 603 (7%) [M+H]$^+$, 438 (32%), 439 (8%), 440 (5%) [M+K]$^+$; 463 (86%), 464 (22%) [M+CH_3CN+Na]$^+$; m/z$^-$: 398 (100%), 399 (32%), 400 (6%) [M+H]$^-$.
Anal. Calcd (%) for $C_{20}H_{21}N_3O_6$: C, 60.14; H, 5.30; N, 10.52. Found: C, 59.81; H, 5.39; N, 10.59.

Ethyl (*E*)-4-(4-(benzylamino)-3,4-dioxobut-1-en-1-yl)-3,5-dimethyl-1*H*-pyrrole-2-carboxylate (10b). Yield 42%, greenish yellow solid, mp = 208-209 °C.
^{1}H NMR spectrum (DMSO-d_6), δ, ppm (*J*, Hz): 1.30 (t, 3H, -$CH_2\underline{CH_3}$, J^3 7.1), 2.39 (s, 3H, 5-CH_3), 2.42 (s, 3H, 3-CH_3), 4.25 (q, 2H, $\underline{-CH_2}$-CH_3, J^3 7.2), 4.36 (d, 2H, $\underline{CH_2}C_6H_5$, J^3 6.3), 7.14 (d, 1H, =CH-, J^3 16.3), 7.21-7.35 (m, 5H, $CH_2\underline{C_6H_5}$), 7.81 (1H, CH, J^3 16.2), 9.20 (t, 1H, NH(CO)), 11.99 (s, 1H, NHPyrrole).

^{13}C NMR spectrum (DMSO) δ, ppm: 11.17, 12.36, 14.33, 39.01, 39.18, 39.35, 39.51, 39.60, 39.68, 39.77, 39.85, 39.94, 40.01, 40.11, 42.14, 59.55, 114.57, 117.29, 118.56, 126.84, 127.34 (2C, Ar), 128.05, 128.24 (2C, Ar), 138.76, 138.85, 140.03, 160.56, 162.96, 185.77.
Mass spectrum (ESI), m/z$^+$: 355 (89%), 356 (22%) $[M+H]^+$; 418 (100%), 419 (27%), 420 (4%) $[M+CH_3CN+Na]^+$; m/z$^-$: 353 (100%), 354 (19%) $[M-H]^-$; 389 (8%) $[M+Cl]^-$.
Anal. Calcd (%) for $C_{20}H_{22}N_2O_4$: C, 67.78; H, 6.26; N, 7.90. Found: C, 67.60; H, 6.32; N, 7.80.

Ethyl (*E*)-4-(4-(cyclohexylamino)-3,4-dioxobut-1-en-1-yl)-3,5-dimethyl-1*H*-pyrrole-2-carboxylate (10c). Yield 40%, yellow solid, mp = 193-195 °C.
^1H NMR spectrum (DMSO-d_6), δ, ppm (*J*, Hz): 1.08-1.13 (m, 1H, C_6H_{11}-), 1.22-1.36 m (8H, C_6H_{11}-, -$CH_2\underline{CH_3}$,), 1.56-1.73 (m, 4H, C_6H_{11}-), 2.38 (s, 3H, 5-CH_3), 2.41 (s, 3H, 3-CH_3), 3.62 (m, 1H, C_6H_{11}-), 4.25 (q, 2H, -$\underline{CH_2}CH_3$), 7.07 (d, 1H, -CH=, J^3 16.2), 7.77 (d, 1H, CH, J^3 16.2) 8.40 (d, 1H, NH(CO)), 11.98 (s, 1H, <u>NHPyrrole</u>).
^{13}C NMR spectrum (DMSO) δ, ppm: 11.16, 12.36, 14.33, 24.71 (2C, Cy), 25.01, 31.85 (2C, Cy), 47.93, 59.55, 114.90, 117.23, 118.49, 127.97, 138.59, 139.81, 160.56, 162.13, 186.33.
Mass spectrum (ESI), m/z$^+$: 347 (100%), 348 (22%), 349 (3%) $[M+H]^+$; 410 (10%), 411 (2%) $[M+CH_3CN+Na]^+$; m/z$^-$: 345 (100%), 346 (19%), 347 (3%) $[M-H]^-$; 381 (6%), 383 (2%) $[M+Cl]^-$
.
Anal. Calcd (%) for $C_{19}H_{26}N_2O_4$: C, 65.88; H, 7.57; N, 7.90. Found: C, 65.59; H, 7.71; N, 8.03.

Ethyl (*E*)-4-(4-(*tert*-butylamino)-3,4-dioxobut-1-en-1-yl)-3,5-dimethyl-1*H*-pyrrole-2-carboxylate (10d). Yield 64%, yellow solid, mp = 192-193 °C.
^1H NMR spectrum (DMSO-d_6), δ, ppm (*J*, Hz): 1.30 (t, 3H, -$CH_2\underline{CH_3}$, J^3 7.1), 1.34 (s, 9H, *t*-Bu), 2.39 (s, 3H, 5-CH_3), 2.42 (s, 3H, 3-CH_3), 4.25 (q, 2H, -$\underline{CH_2}CH_3$, J^3 7.1), 7.05 (d, 1H, -CH=, J^3 16.3), 7.74 (d, 1H, CH, J^3 16.3), 7.84 (s, 1H, NH(CO)), 12.0 (s, 1H, <u>NHPyrrole</u>).
^1H NMR spectrum ($CDCl_3$), δ, ppm (*J*, Hz): 1.39 (t, 3H, -$CH_2\underline{CH_3}$, J^3 7.1), 1.44 (s, 9H, *t*-Bu), 2.50 (s, 3H, 5-CH_3), 2.53 (s, 3H, 3-CH_3), 4.35 (q, 2H, -$\underline{CH_2}CH_3$, J^3 7.1), 7.17 (s, 1H, NH(CO)), 7.50 (d, 1H, CH, J^3 16.2), 7.96 (d, 1H, =CH-, J^3 16.4), 9.22 (s, 1H, <u>NHPyrrole</u>).
^{13}C NMR spectrum (DMSO-d_6), δ, ppm: 11.15, 12.35, 14.33, 28.05 (3C, *t*-Bu), 38.95, 39.11, 39.28, 39.45, 39.53, 39.61, 39.70, 39.78, 39.87, 39.95, 50.56, 59.56, 114.35, 117.21, 118.49, 127.98, 138.62, 140.00, 160.56, 162.61, 186.60.
Mass spectrum (ESI), m/z$^+$: 321 (38%), 322 (8%) $[M+H]^+$; 383 (100%), 384 (22%), 385 (3%) $[M+CH_3CN+Na]^+$; m/z$^-$: 319 (100%), 320 (18%), 321 (3%) $[M-H]^-$; 355 (19%), 357 (6%), 356 (4%) $[M+Cl]^-$.

Anal. Calcd (%) for $C_{17}H_{24}N_2O_4$: C, 63.73; H, 7.55; N, 8.74. Found: C, 63.79; H, 7.63; N, 8.91.

Ethyl 4-(3-(2-chloro-*N*-(4-(trifluoromethyl)phenyl)acetamido)-4-((2-nitrobenzyl)amino)-4-oxobutanoyl)-3,5-dimethyl-1*H*-pyrrole-2-carboxylate (12a). Yield 20%, light purple solid, mp = 107-109 °C (with decomp.).

^{1}H NMR spectrum (DMSO-d_6), δ, ppm (J, Hz):, 1.2 (t, 3H, -$CH_2\underline{CH_3}$, J^3 7.2), 2.29 (s, 3H, 5-CH_3), 2.35 (s, 3H, 3-CH_3), 2.97 (dd, 1H, -$\underline{CH_2}$CH-, J^2 17.1, J^3 6.0), 3.17 (dd, 1H, -$\underline{CH_2}$CH-, J^2 17.1, J^3 6.1), 4.03 (d, 2H, CH_2Cl) 4.22 (q, 2H, -$\underline{CH_2}CH_3$-, J^3 7.3), 4.59 (d, 2H, $\underline{CH_2}C_6H_4$, J^2 6.0) 5.58 (br.s, CH), 7.50-8.04 (m, 8H, *p*-C_6H_4, *o*-C_6H_4), 8.43 (br.s, 1H, NH(CO)), 11.79 (s, 1H, $\underline{\text{NH}}$Pyrrol).

Mass spectrum (ESI), m/z$^+$: 637 (100%), 639 (41%), 638 (33%), 640 (11%), 641 (8%) $[M+H]^+$; 659 (48%), 661 (20%), 660 (13%) $[M+Na]^+$; 675 (34%), 677 (17%), 676 (10%) $[M+K]^+$; 700 (20%), 702 (10%), 701 (6%) $[M+ACN+Na]^+$; m/z$^-$: 635 (100%), 637 (38%), 636 (34%), 638 (11%) $[M-H]^-$; 671 (36%), 673 (24%), 672 (13%), 674 (9%) $[M+Cl]^-$.

Anal. Calcd (%) for $C_{29}H_{28}ClF_3N_4O_7$: C, 54.68; H, 4.43; N, 8.80. Found: C, 54.95; H, 4.65; N, 8.69.

Ethyl 4-(4-(benzylamino)-3-(2-chloro-*N*-(4-methoxyphenyl)acetamido)-4-oxobutanoyl)-3,5-dimethyl-1*H*-pyrrole-2-carboxylate (12b).

Mass spectrum (ESI), m/z$^+$: 554 (100%), 556 (40%), 555 (35%), 557 (12%) $[M+H]^+$; 576 (22%), 578 (6%) $[M+Na]^+$; m/z$^-$: 552 (100%), 554 (37%), 553 (35%), 555 (10%) $[M-H]^-$; 588 (14%), 590 (7%) $[M+Cl]^-$.

Ethyl 4-(4-(benzylamino)-3-(*N*-(4-bromophenyl)-2-chloroacetamido)-4-oxobutanoyl)-3,5-dimethyl-1*H*-pyrrole-2-carboxylate (12c). Yield 9%, white solid, mp = 229-231 °C.

^{1}H NMR spectrum (DMSO-d_6), δ, ppm (J, Hz):, 1.28 (t, 3H, -$CH_2\underline{CH_3}$, J^3 7.1), 2.26 (m, 6H, 5-CH_3, 3-CH_3), 2.68 (d, 1H, -$\underline{CH_2}$CH-, J^2 13.5), 3.10-3.40 (1H, -$\underline{CH_2}$CH-), 3.92 (d, 1H, CH_2Cl, J^2 13.1) 4.22 (q, 2H, -$\underline{CH_2}CH_3$-, J^3 7.0), 4.35 (br.s, 1H, CH_2Cl), 4.53 (d, 1H, $\underline{CH_2}C_6H_5$, J^2 15.0), 4.69 (d, 1H, $\underline{CH_2}C_6H_5$, J^2 15.3), 5.99 (br.s, 1H, CH), 7.07-7.52 (m, 9H, -C_6H_4, -C_6H_5), 8.43 (s, 1H, NH(CO)), 11.66 (s, 1H, $\underline{\text{NHPyrrole}}$).

Mass spectrum (ESI), m/z$^+$: 602 (100%), 600 (75%), 603 (30%), 604 (27%), 601 (21%), 605 (7%) $[M+H]^+$; 645 (2%) $[M+CH_3CN+H]^+$.

Anal. Calcd (%) for $C_{28}H_{29}BrClN_3O_5$: C, 55.78; H, 4.85; N, 6.97. Found: C, 55.69; H, 4.91; N, 7.05.

Ethyl 4-(3-(2-chloro-*N*-(*p*-tolyl)acetamido)-4-(cyclohexylamino)-4-oxobutanoyl)-3,5-dimethyl-1*H*-pyrrole-2-carboxylate (12d). Yield 71%, yellowish solid, mp = 100-102 °C.

^{1}H NMR spectrum (DMSO-d_6), δ, ppm (*J*, Hz): 1.08-1.24 (m, 6H, Cy), 1.27 (t, 3H, OCH$_2$<u>CH$_3$</u>, J^3 7.1), 2.26-2.32 (m, 6H, 5-CH$_3$, 3-CH$_3$), 2.35 (s, 3H, p-CH$_3$), 2.77 (dd, 1H, -<u>CH$_2$</u>CH-, J^2 17.2, J^3 6.9), 3.10 (1H, -<u>CH$_2$</u>CH-, J^2 17.2, J^3 7.1), 3.45 (m, 1H, Cy), 3.84 (d, 1H, CH$_2$Cl, J^2 14.0), 3.92 (d, 1H, CH$_2$Cl, J^2 13.9) 4.22 (q, 2H, -<u>CH$_2$</u>CH$_3$-, J^3 7.1), 5.51 (t, 1H, CH, J^3 6.9), 7.15-7.26 (m, 4H, -C$_6$H$_4$), 7.86 (d, 1H, NH(CO), J^3 7.9), 11.76 (s, 1H, <u>NHPyrrole</u>).

^{13}C NMR spectrum (DMSO-d_6), δ, ppm: 12.21, 14.19, 14.41 (2C, Cy), 20.65, 24.64 (2C, Cy), 25.24, 32.27, 38.89, 39.10, 39.31, 39.52, 39.73, 39.77, 39.94, 39.98, 40.15, 41.97, 43.08, 47.97, 55.52, 59.57, 117.44, 121.97, 128.45, 129.61 (2C, Ar), 129.75 (2C, Ar), 135.29, 138.36, 138.49, 160.75, 165.51, 168.15, 193.20.

Mass spectrum (ESI), m/z$^+$: 530 (91%), 532 (36%), 531 (29%), 533 (9%) [M+H]$^+$, 568 (12%), 570 (6%) [M+K]$^+$, 631 (100%), 633 (42%), 632 (39%), 634 (13%) [M+TEA+H]$^+$; 528 (100%), 530 (39%), 529 (35%), 531 (11%) [M-H]$^-$, 564 (5%) [M+Cl]$^-$, 626 (56%), 628 (24%), 627 (19%) [M+HSO$_4$]$^-$.

Anal. Calcd (%) for $C_{28}H_{36}ClN_3O_5$: C, 63.45; H, 6.85; N, 7.93. Found: C, 63.24; H, 6.77; N, 8.05.

Ethyl 4-(3-(*N*-(4-bromophenyl)-2-chloroacetamido)-4-(*tert*-butylamino)-4-oxobutanoyl)-3,5-dimethyl-1*H*-pyrrole-2-carboxylate (12e). Yield 15% (46% in MeCN), white solid, mp = 215-217 °C.

^{1}H NMR spectrum (DMSO-d_6), δ, ppm (*J*, Hz): 1.20 (s, 9H, *t*-Bu), 1.28 (t, 3H, -CH$_2$<u>CH$_3$</u>, J^3 6.9), 2.30 (s, 3H, 5-CH$_3$), 2.37 (s, 3H, 3-CH$_3$), 2.77 (d, 1H, -<u>CH$_2$</u>CH-, J^2 14.5), 3.10 (d, 1H, -<u>CH$_2$</u>CH-, J^2 15.0), 3.95 (dd, 2H, CH$_2$Cl, J^2 14.0) 4.24 (q, 2H, -<u>CH$_2$</u>CH$_3$-, J^3 7.0), 5.44 (br.s, 1H, CH), 7.34-7.61 (m, 4H, -C$_6$H$_4$), 7.64 (s, 1H, NH(CO)), 11.78 (s, 1H, <u>NHPyrrole</u>).

^{13}C NMR (DMSO) δ, ppm: 12.13, 14.09, 14.33, 28.27 (3C, *t*-Bu), 38.96, 39.13, 39.30, 39.47, 39.56, 39.63, 39.72, 39.80, 39.89, 39.97, 41.85, 43.09, 50.31, 56.16, 59.49, 117.41, 121.93 (2C, Ar), 122.08, 128.28, 132.13 (3C, Ar), 137.16, 138.23, 160.65, 165.11, 168.37, 193.15.

Mass spectrum (ESI), m/z$^+$: 570 (100%), 568 (71%), 572 (29%), 571 (28%), 569 (21%), 573 (6%) [M+H]$^+$, 592 (7%), 590 (4%) [M+Na]$^+$, 608 (6%), 606 (4%) [M+K]$^+$, 663 (15%), 661 (12%), 664 (5%), 665 (4%) [M+ACN+Na]$^+$; m/z$^-$ 568 (100%), 566 (79%), 569 (29%), 570 (26%), 567 (25%), 571 (7%) [M-H]$^-$, 604 (10%), 602 (6%), 606 (5%) [M+Cl]$^-$.

Anal. Calcd (%) for $C_{25}H_{31}BrClN_3O_5$: C, 52.78; H, 5.49; N, 7.39. Found: C, 52.70; H, 5.39; N, 7.25.

Ethyl 4-(4-(*tert*-butylamino)-3-(2-chloro-*N*-(4-methoxyphenyl)acetamido)-4-oxobutanoyl)-3,5-dimethyl-1*H*-pyrrole-2-carboxylate (12f). Yield 21%, white solid, mp = 190-191 °C.

^{1}H NMR spectrum (DMSO-d_6), δ, ppm (*J*, Hz): 1.21 (s, 9H, *t*-Bu), 1.28 (t, 3H, -CH$_2$<u>CH$_3$</u>, J^3 7.1), 2.29 (s, 3H, 5-CH$_3$), 2.36 (s, 3H, 3-CH$_3$), 2.75 (dd, 2H, -<u>CH$_2$</u>CH-, J^2 17.2, J^3 6.8), 3.05 (dd, 2H, -<u>CH$_2$</u>CH-, J^2 17.2, J^3 7.1), 3.73 (s, 3H, -OCH$_3$), 3.87 (d, 1H, -CH$_2$Cl, J^2 13.8), 3.94 (d, 1H, -CH$_2$Cl, J^2 13.8), 4.23 (q, 2H, -<u>CH$_2$</u>CH$_3$, J^3 7.2), 5.44 (t, 1H, -CH$_2$CH-, J^3 6.8), 6.92-7.27 (m, 4H, -C$_6$H$_4$), 7.51 (s, 1H, <u>NH</u>(CO)), 11.75 (s, 1H, <u>NHPyrrole</u>).

^{13}C NMR (DMSO) δ, ppm: 12.12, 14.08, 14.32, 28.32 (3C, *t*-Bu), 38.95, 39.11, 39.28, 39.37, 39.45, 39.54, 39.62, 39.71, 39.78, 39.87, 39.95, 40.04, 41.84, 42.96, 50.26, 55.31, 55.95, 59.49, 114.23 (2C, Ar), 117.37, 122.04 (2C, Ar), 128.31, 130.24, 131.00, 138.16, 159.15, 160.67, 165.75, 168.61, 193.29.

Mass spectrum (ESI), m/z$^+$: 520 (100%), 522 (42%), 521 (36%), 523 (11%) [M+H]$^+$, 542 (49%), 544 (19%), 543 (15%), 545 (5%) [M+Na]$^+$, 557 (16%), 559 (7%), 558 (4%) [M+K]$^+$, 583 (69%), 585 (29%), 584 (20%), 586 (8%) [M+ACN+Na]$^+$; m/z$^-$: 518 (94%), 520 (36%), 519 (31%), 522 (12%) [M-H]$^-$, 554 (11%), 556 (7%), 555 (3%) [M+Cl]$^-$, 632 (100%), 634 (41%), 633 (36%), 635 (13%) [M+TFA-H]$^-$.

Anal. Calcd (%) for $C_{26}H_{34}ClN_3O_6$: C, 60.05; H, 6.59; N, 8.08. Found: C, 59.96; H, 6.46; N, 7.99.

^{1}H NMR and ^{13}C NMR spectra of compounds 5–8, 10a–d, and 12a,c–f

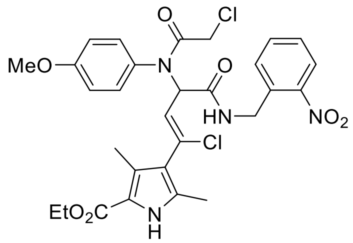

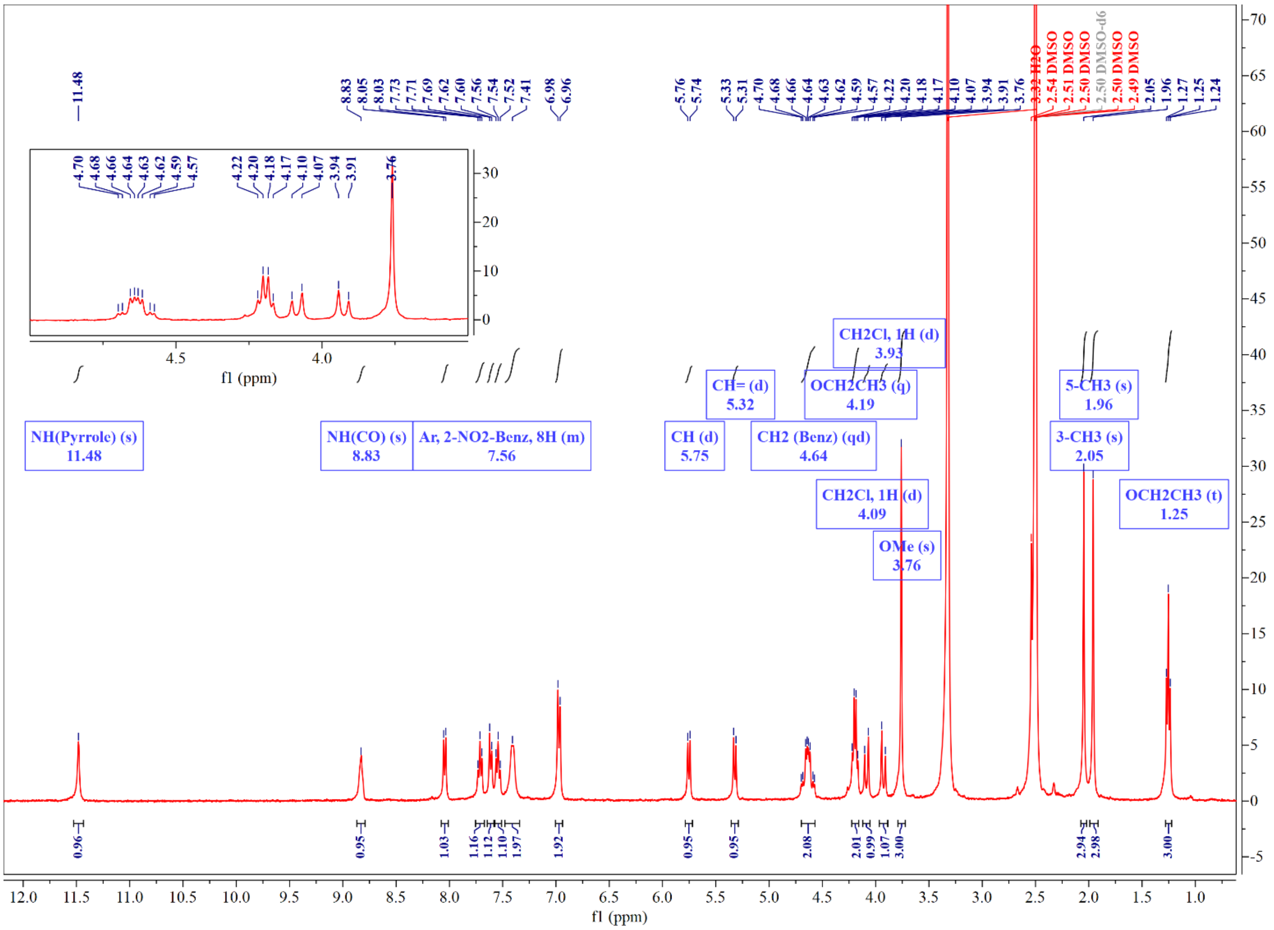

Figure S1. ^{1}H NMR spectrum of compound **5a** in DMSO-d_6.

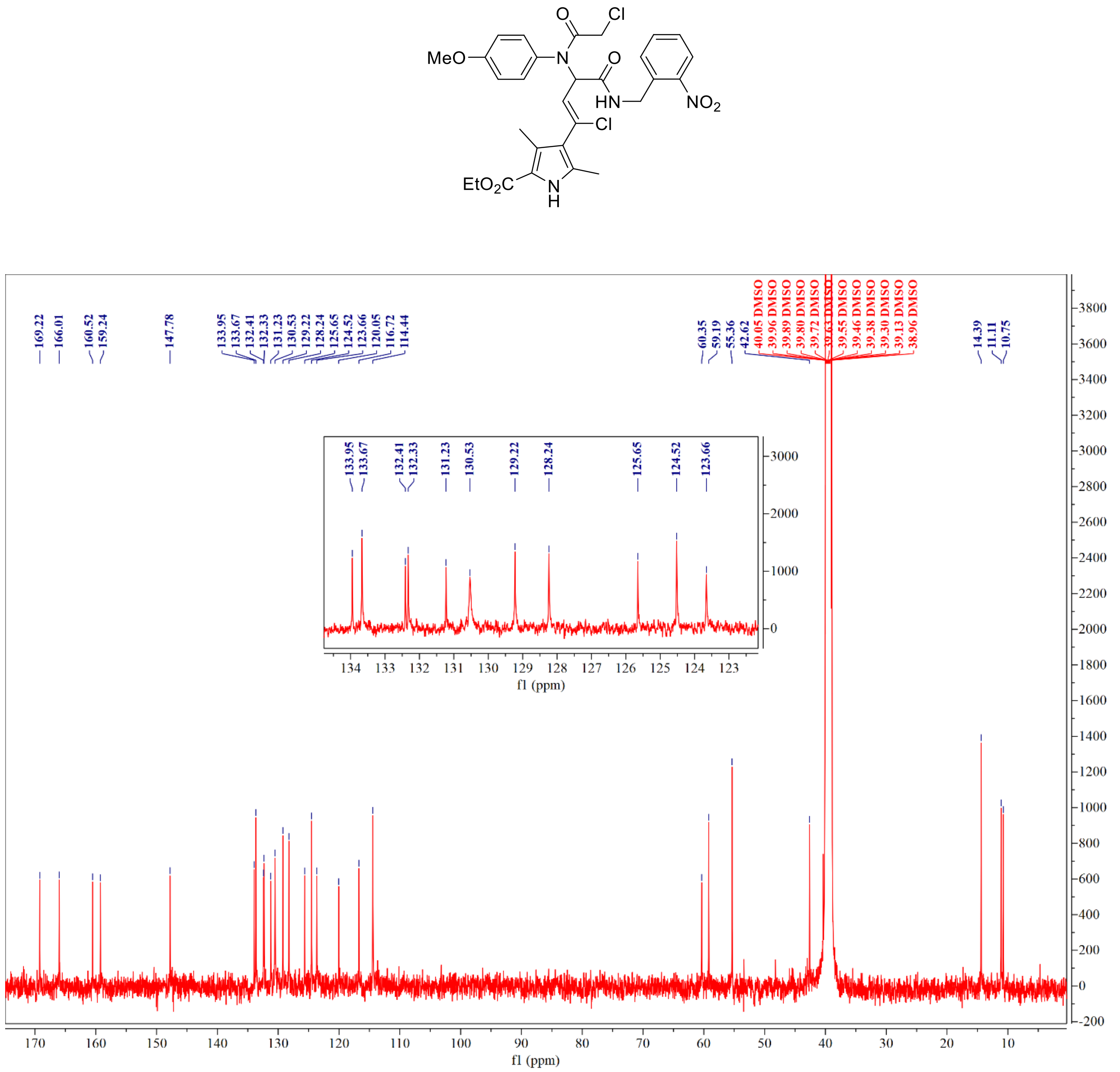

Figure S2. ^{13}C NMR spectrum of compound **5a**

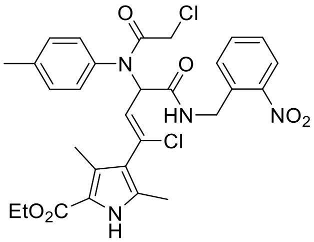

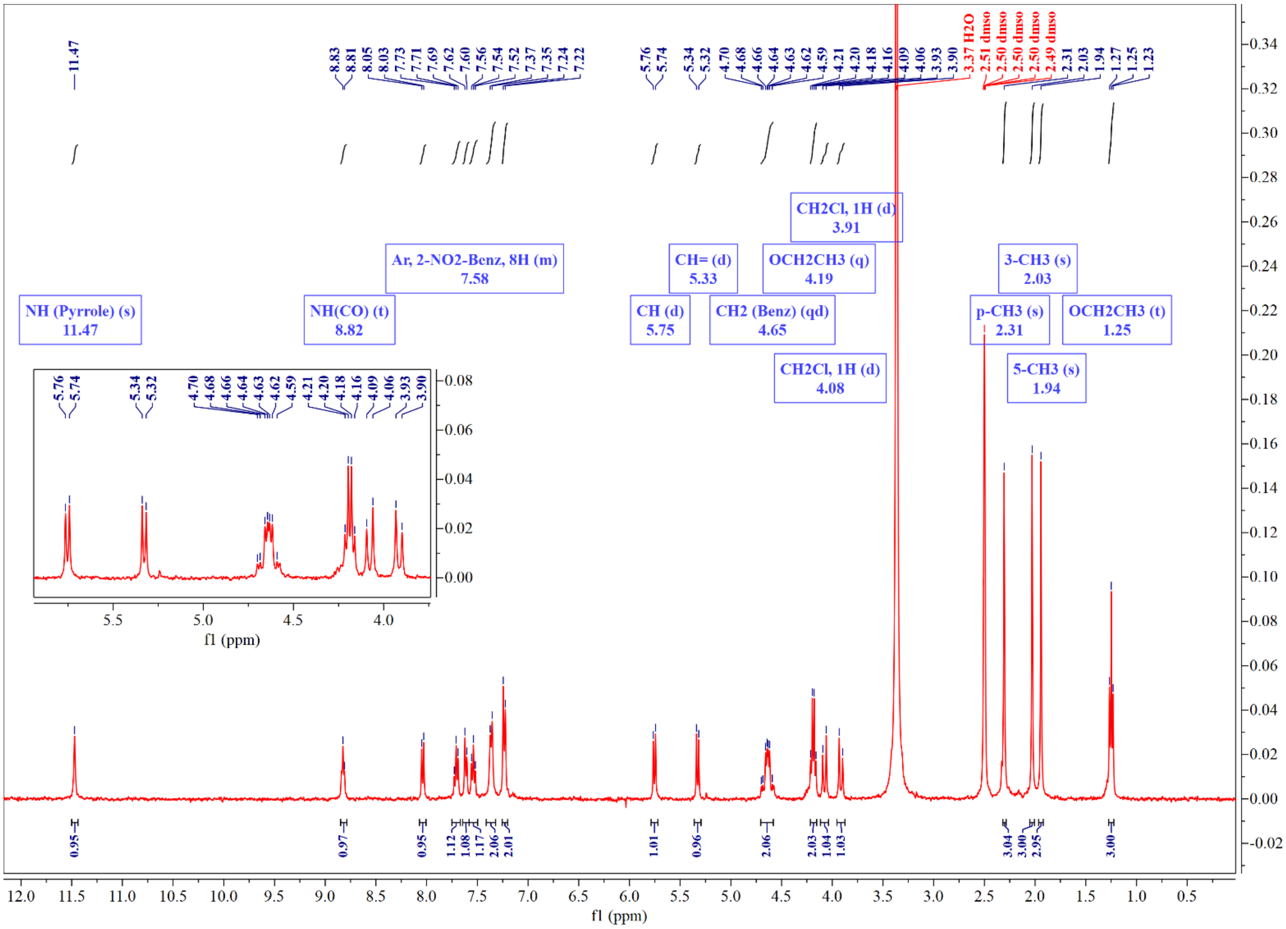

Figure S3. ^1H NMR spectrum of compound **5b** in DMSO-d_6.

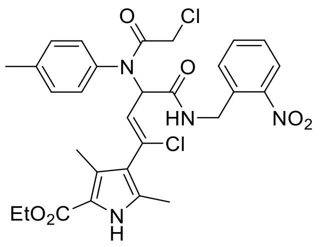

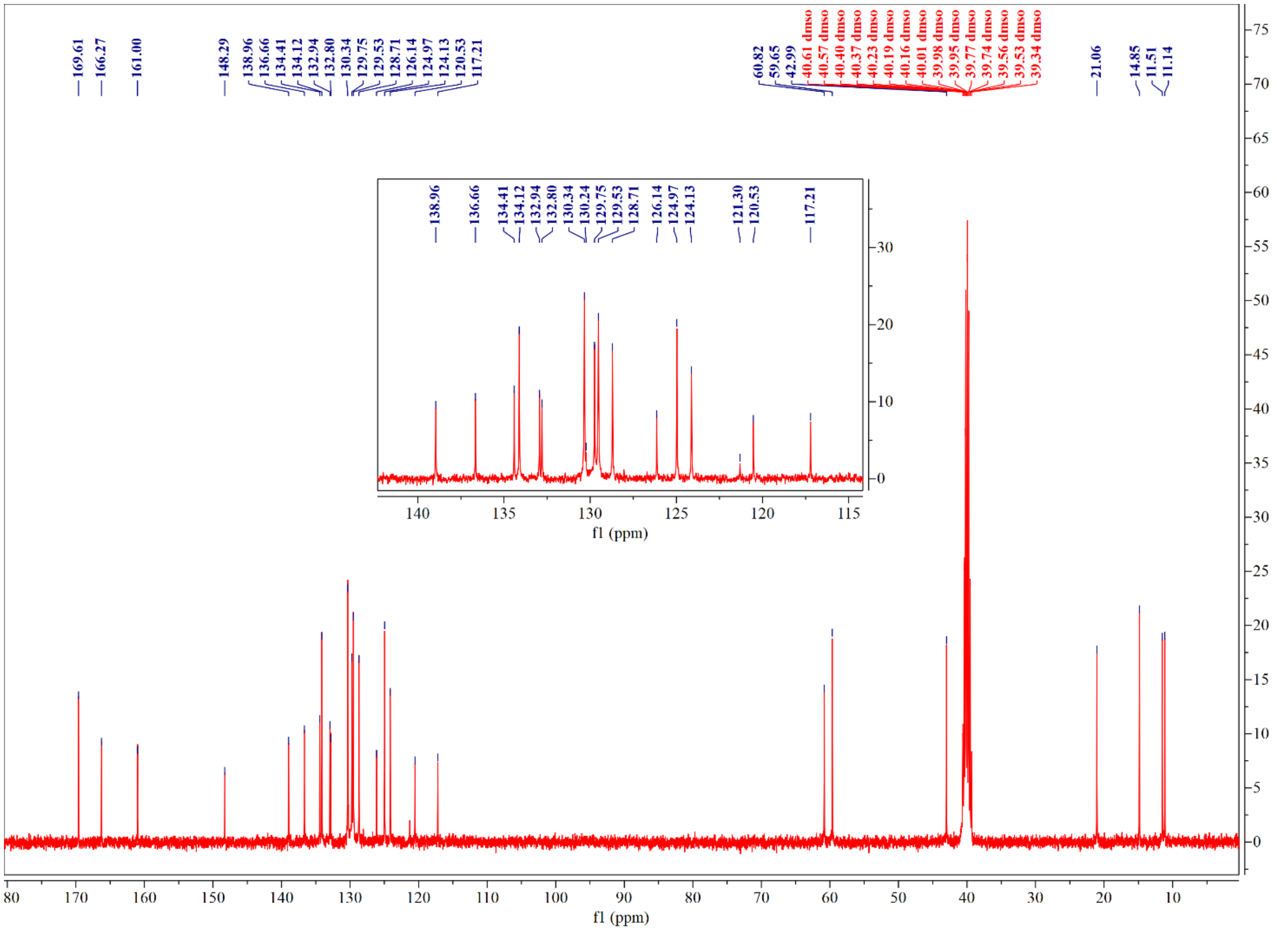

Figure S4. ^{13}C NMR spectrum of compound **5b**

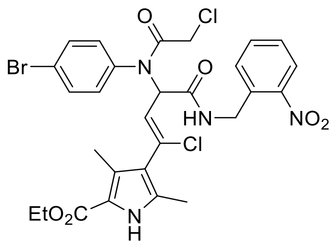

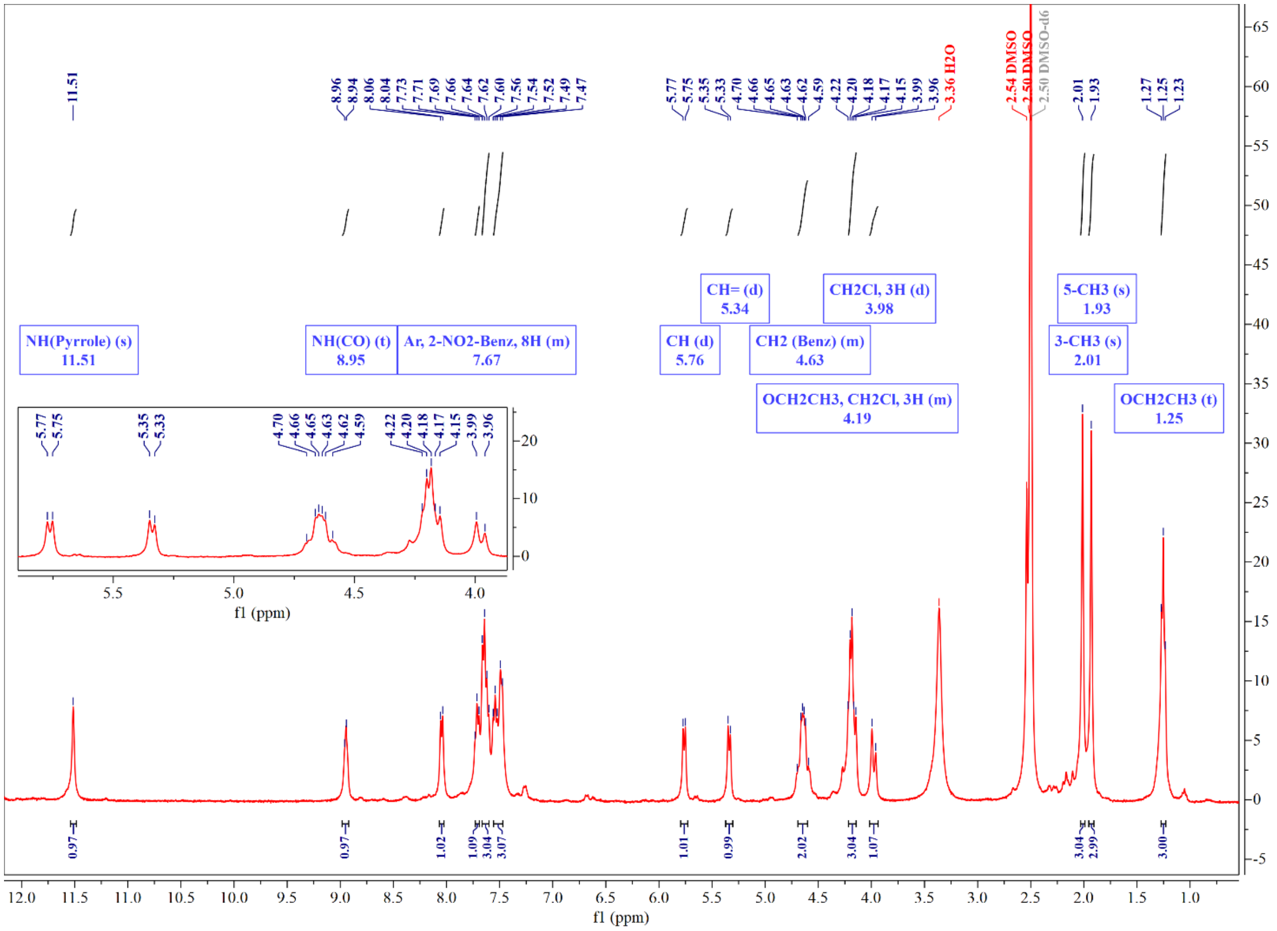

Figure S5. ^{1}H NMR spectrum of compound **5c** in DMSO-d_6.

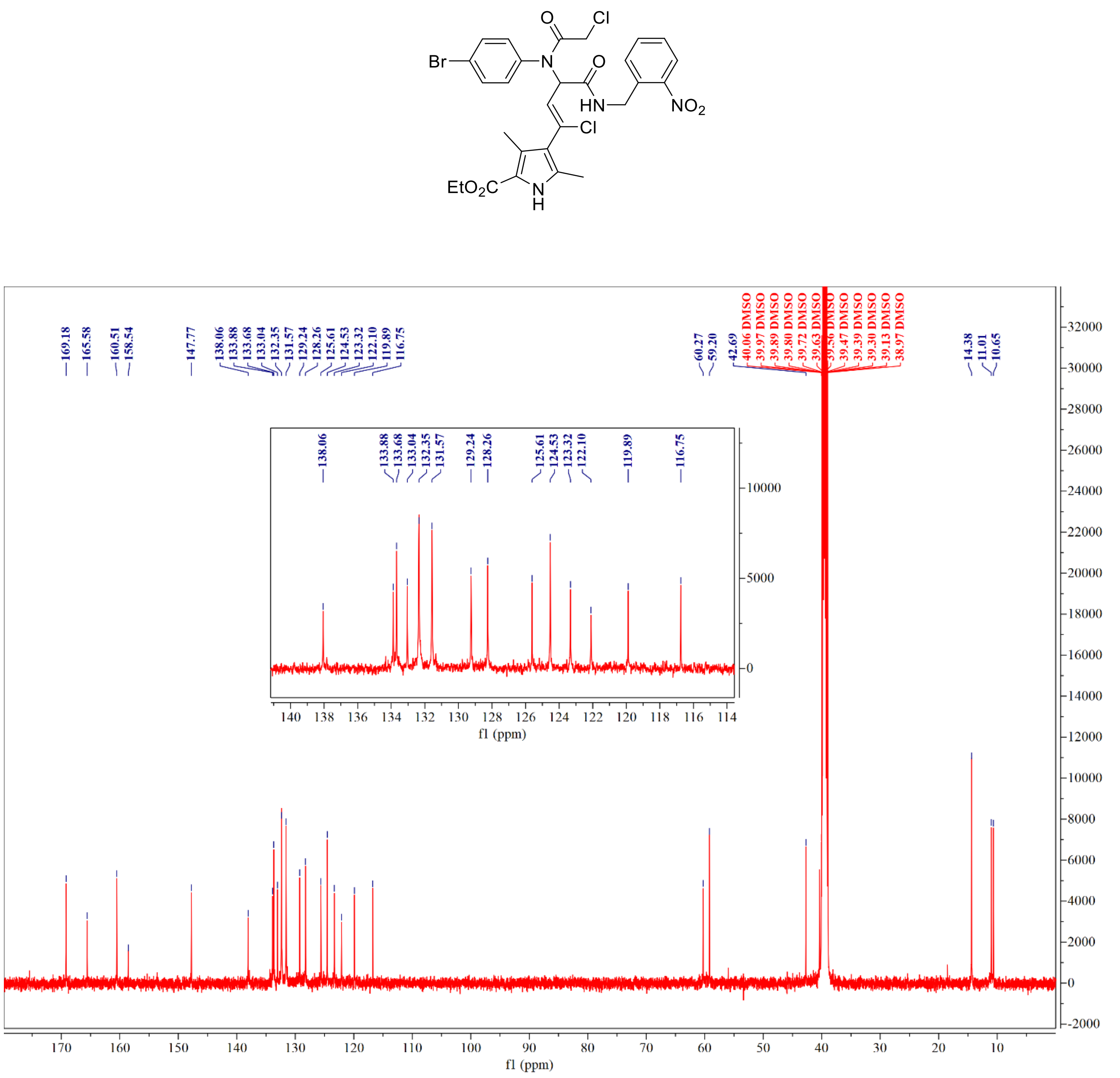

Figure S6. ^{13}C NMR spectrum of compound **5c**

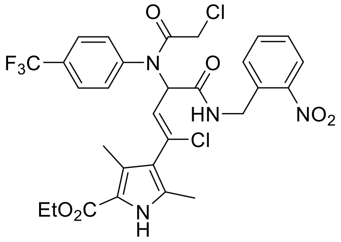

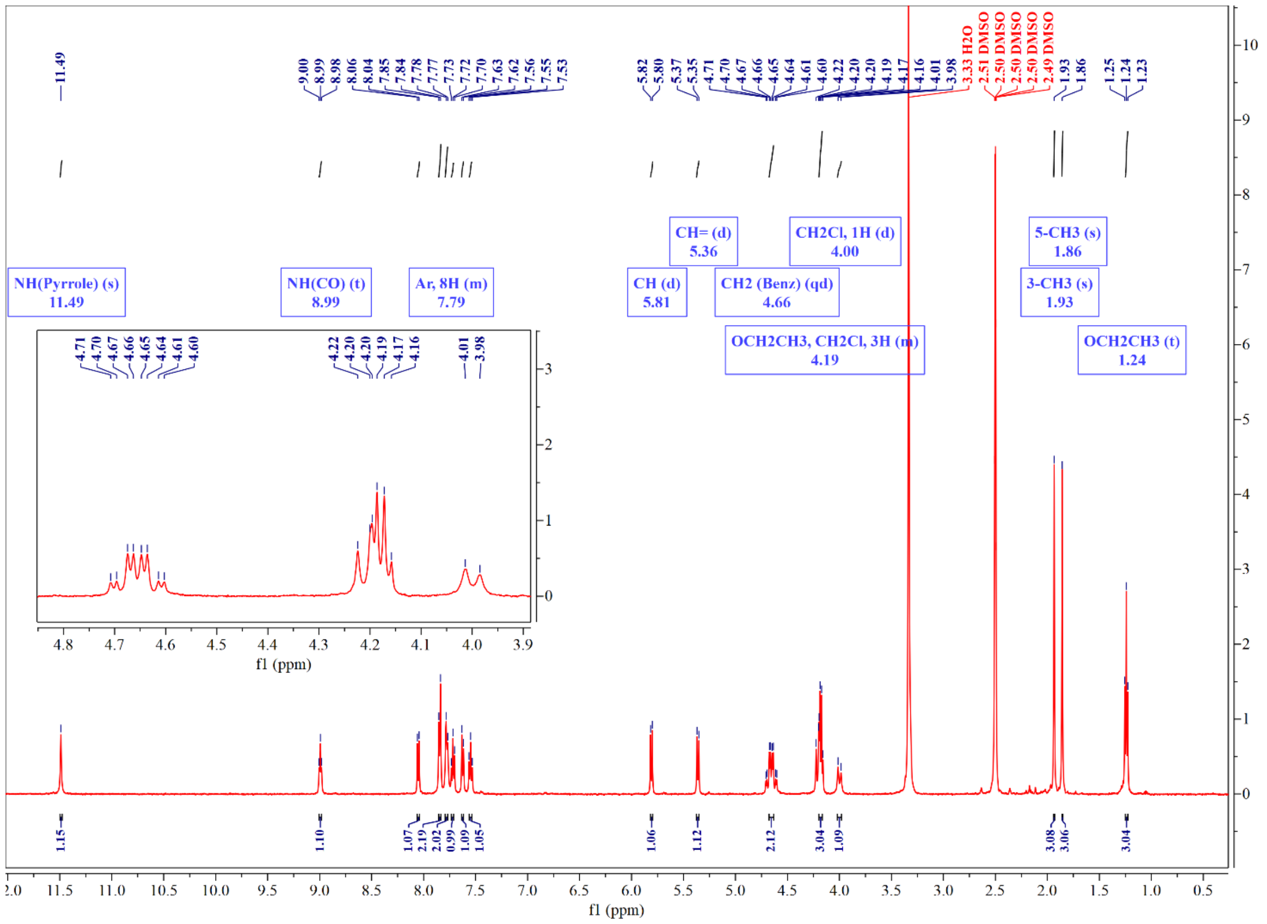

Figure S7. ^{1}H NMR spectrum of compound **5d** in DMSO-d_6.

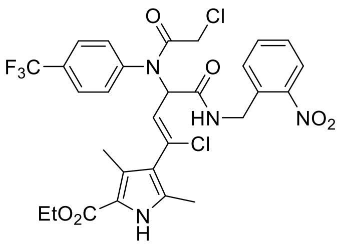

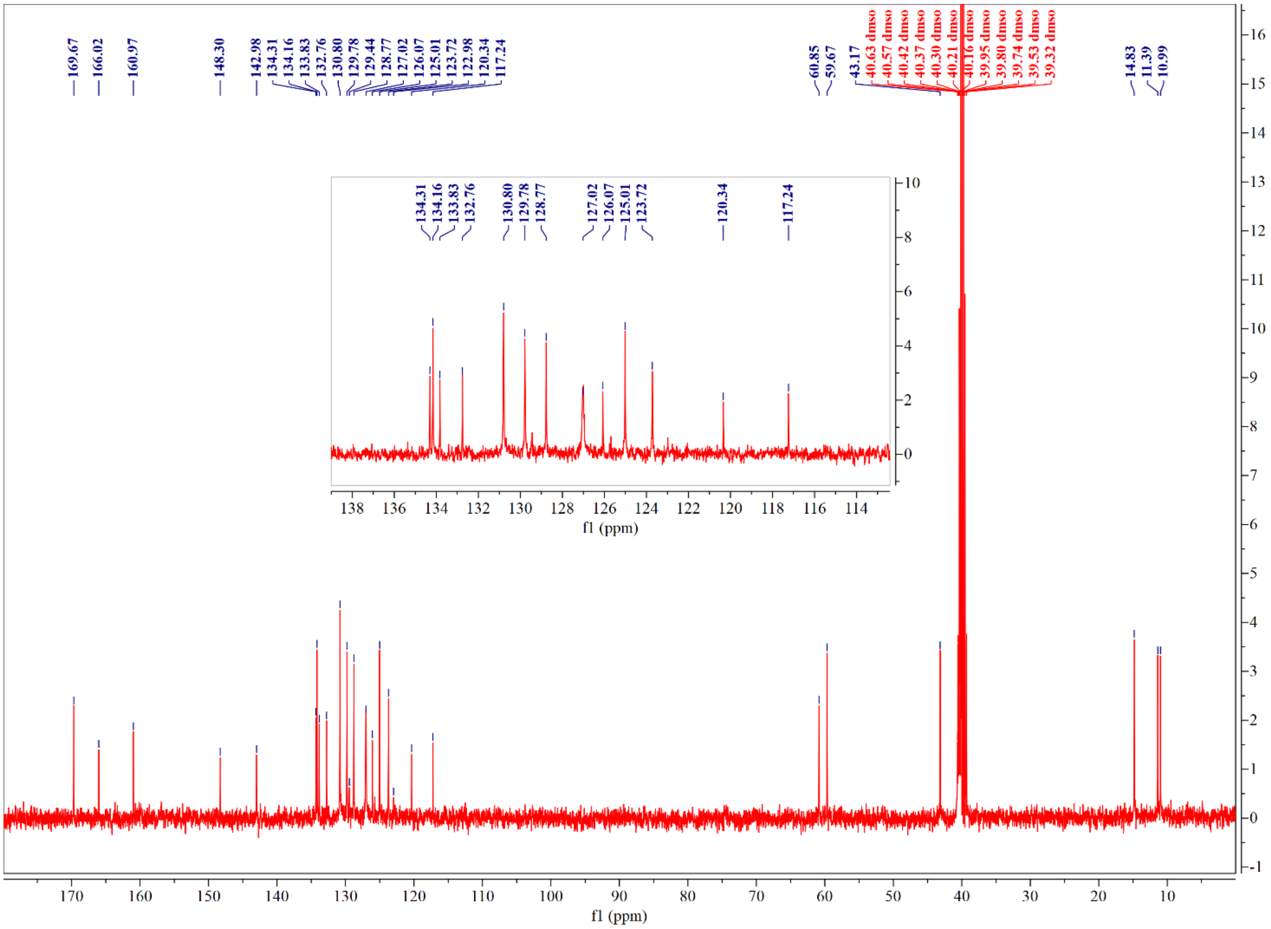

Figure S8. ^{13}C NMR spectrum of compound **5d**

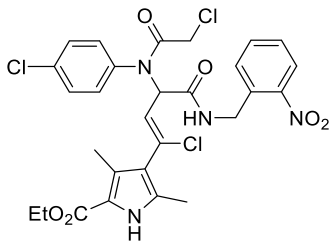

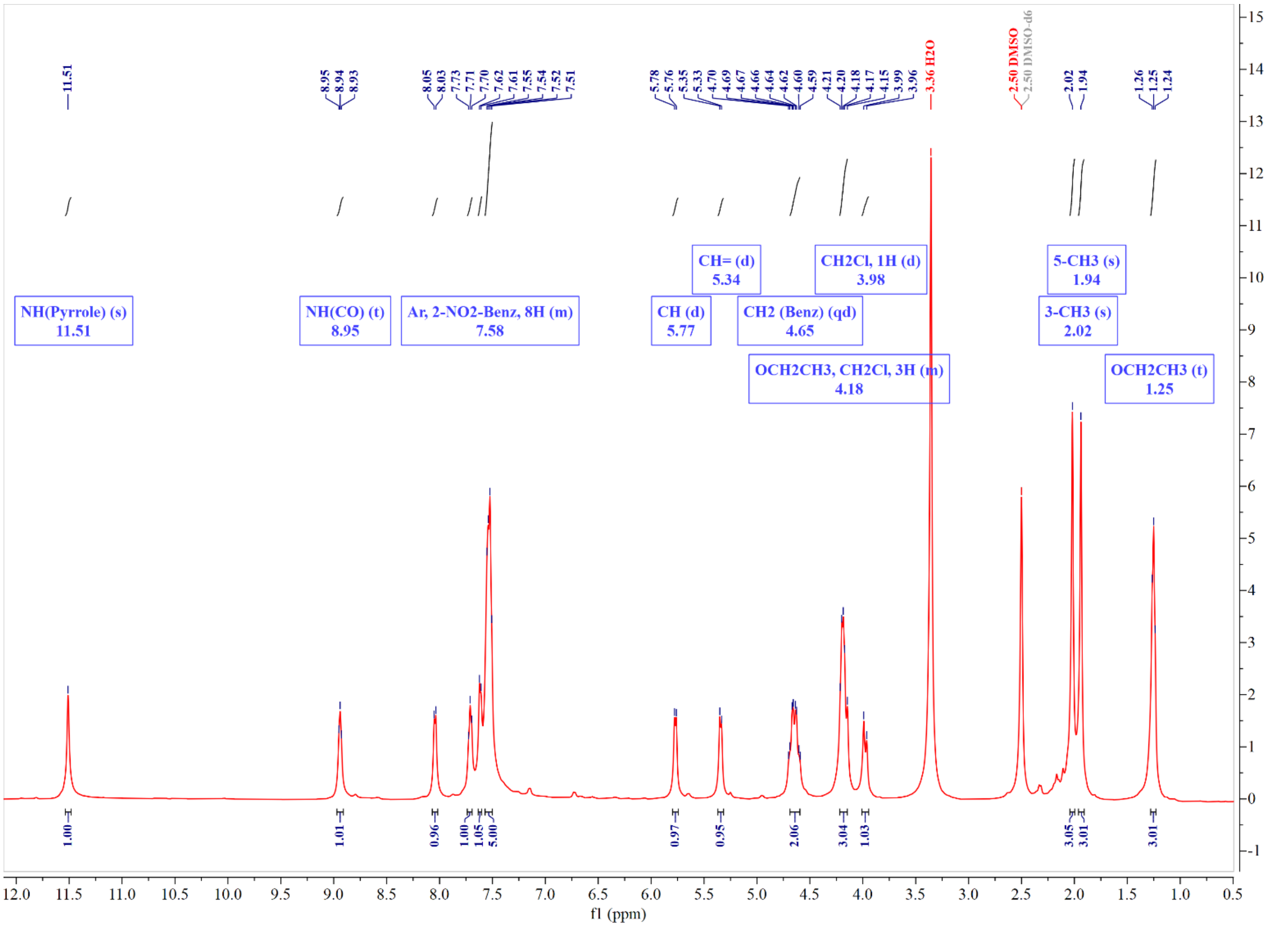

Figure S9. ^{1}H NMR spectrum of compound **5e** in DMSO-d_6.

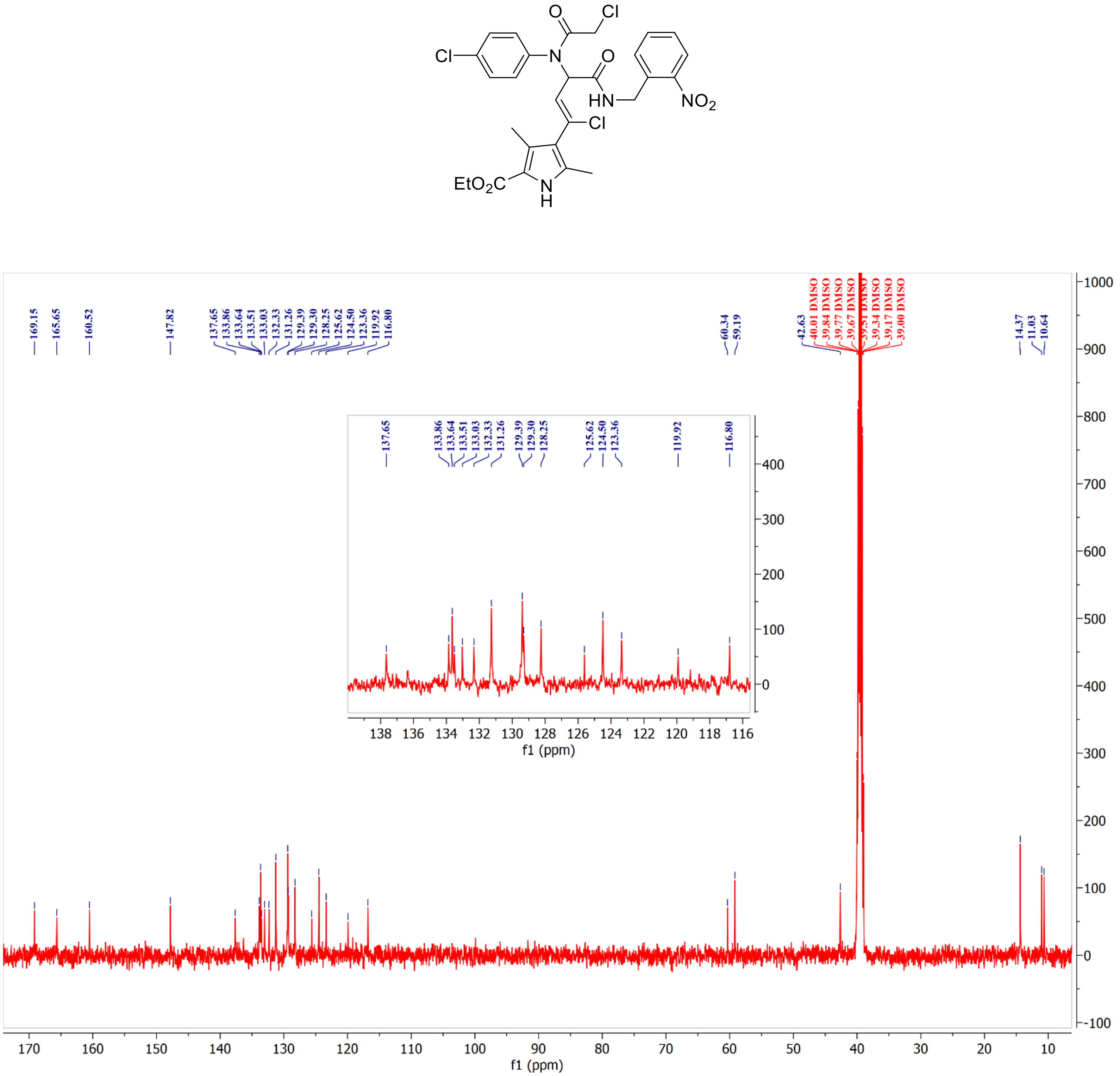

Figure S10. ^{13}C NMR spectrum of compound **5e**

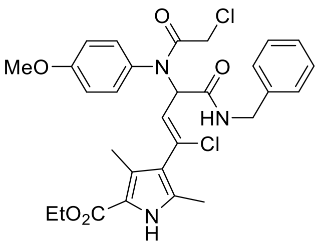

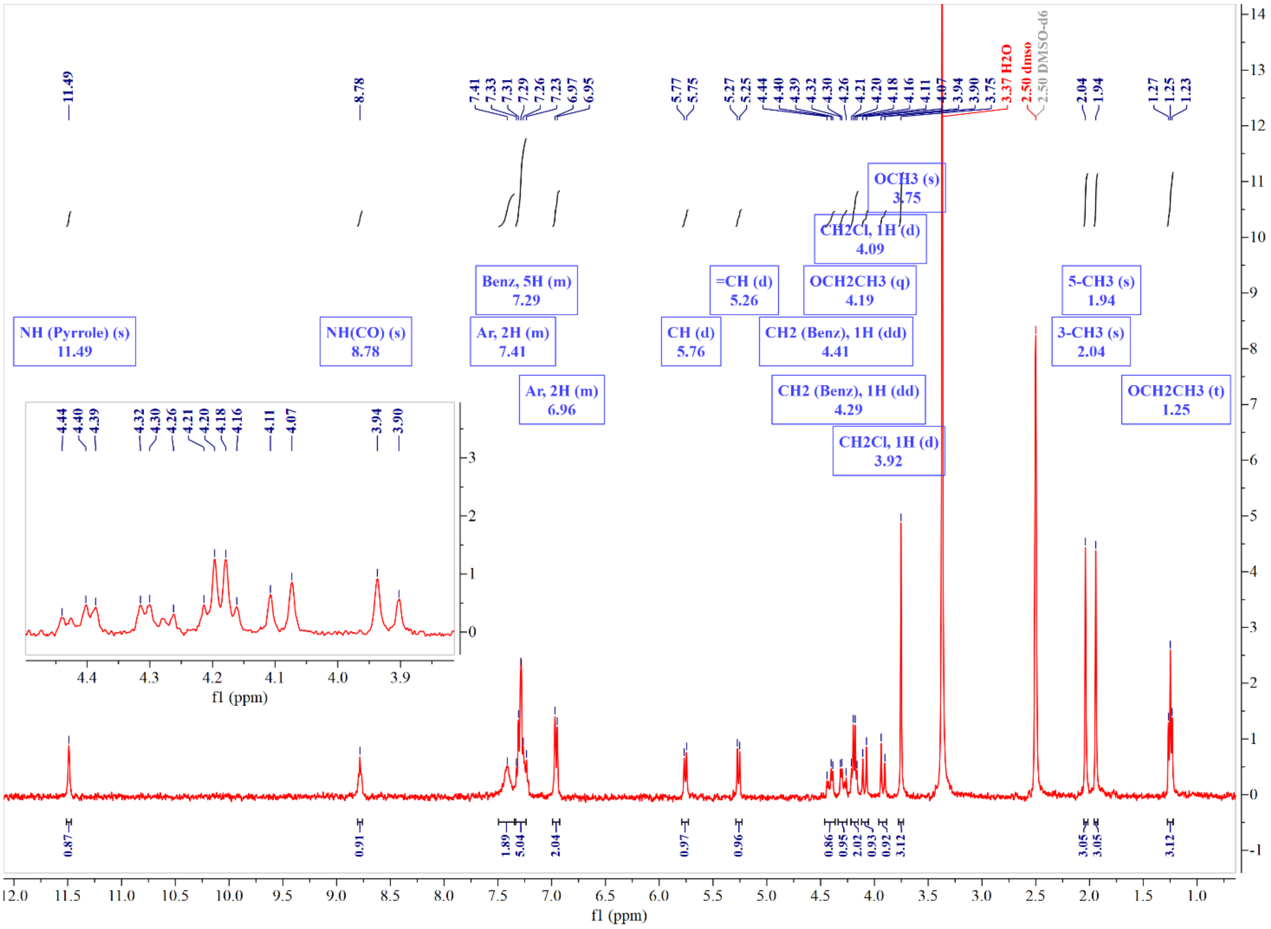

Figure S11. ^{1}H NMR spectrum of compound **6a** in DMSO-d_6.

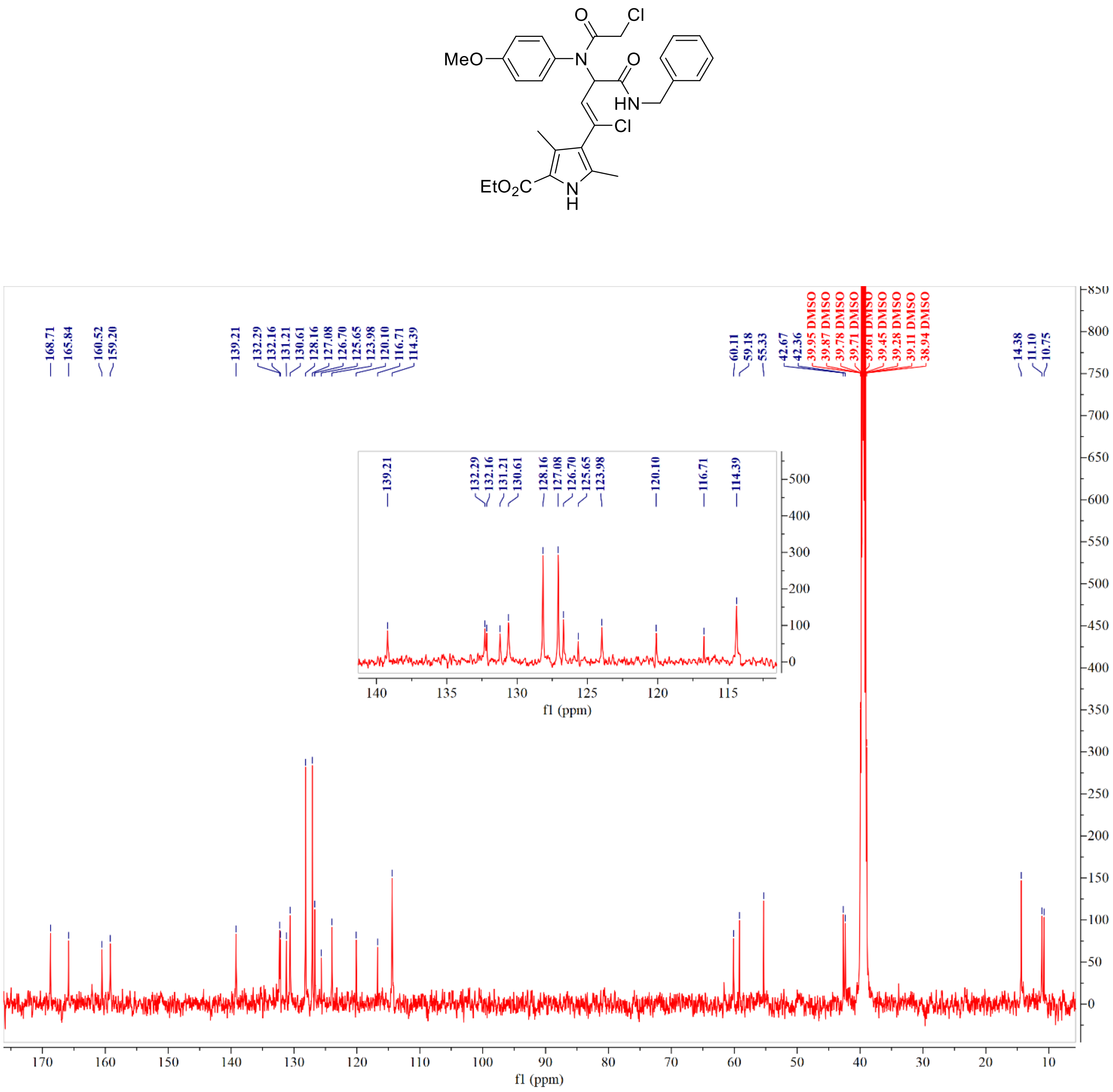

Figure S12. ^{13}C NMR spectrum of compound **6a**

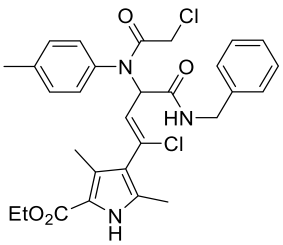

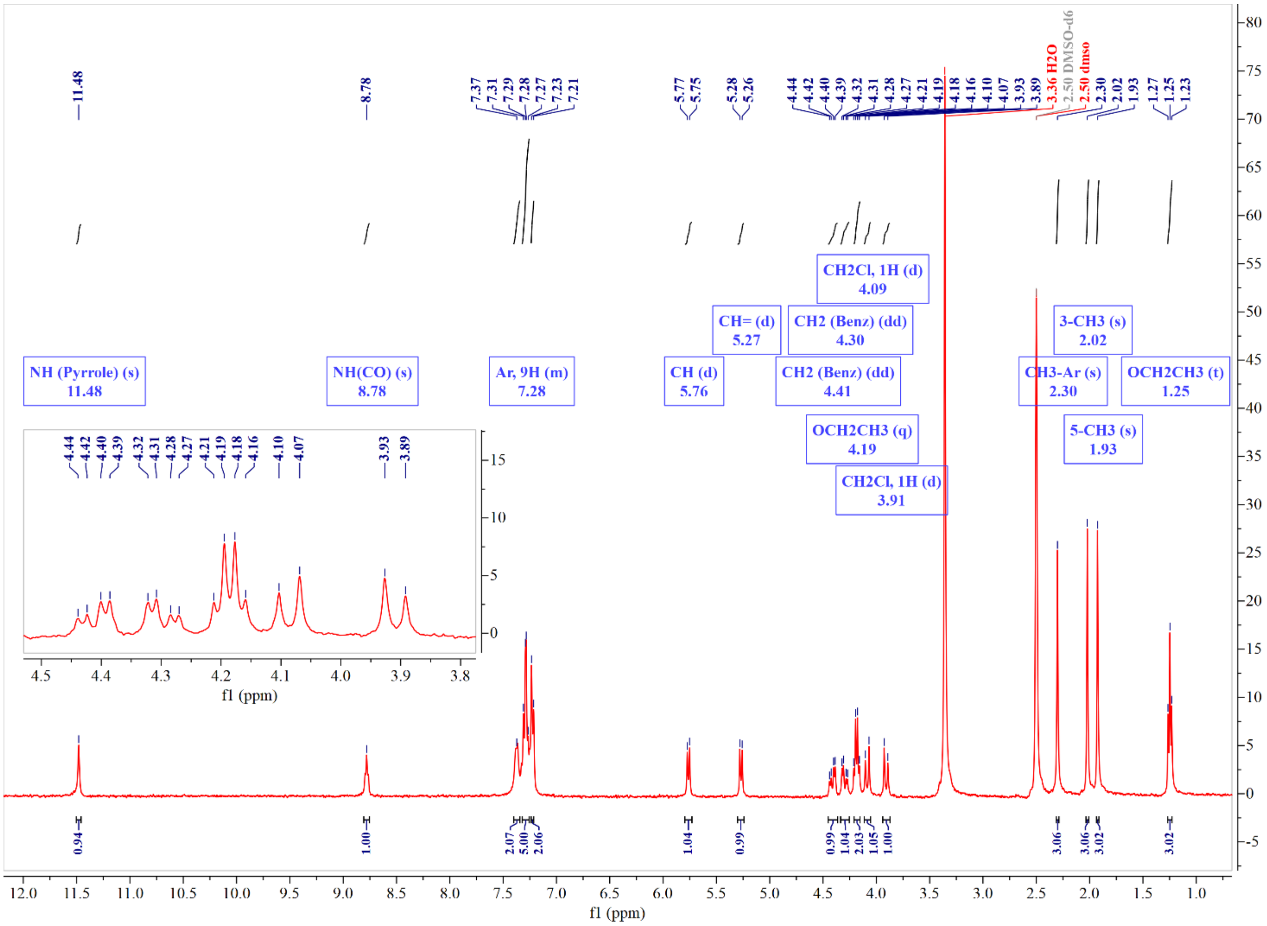

Figure S13. ^{1}H NMR spectrum of compound **6b** in DMSO-d_6.

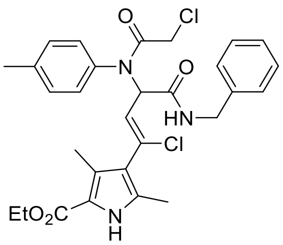

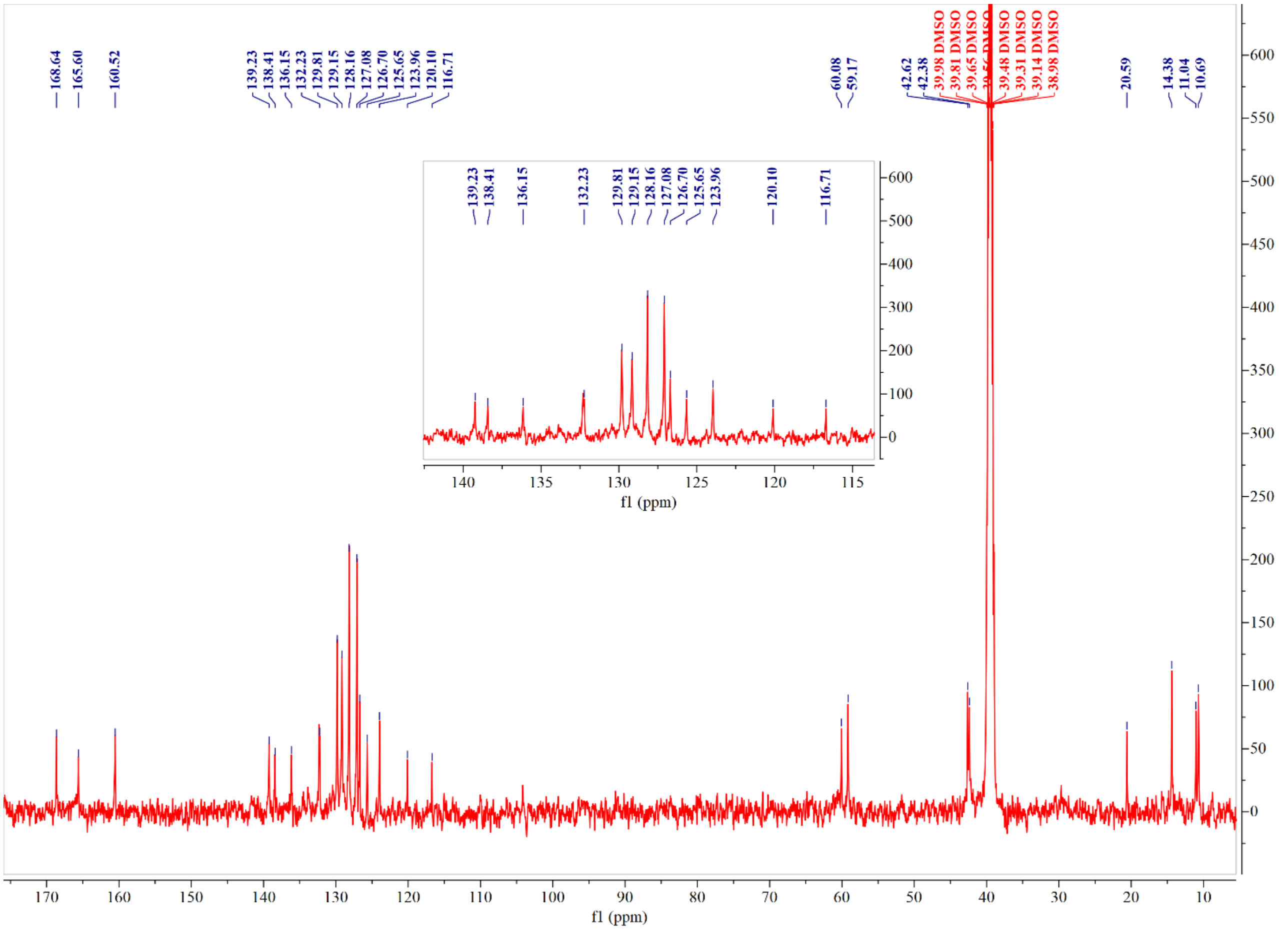

Figure S14. ^{13}C NMR spectrum of compound **6b**

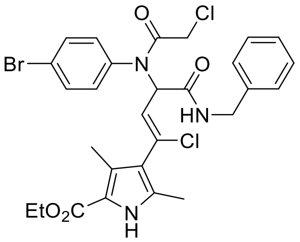

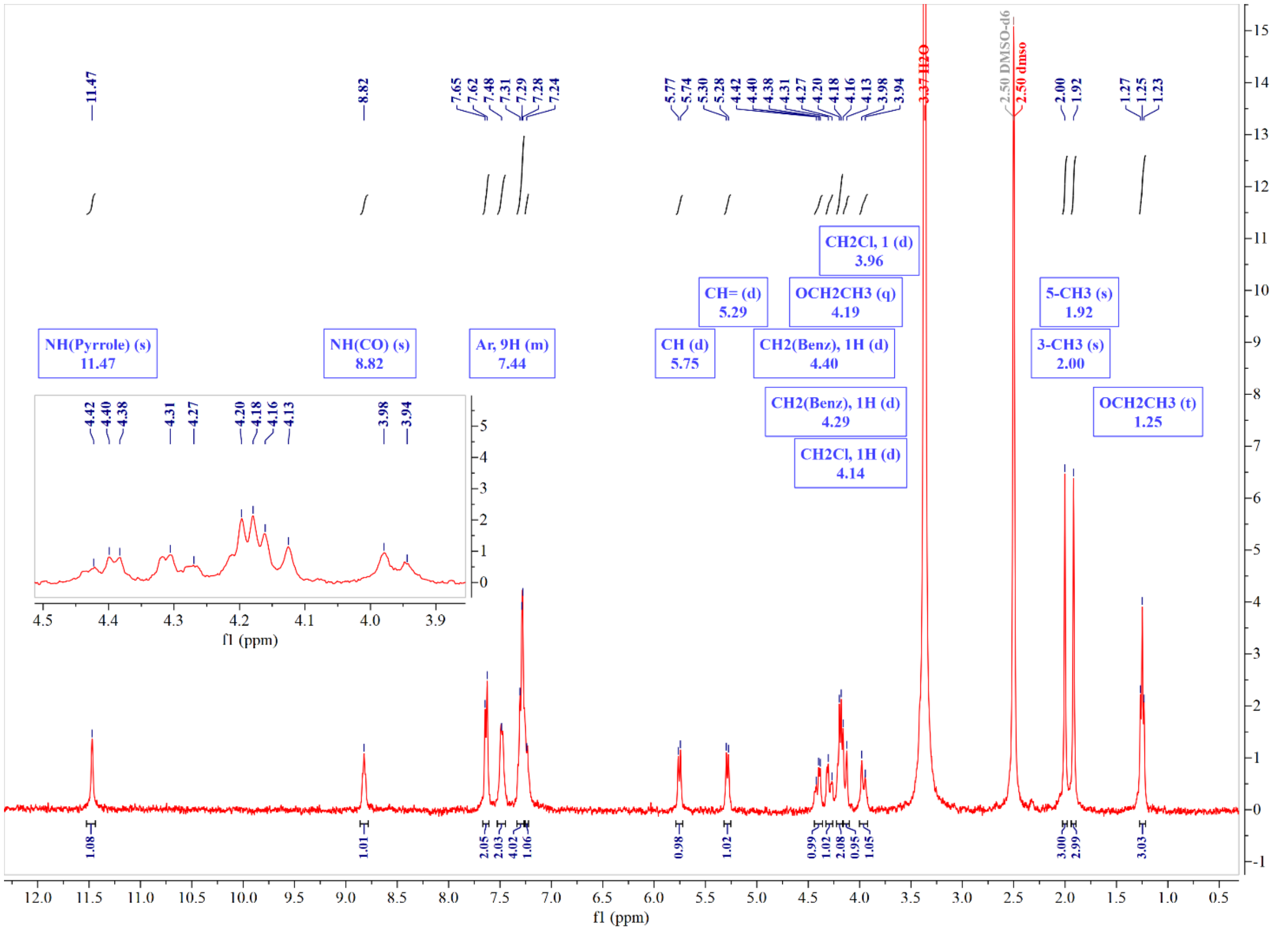

Figure S15. ^{1}H NMR spectrum of compound **6c** in DMSO-*d*$_6$.

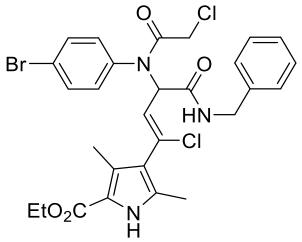

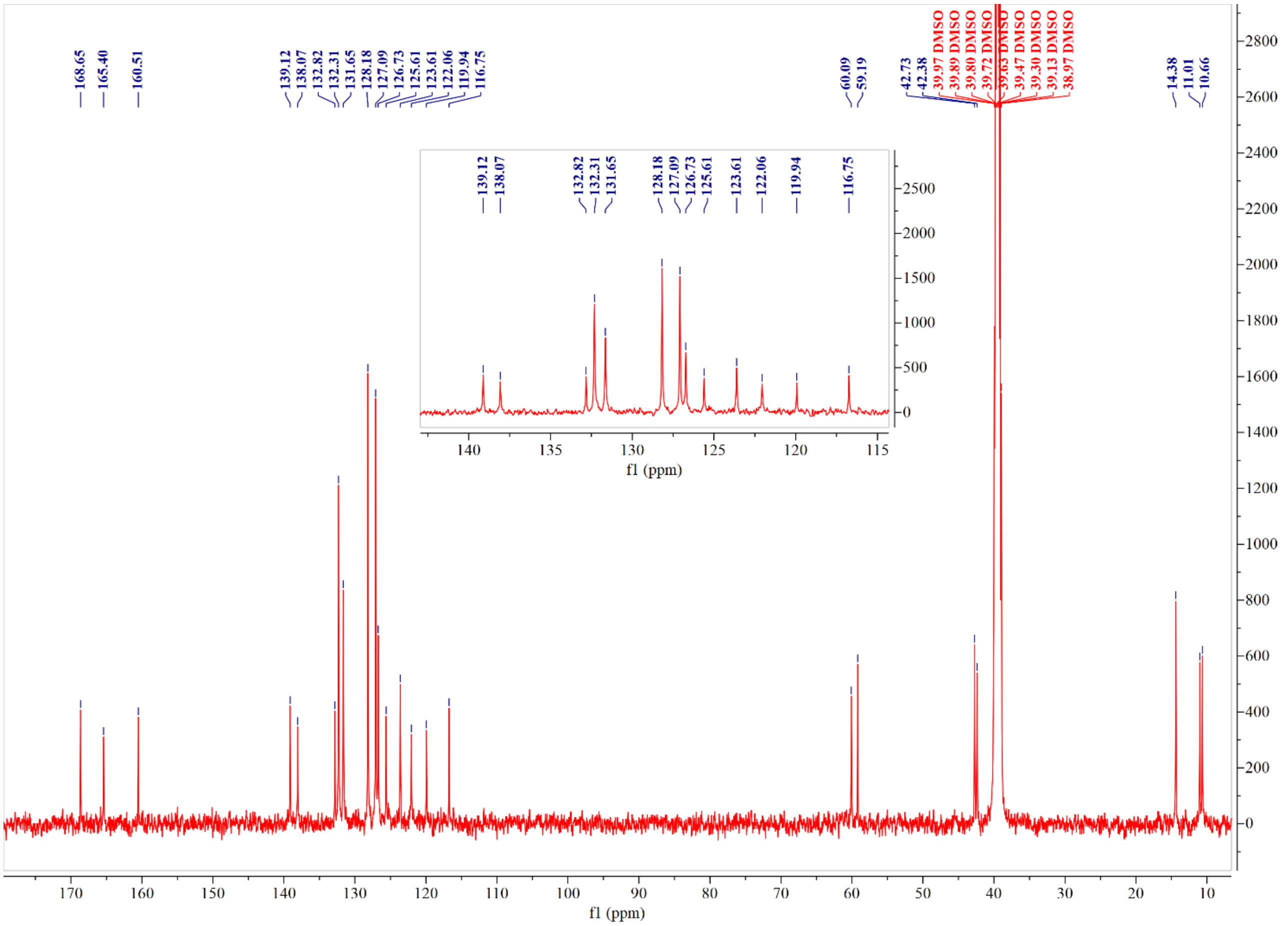

Figure S16. ^{13}C NMR spectrum of compound **6c**

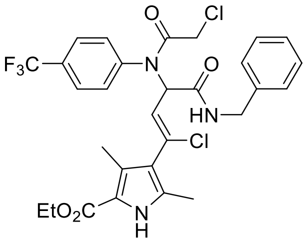

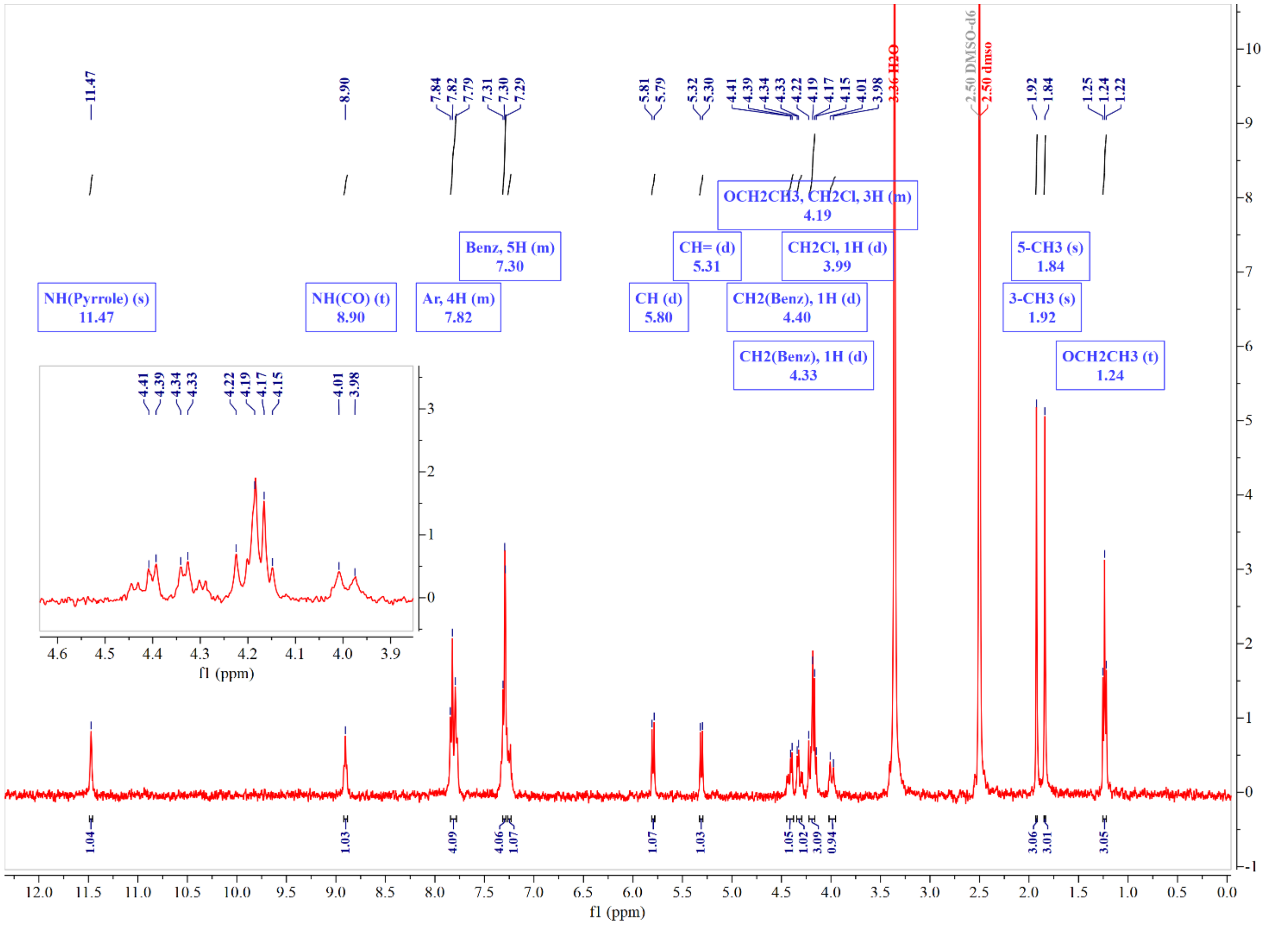

Figure S17. ^{1}H NMR spectrum of compound **6d** in DMSO-d_6.

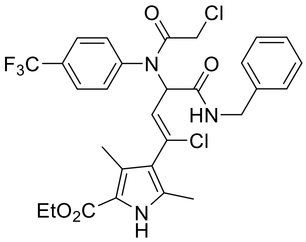

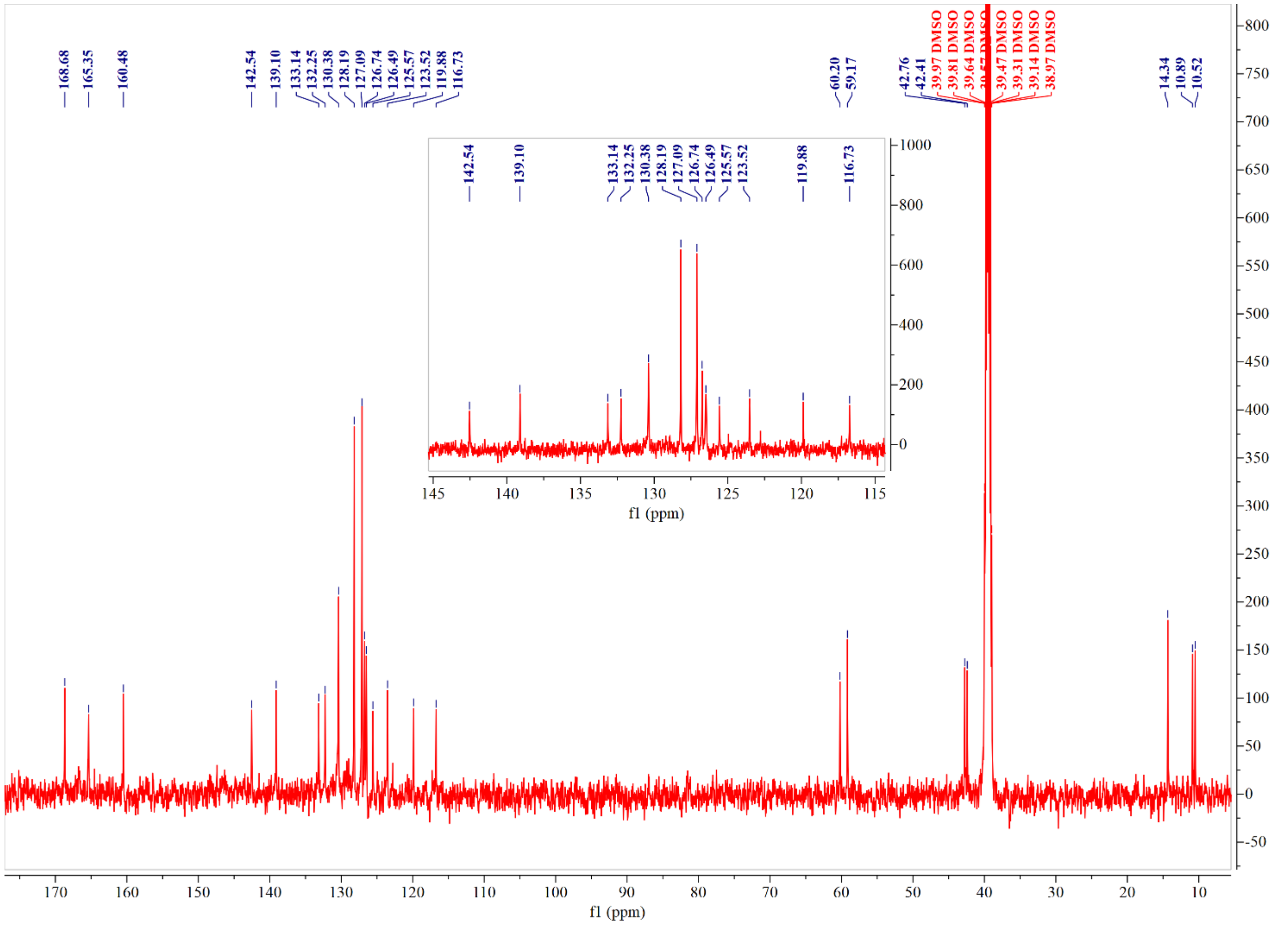

Figure S18. ^{13}C NMR spectrum of compound **6d**

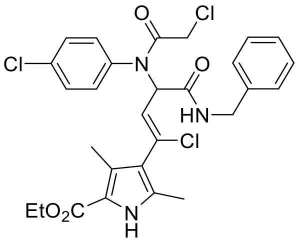

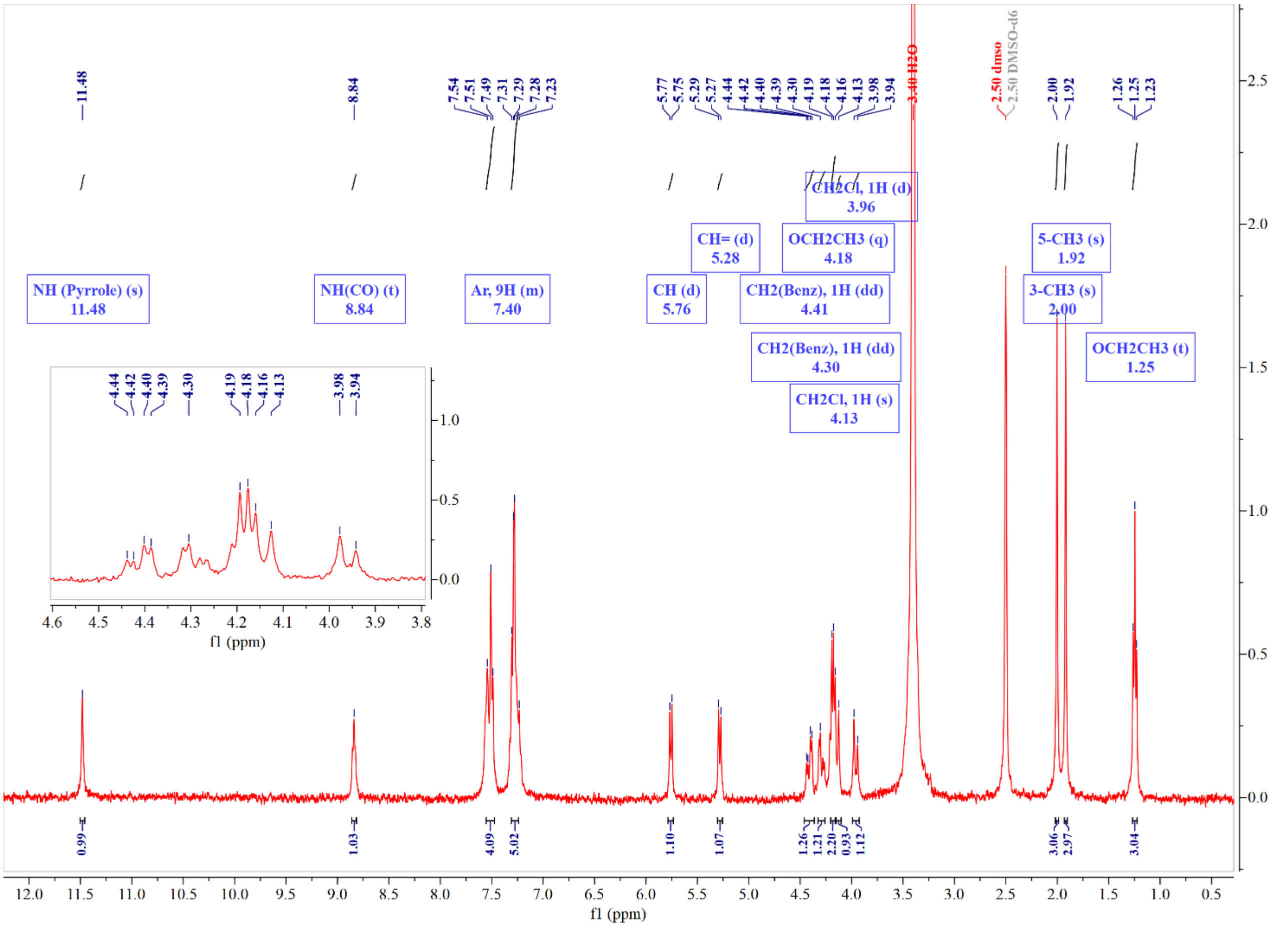

Figure S19. ^{1}H NMR spectrum of compound **6e** in DMSO-d_6.

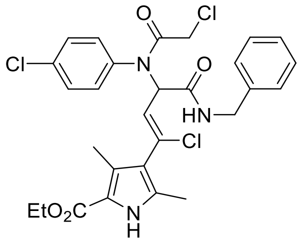

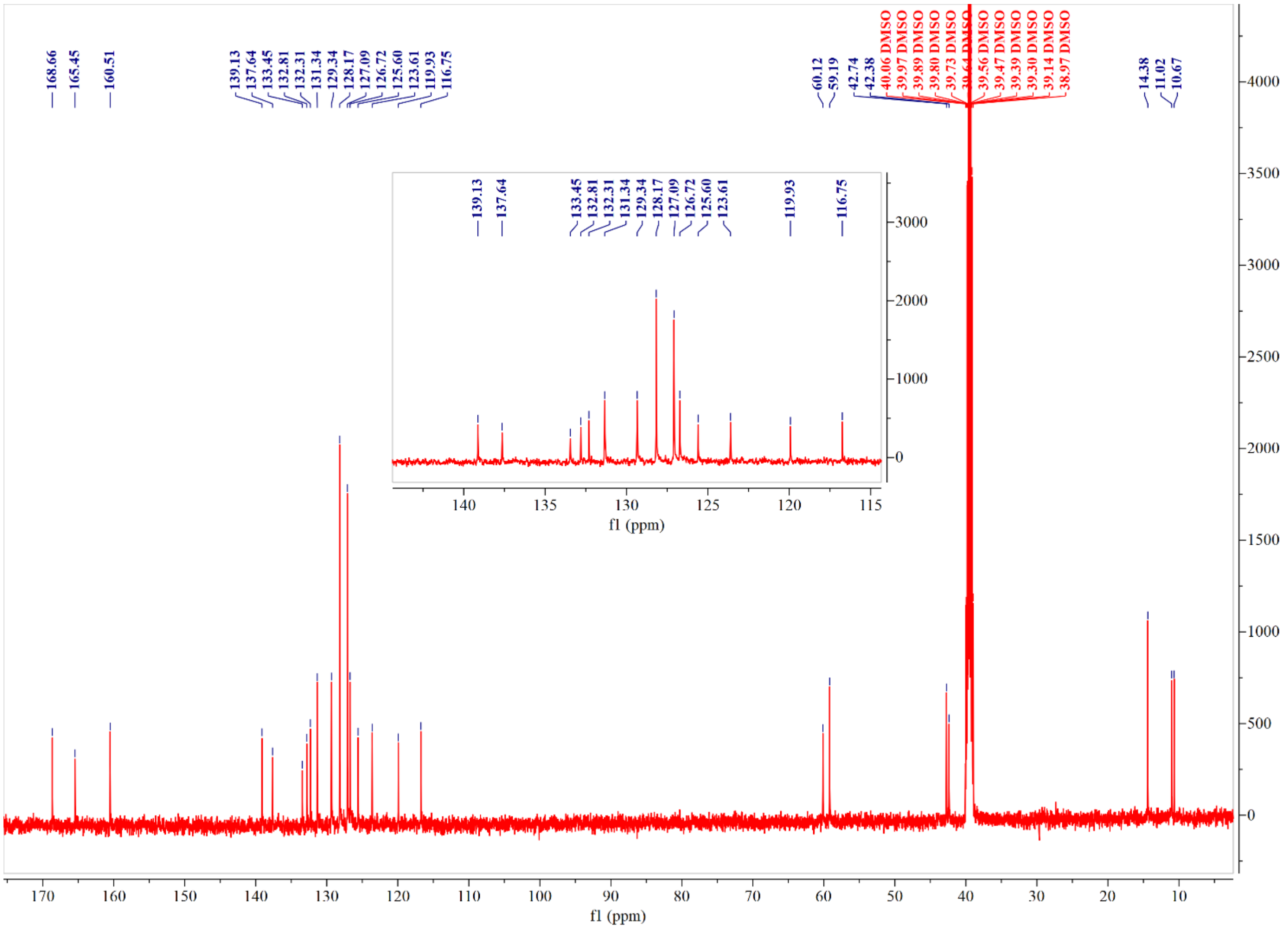

Figure S20. ^{13}C NMR spectrum of compound **6e**

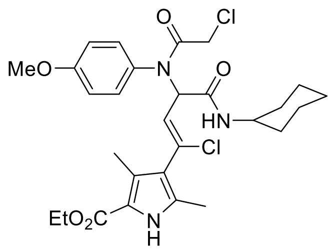

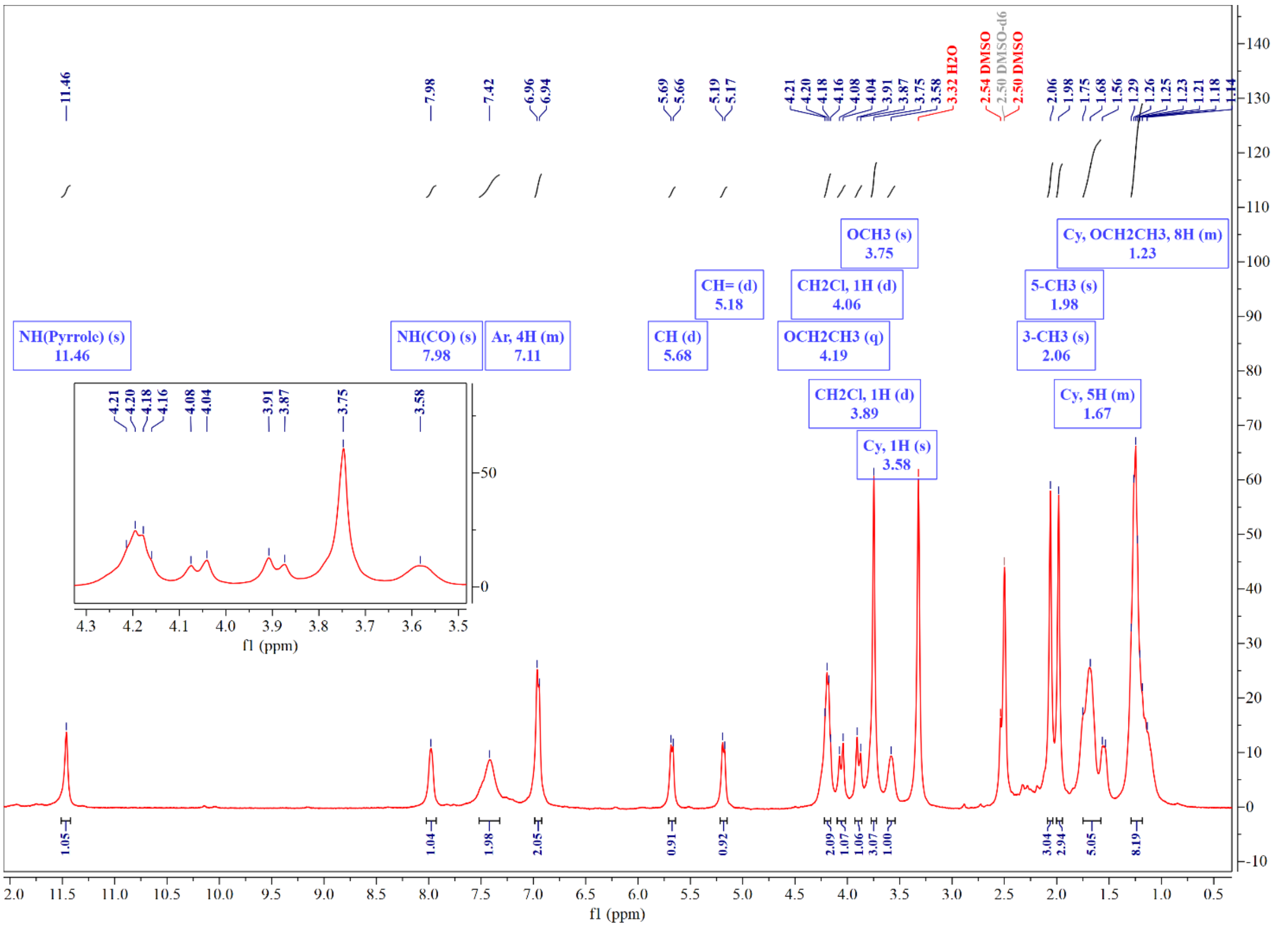

Figure S21. ¹H NMR spectrum of compound **7a** in DMSO-d_6.

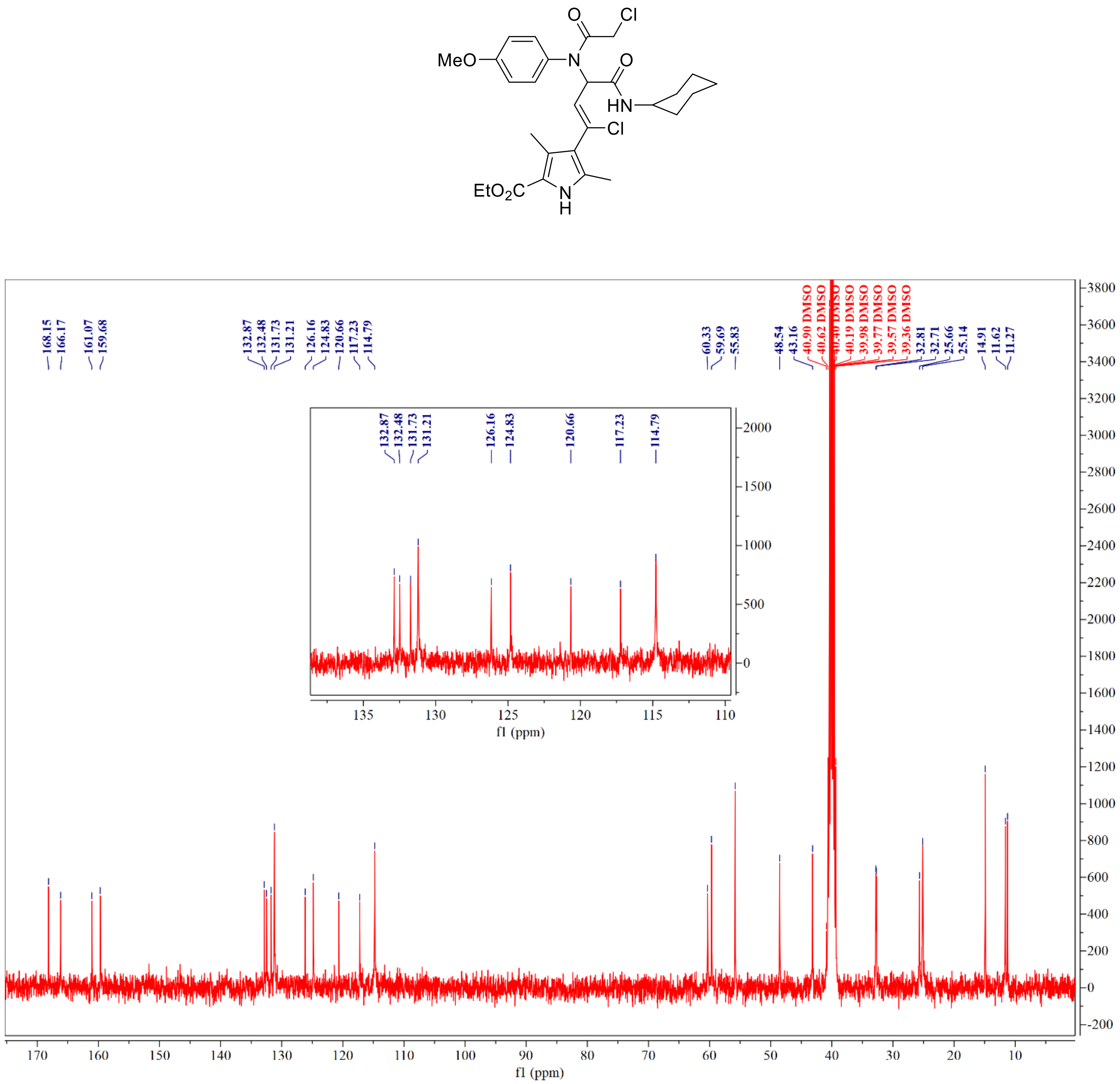

Figure S22. ^{13}C NMR spectrum of compound **7a**

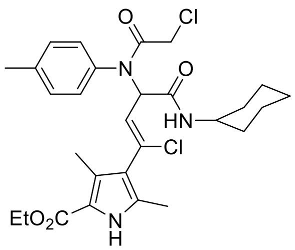

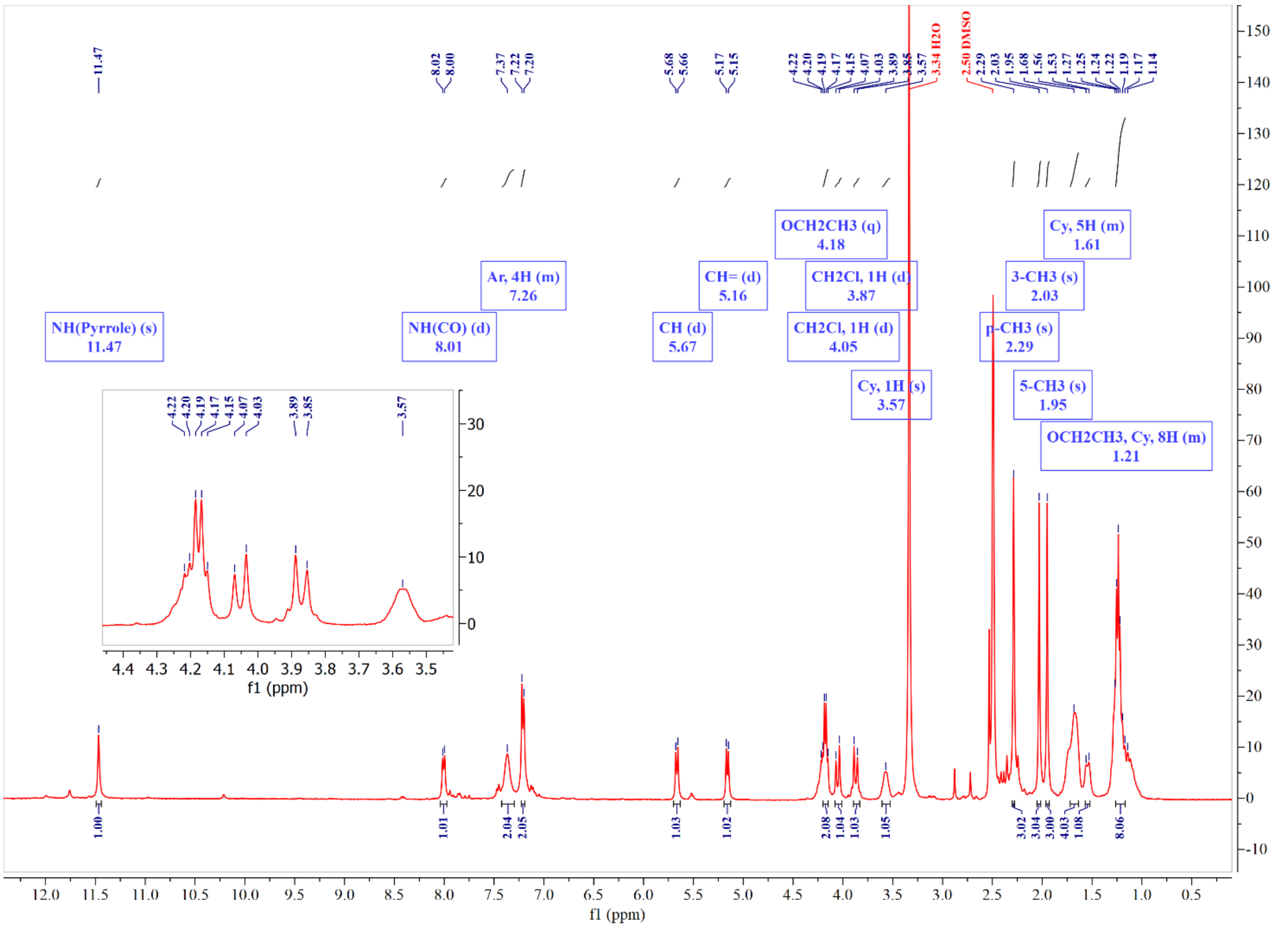

Figure S23. ^{1}H NMR spectrum of compound **7b** in DMSO-d_6.

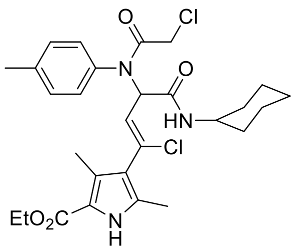

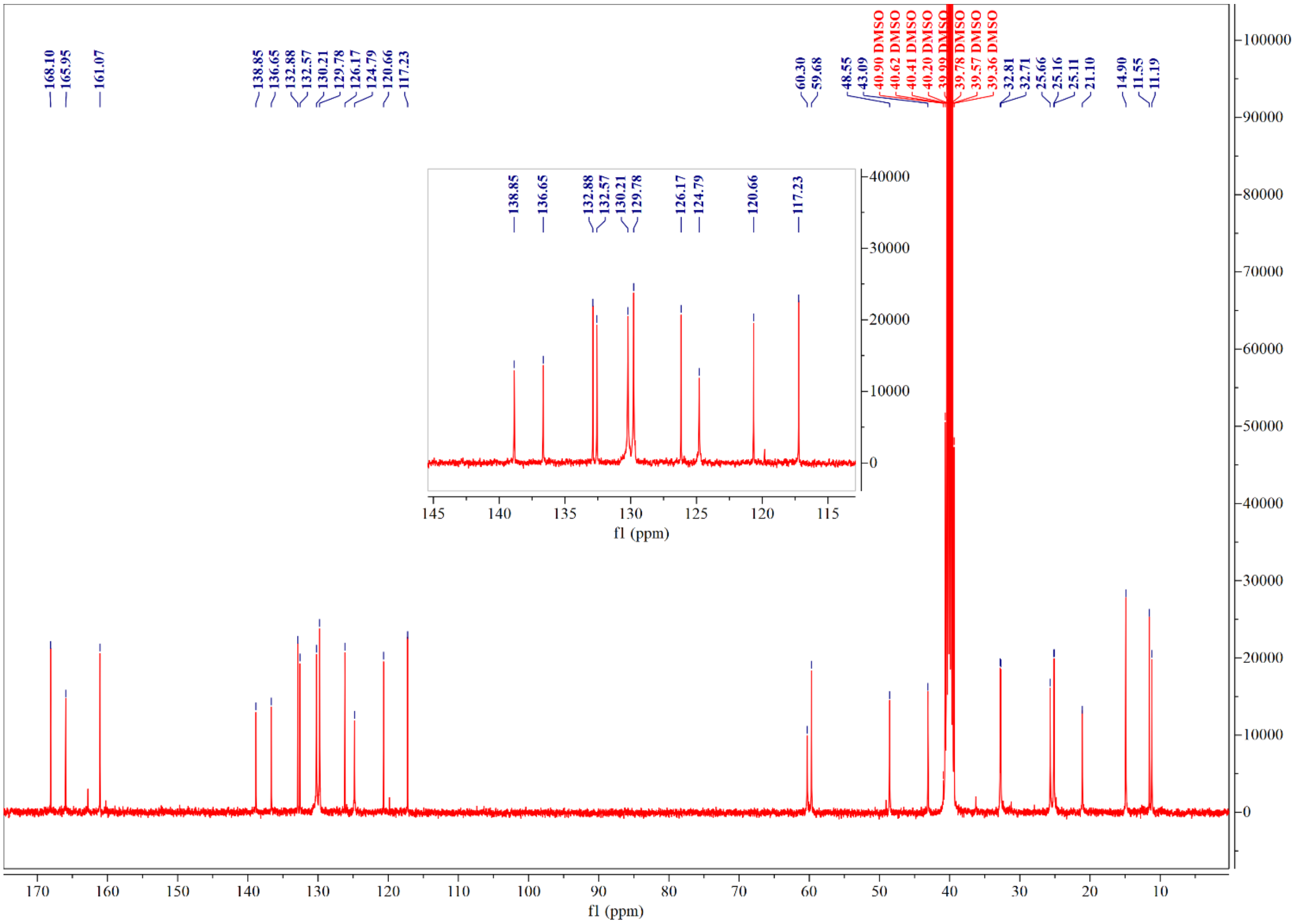

Figure S24. ^{13}C NMR spectrum of compound **7b**

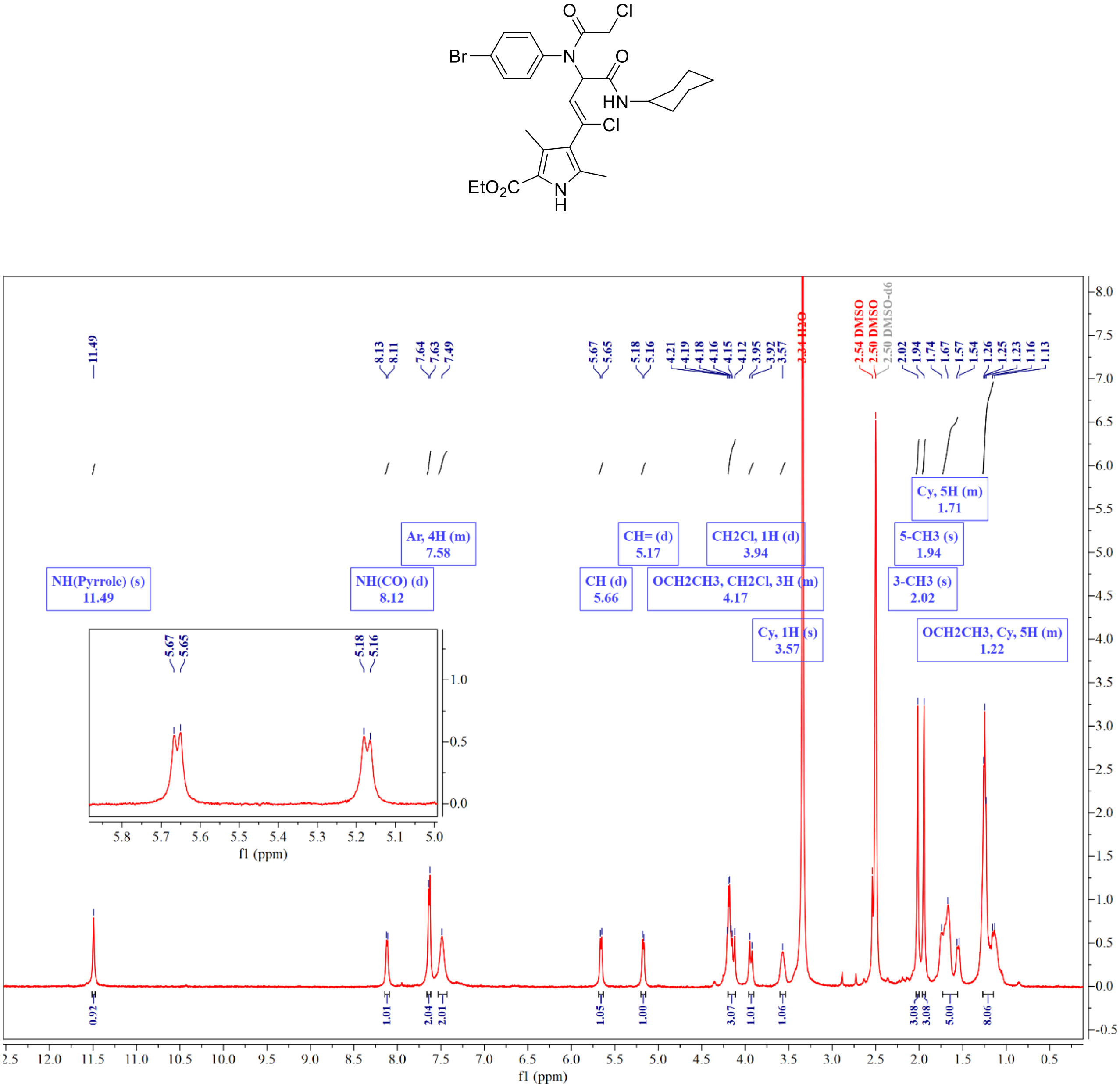

Figure S25. ^{1}H NMR spectrum of compound **7c** in DMSO-d_6.

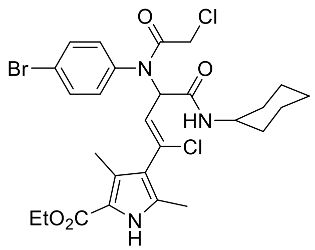

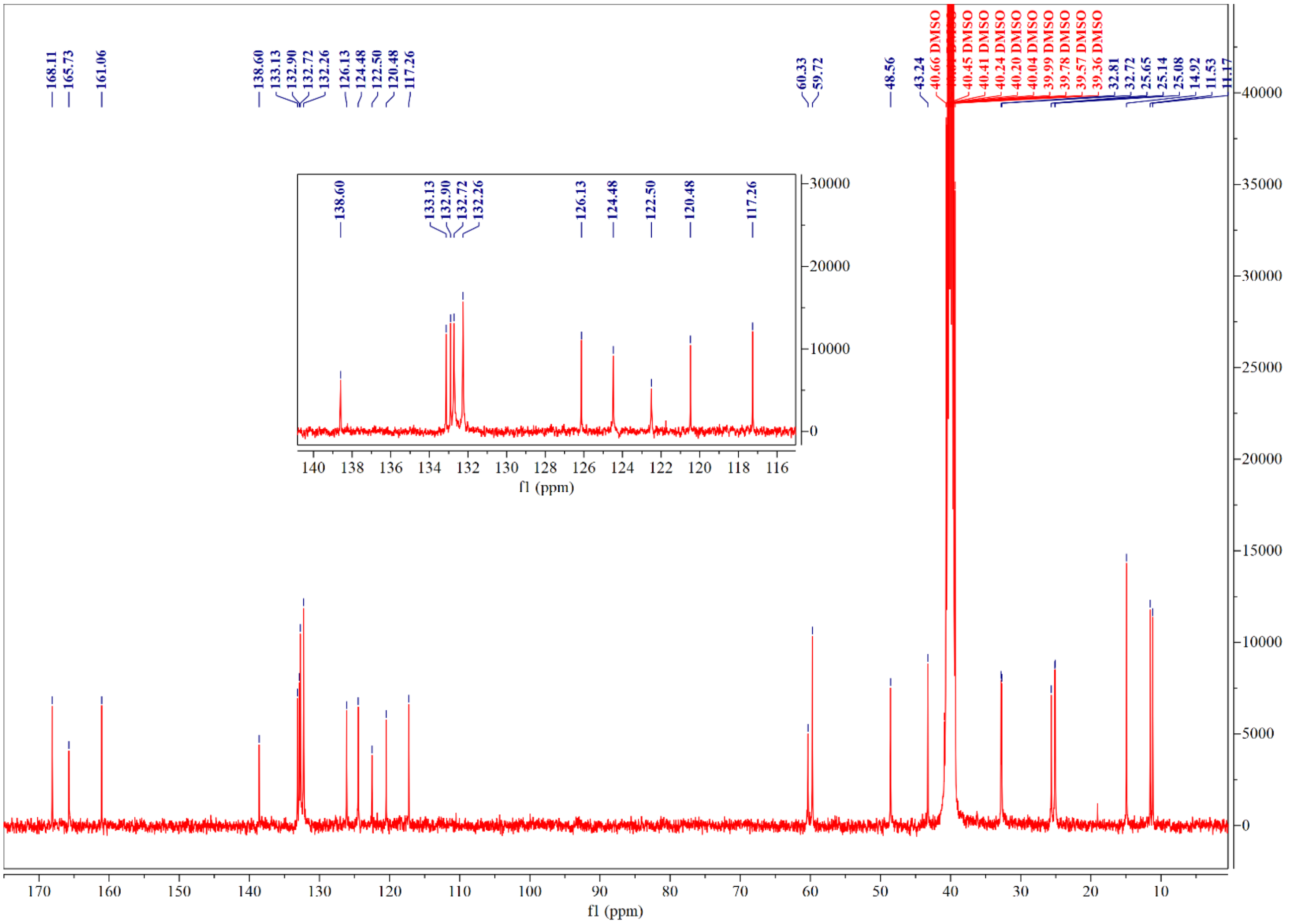

Figure S26. ^{13}C NMR spectrum of compound **7c**

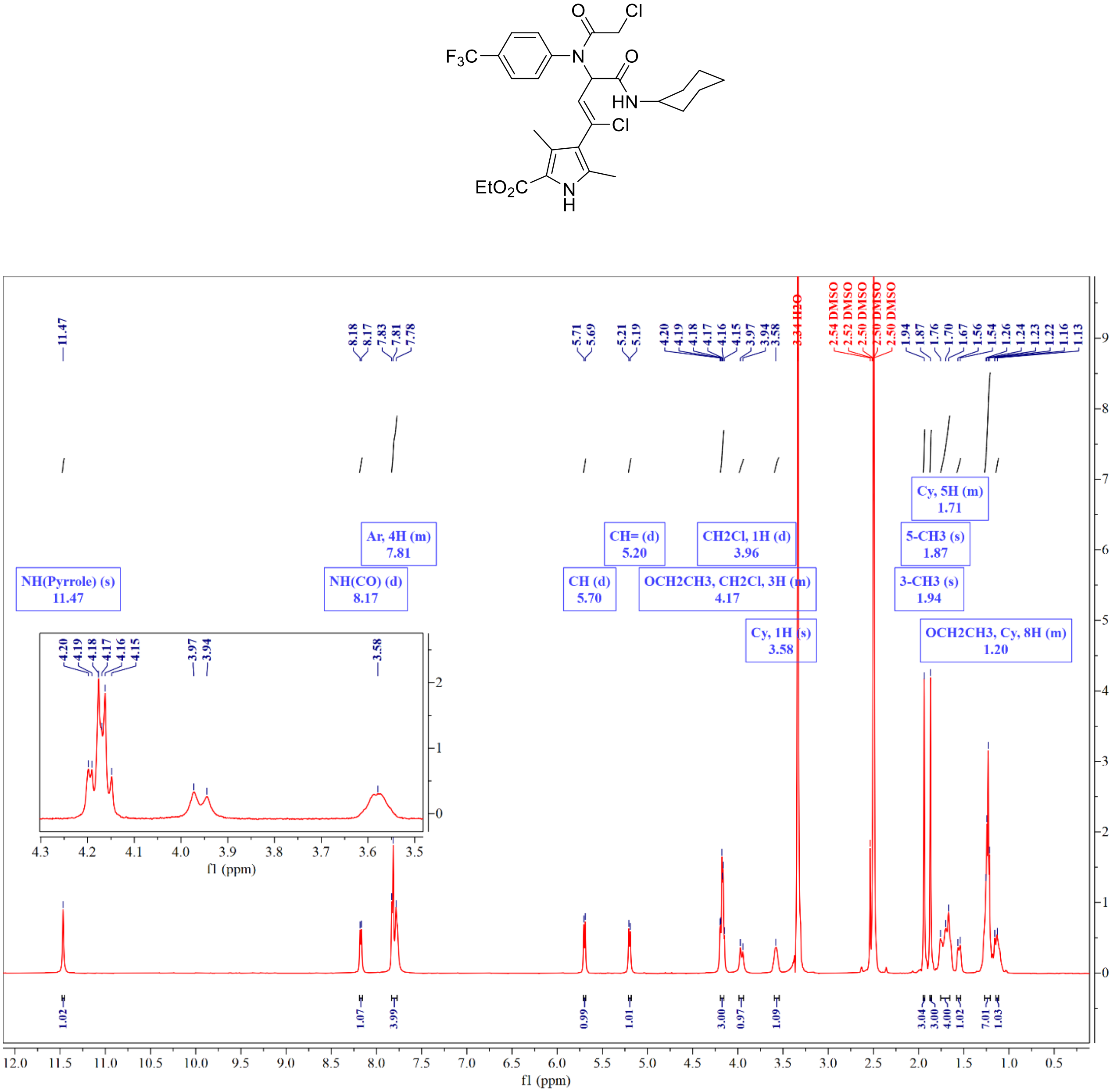

Figure S27. ^{1}H NMR spectrum of compound **7d** in DMSO-d_6.

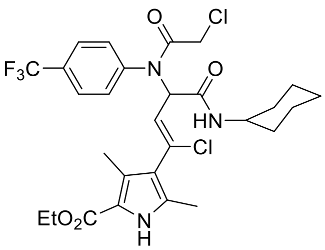

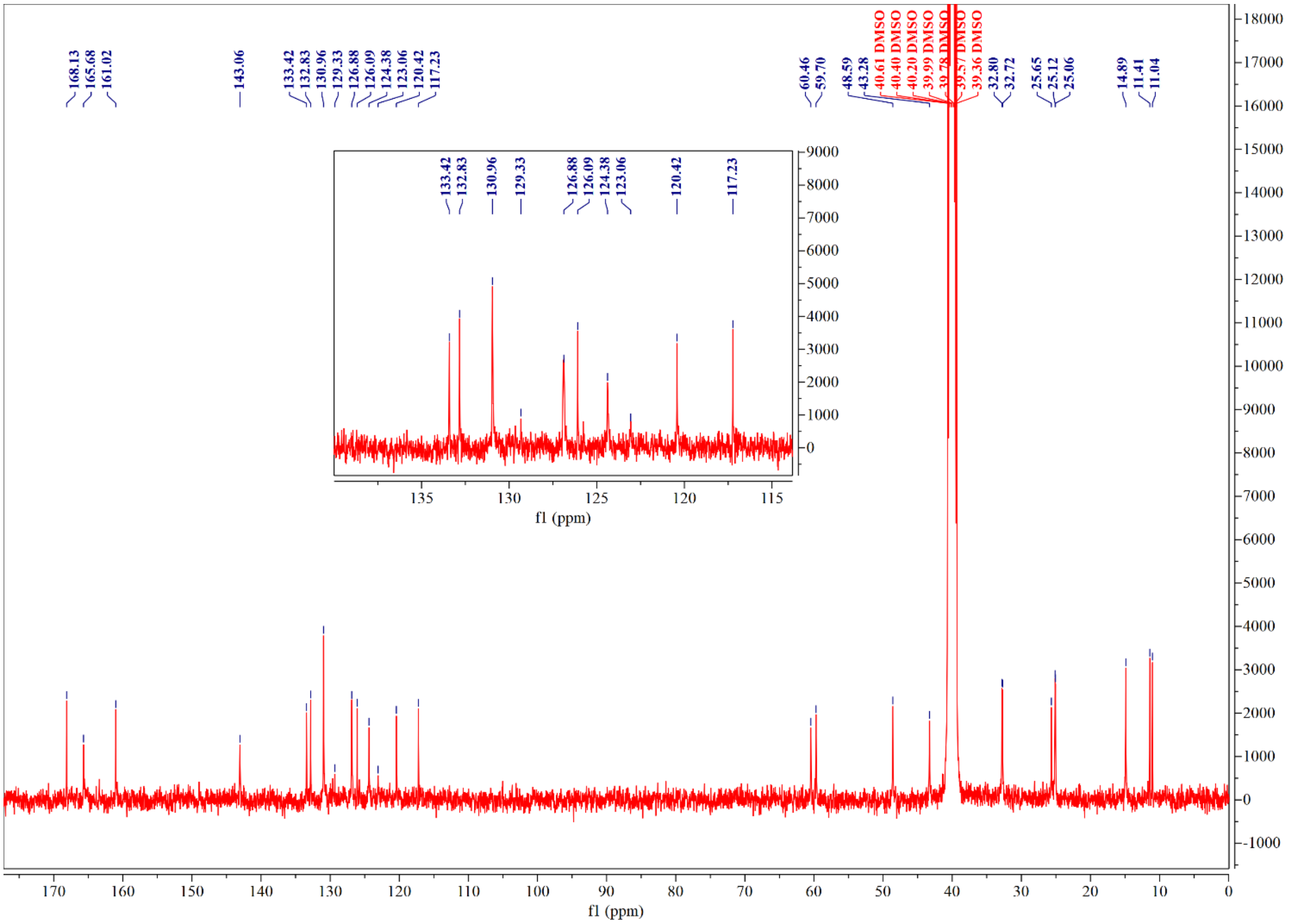

Figure S28. ^{13}C NMR spectrum of compound **7d**

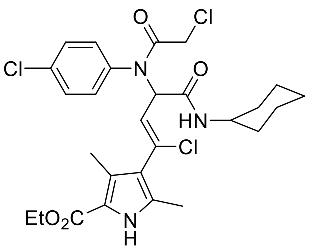

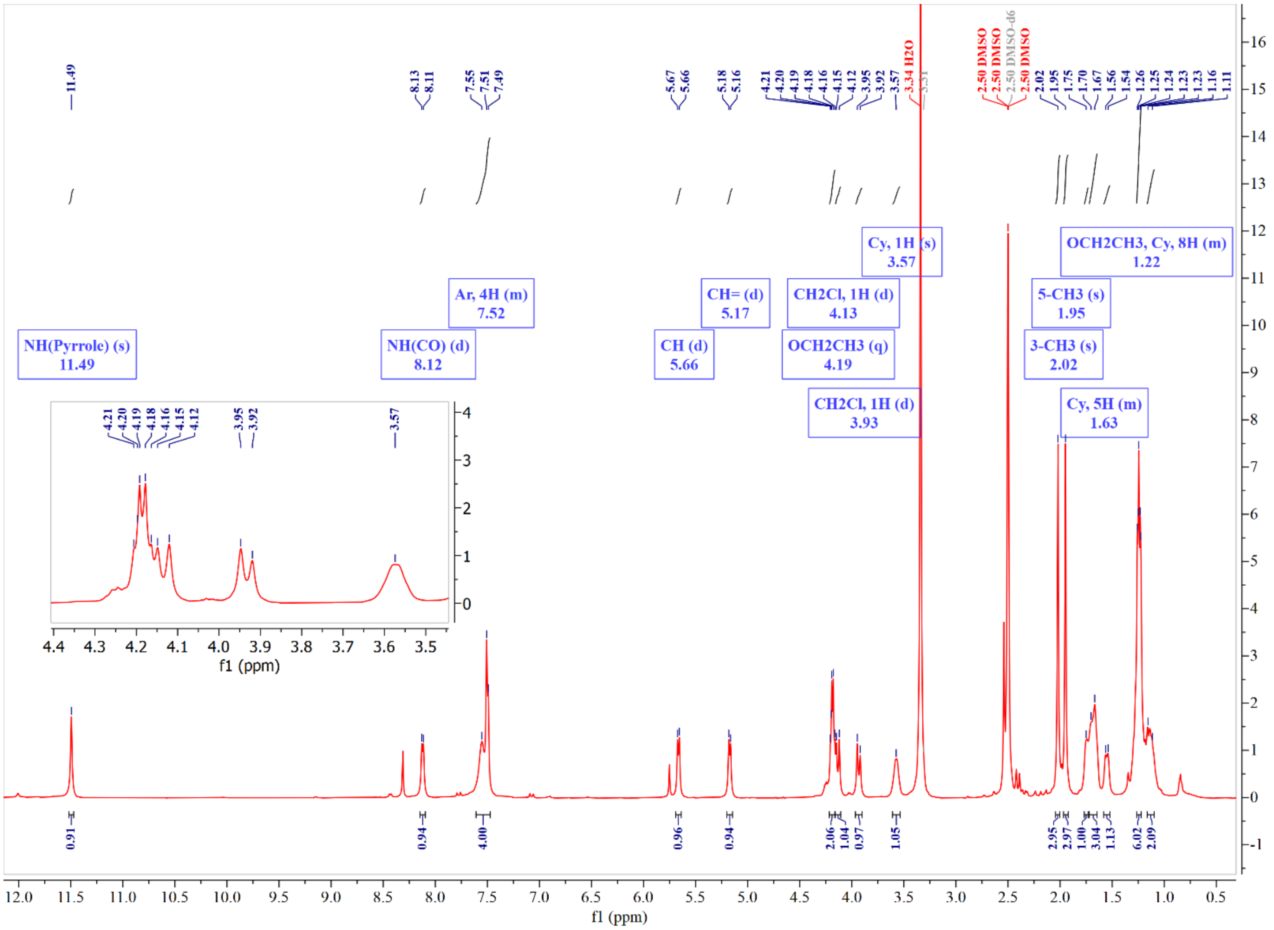

Figure S29. ^{1}H NMR spectrum of compound **7e** in DMSO-d_6.

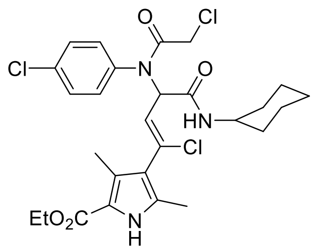

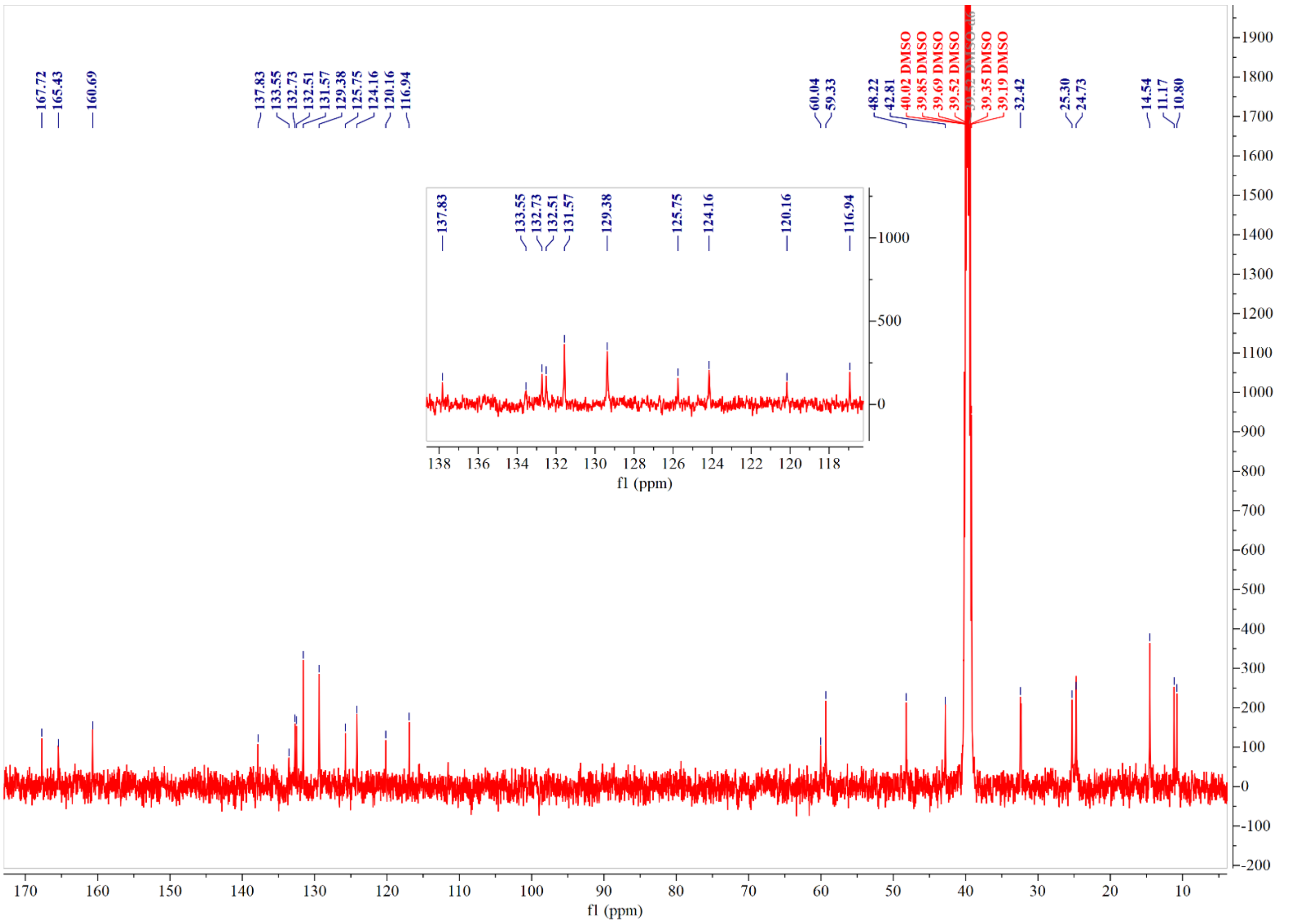

Figure S30. ^{13}C NMR spectrum of compound **7e**

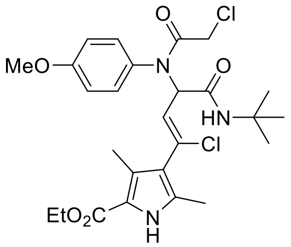

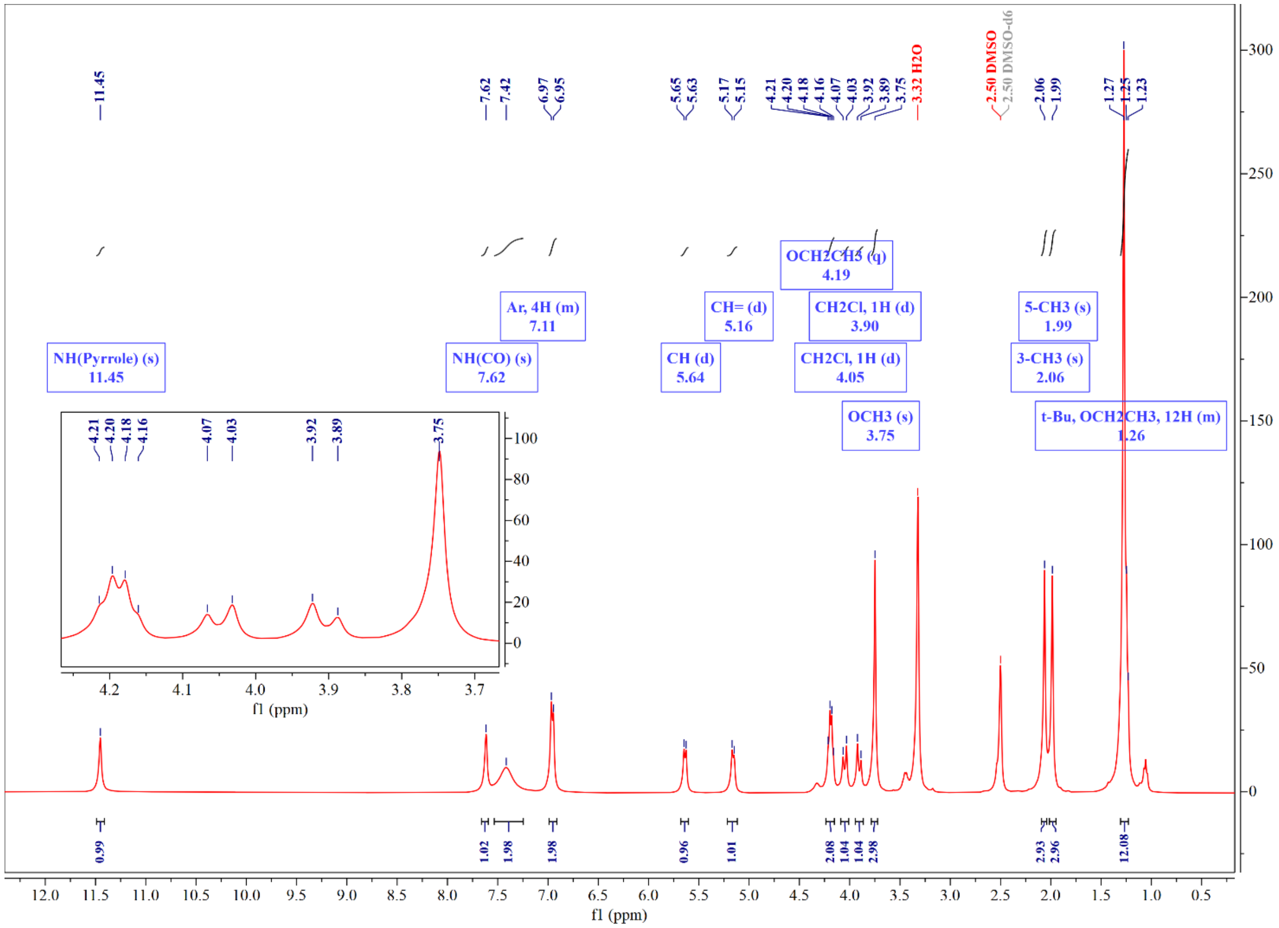

Figure S31. ^{1}H NMR spectrum of compound **8a** in DMSO-d_6.

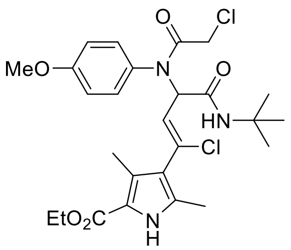

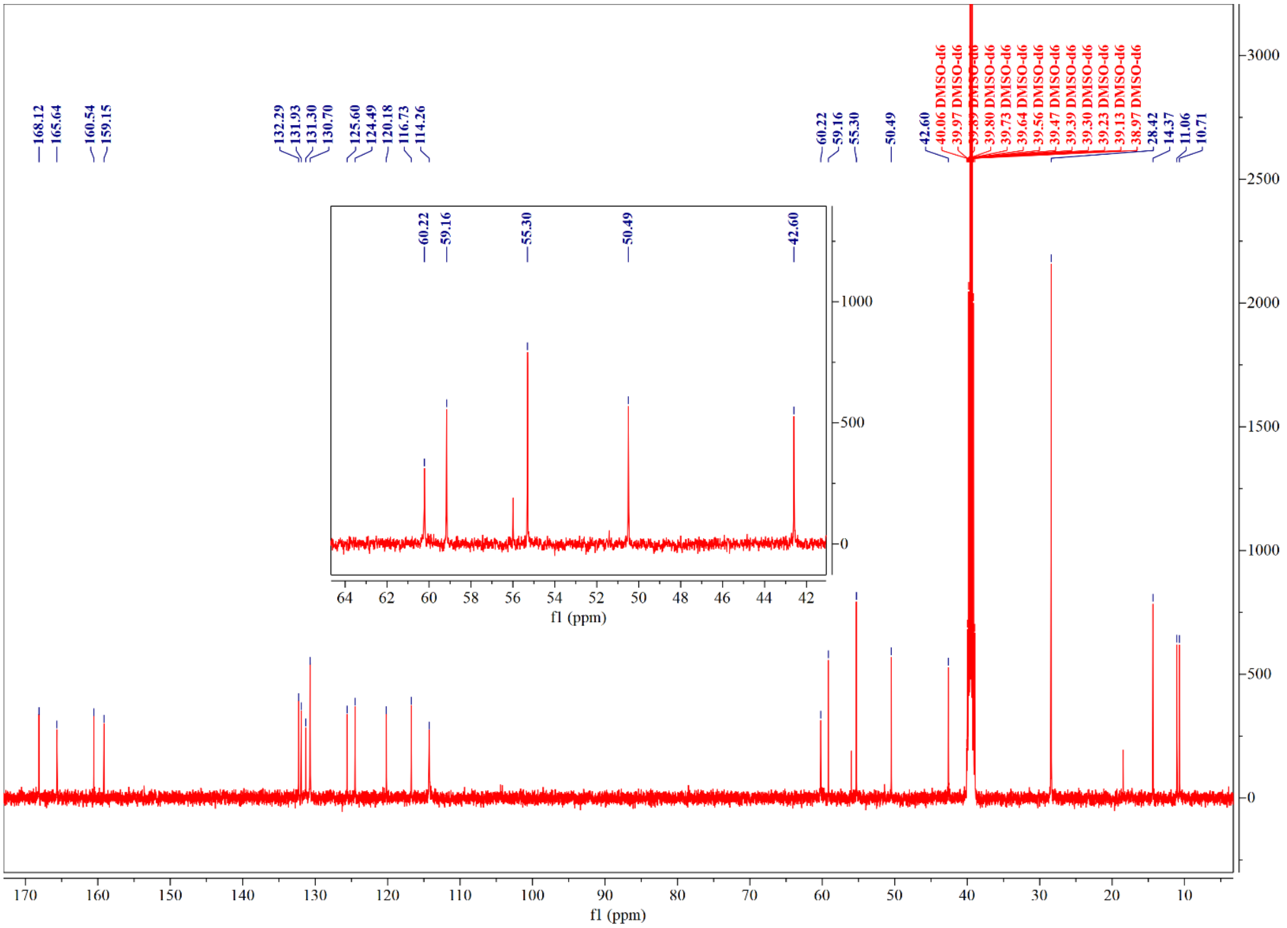

Figure S32. ^{13}C NMR spectrum of compound **8a**

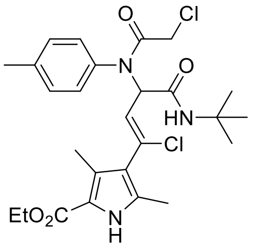

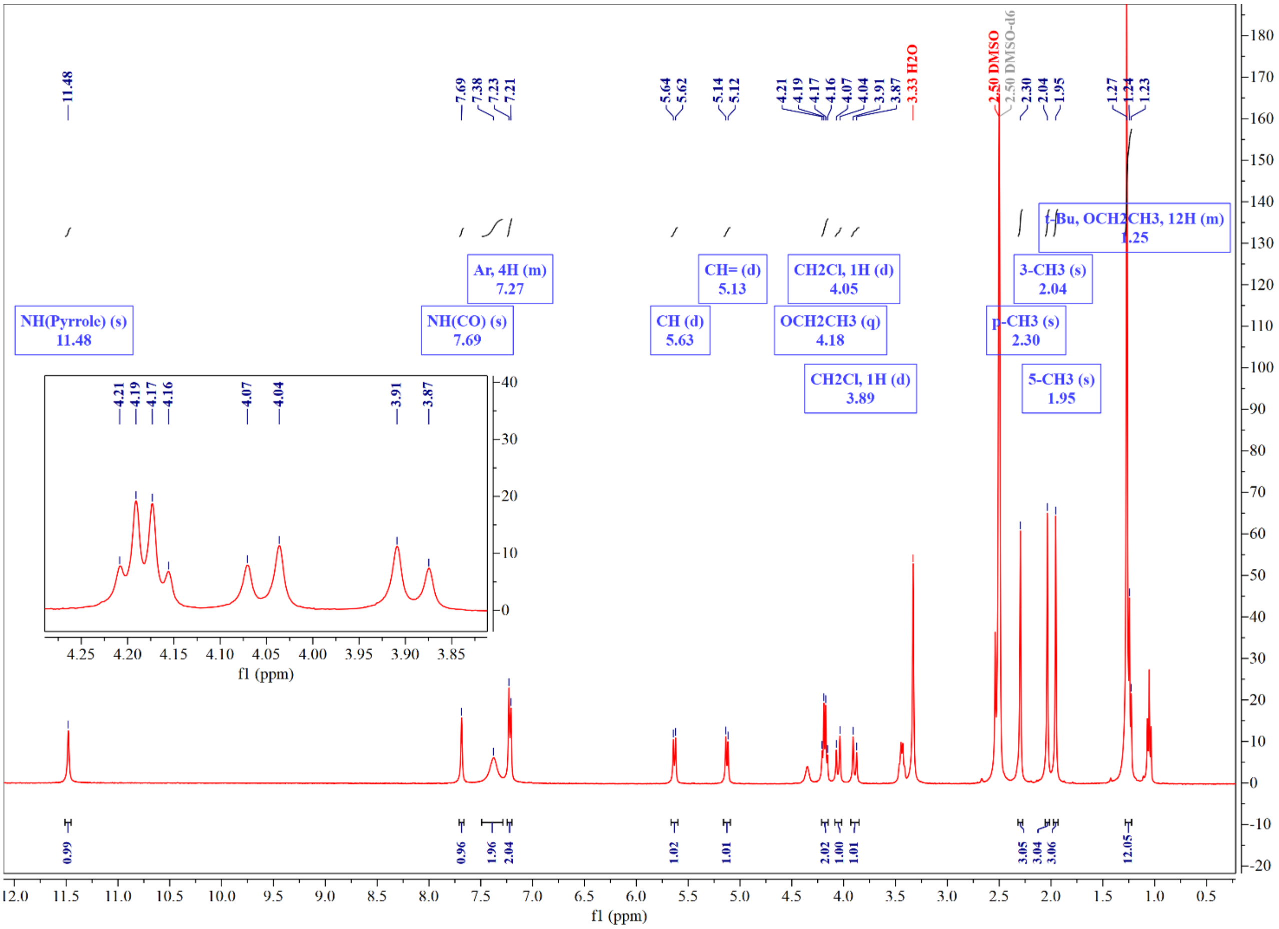

Figure S33. ^{1}H NMR spectrum of compound **8b** in DMSO-d_6.

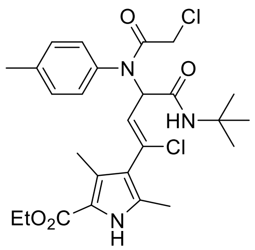

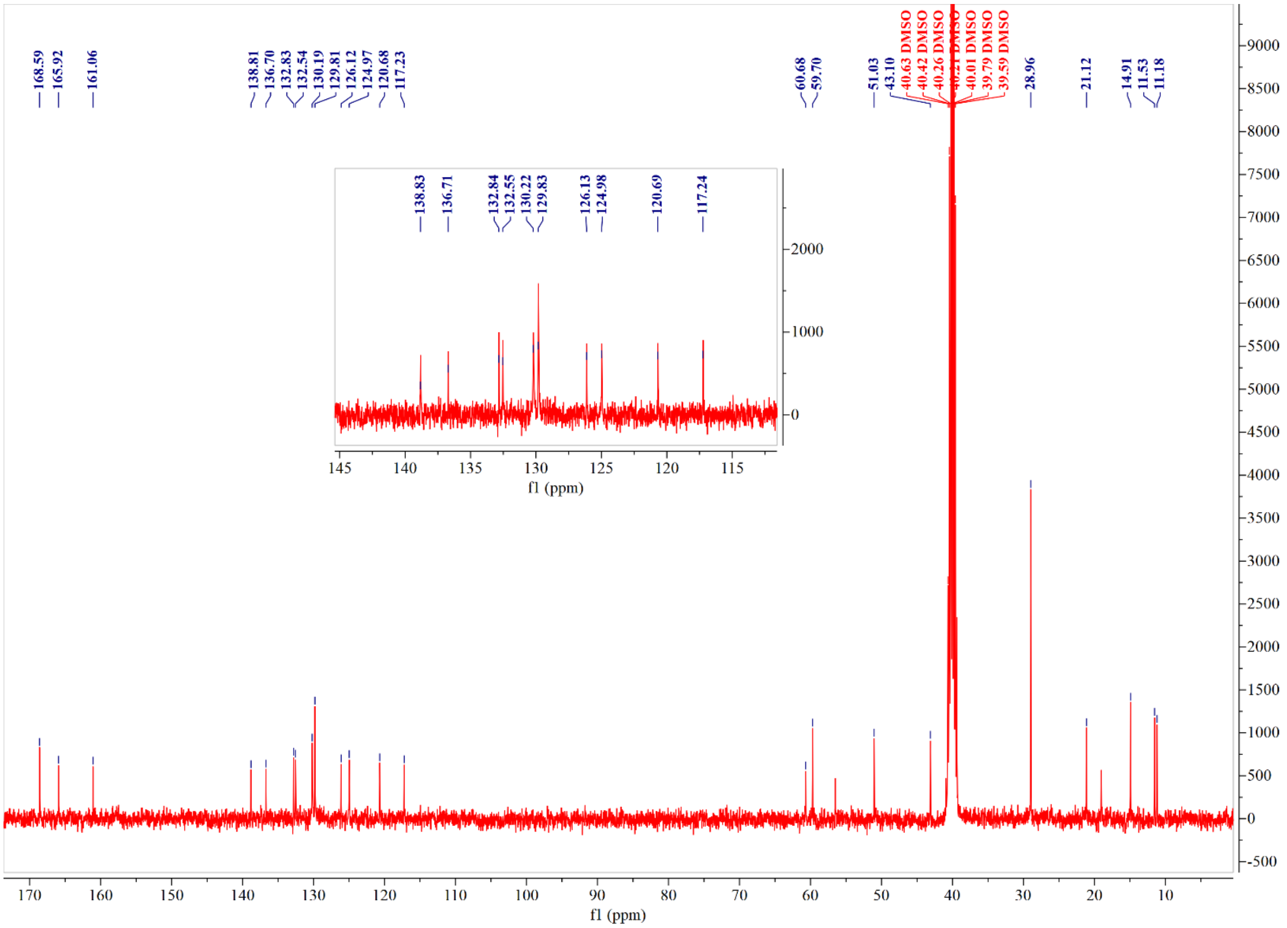

Figure S34. ^{13}C NMR spectrum of compound **8b**

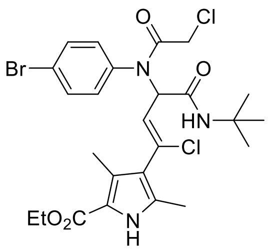

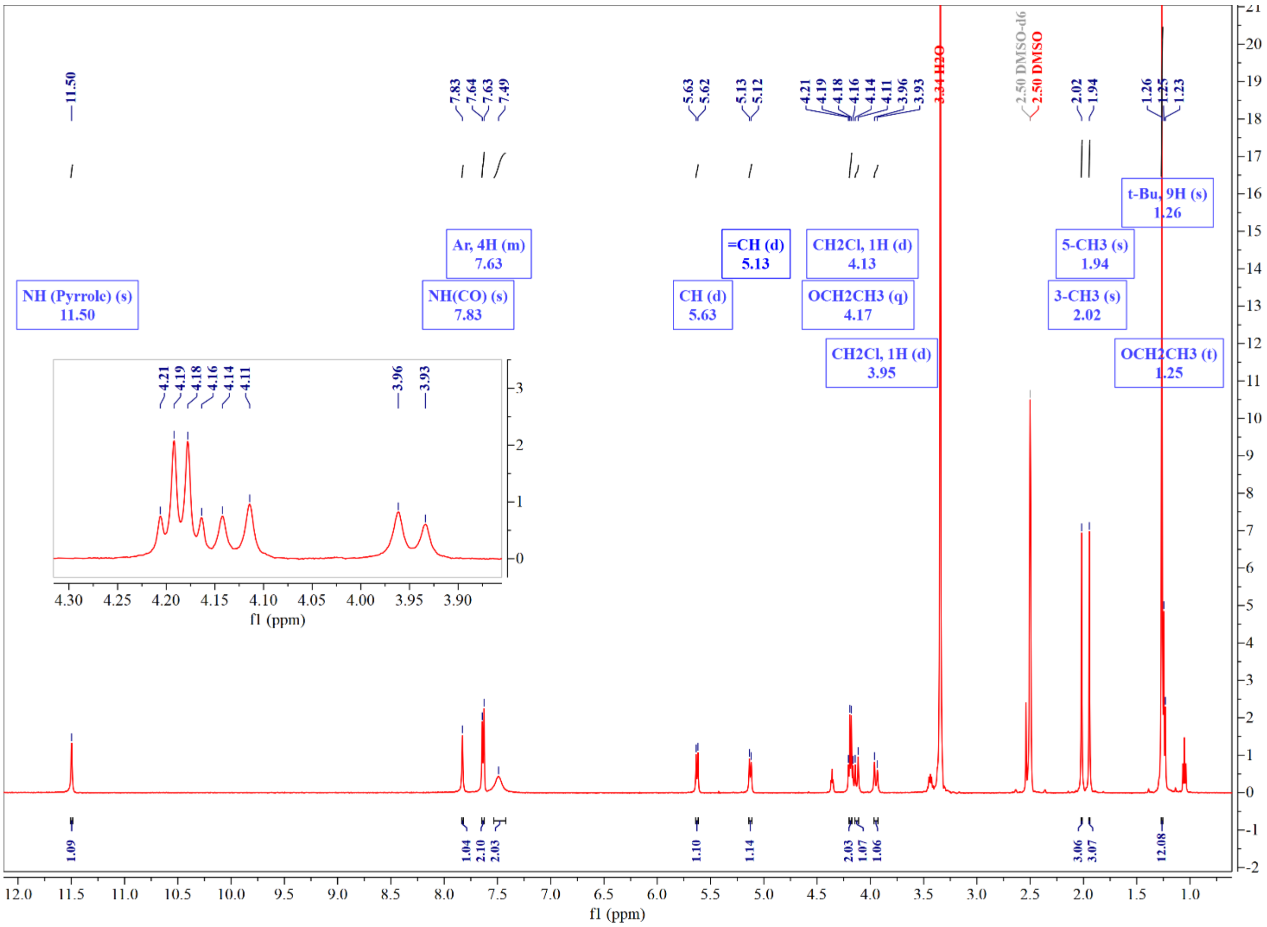

Figure S35. ^{1}H NMR spectrum of compound **8c** in DMSO-d_6.

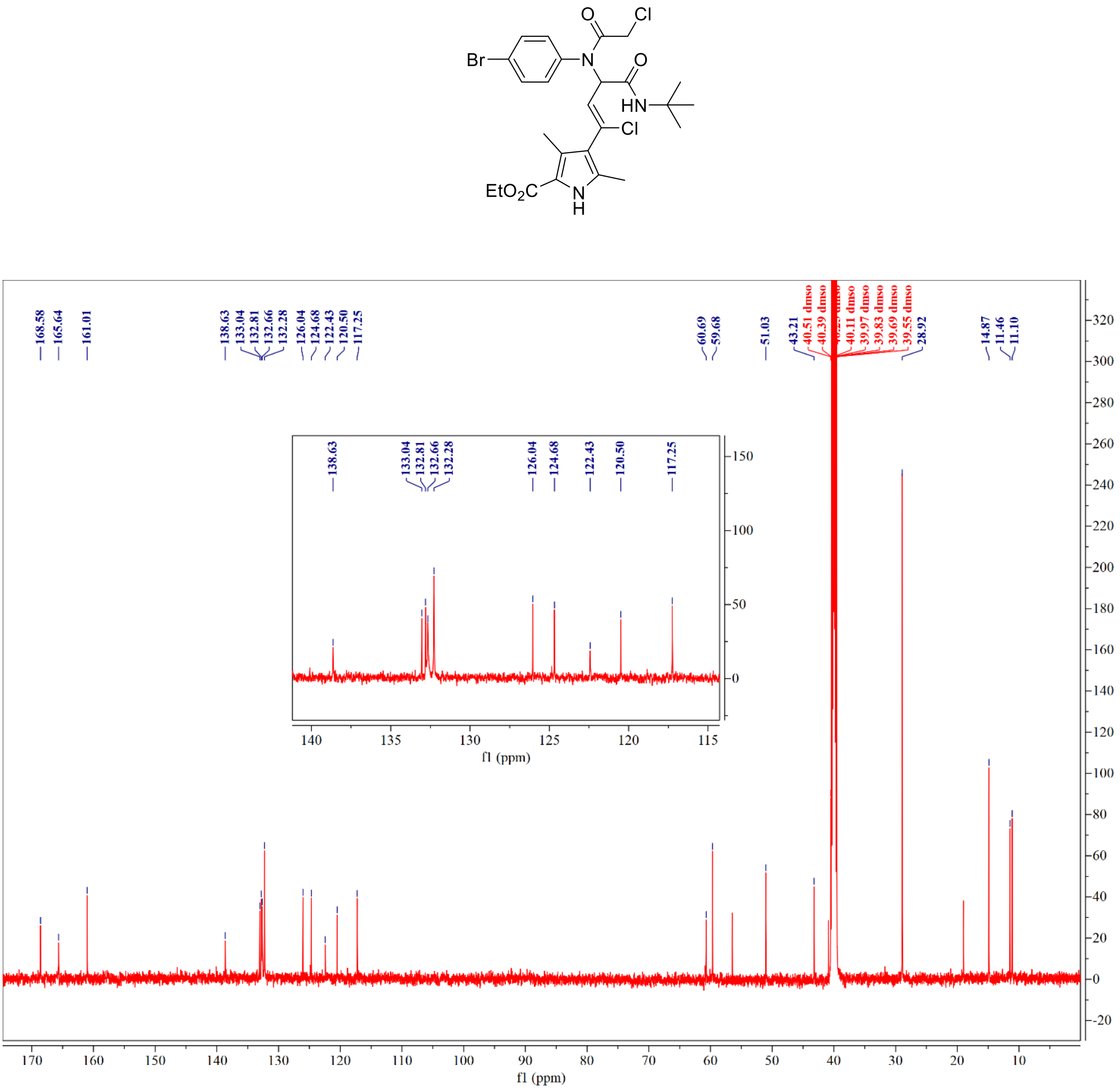

Figure S36. ^{13}C NMR spectrum of compound **8c**

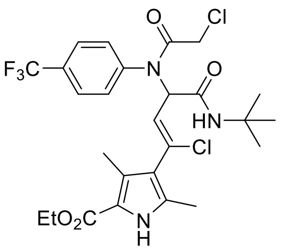

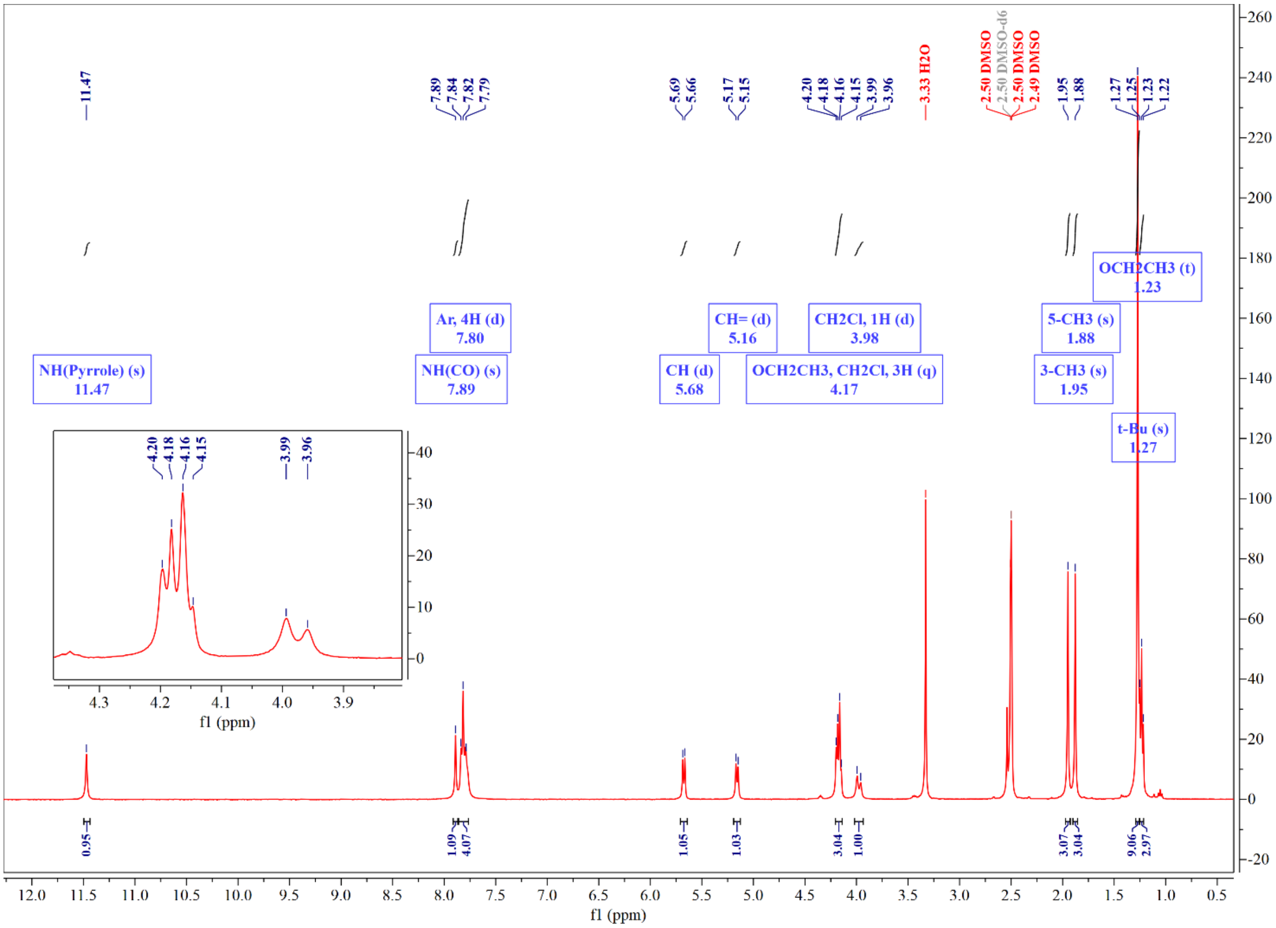

Figure S37. ^{1}H NMR spectrum of compound **8d** in DMSO-d_6.

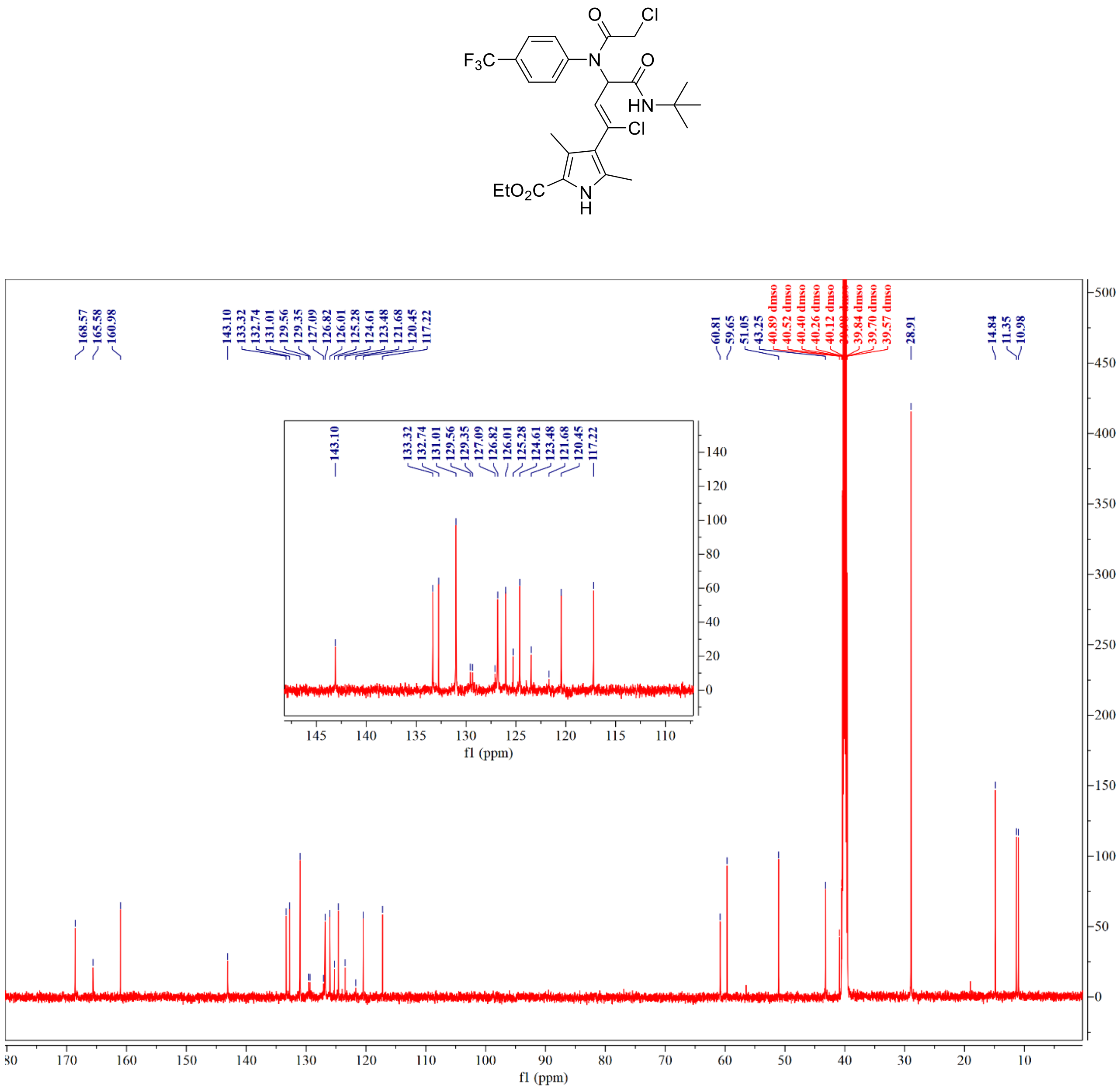

Figure S38. ^{13}C NMR spectrum of compound **8d**

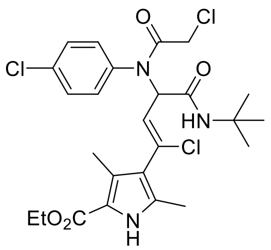

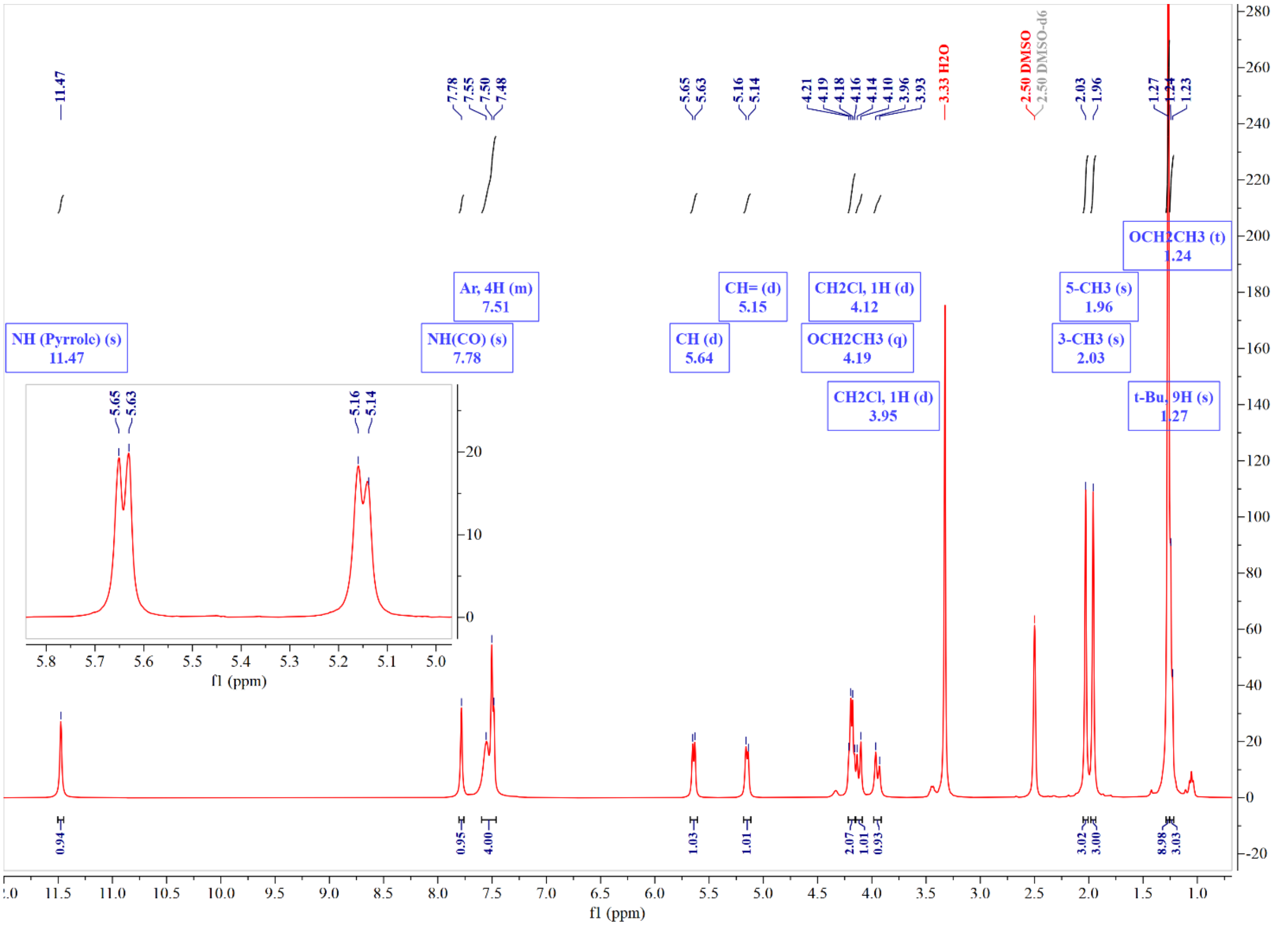

Figure S39. ^{1}H NMR spectrum of compound **8e** in DMSO-d_6.

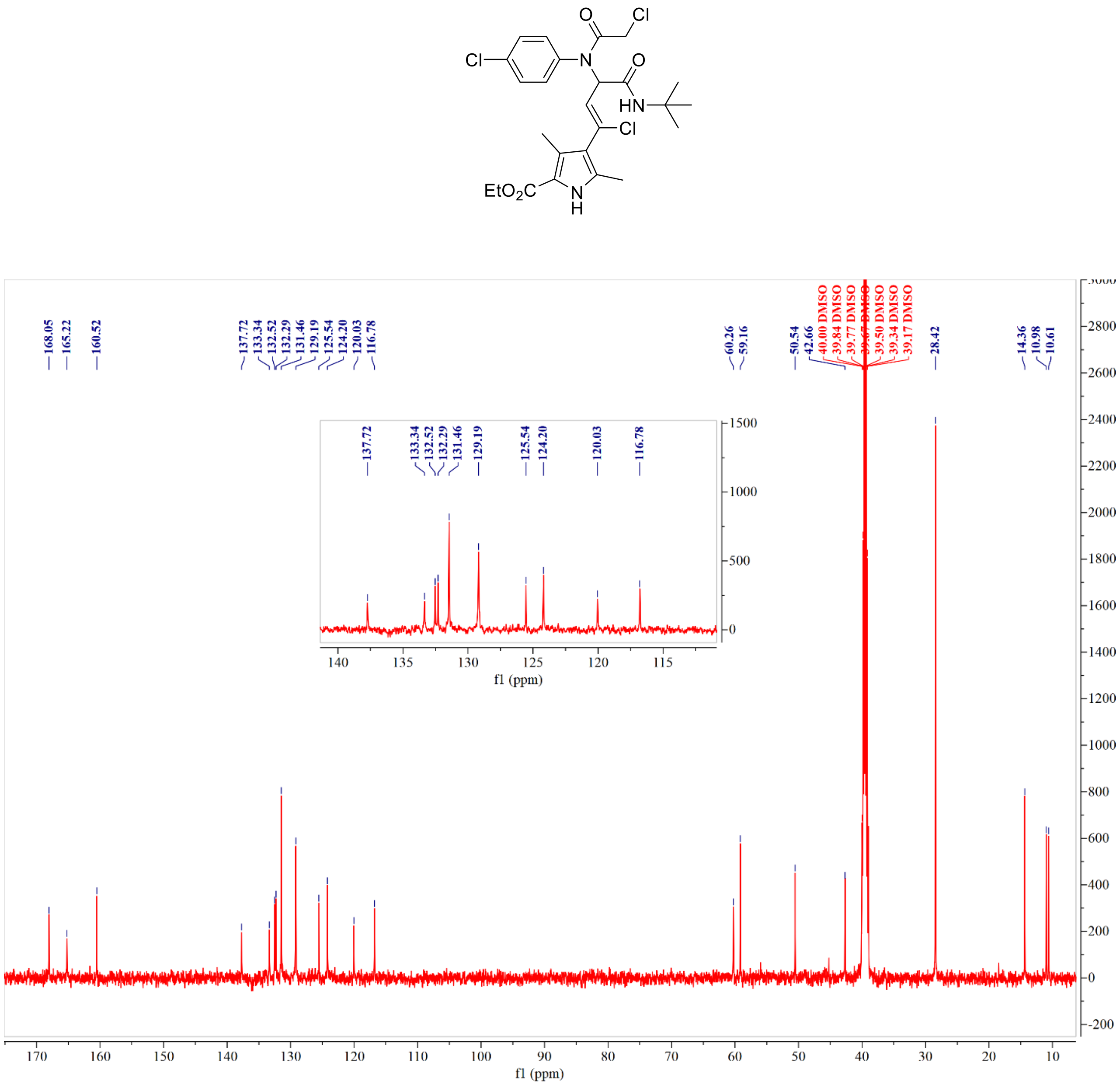

Figure S40. ^{13}C NMR spectrum of compound **8e**

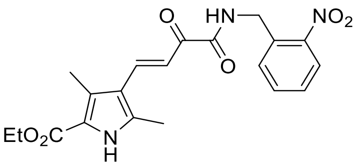

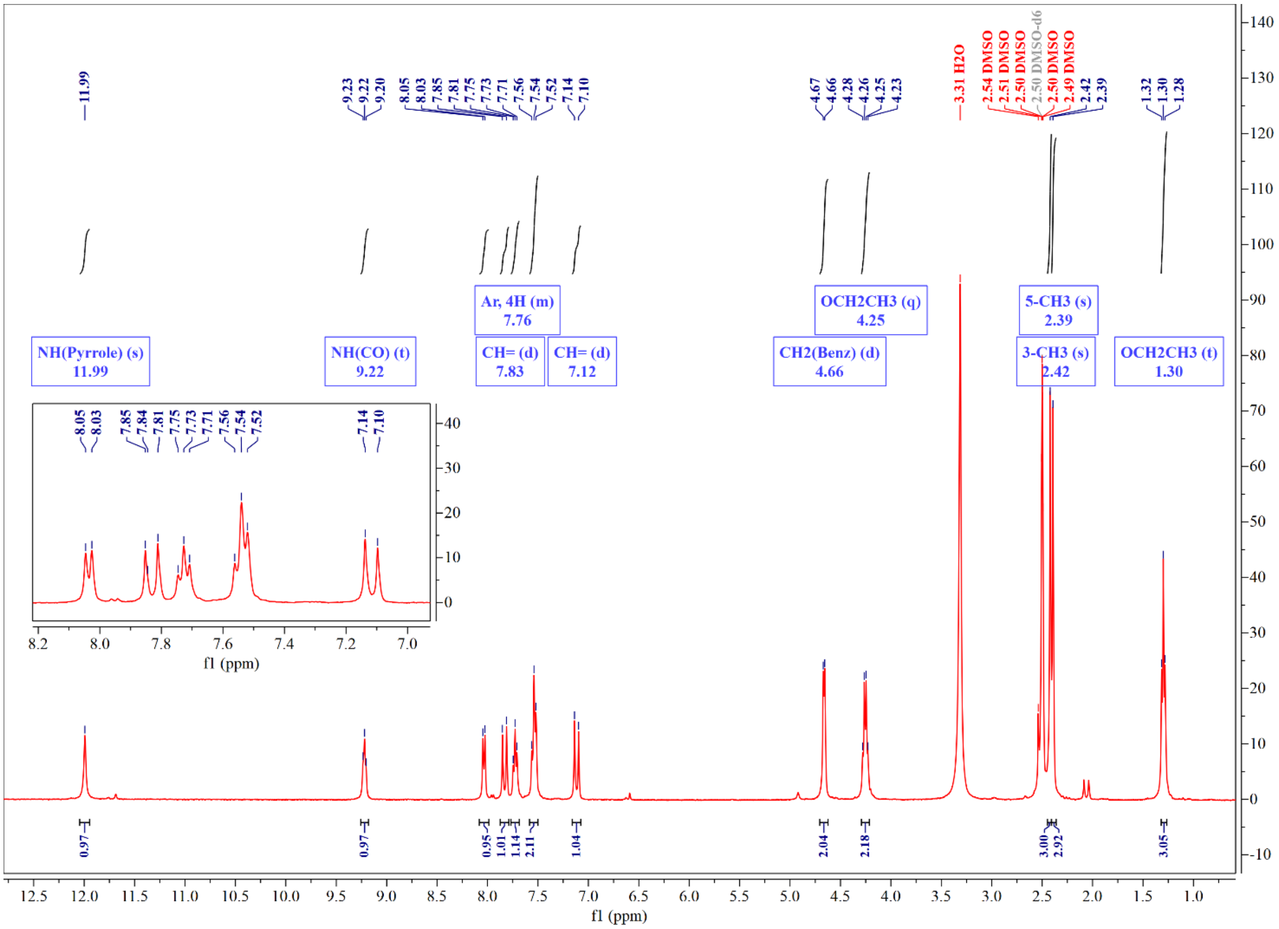

Figure S41. ^{1}H NMR spectrum of compound **10a** in DMSO-d_6.

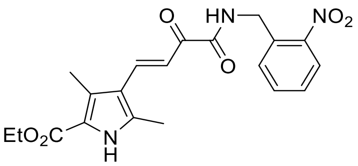

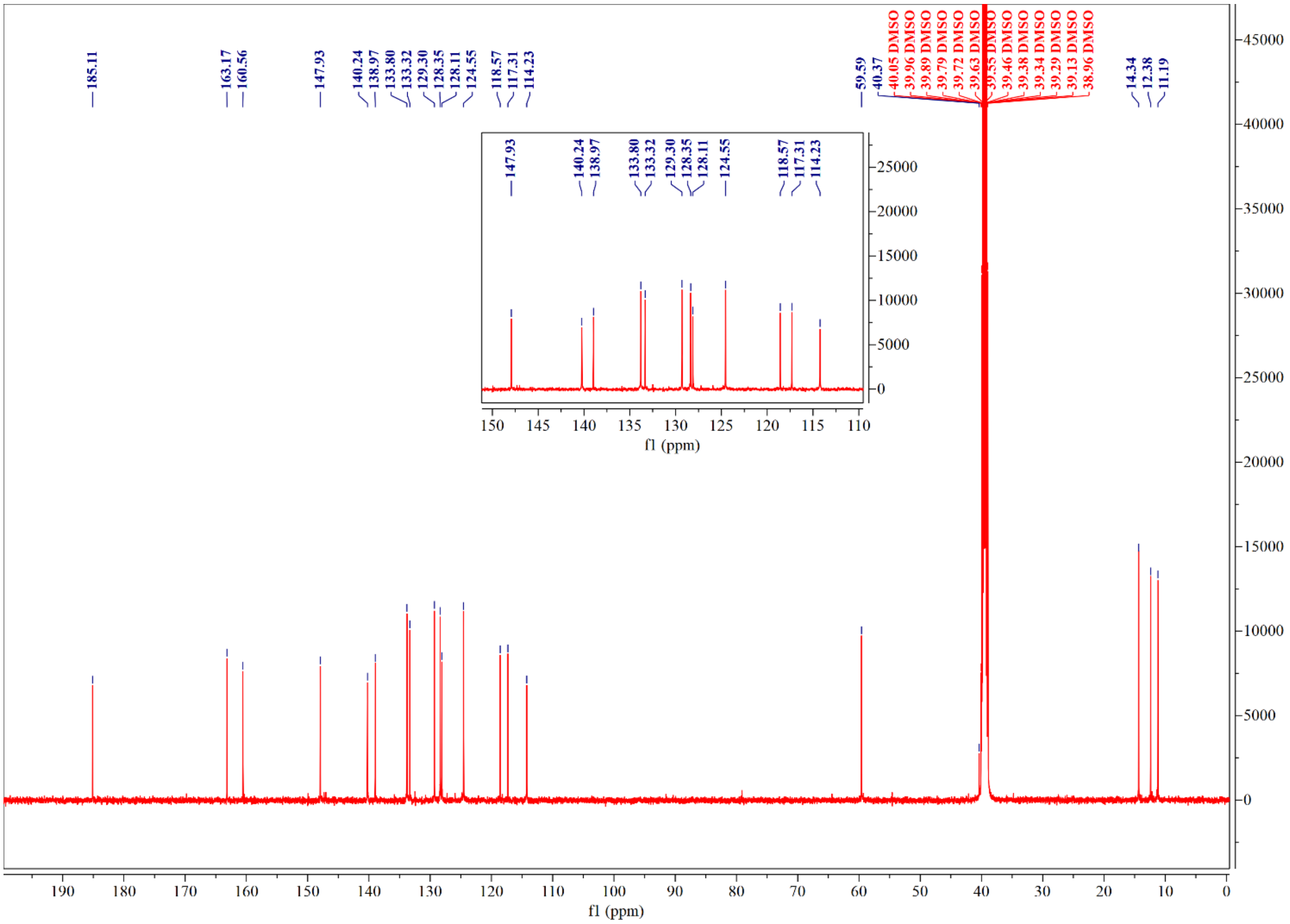

Figure S42. ^{13}C NMR spectrum of compound **10a**

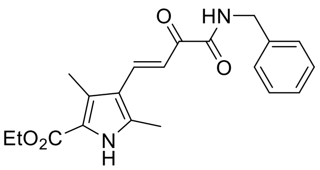

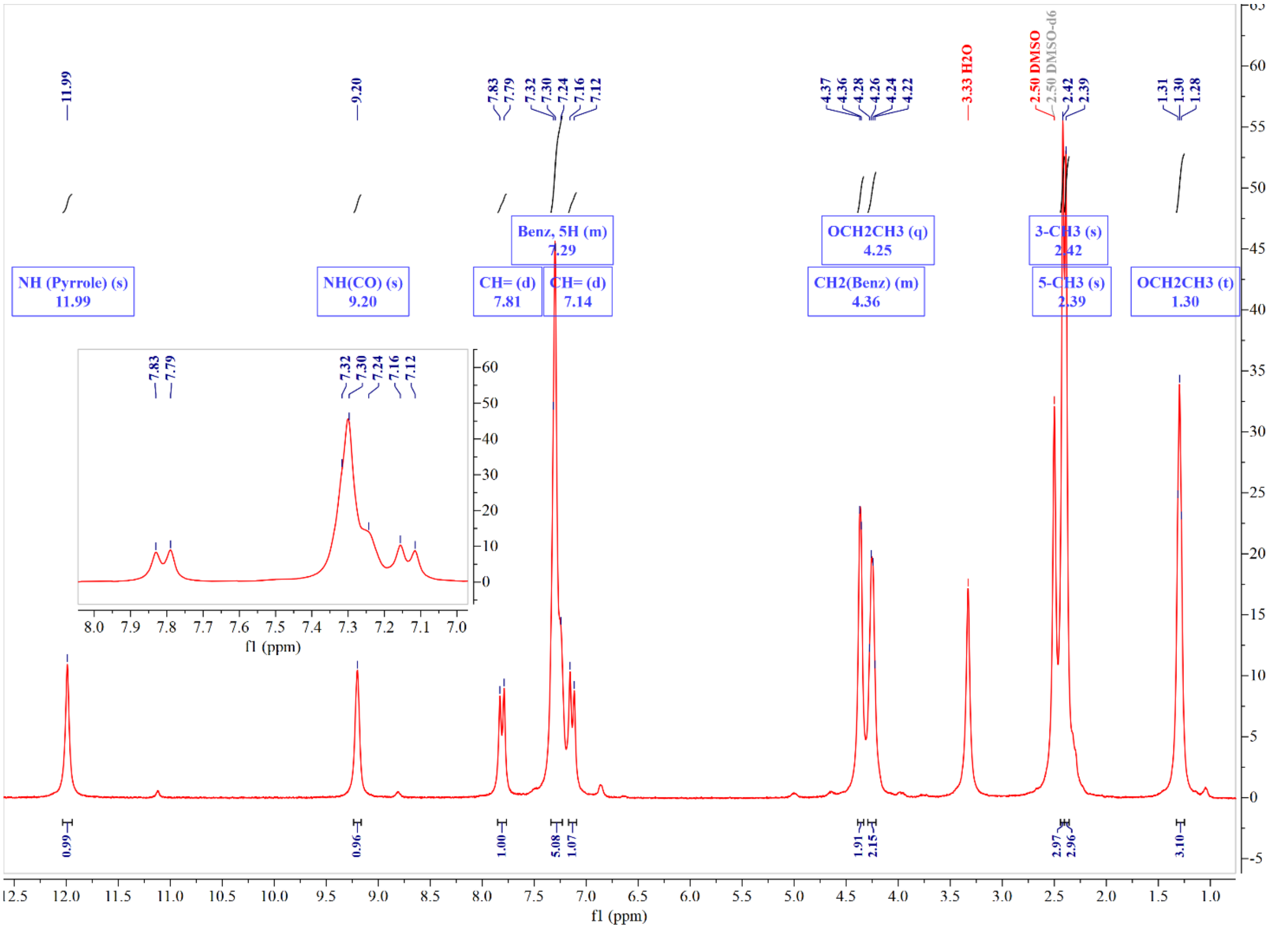

Figure S43. ^{1}H NMR spectrum of compound **10b** in DMSO-d_6.

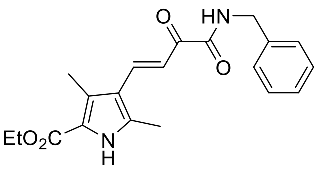

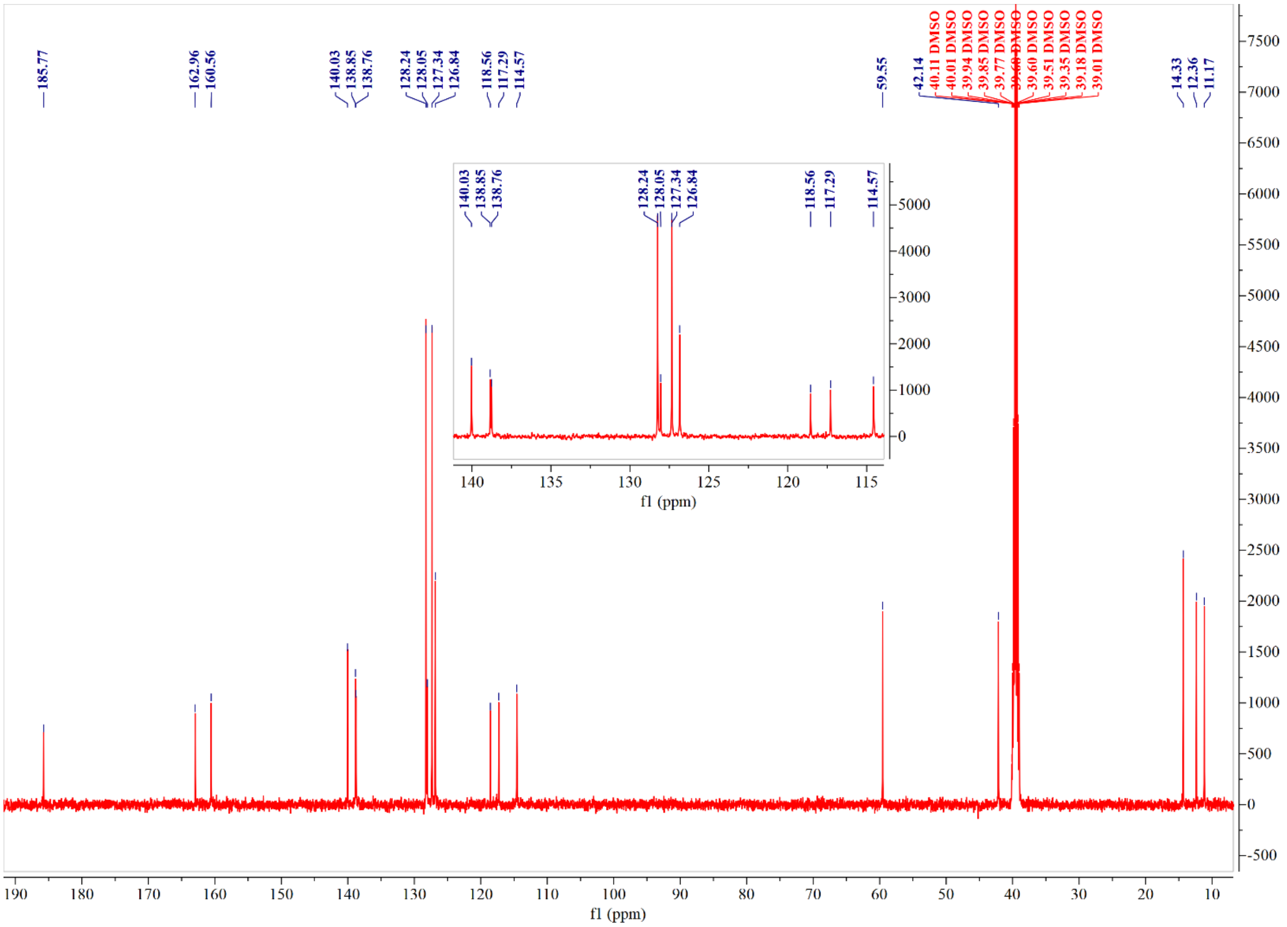

Figure S44. ^{13}C NMR spectrum of compound **10b**

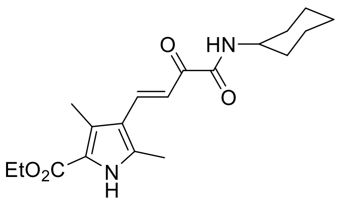

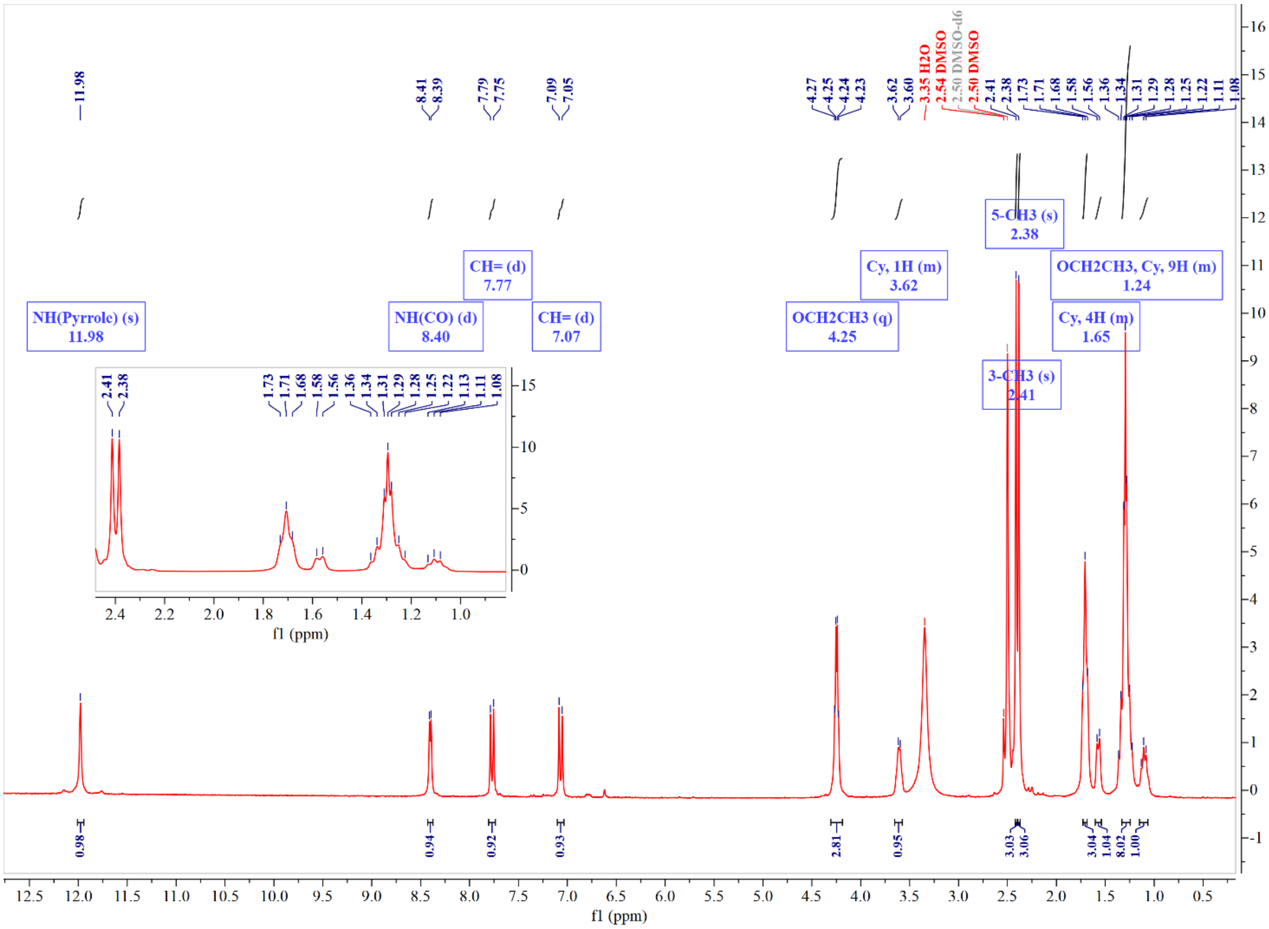

Figure S45. ^{1}H NMR spectrum of compound **10c** in DMSO-d_6.

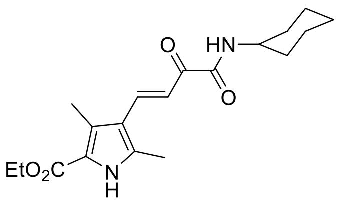

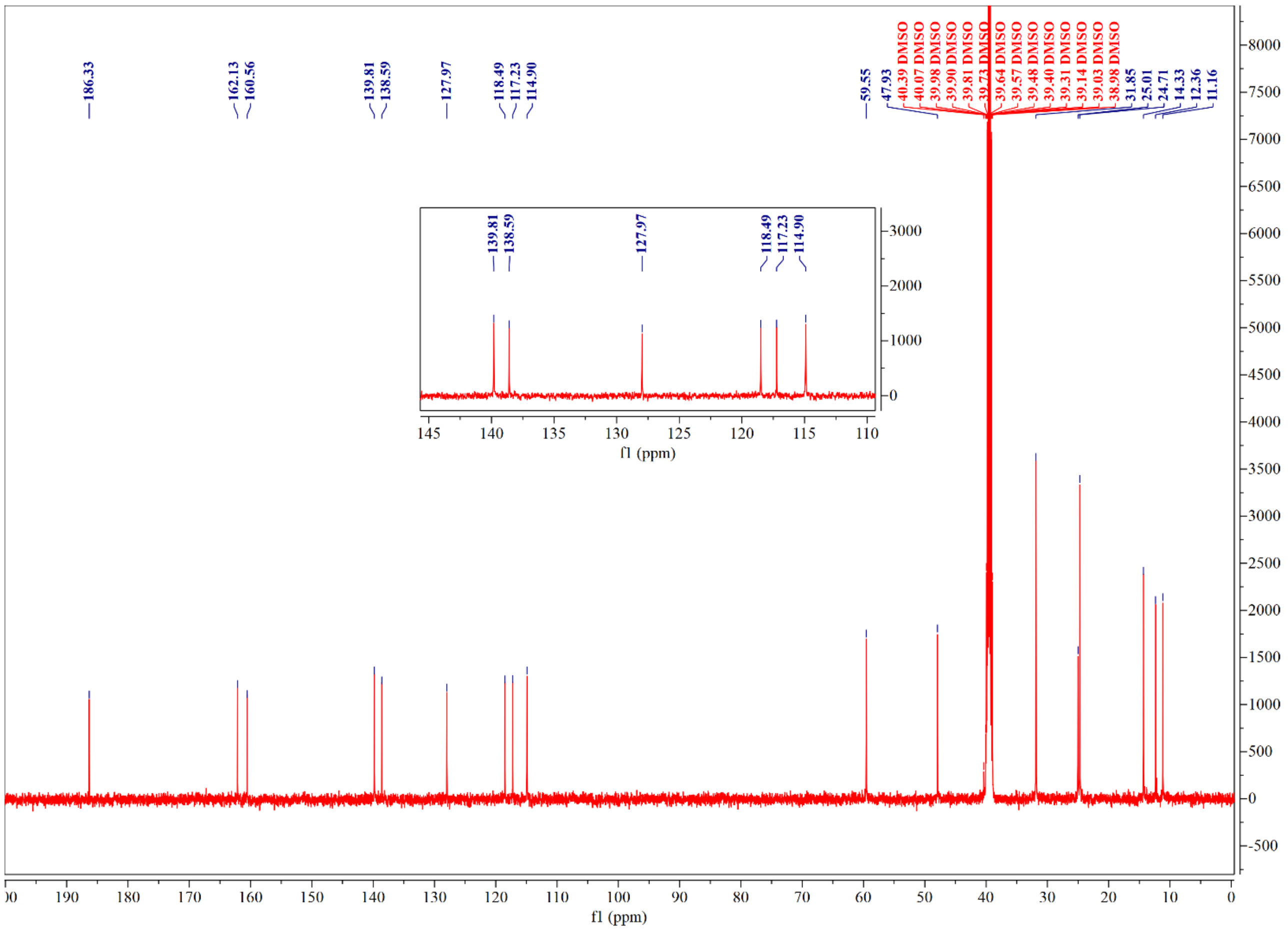

Figure S46. ^{13}C NMR spectrum of compound **10c**

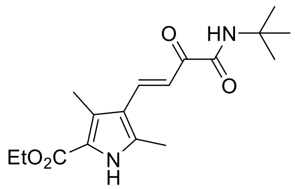

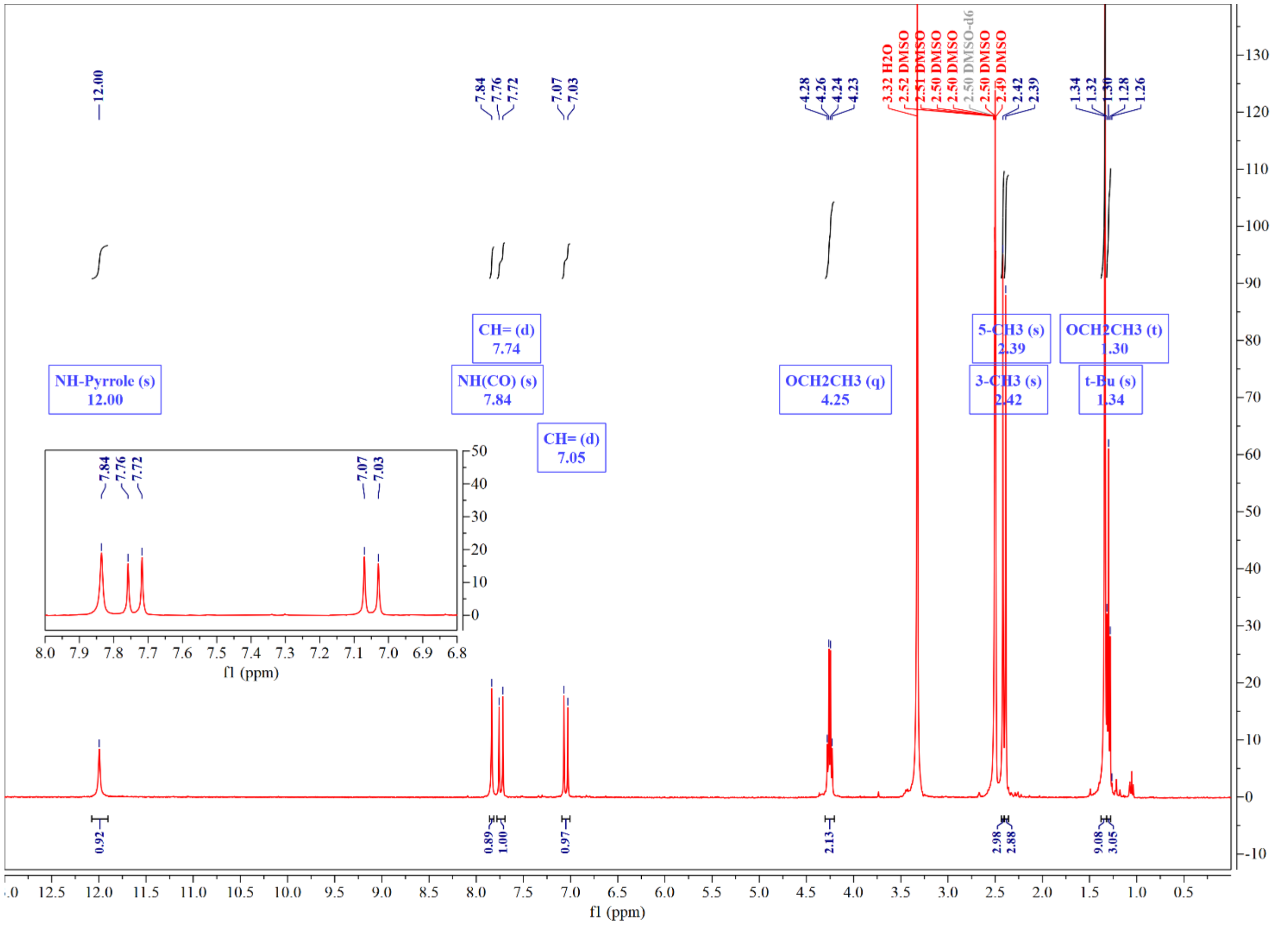

Figure S47. ^{1}H NMR spectrum of compound **10d** in DMSO-d_6.

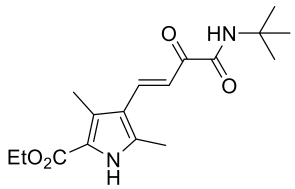

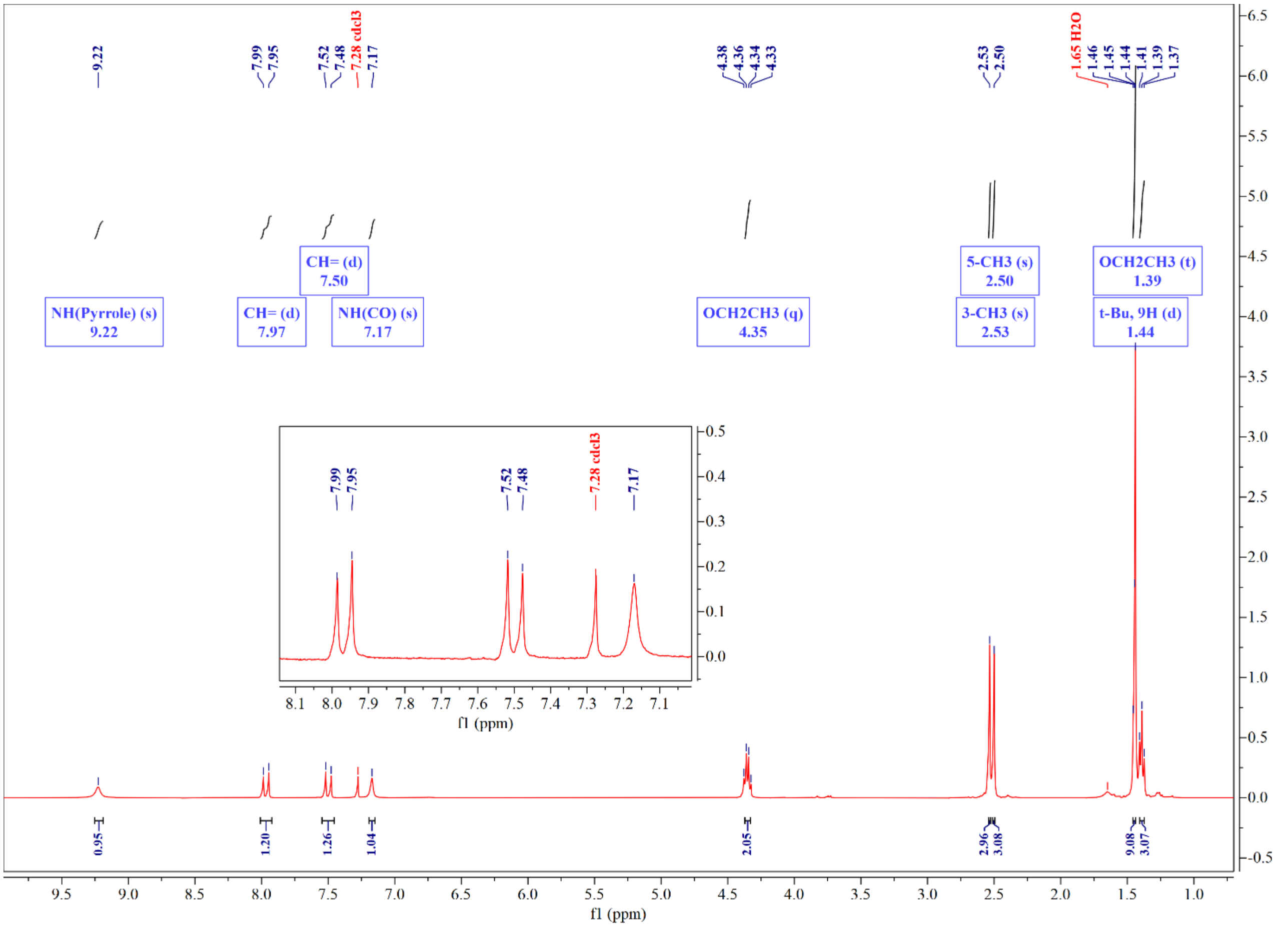

Figure S48. ^{1}H NMR spectrum of compound **10d** in $CDCl_3$

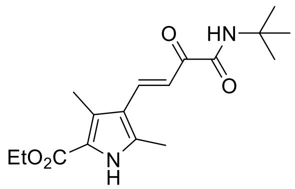

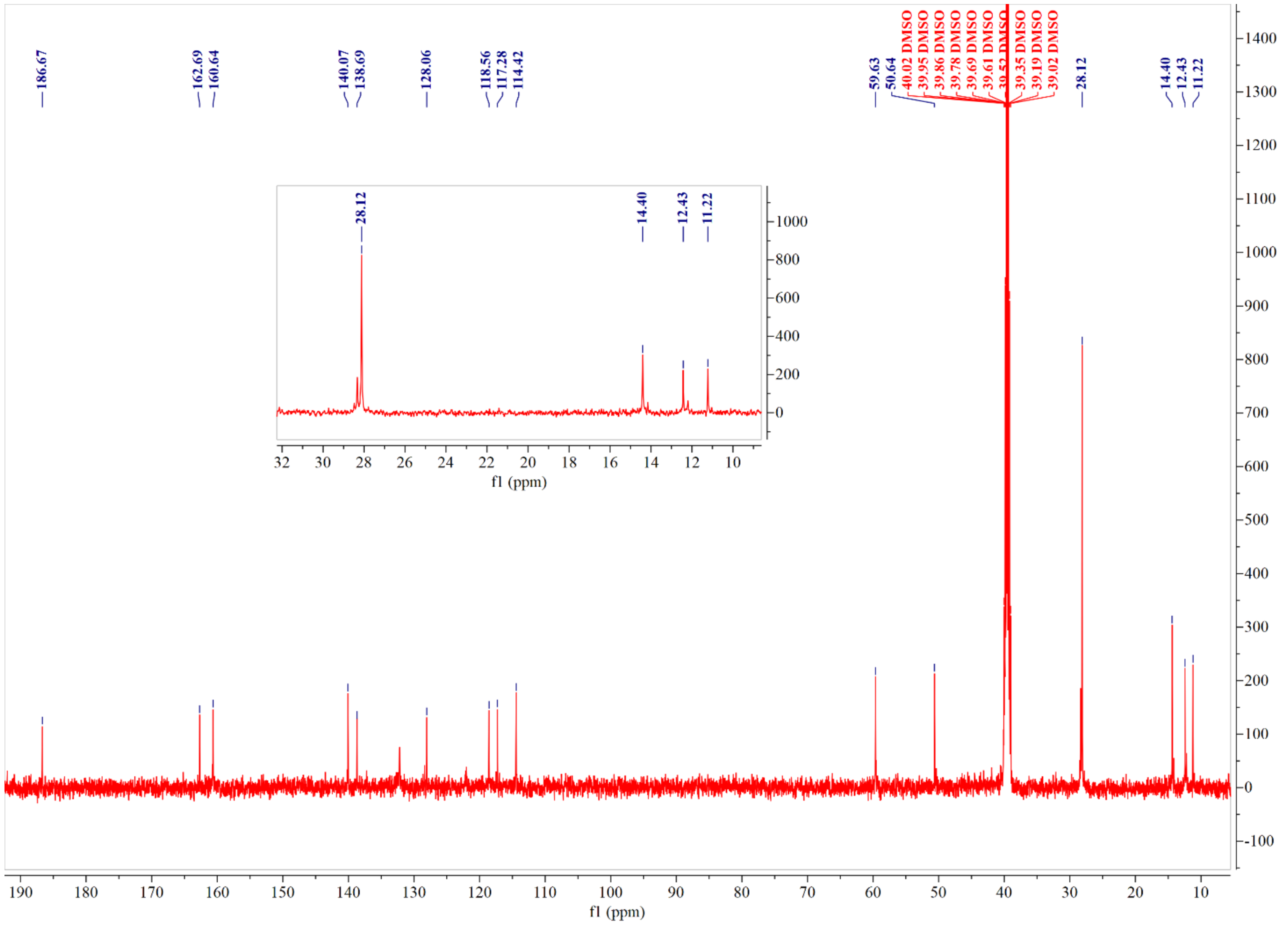

Figure S49. ^{13}C NMR spectrum of compound **10d**

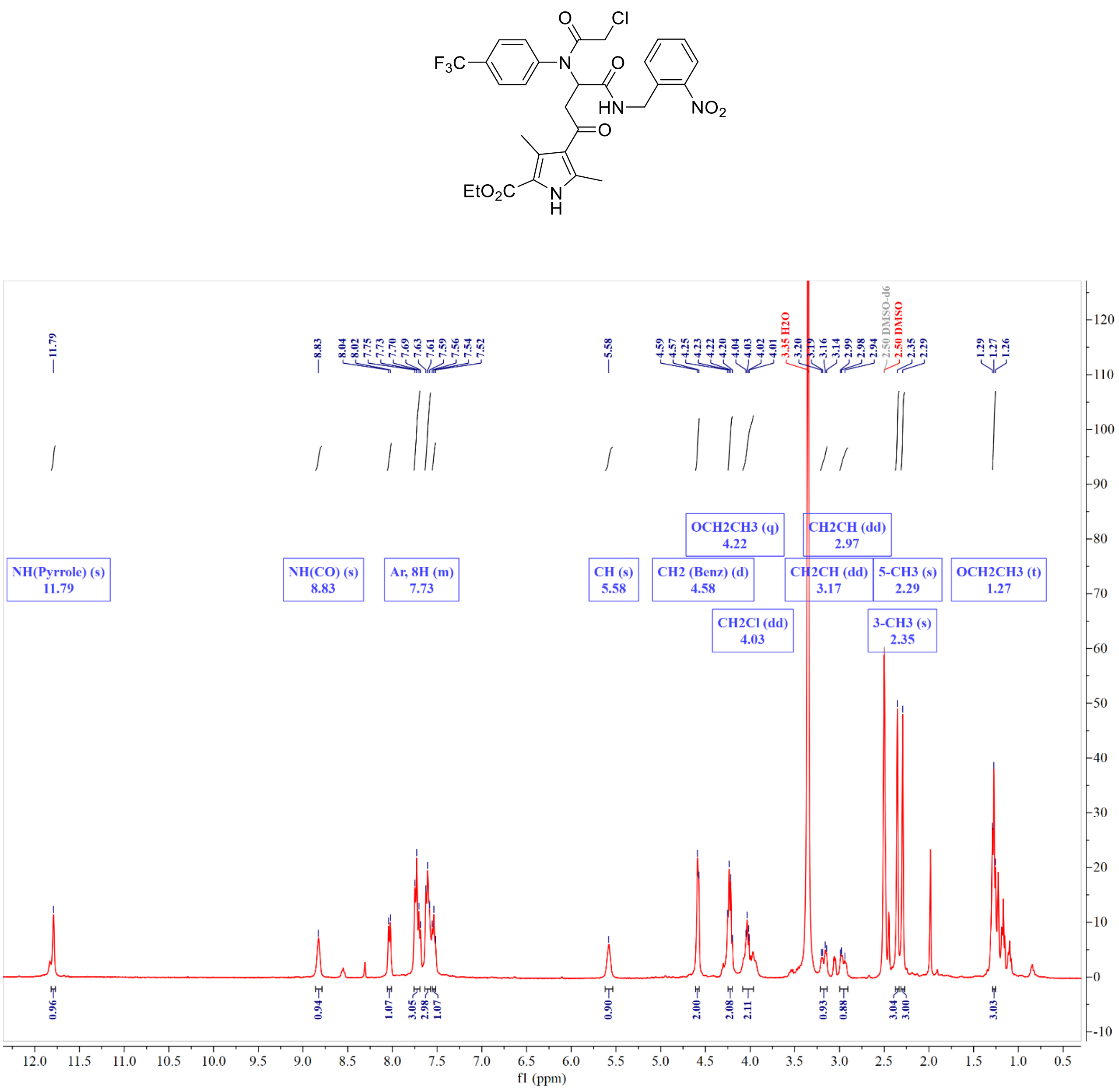

Figure S50. ^{1}H NMR spectrum of compound **12a** in DMSO-d_6.

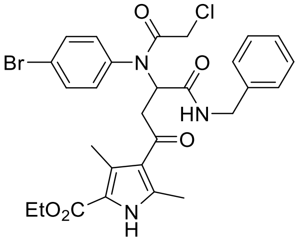

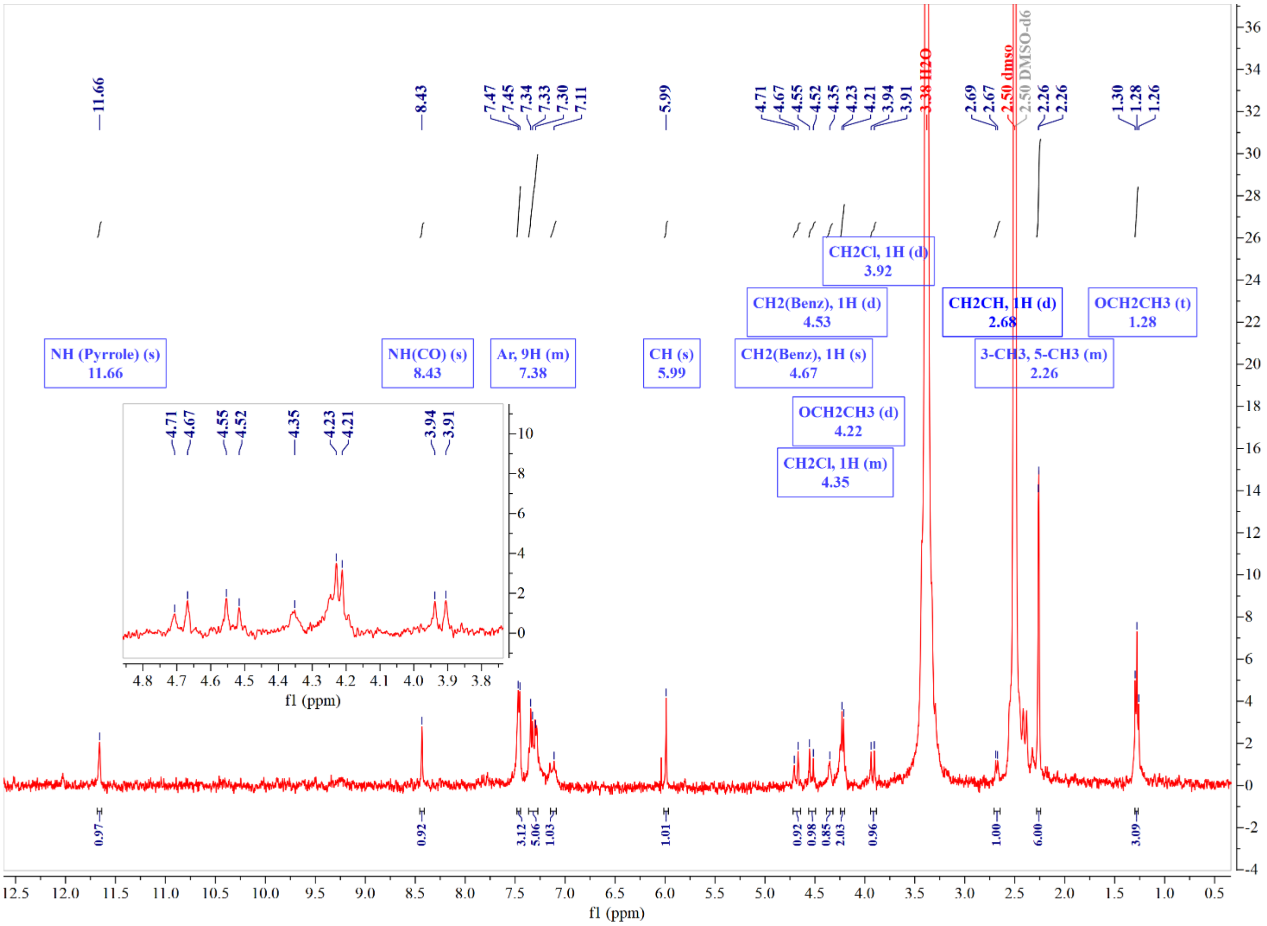

Figure S51. ^{1}H NMR spectrum of compound **12c** in DMSO-d_6.

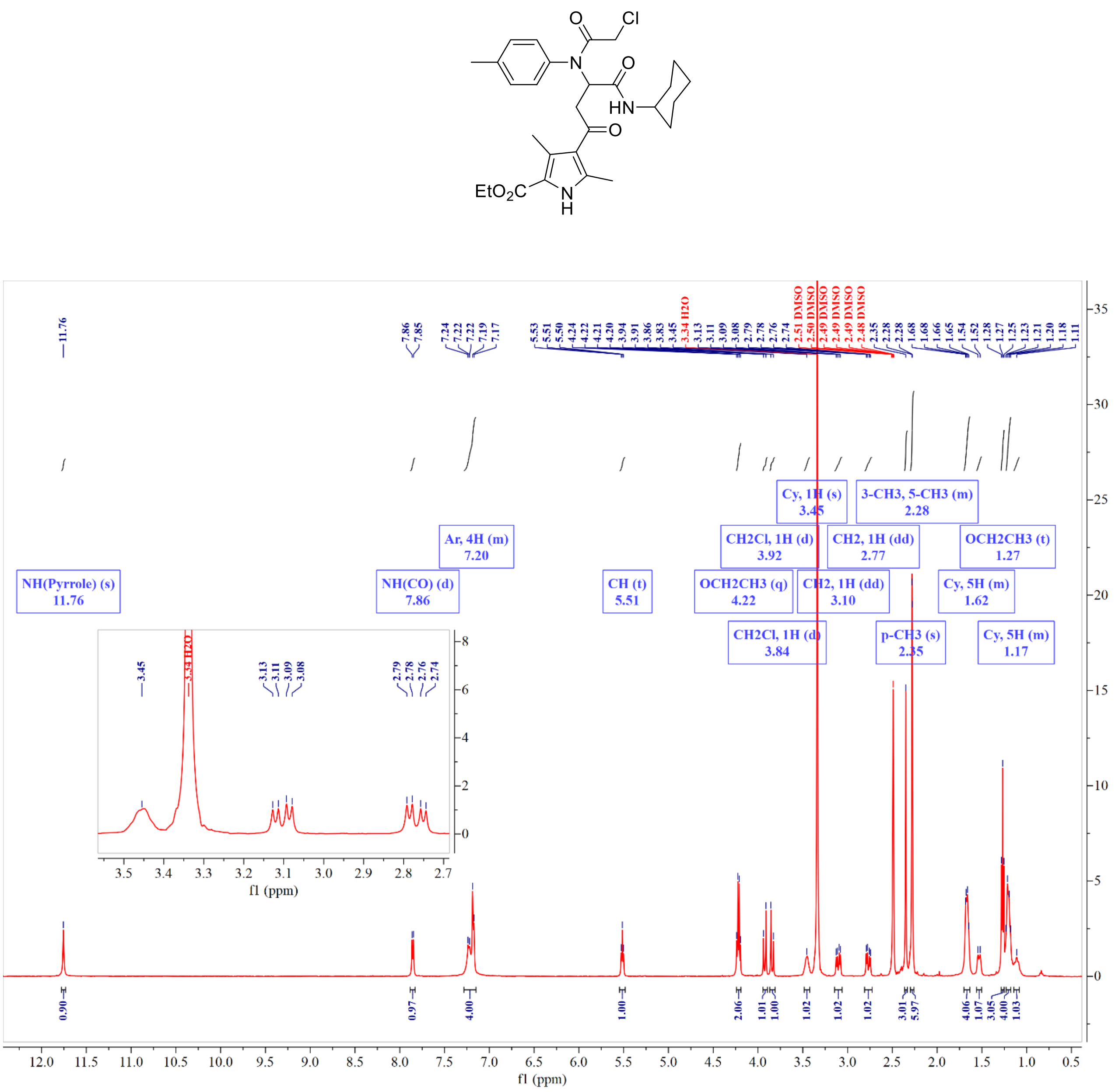

Figure S52. ^{1}H NMR spectrum of compound **12d** in DMSO-d_6.

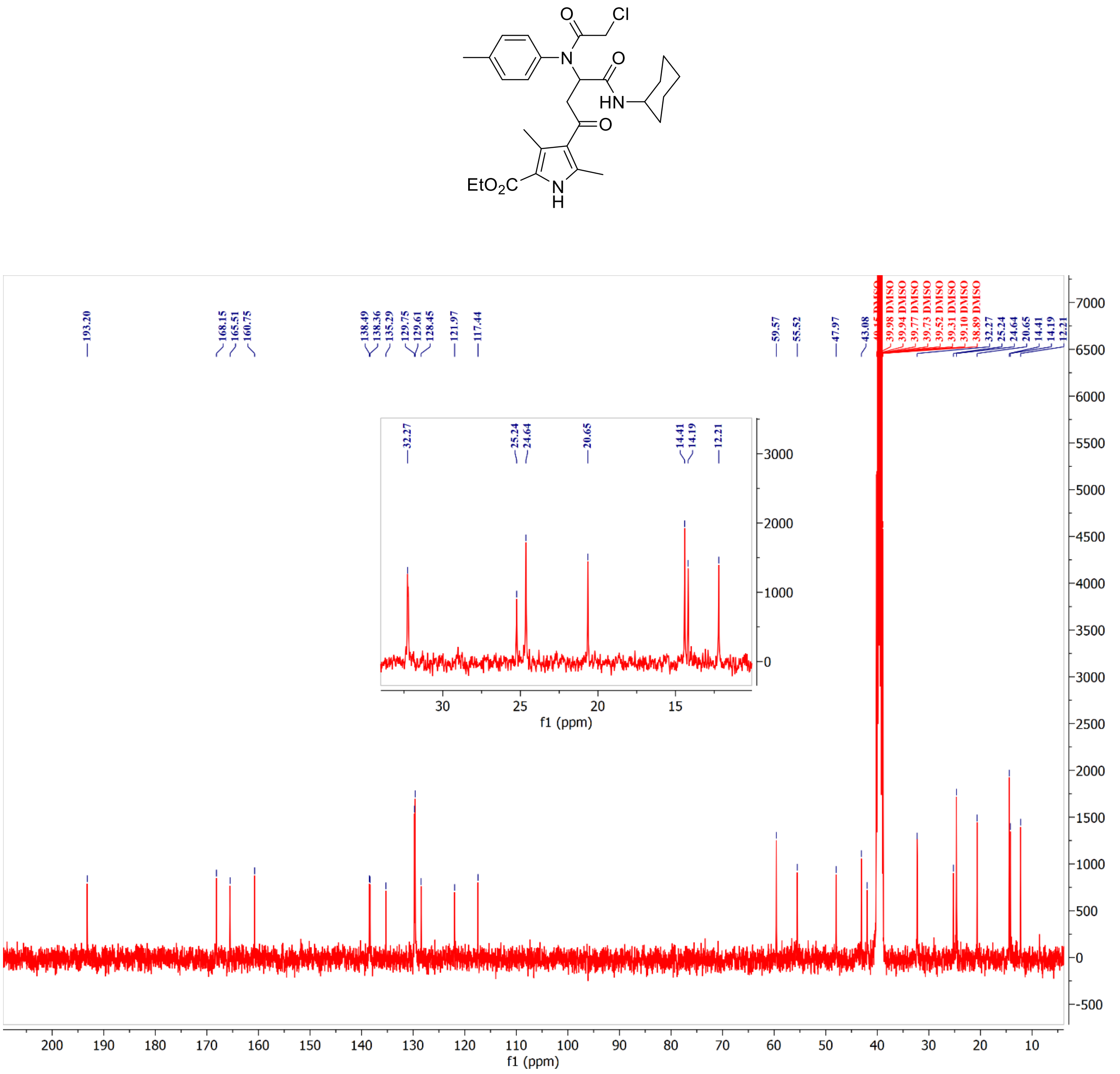

Figure S53. ^{13}C NMR spectrum of compound **12d**

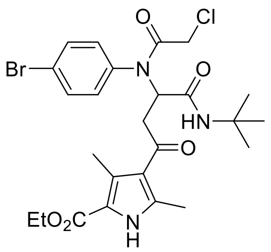

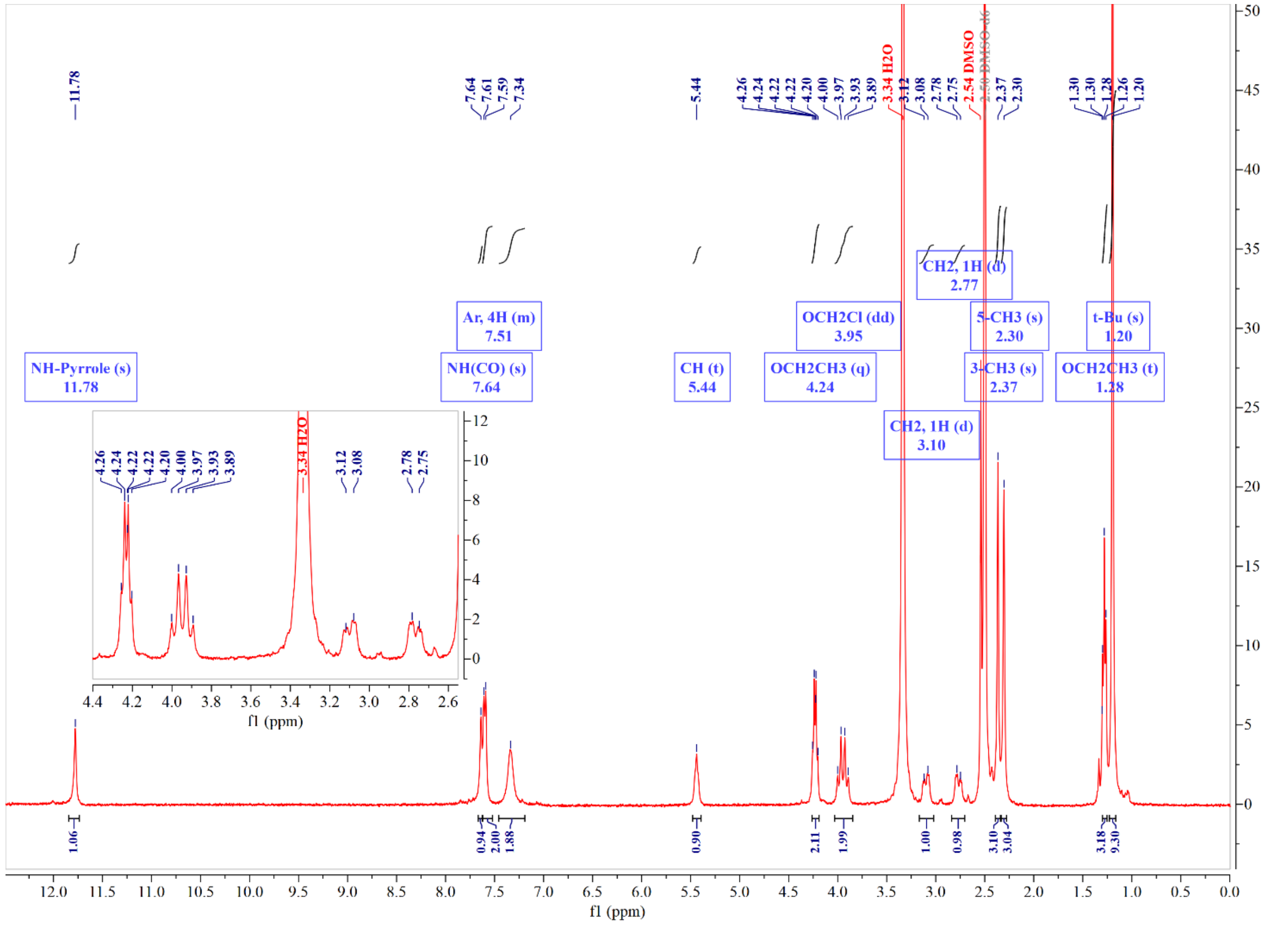

Figure S54. ^{1}H NMR spectrum of compound **12e** in DMSO-d_6.

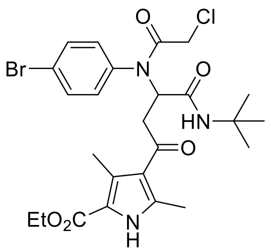

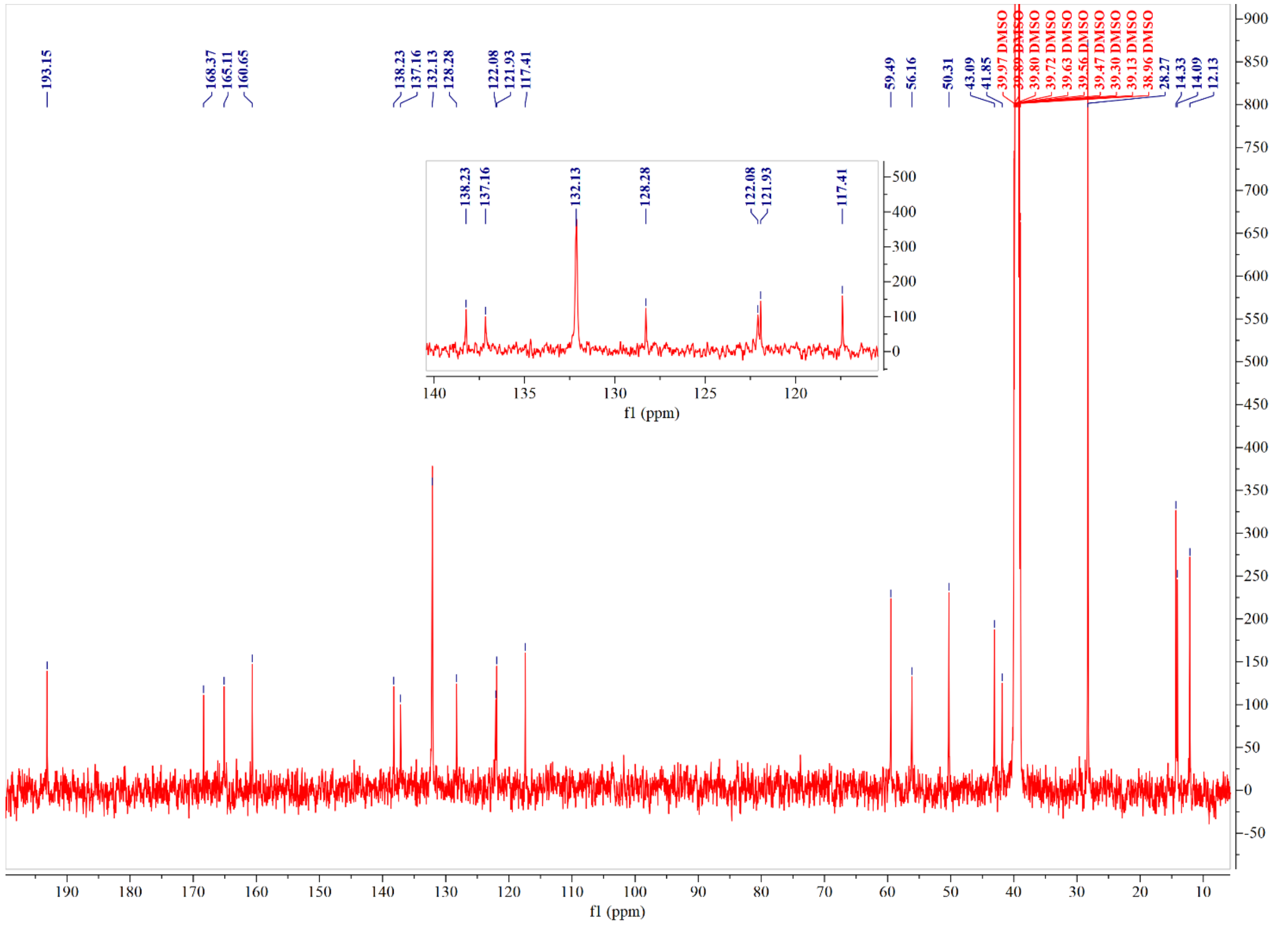

Figure S55. ^{13}C NMR spectrum of compound **12e**

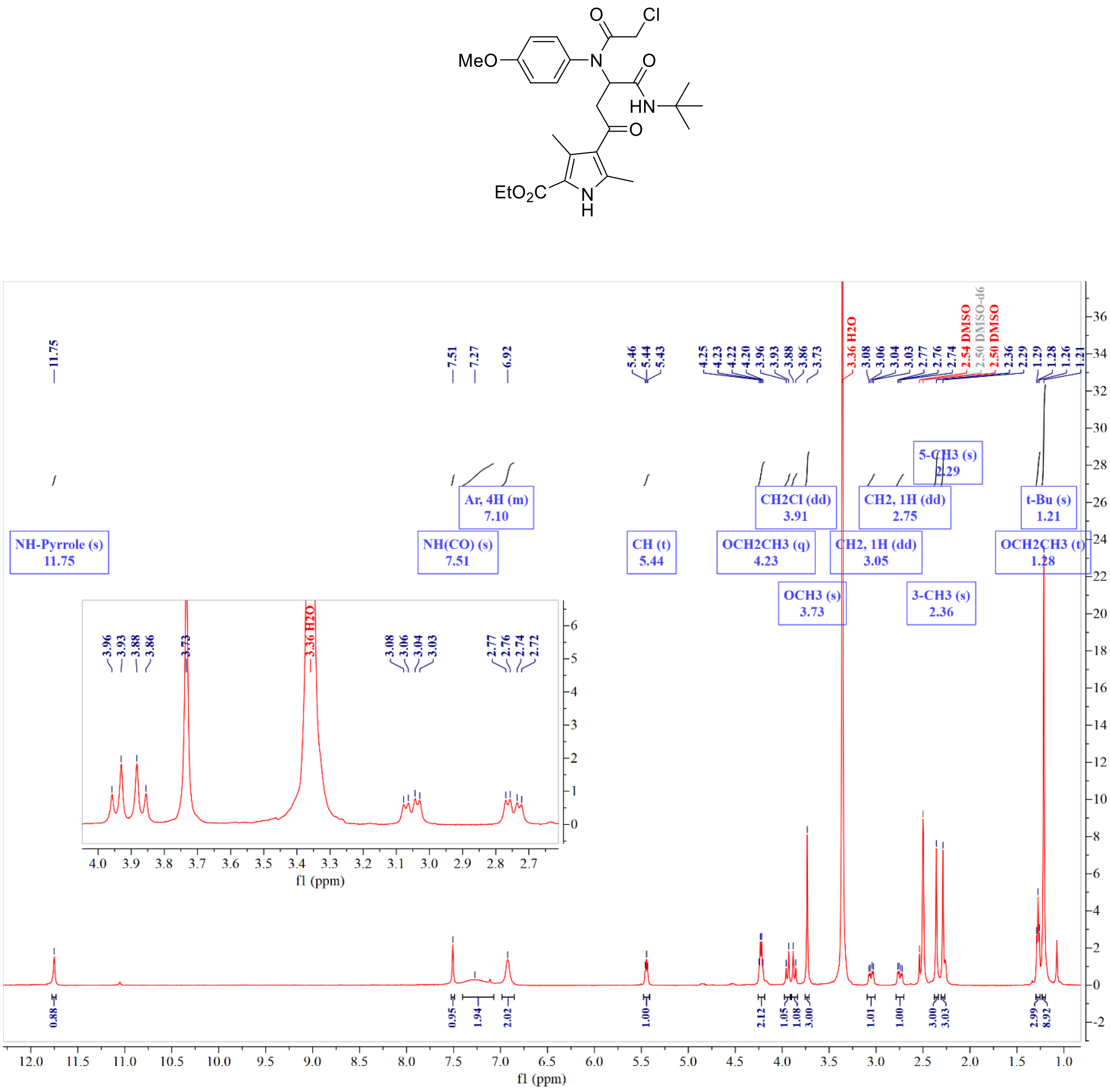

Figure S56. ^{1}H NMR spectrum of compound **12f** in DMSO-d_6.

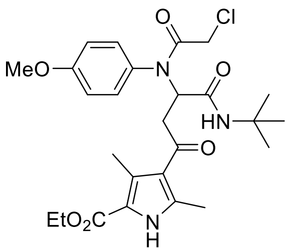

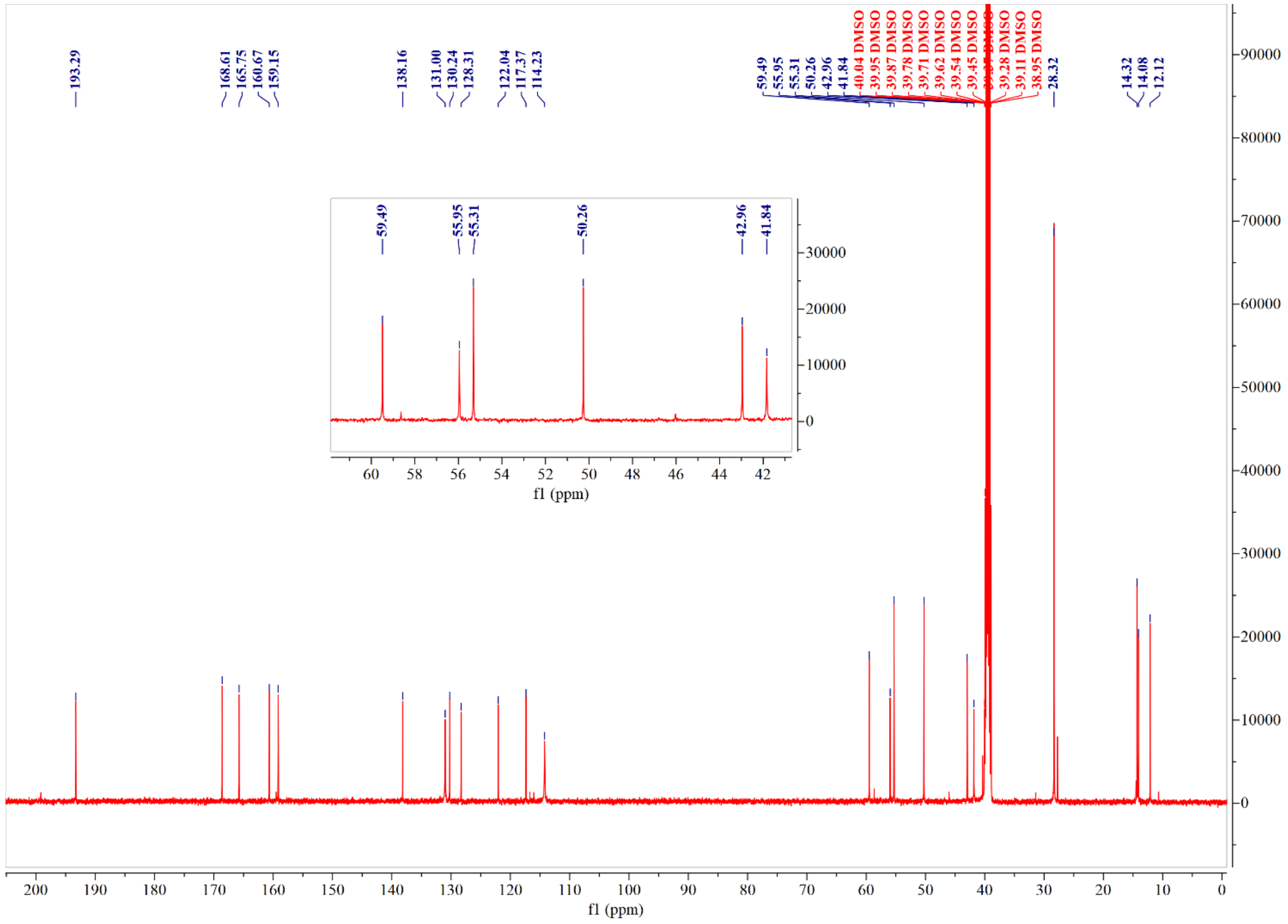

Figure S57. ^{13}C NMR spectrum of compound **12f**

LC–MS spectra of compounds 5–8, 10a–d, and 12a–f

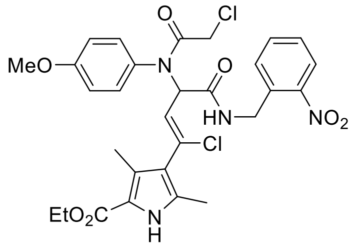

Exact Mass: 616,15
Molecular Weight: 617,48

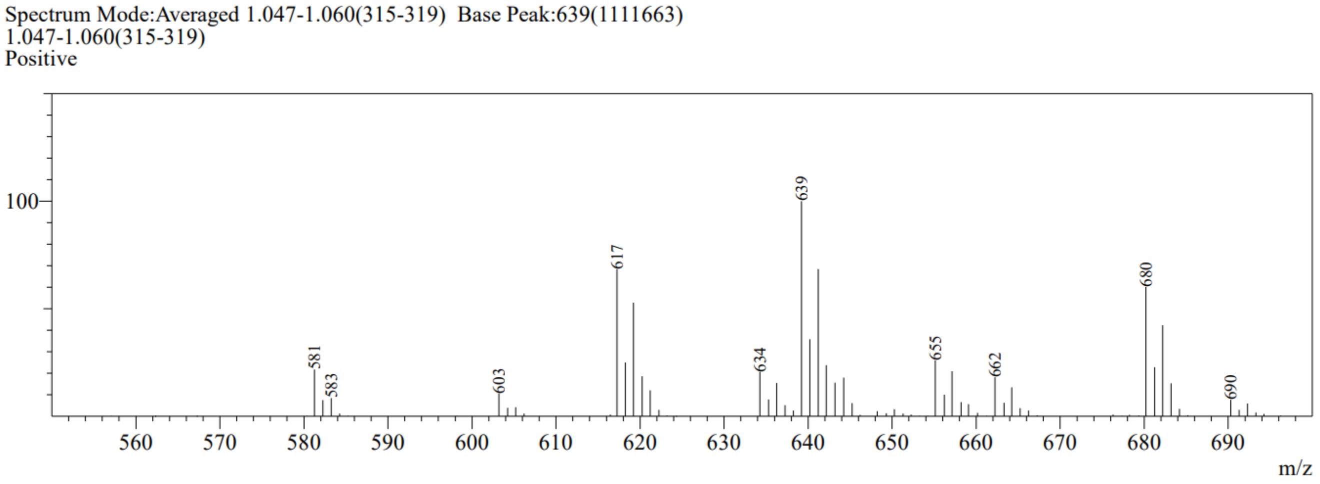

Figure S58. LC–MS spectrum of compound **5a**

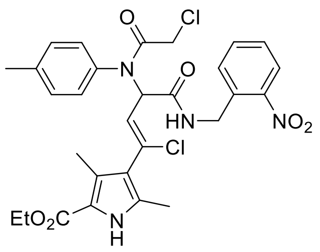

Exact Mass: 600,15
Molecular Weight: 601,48

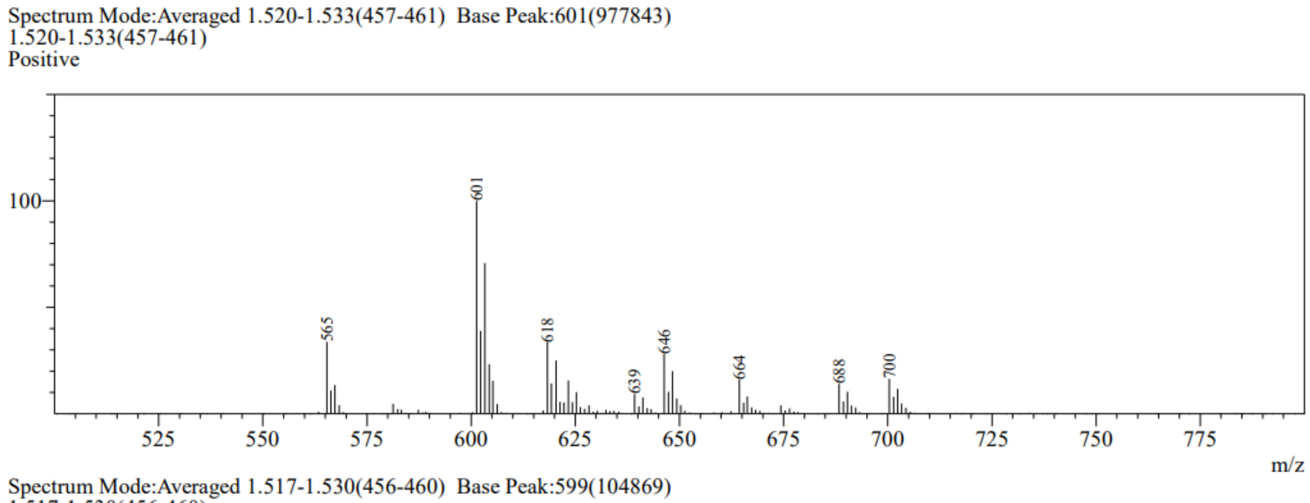

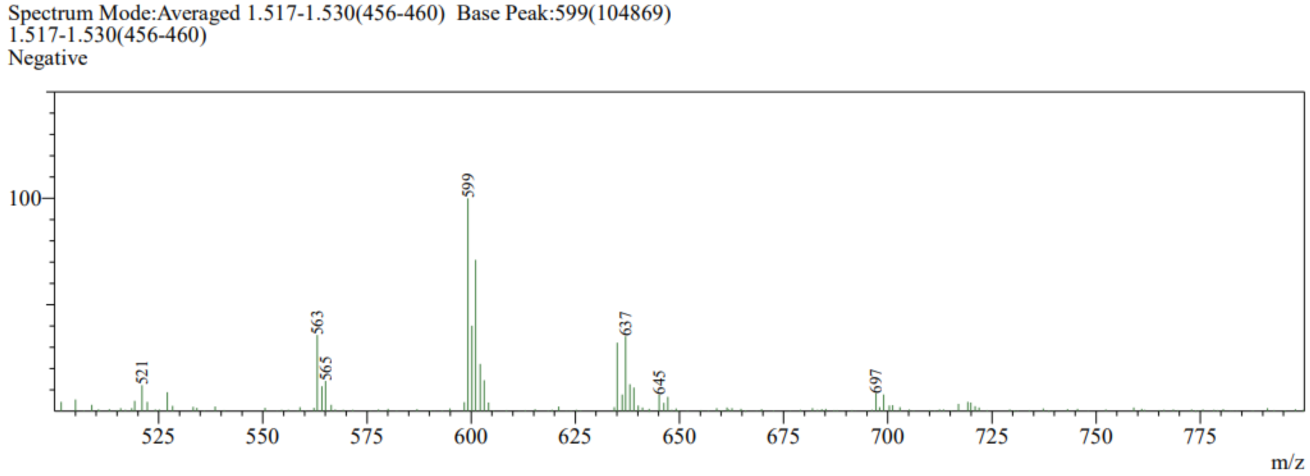

Figure S59. LC–MS spectra of compound **5b**

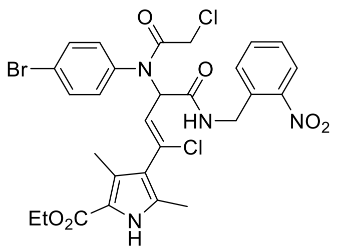

Exact Mass: 664,05
Molecular Weight: 666,35

Spectrum Mode:Averaged 1.573-1.587(473-477) Base Peak:667(249460)
1.573-1.587(473-477)
Positive

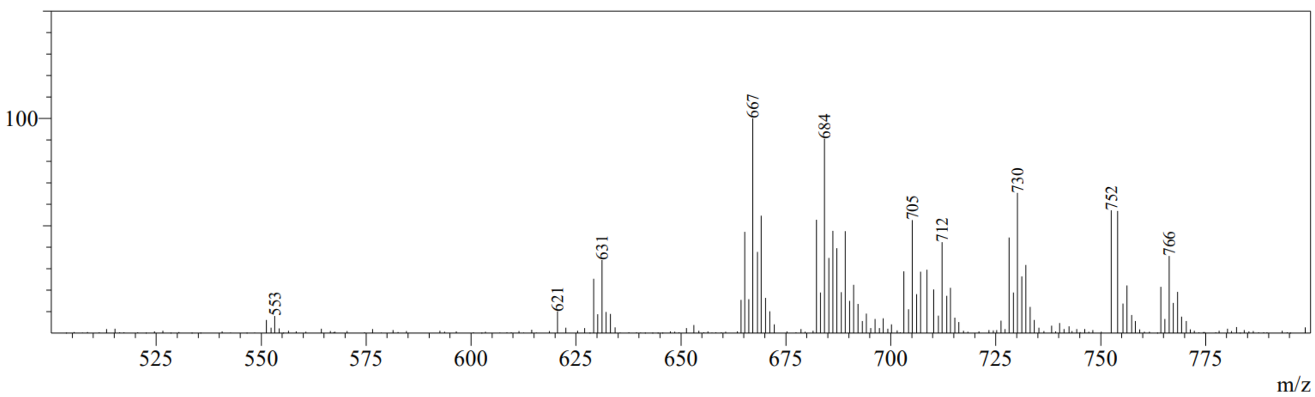

Spectrum Mode:Averaged 1.570-1.583(472-476) Base Peak:665(174944)
1.570-1.583(472-476)
Negative

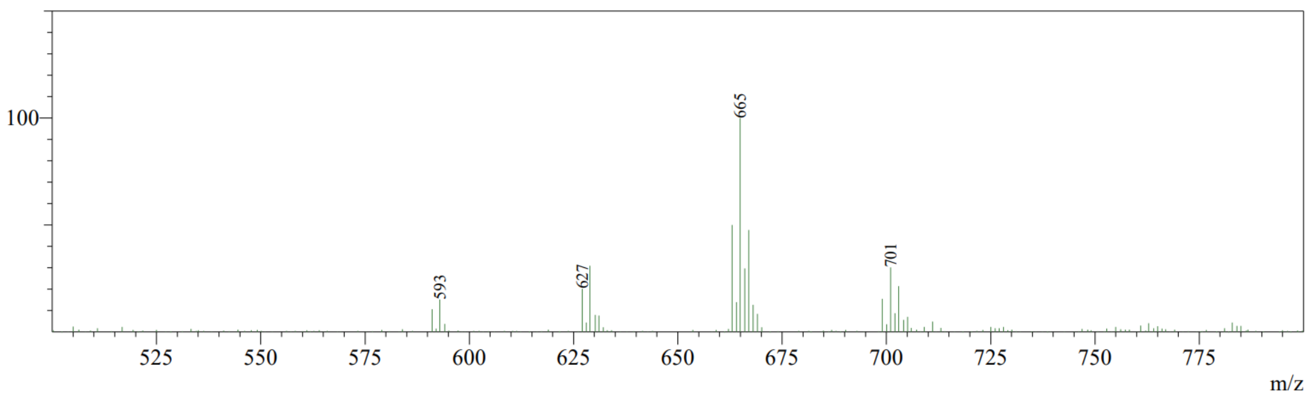

Figure S60. LC–MS spectra of compound **5c**

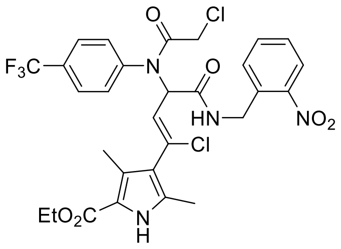

Exact Mass: 600,15
Molecular Weight: 601,48

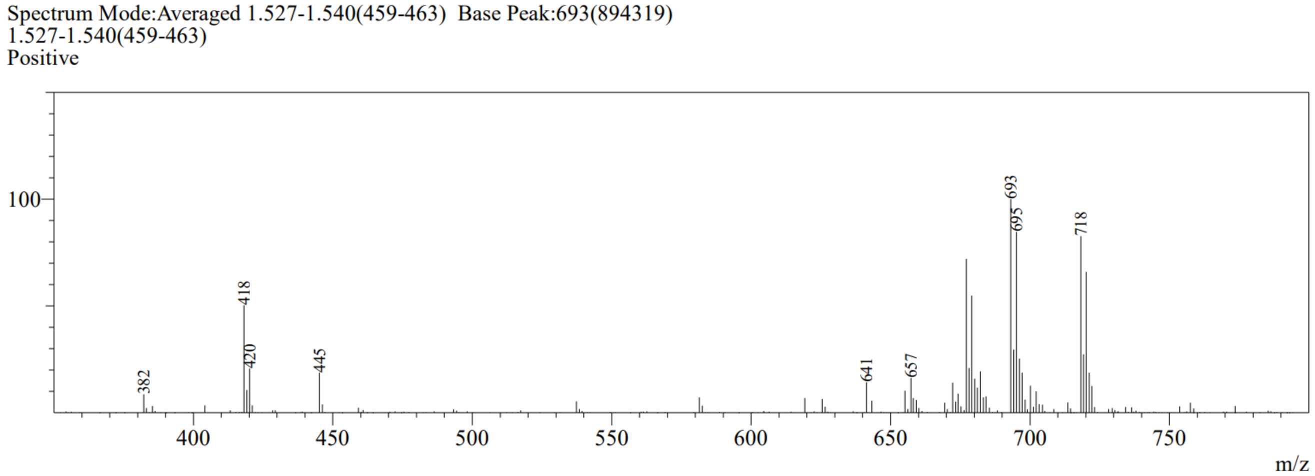

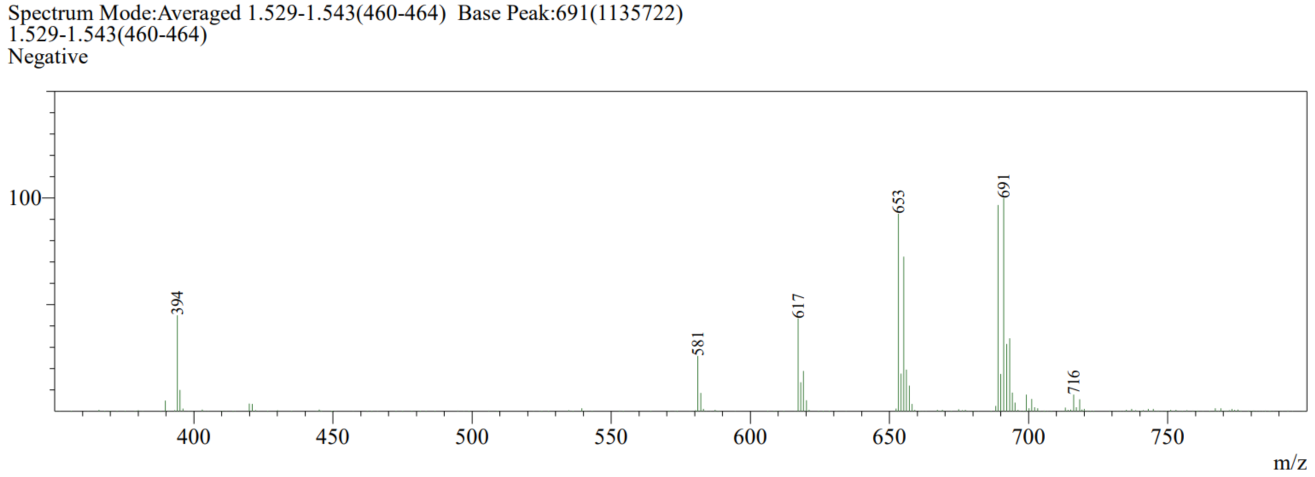

Figure S61. LC–MS spectra of compound **5d**

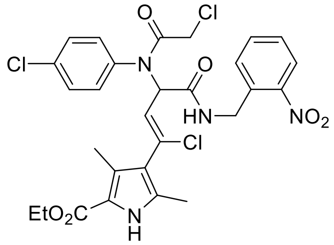

Exact Mass: 620,10
Molecular Weight: 621,90

Spectrum Mode:Averaged 2.620-2.640(525-529) Base Peak:661(1129566)
2.620-2.640(525-529)
Positive

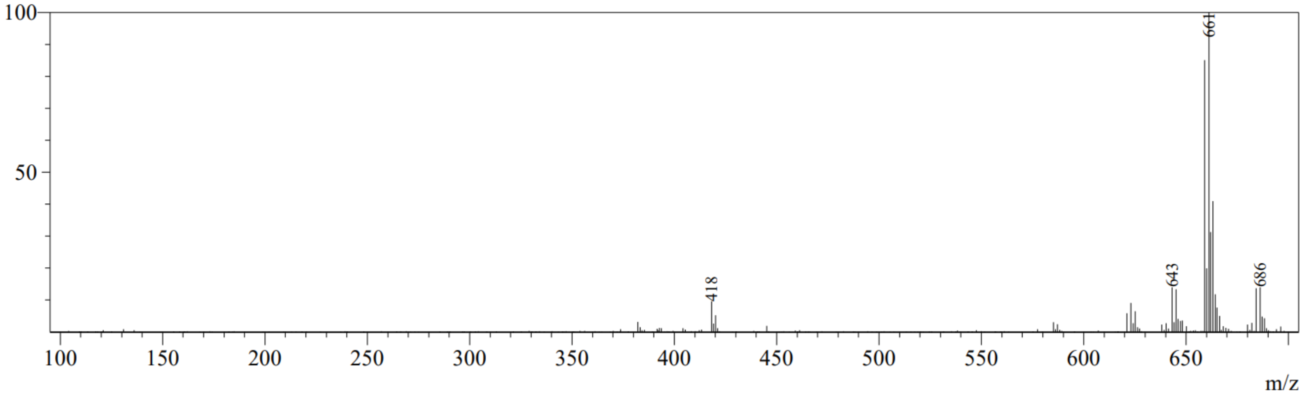

Figure S62. LC–MS spectrum of compound **5e**

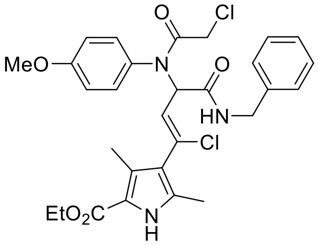

Exact Mass: 571,16
Molecular Weight: 572,48

Line#:1 R.Time:----(Scan#:----)
MassPeaks:115
Spectrum Mode:Averaged 1.690-1.710(339-343) Base Peak:572(3689985)
BG Mode:Calc Segment 1 - Event 1

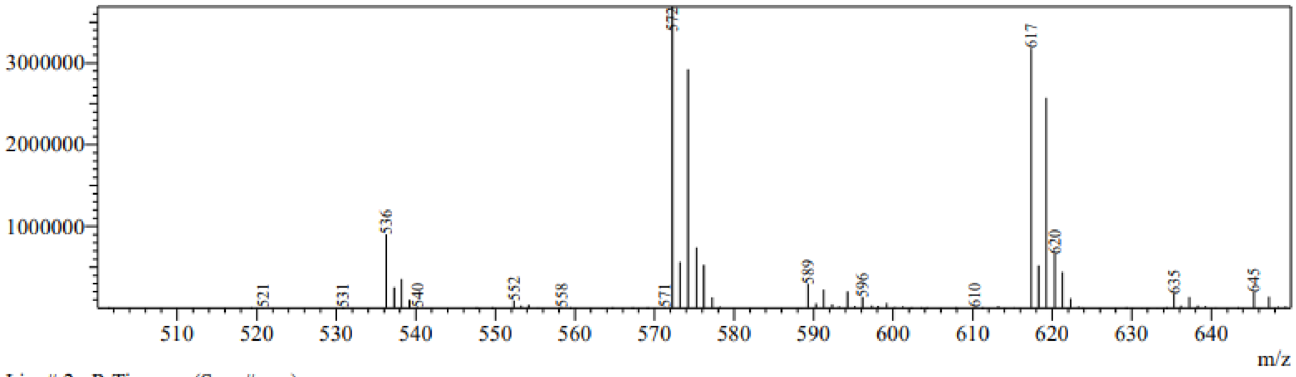

Line#:2 R.Time:----(Scan#:----)
MassPeaks:315
Spectrum Mode:Averaged 1.685-1.705(338-342) Base Peak:519(229852)
BG Mode:Calc Segment 1 - Event 2

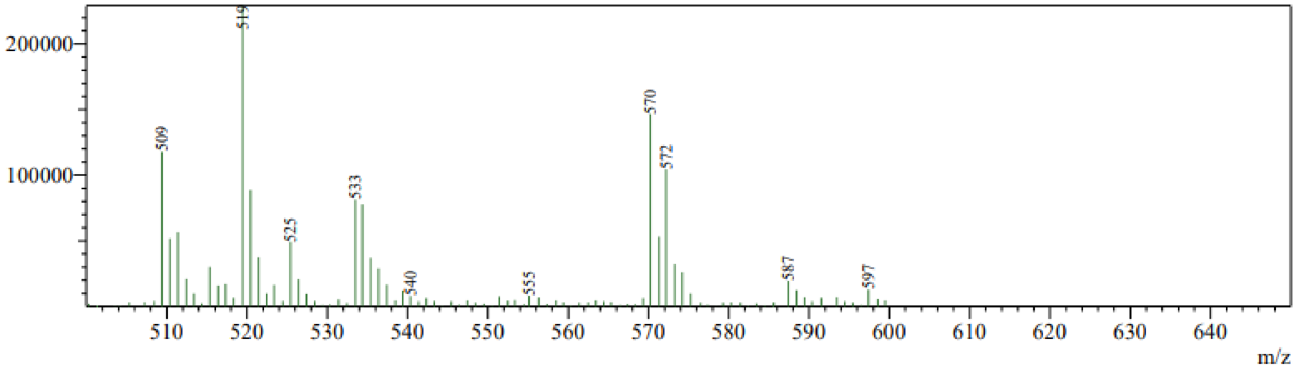

Figure S63. LC–MS spectra of compound **6a**

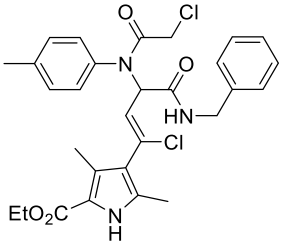

Exact Mass: 555,17
Molecular Weight: 556,48

Spectrum Mode:Averaged 2.390-2.410(479-483) Base Peak:556(4527706)
BG Mode:Calc Segment 1 - Event 1

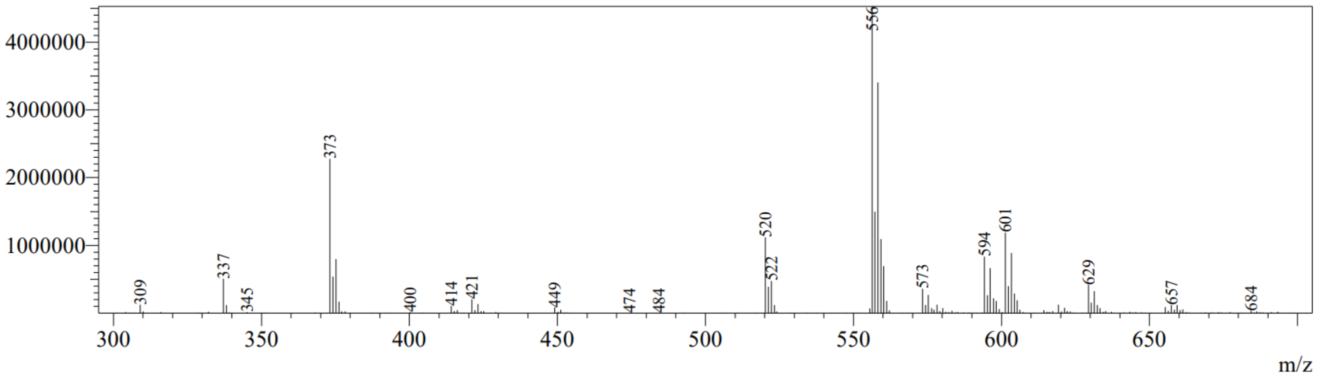

Spectrum Mode:Averaged 2.395-2.415(480-484) Base Peak:335(104895)
BG Mode:Calc Segment 1 - Event 2

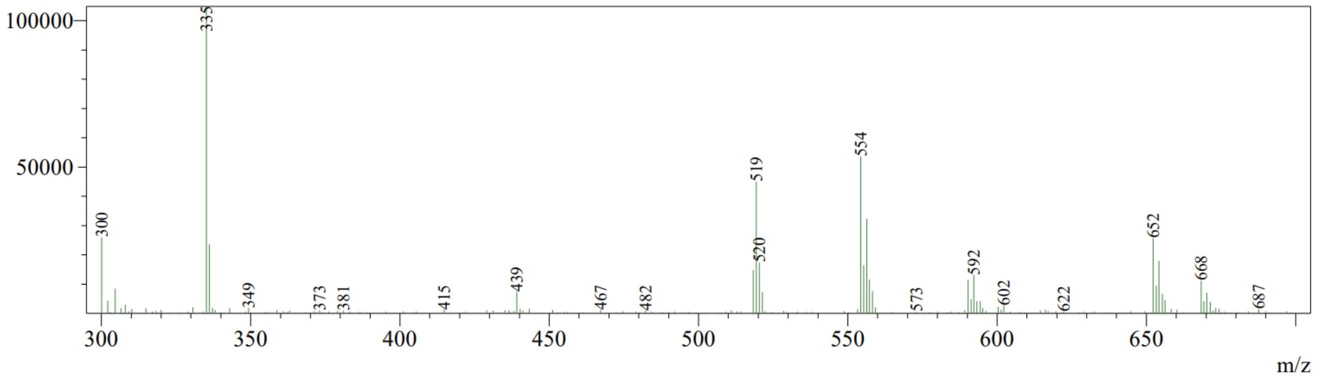

Figure S64. LC–MS spectra of compound **6b**

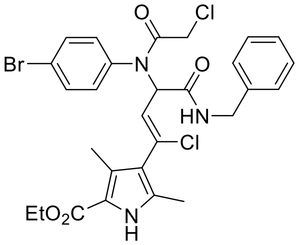

Exact Mass: 619,06
Molecular Weight: 621,35

Spectrum Mode:Averaged 2.220-2.240(445-449) Base Peak:667(25959)
2.220-2.240(445-449)
Positive

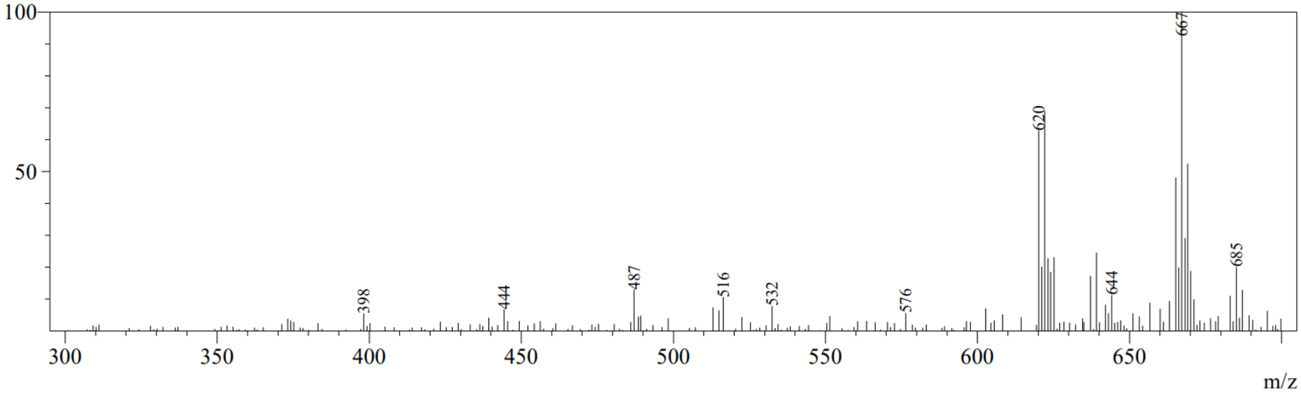

Spectrum Mode:Averaged 2.025-2.045(406-410) Base Peak:656(596910)
2.025-2.045(406-410)
Negative

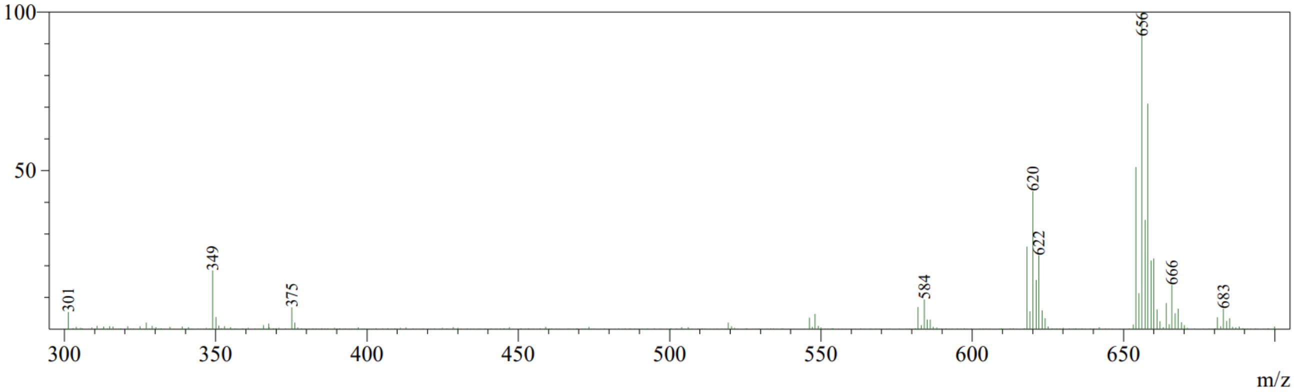

Figure S65. LC–MS spectra of compound **6c**

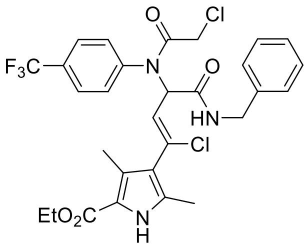

Exact Mass: 609,14
Molecular Weight: 610,46

Spectrum Mode:Averaged 2.440-2.460(489-493) Base Peak:373(1087913)
BG Mode:Calc Segment 1 - Event 1

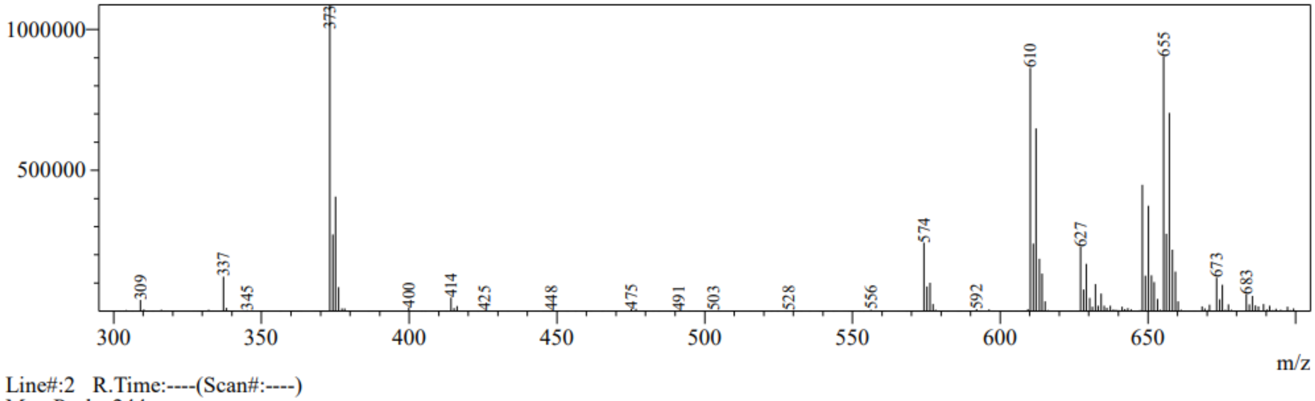

Line#:2 R.Time:----(Scan#:----)
MassPeaks:244
Spectrum Mode:Averaged 2.445-2.465(490-494) Base Peak:608(72371)
BG Mode:Calc Segment 1 - Event 2

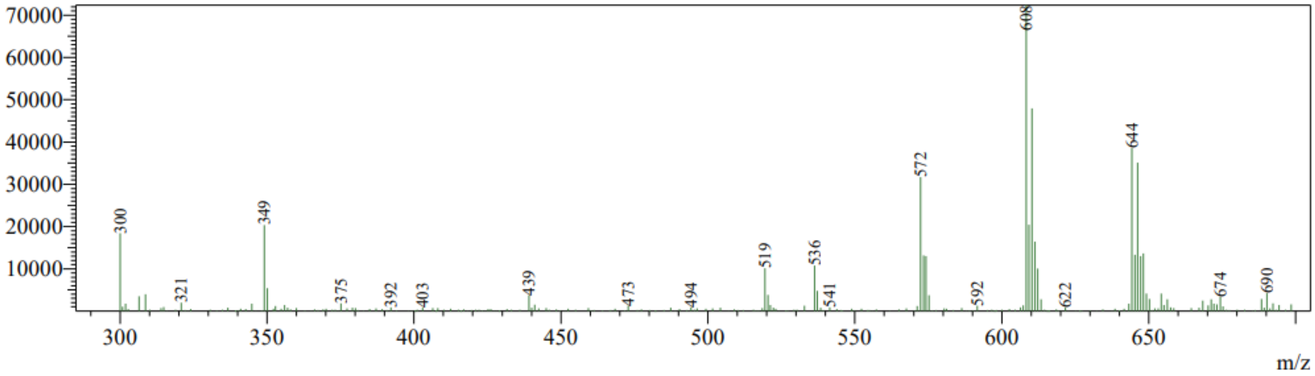

Figure S66. LC–MS spectra of compound **6d**

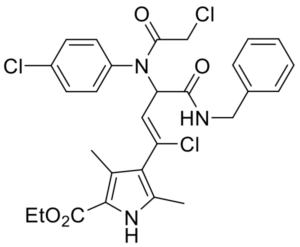

Exact Mass: 575,11
Molecular Weight: 576,90

Spectrum Mode:Averaged 2.970-2.990(595-599) Base Peak:373(1246562)
2.970-2.990(595-599)
Positive

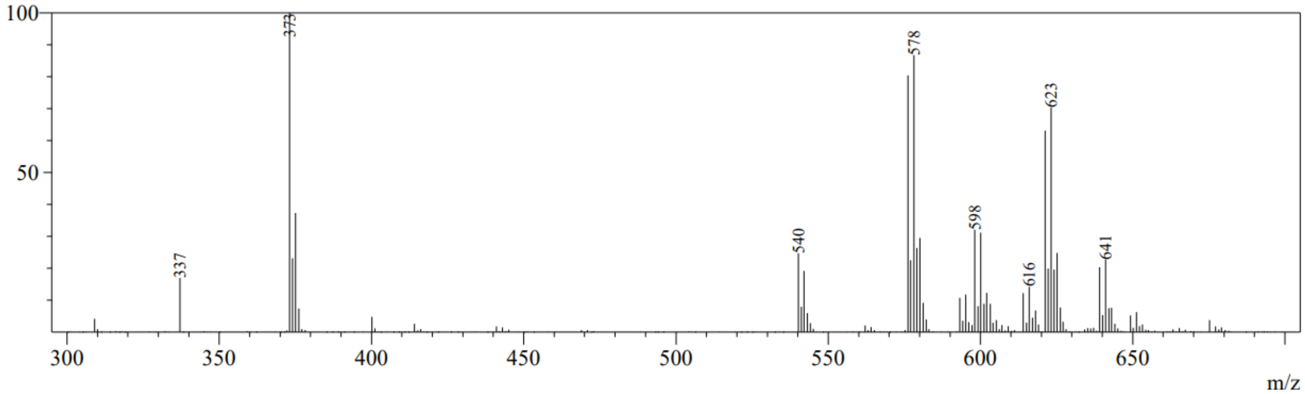

Spectrum Mode:Averaged 2.975-2.995(596-600) Base Peak:612(103027)
2.975-2.995(596-600)
Negative

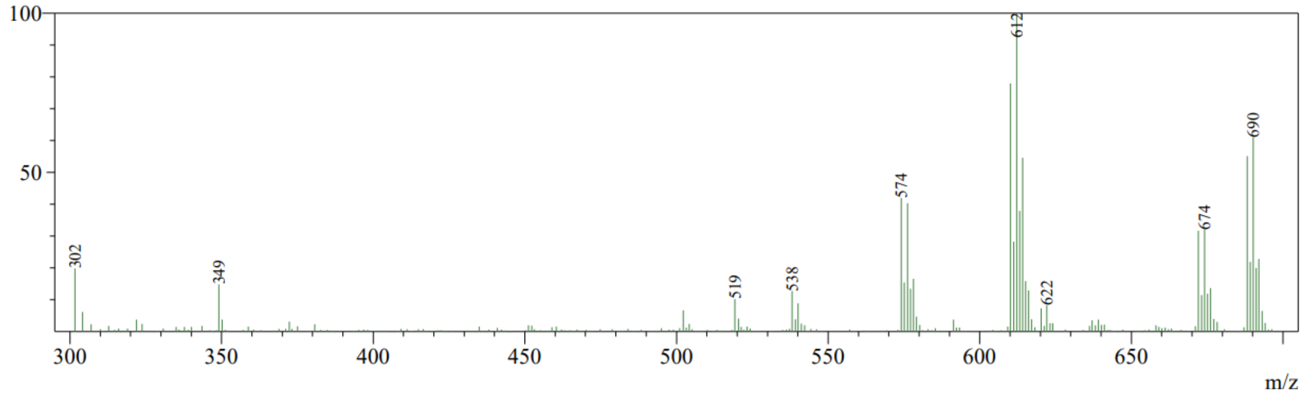

Figure S67. LC–MS spectra of compound **6e**

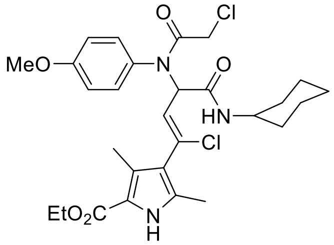

Exact Mass: 563,20
Molecular Weight: 564,50

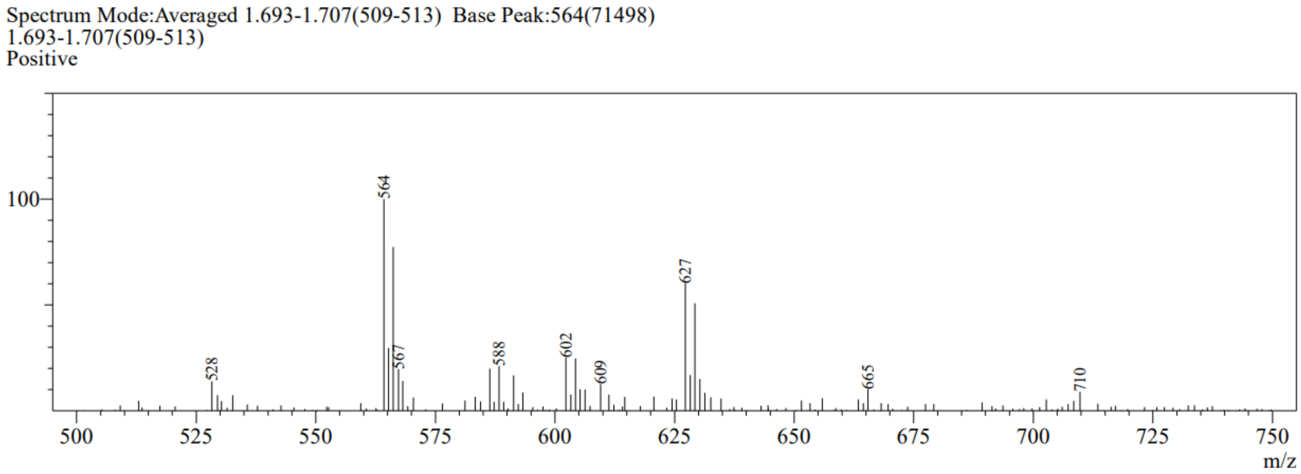

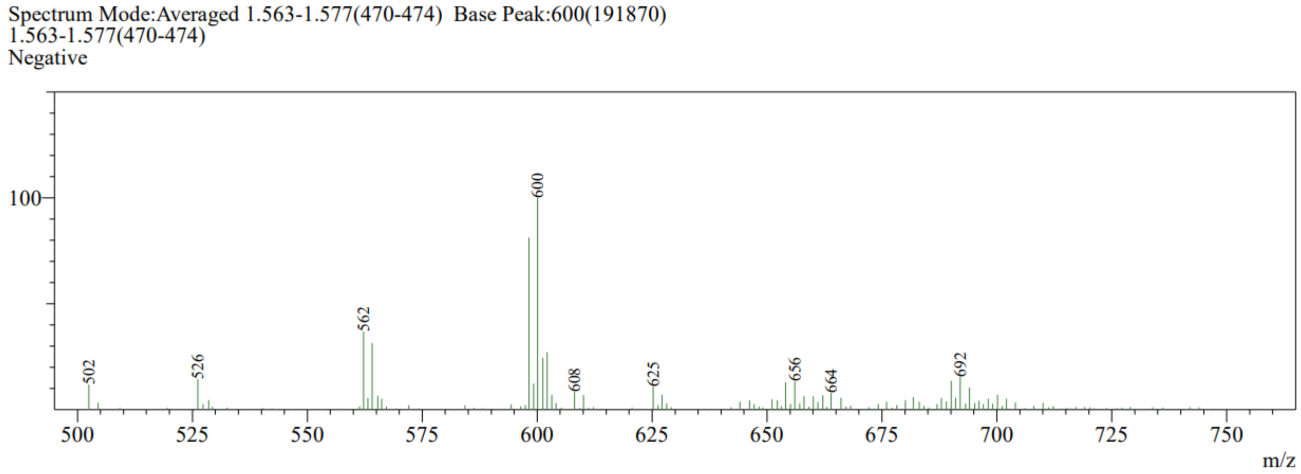

Figure S68. LC–MS spectra of compound **7a**

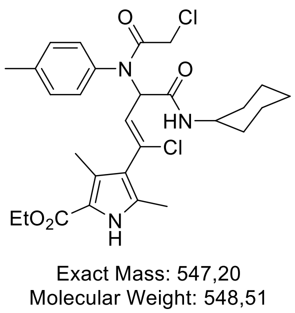

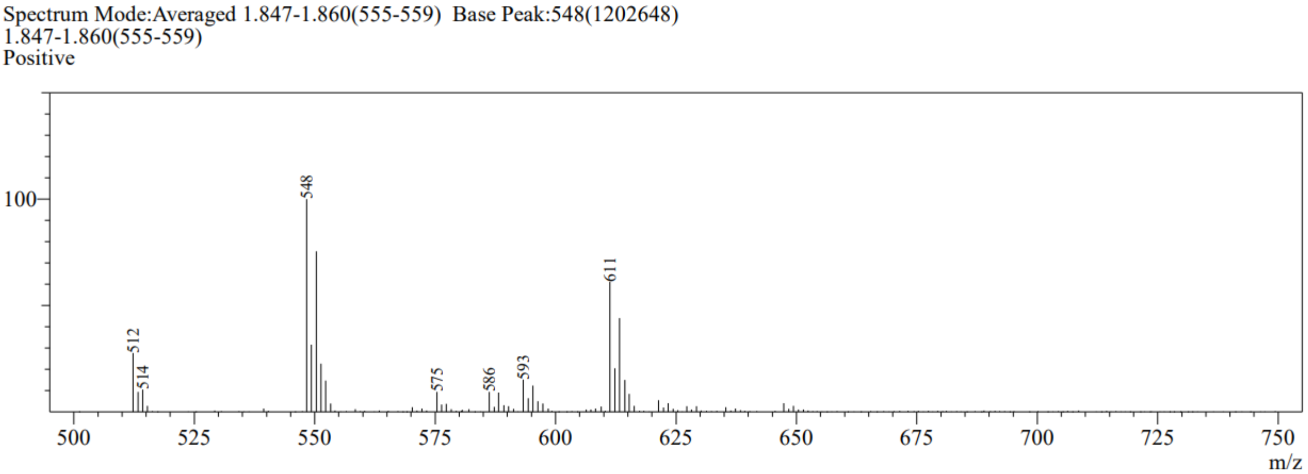

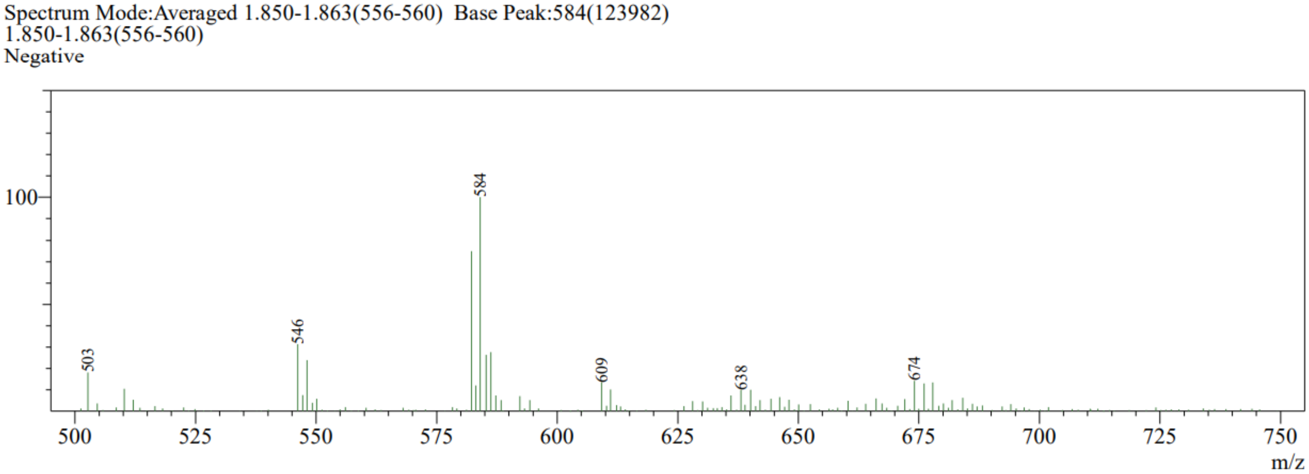

Figure S69. LC–MS spectra of compound **7b**

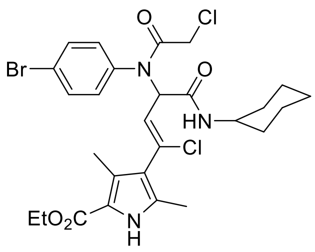

Exact Mass: 611,10
Molecular Weight: 613,37

Spectrum Mode:Averaged 1.373-1.387(413-417) Base Peak:677(737642)
1.373-1.387(413-417)
Positive

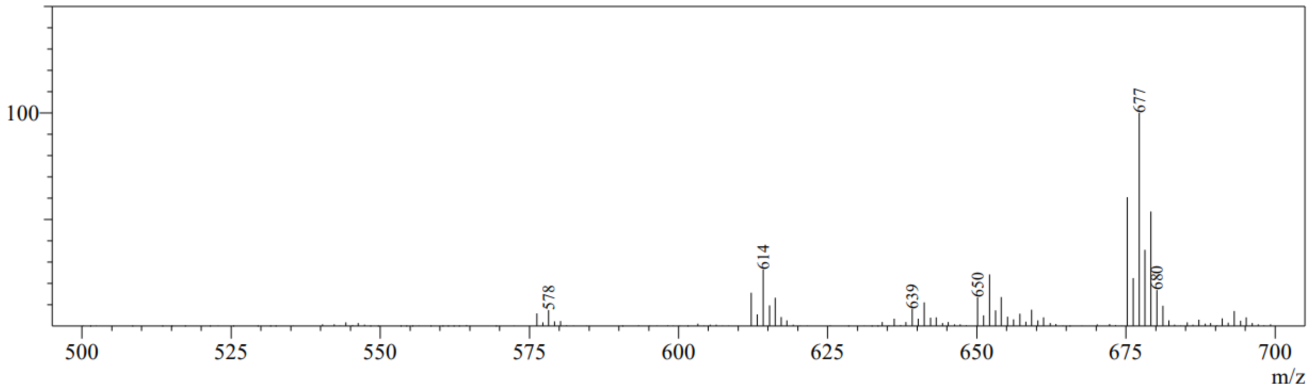

Figure S70. LC–MS spectrum of compound **7c**

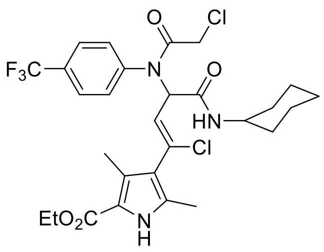

Exact Mass: 601,17
Molecular Weight: 602,48

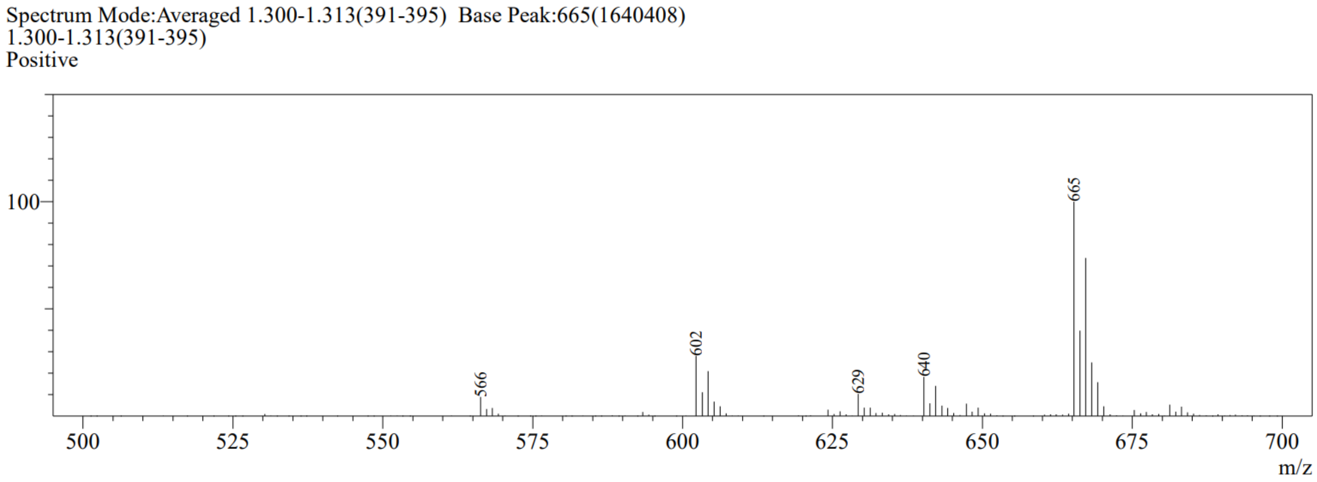

Figure S71. LC–MS spectrum of compound **7d**

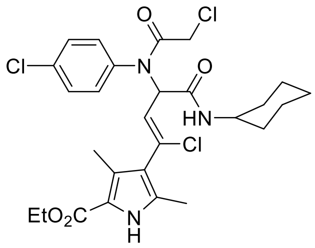

Exact Mass: 567,15
Molecular Weight: 568,92

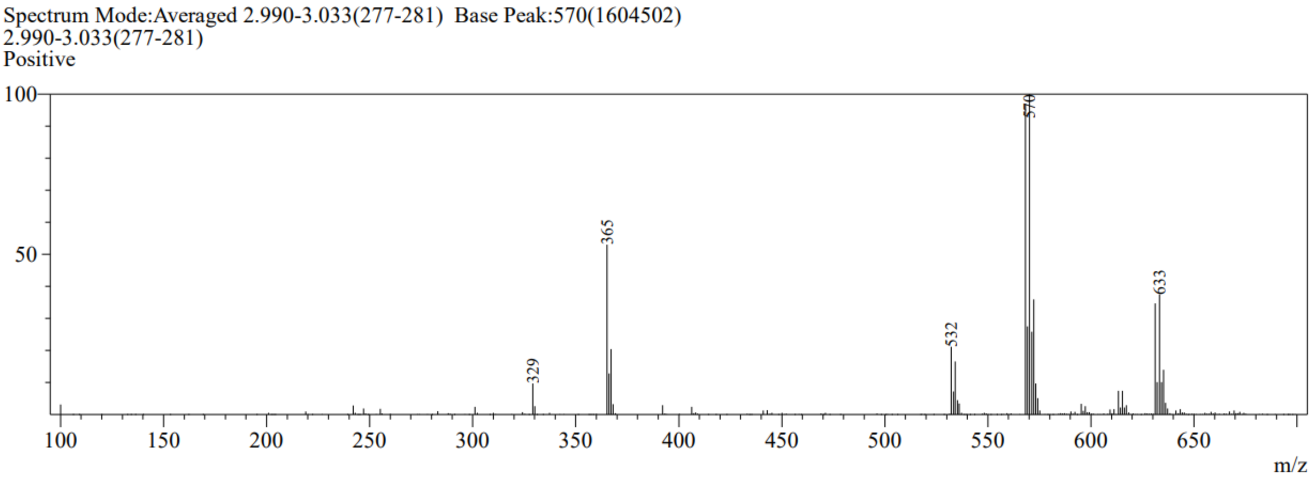

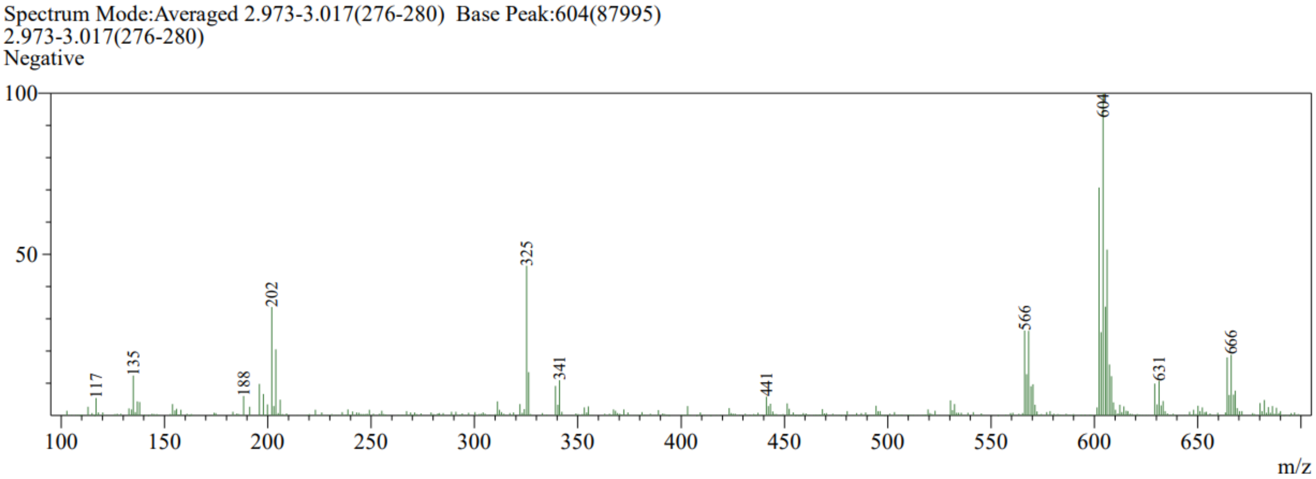

Figure S72. LC–MS spectra of compound **7e**

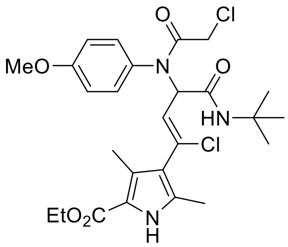

Exact Mass: 537,18
Molecular Weight: 538,47

Spectrum Mode:Averaged 1.140-1.153(343-347) Base Peak:538(4440576)
1.140-1.153(343-347)
Positive

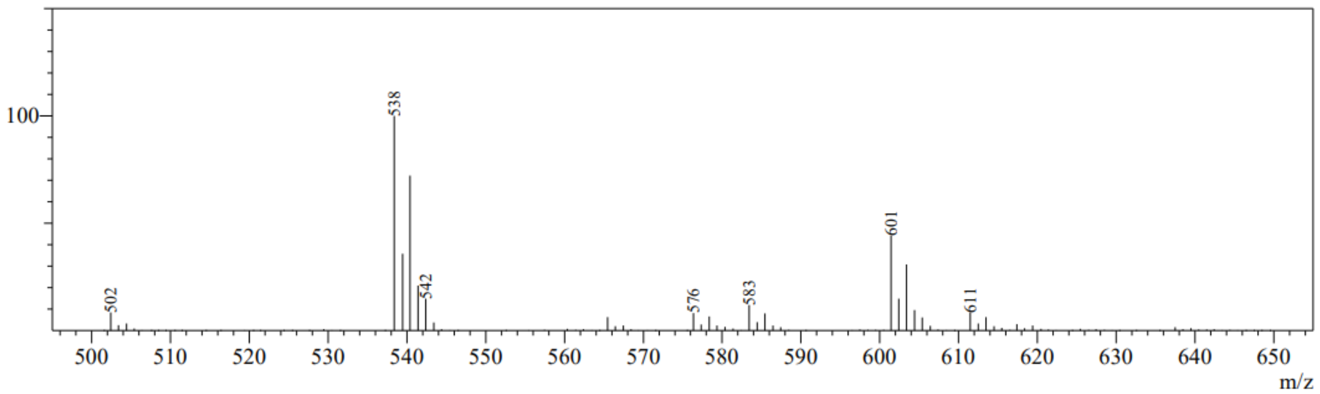

Spectrum Mode:Averaged 1.143-1.157(344-348) Base Peak:536(799879)
1.143-1.157(344-348)
Negative

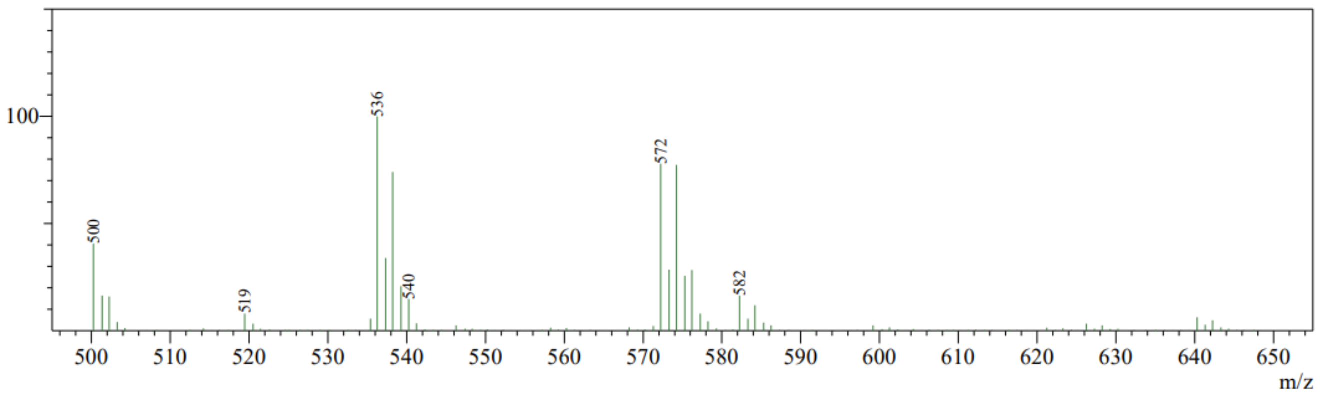

Figure S73. LC–MS spectra of compound **8a**

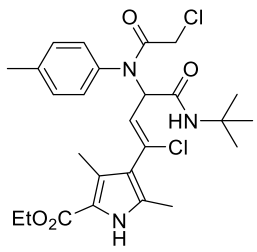

Exact Mass: 521,18
Molecular Weight: 522,47

Spectrum Mode:Averaged 1.700-1.713(511-515) Base Peak:522(2181090)
1.700-1.713(511-515)
Positive

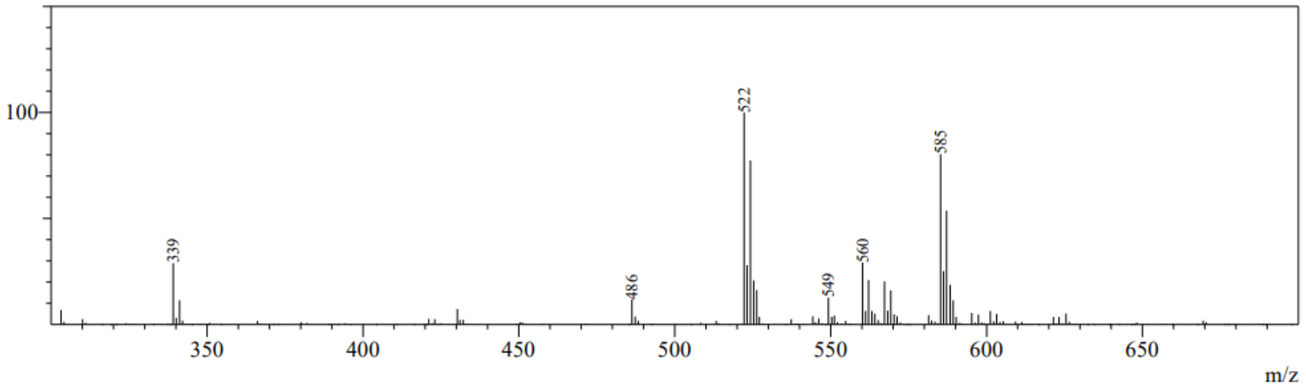

Spectrum Mode:Averaged 1.703-1.716(512-516) Base Peak:520(678475)
1.703-1.716(512-516)
Negative

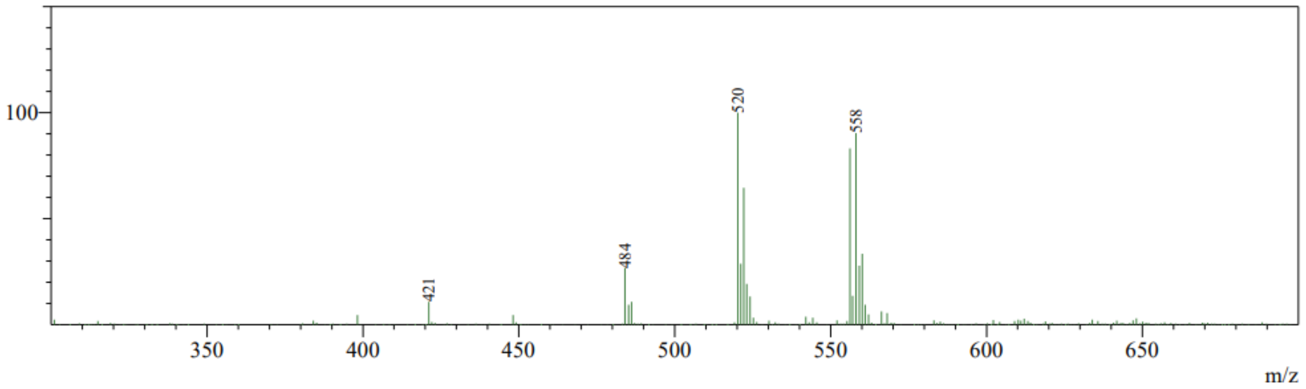

Figure S74. LC–MS spectra of compound **8b**

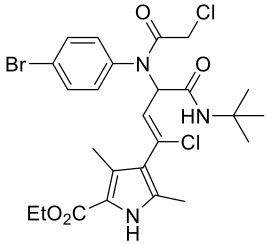

Exact Mass: 585,08
Molecular Weight: 587,34

Spectrum Mode:Averaged 2.250-2.270(451-455) Base Peak:588(359927)
BG Mode:Calc Segment 1 - Event 1

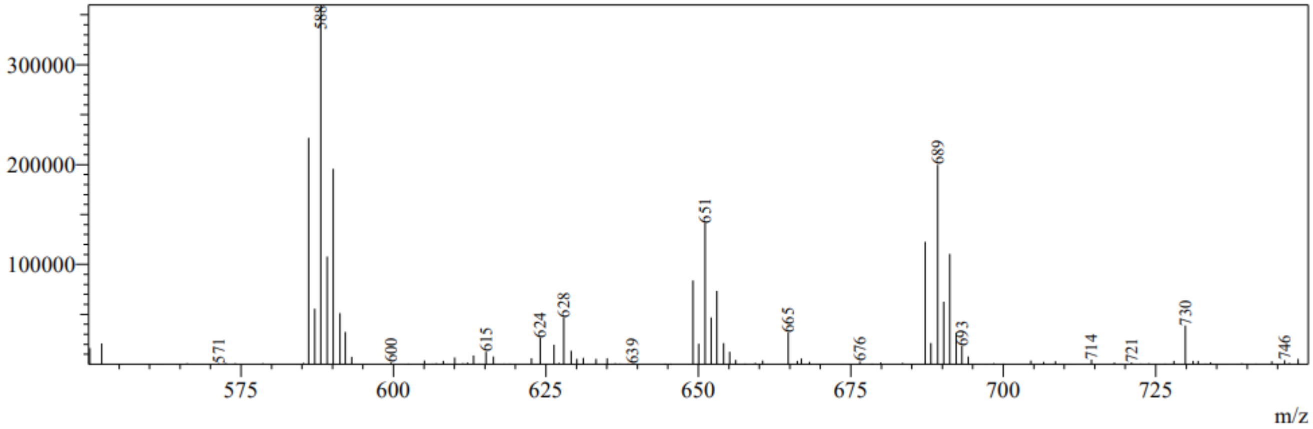

Line#:2 R.Time:----(Scan#:----)
MassPeaks:361
Spectrum Mode:Averaged 2.255-2.275(452-456) Base Peak:622(472819)
BG Mode:Calc Segment 1 - Event 2

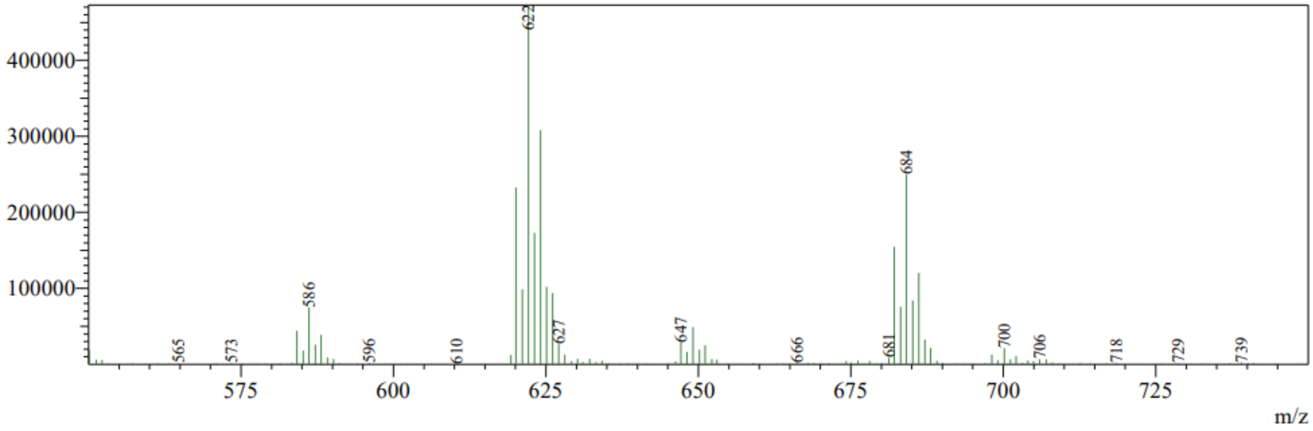

Figure S75. LC–MS spectra of compound **8c**

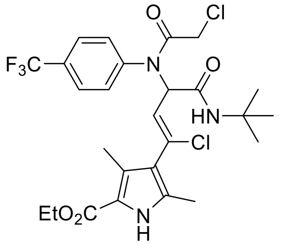

Exact Mass: 575,16
Molecular Weight: 576,44

Spectrum Mode:Averaged 1.740-1.753(523-527) Base Peak:639(2089127)
1.740-1.753(523-527)
Positive

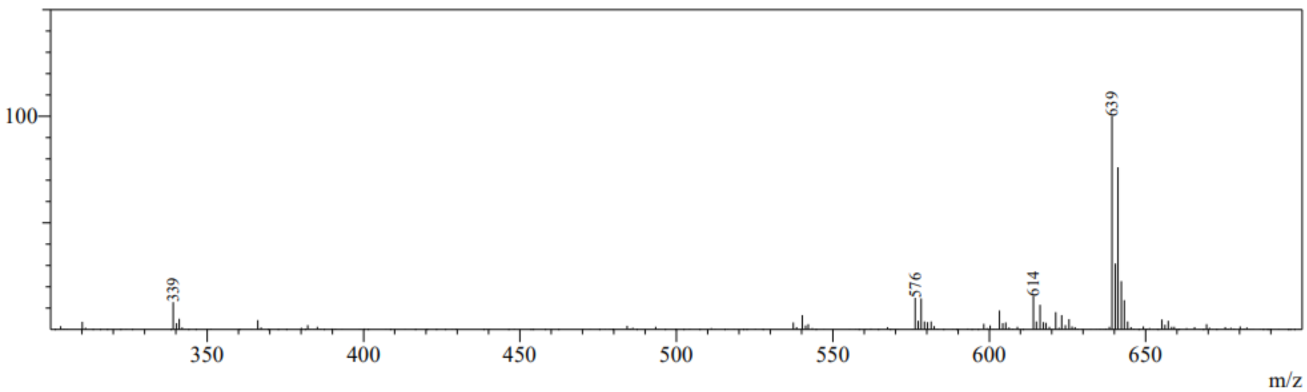

Spectrum Mode:Averaged 1.736-1.749(522-526) Base Peak:612(1417953)
1.736-1.749(522-526)
Negative

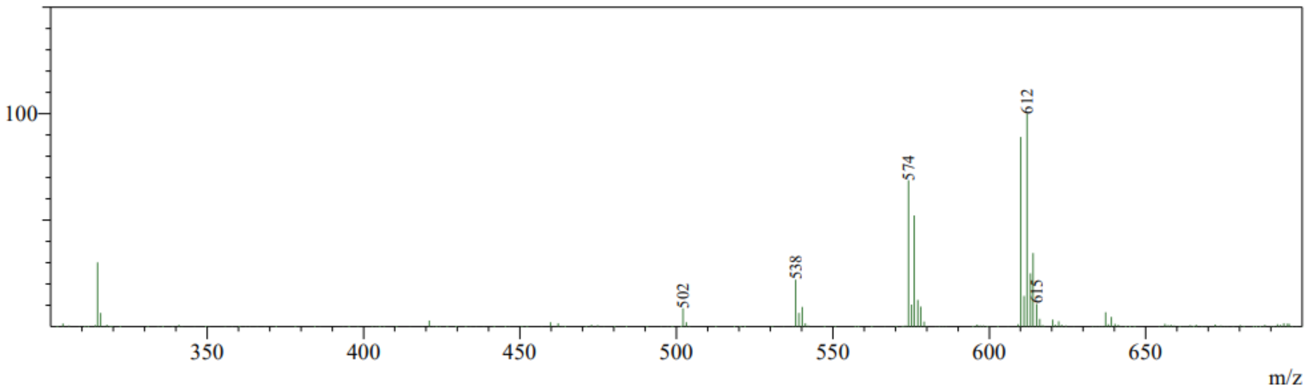

Figure S76. LC–MS spectra of compound **8d**

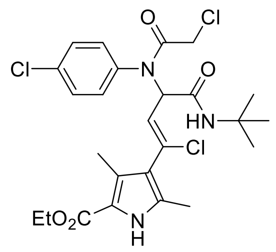

Exact Mass: 541,13
Molecular Weight: 542,88

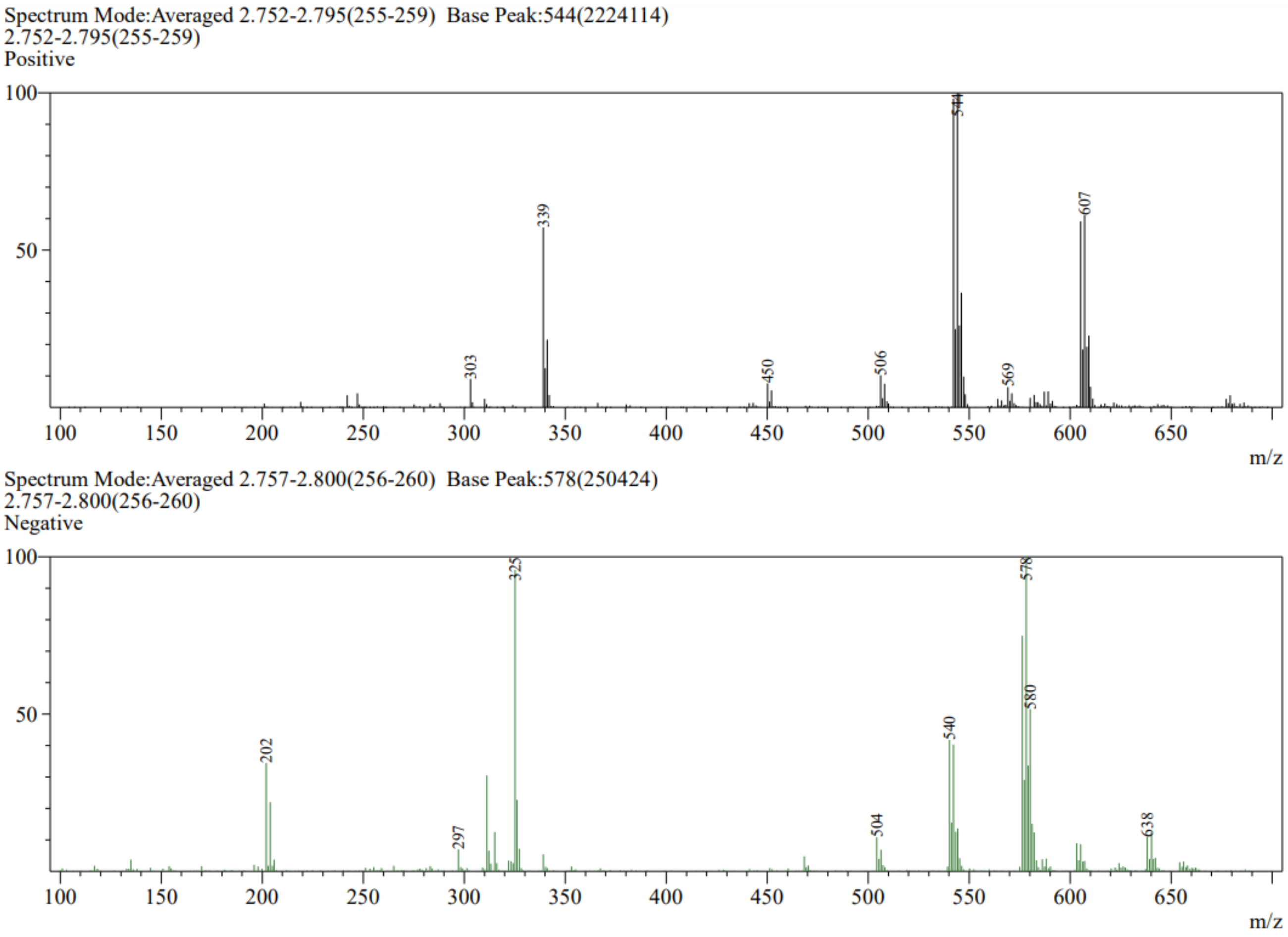

Figure S77. LC–MS spectra of compound **8e**

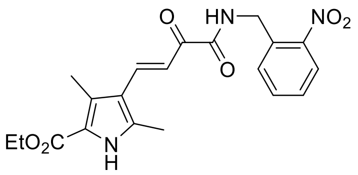

Exact Mass: 399,14
Molecular Weight: 399,40

Spectrum Mode:Averaged 1.160-1.173(349-353) Base Peak:463(1638972)
1.160-1.173(349-353)
Positive

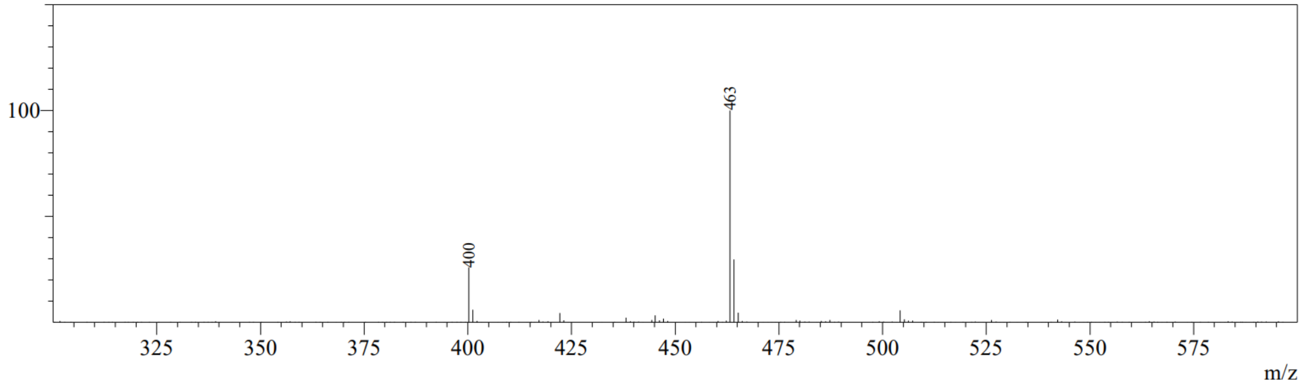

Spectrum Mode:Averaged 1.156-1.170(348-352) Base Peak:398(2096431)
1.156-1.170(348-352)
Negative

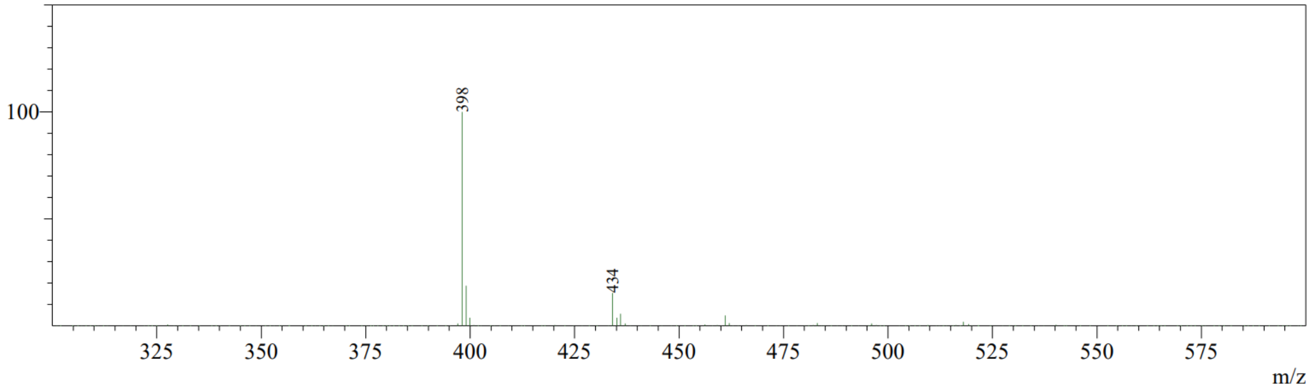

Figure S78. LC–MS spectra of compound **10a**

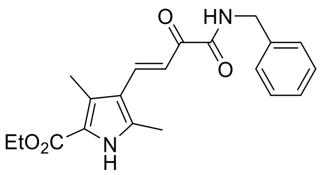

Exact Mass: 354,16
Molecular Weight: 354,41

Spectrum Mode:Averaged 1.510-1.530(303-307) Base Peak:355(603527)
1.510-1.530(303-307)
Positive

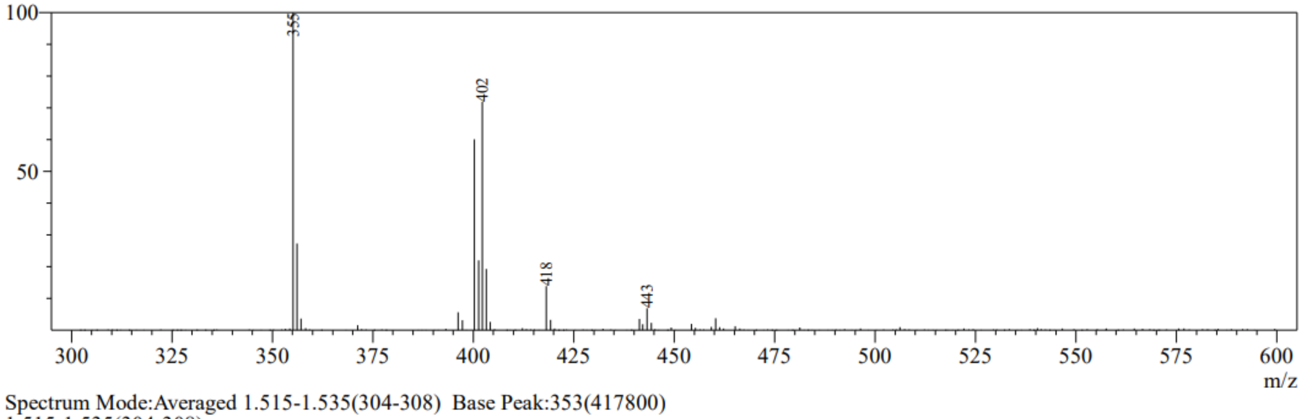

Spectrum Mode:Averaged 1.515-1.535(304-308) Base Peak:353(417800)
1.515-1.535(304-308)
Negative

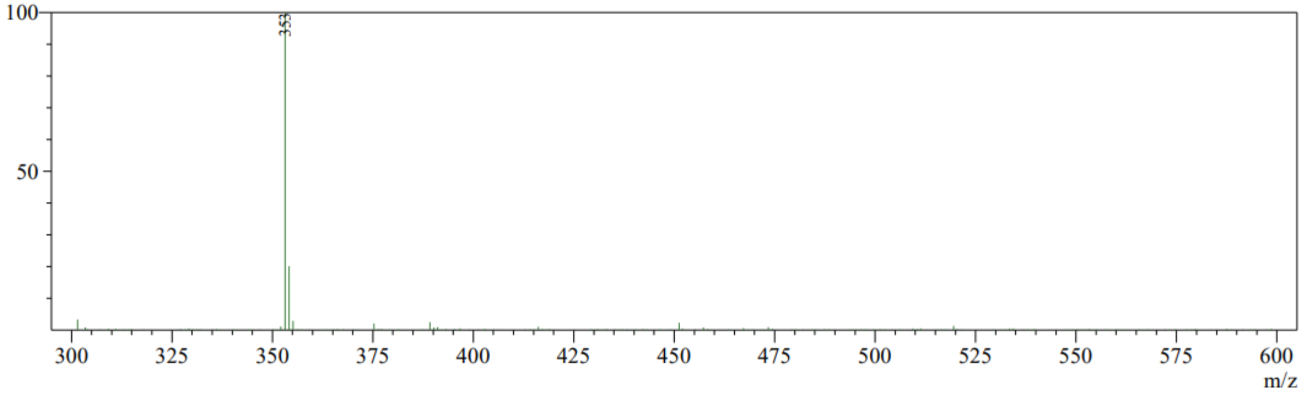

Figure S79. LC–MS spectra of compound **10b**

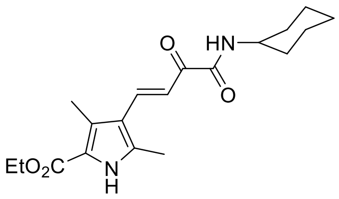

Exact Mass: 346,19
Molecular Weight: 346,43

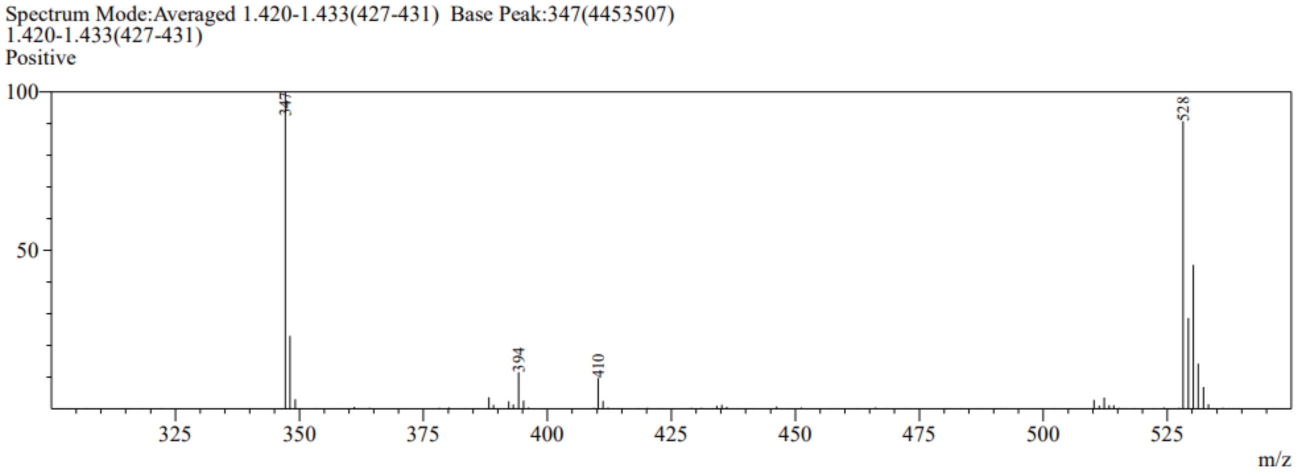

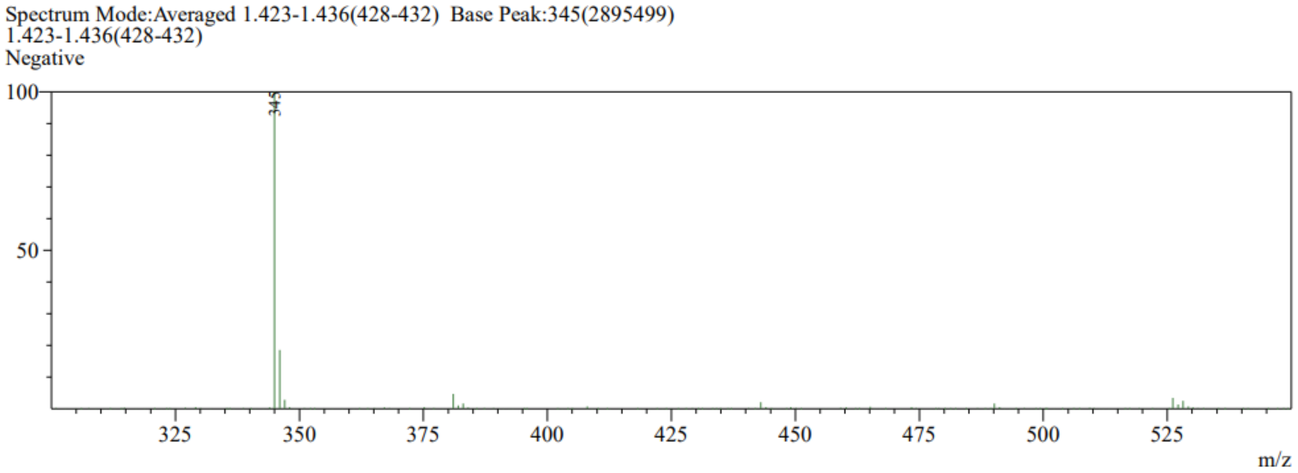

Figure S80. LC–MS spectra of compound **10c**

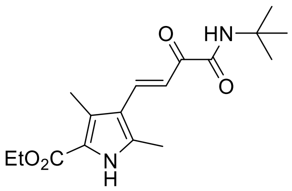

Exact Mass: 320,17
Molecular Weight: 320,39

Spectrum Mode:Averaged 1.333-1.347(401-405) Base Peak:384(481226)
1.333-1.347(401-405)
Positive

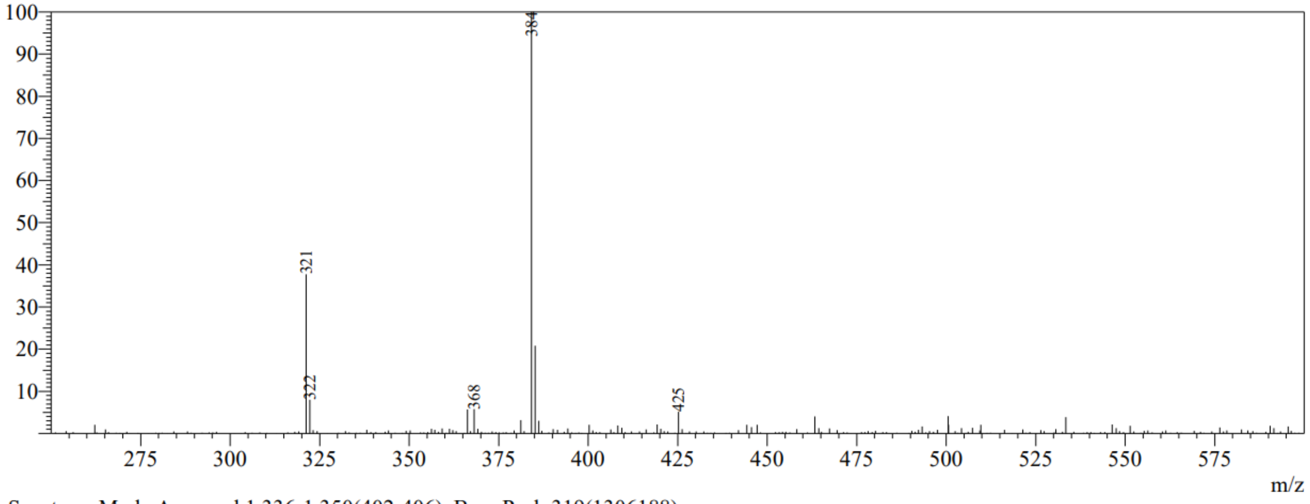

Spectrum Mode:Averaged 1.336-1.350(402-406) Base Peak:319(1306188)
1.336-1.350(402-406)
Negative

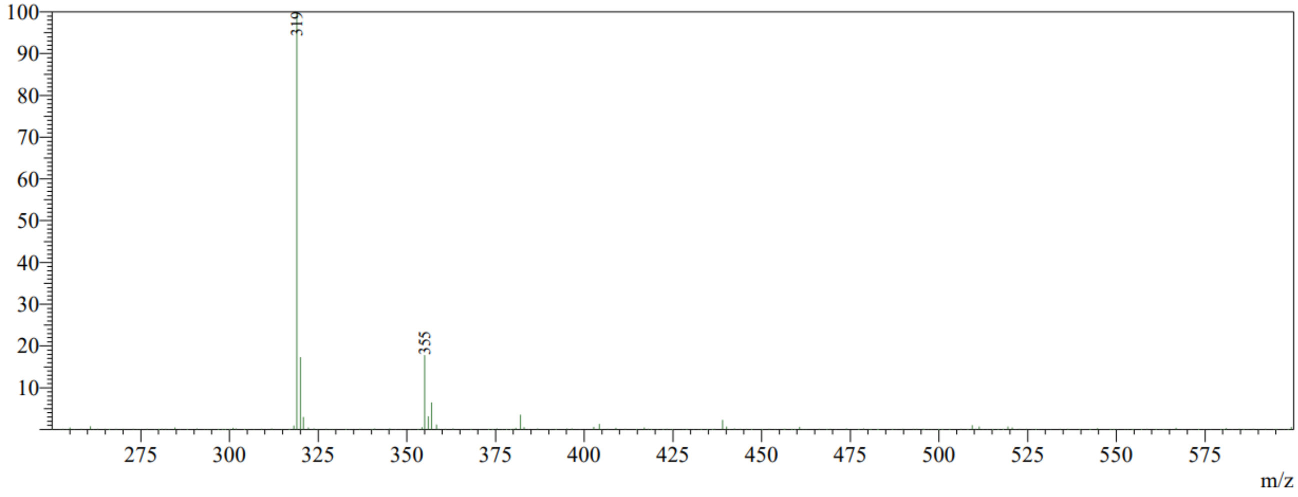

Figure S81. LC–MS spectra of compound **10d**

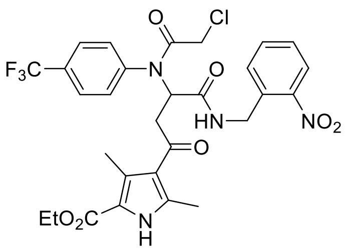

Exact Mass: 636,16
Molecular Weight: 637,01

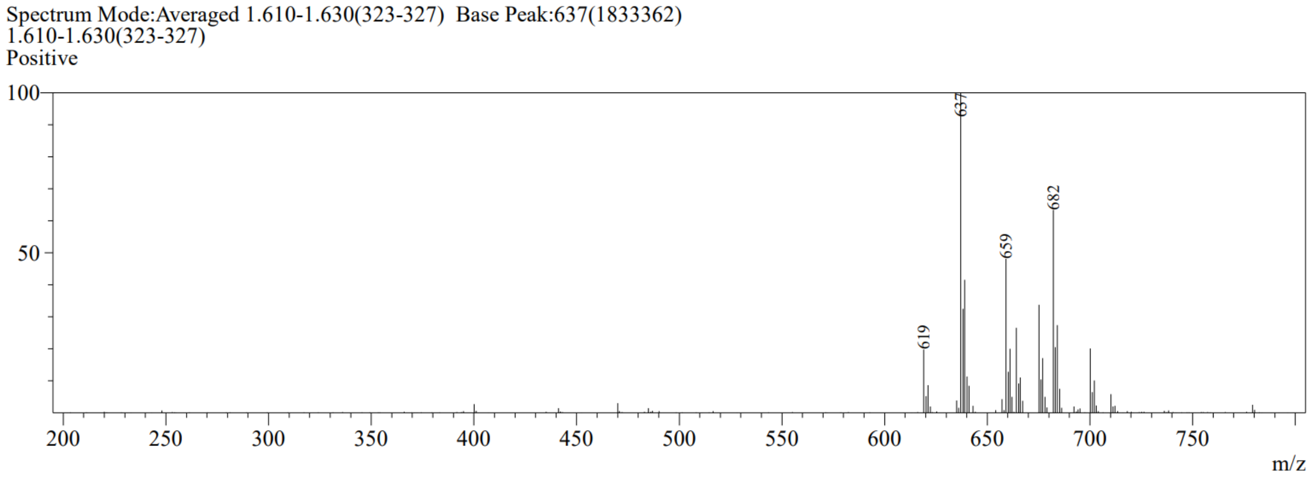

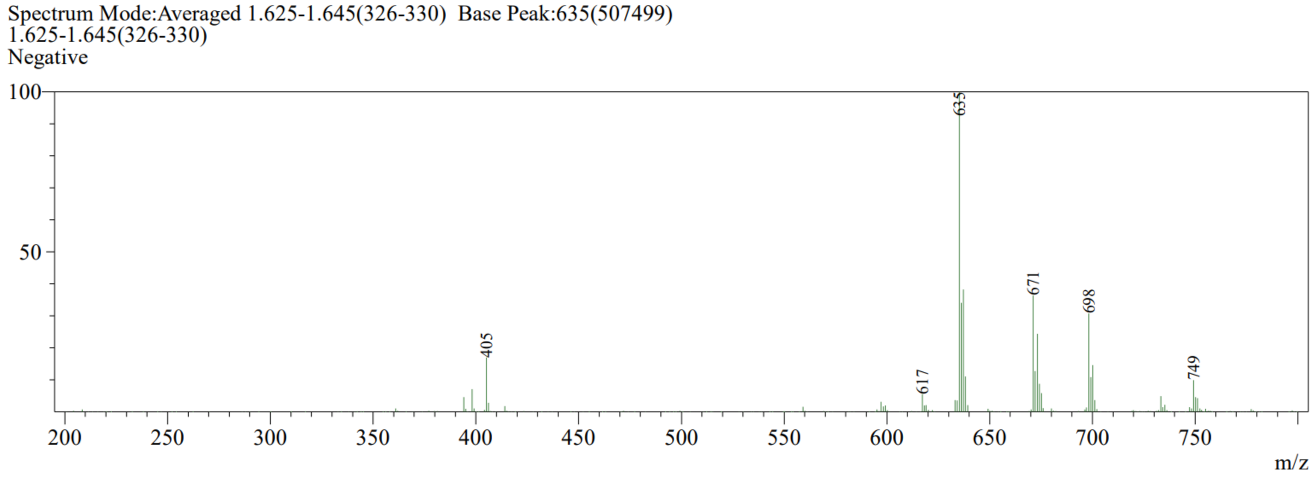

Figure S82. LC–MS spectra of compound **12a**

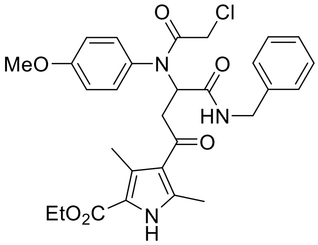

Exact Mass: 553,20
Molecular Weight: 554,04

Spectrum Mode:Averaged 1.890-1.910(379-383) Base Peak:536(3246209)
BG Mode:Calc Segment 1 - Event 1

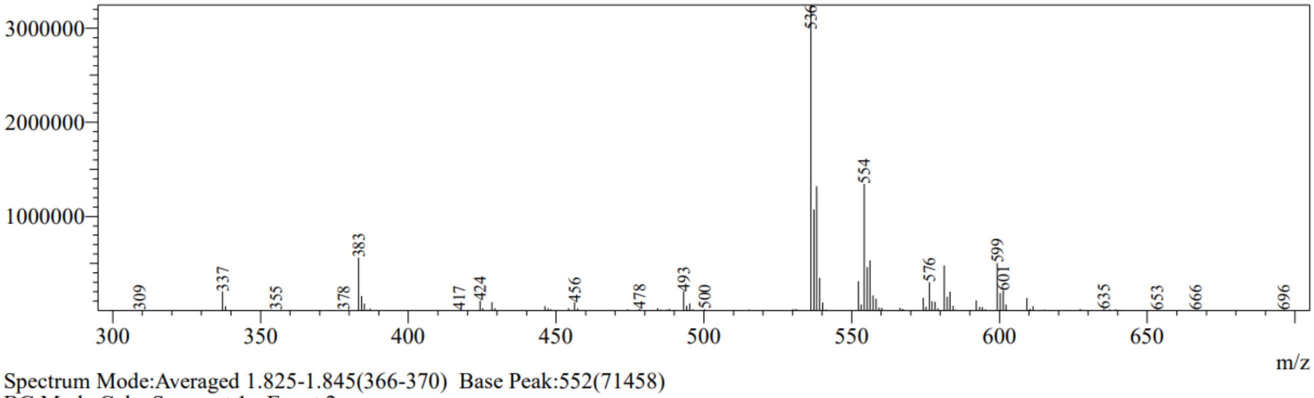

Spectrum Mode:Averaged 1.825-1.845(366-370) Base Peak:552(71458)
BG Mode:Calc Segment 1 - Event 2

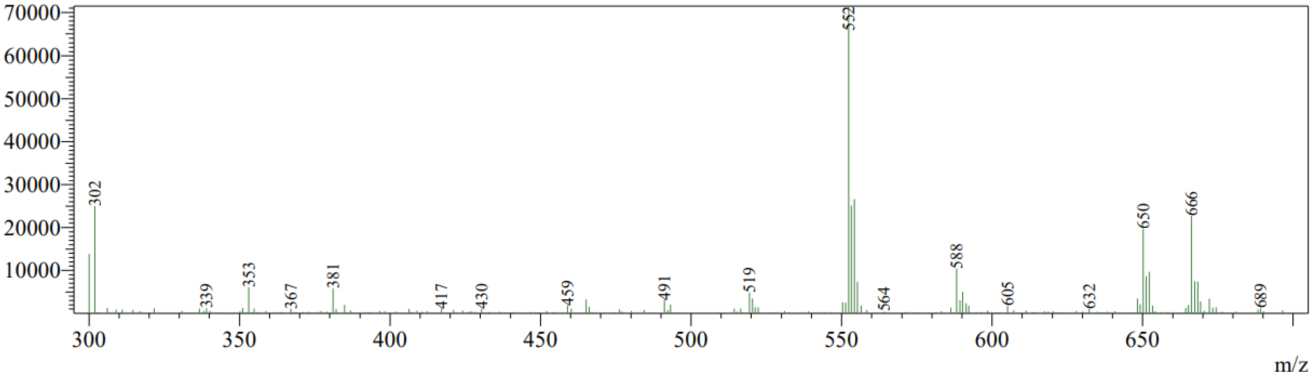

Figure S83. LC–MS spectra of compound **12b**

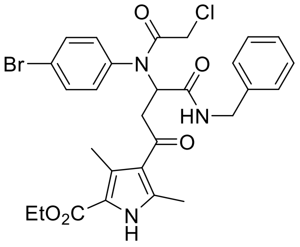

Exact Mass: 601,10
Molecular Weight: 602,91

Spectrum Mode:Averaged 2.130-2.150(427-431) Base Peak:602(2428274)
2.130-2.150(427-431)
Positive

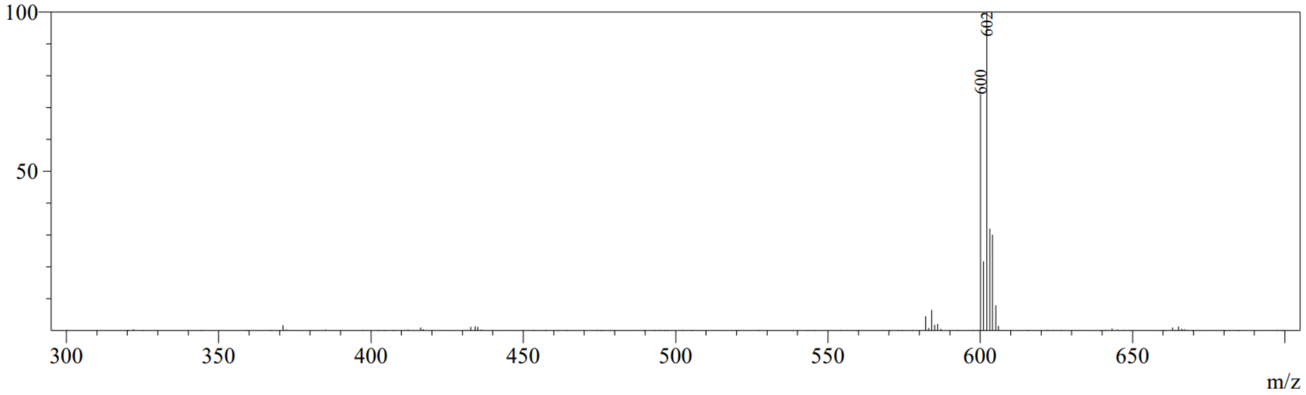

Spectrum Mode:Averaged 2.115-2.135(424-428) Base Peak:636(146161)
2.115-2.135(424-428)
Negative

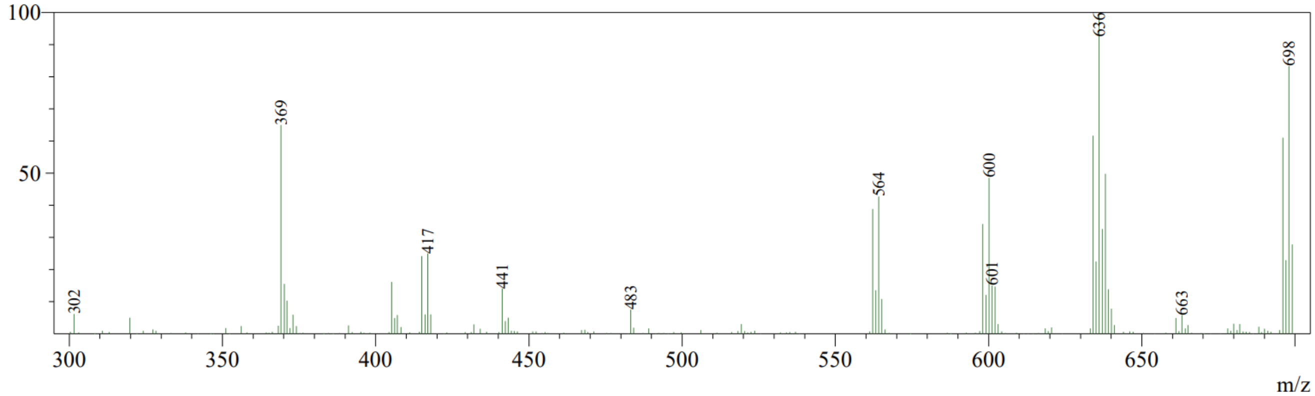

Figure S84. LC–MS spectra of compound **12c**

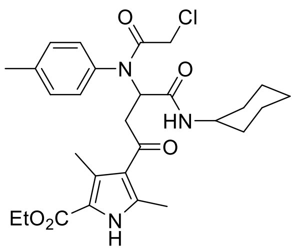

Exact Mass: 529,23
Molecular Weight: 530,06

Spectrum Mode:Averaged 2.540-2.560(509-513) Base Peak:631(1708764)
2.540-2.560(509-513)
Positive

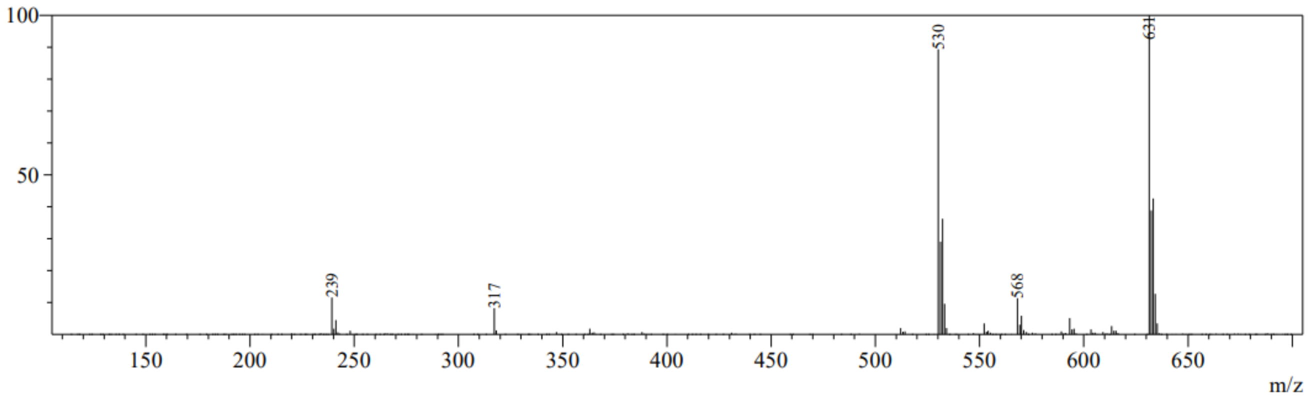

Spectrum Mode:Averaged 2.545-2.565(510-514) Base Peak:528(135940)
2.545-2.565(510-514)
Negative

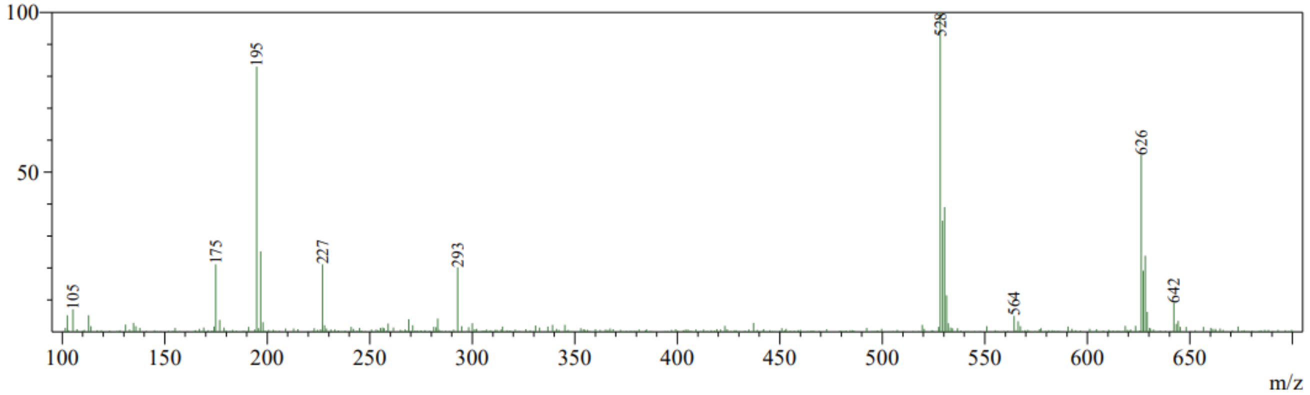

Figure S85. LC–MS spectra of compound **12d**

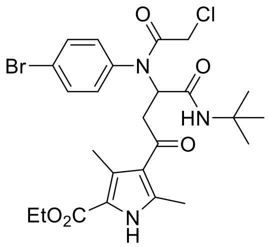

Exact Mass: 567,11
Molecular Weight: 568,89

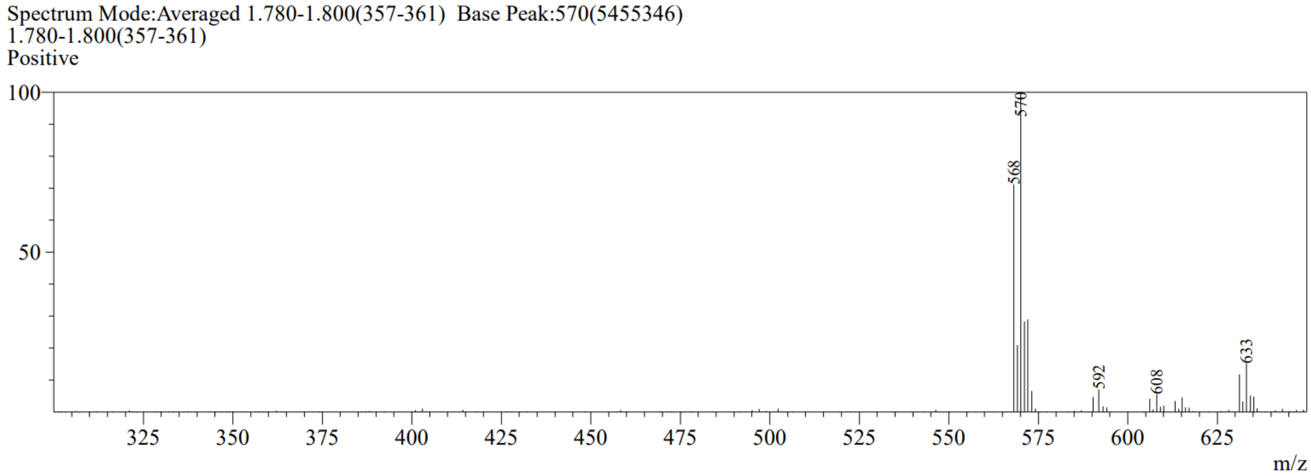

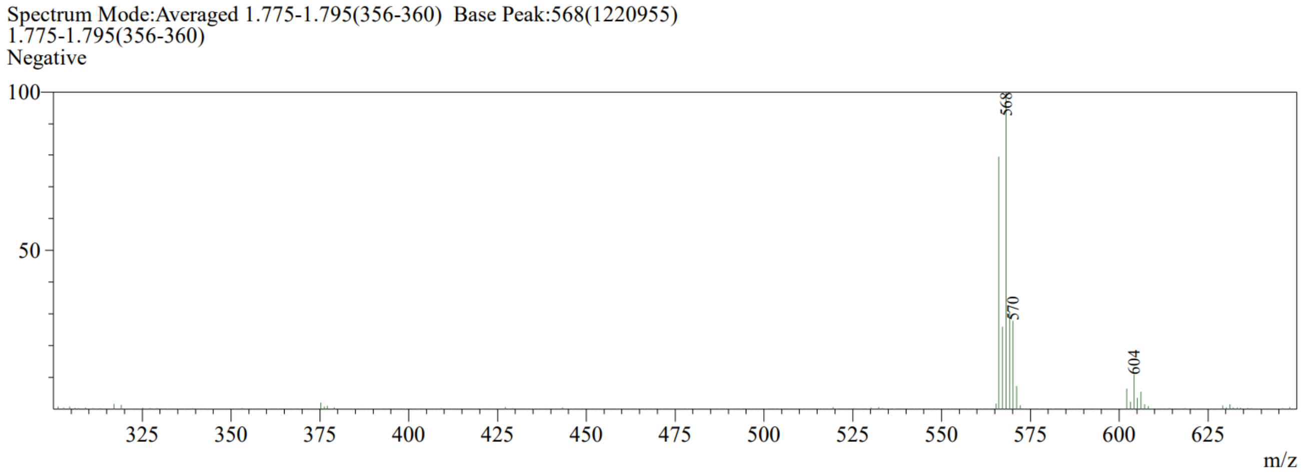

Figure S86. LC–MS spectra of compound **12e**

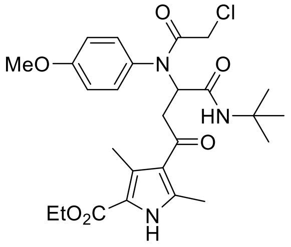

Exact Mass: 519,21
Molecular Weight: 520,02

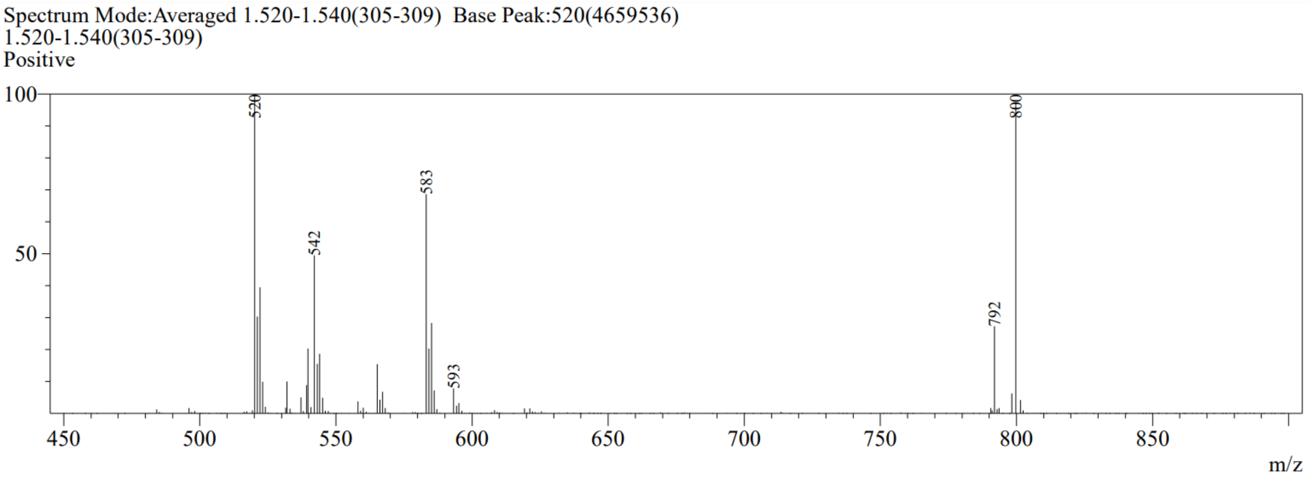

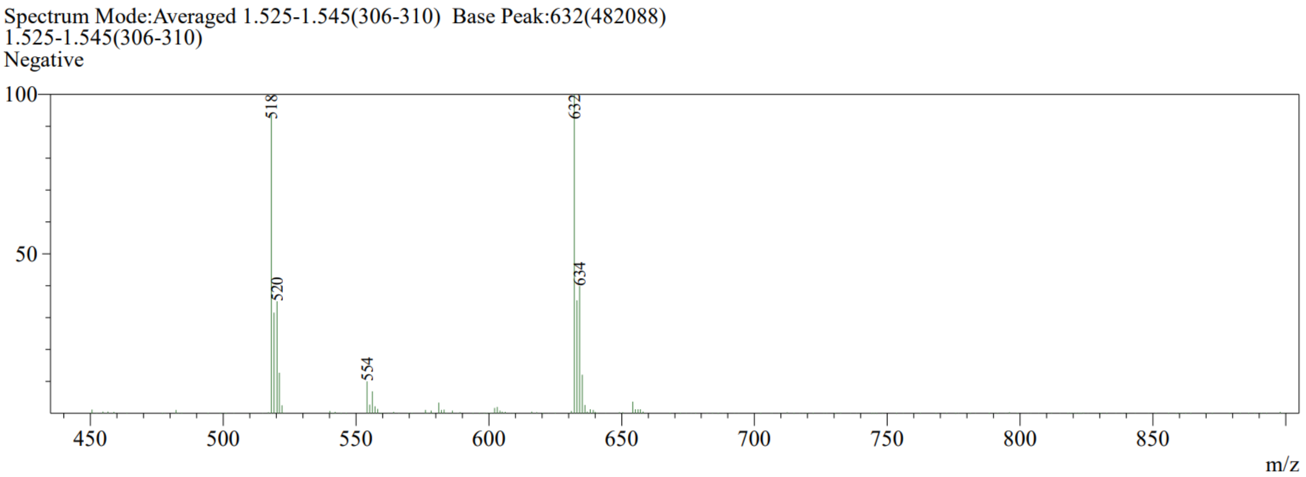

Figure S87. LC–MS spectra of compound **12f**

X-ray diffraction study of compounds 8c, 10d, and 12e

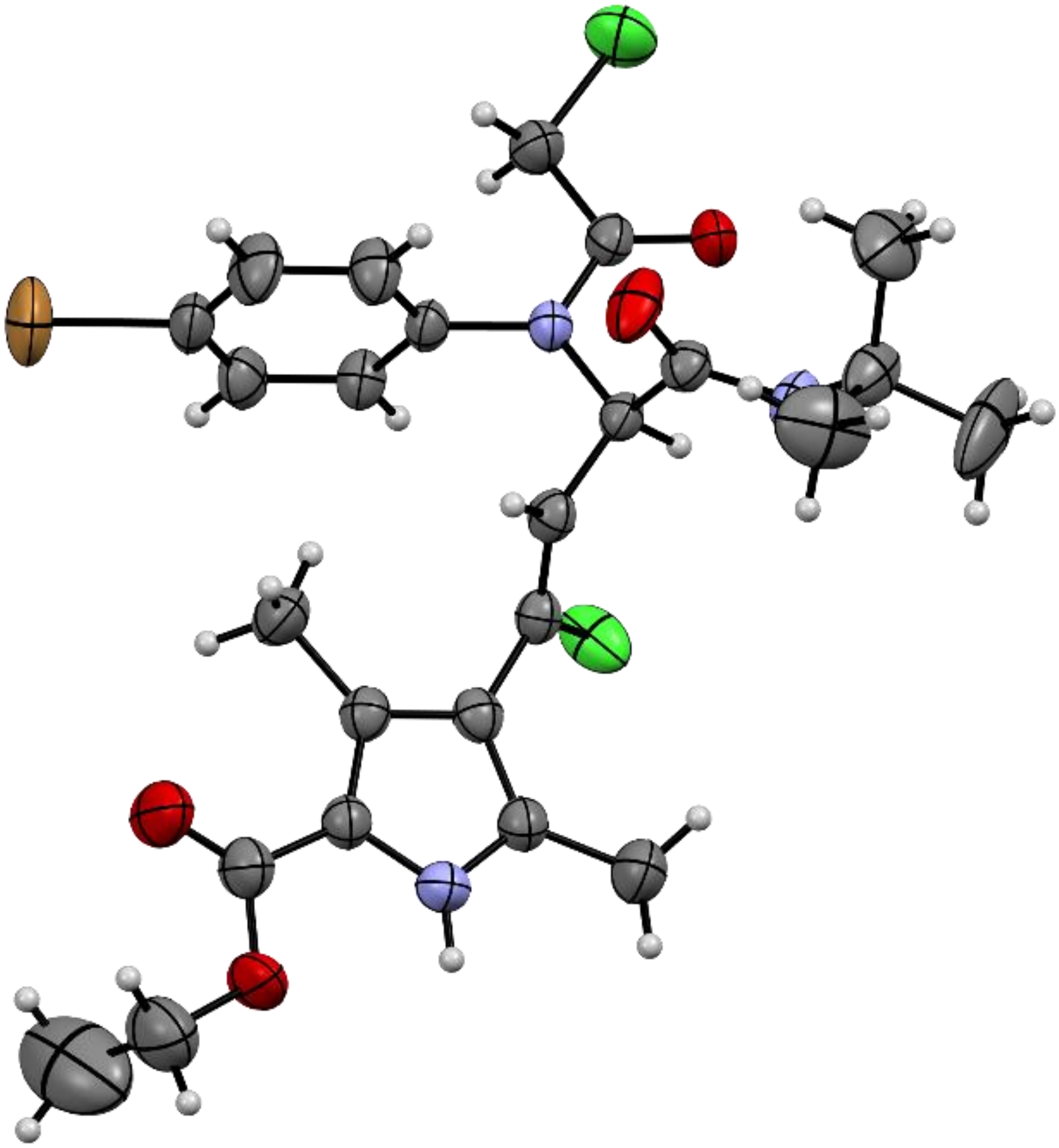

Figure S88. Molecular structure of compound **8c** according to the X-ray diffraction data. Thermal displacement ellipsoids are shown at 50% probability level.

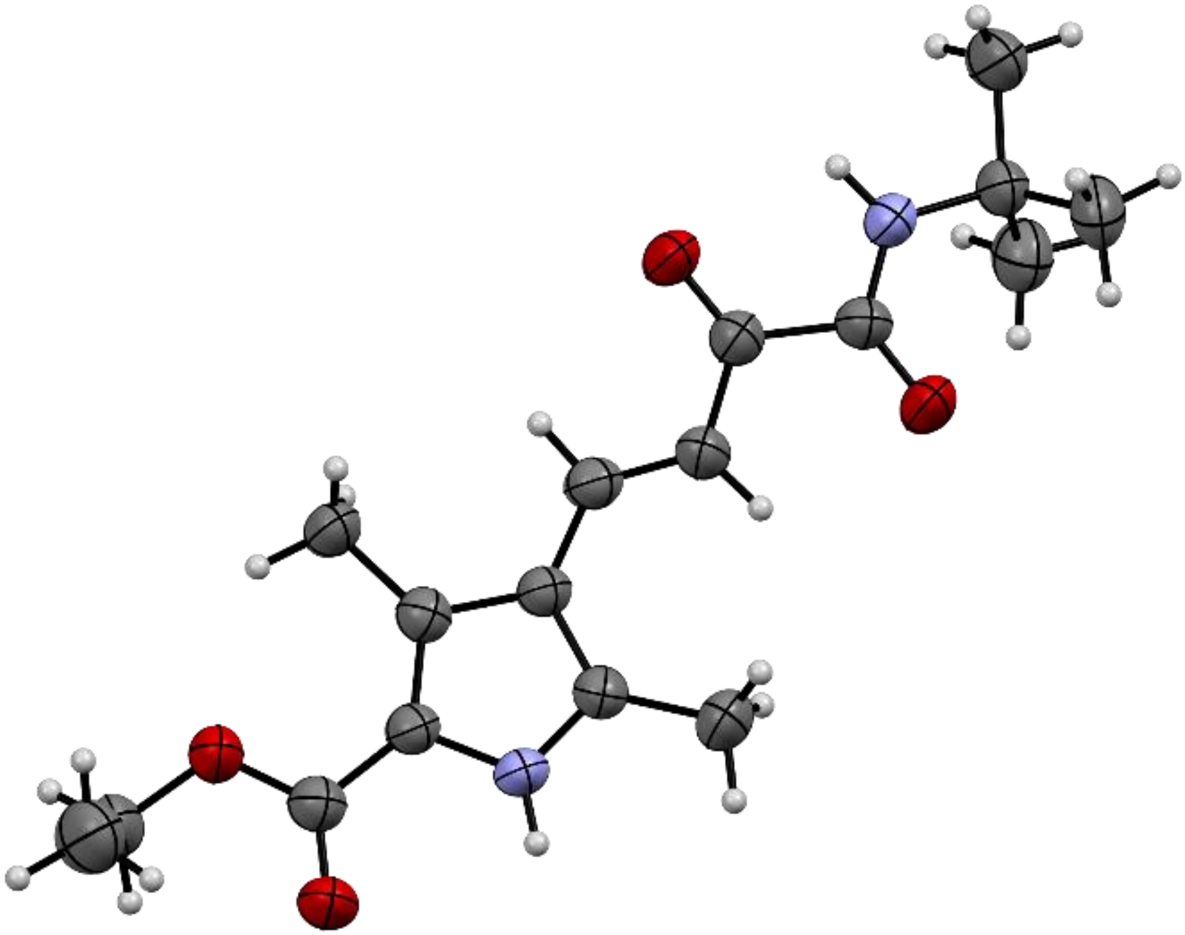

Figure S89. Molecular structure of compound **10d** according to the X-ray diffraction data. Thermal displacement ellipsoids are shown at 50% probability level.

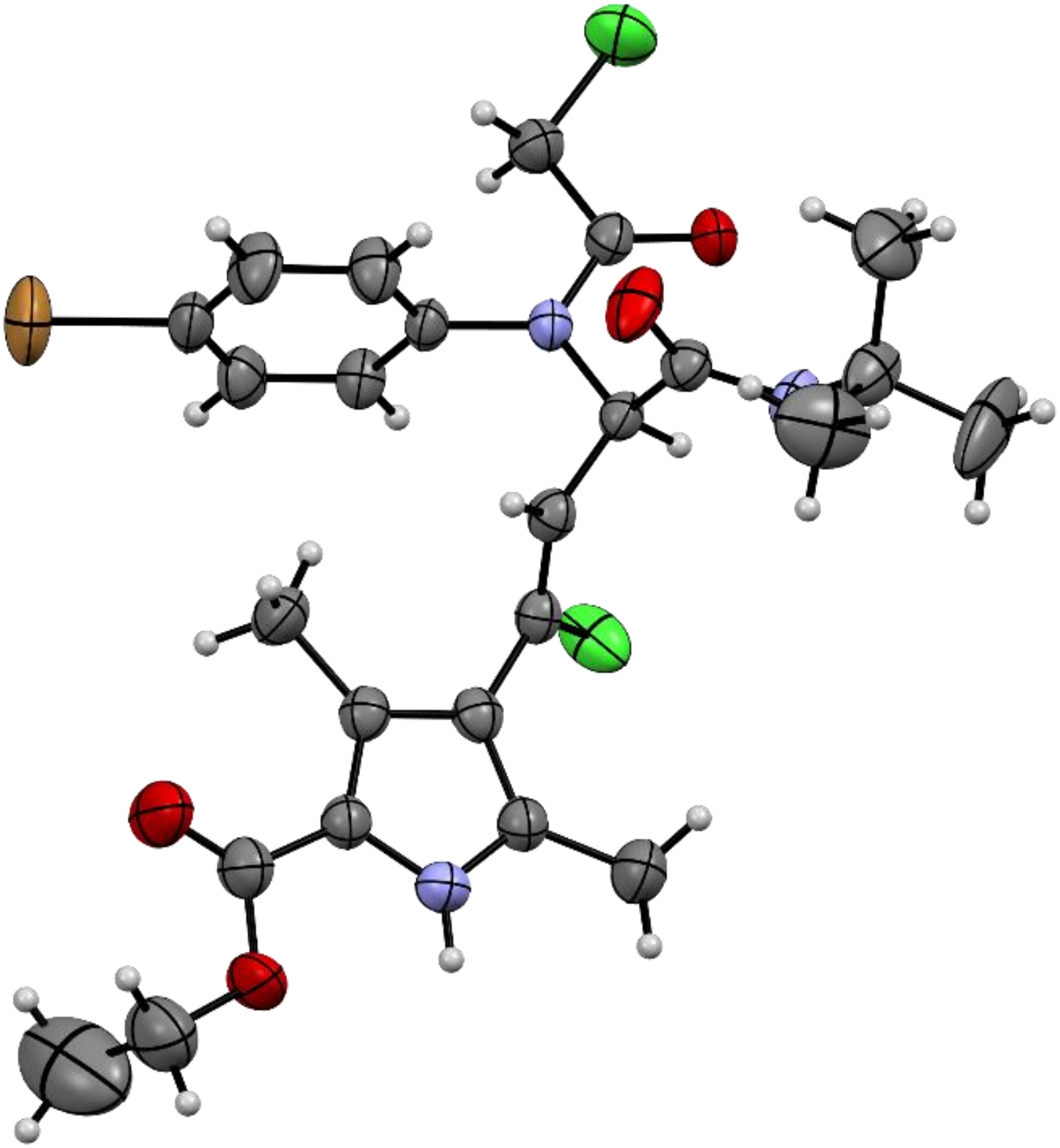

Figure S90. Molecular structure of compound **12e** according to the X-ray diffraction data. Thermal displacement ellipsoids are shown at 50% probability level.

References

1. Sheldrick G.M. // *Acta Crystallogr., Sect. A*, **2008**, A64, p.112-122.
